# Identification of the Genes Required for the Culture of *Liberibacter crescens*, the Closest Cultured Relative of the *Liberibacter* Plant Pathogens

**DOI:** 10.3389/fmicb.2016.00547

**Published:** 2016-04-20

**Authors:** Kin-Kwan Lai, Austin G. Davis-Richardson, Raquel Dias, Eric W. Triplett

**Affiliations:** Microbiology and Cell Science Department, Institute of Food and Agricultural Sciences, University of FloridaGainesville, FL, USA

**Keywords:** *Liberibacter*, transposon mutagenesis, essential genes, citrus greening disease, Huanglongbing, HLB, citrus

## Abstract

Here Tn5 random transposon mutagenesis was used to identify the essential elements for culturing *Liberibacter crescens* BT-1 that can serve as antimicrobial targets for the closely related pathogens of citrus, *Candidatus* Liberibacter asiaticus (Las) and tomato and potato, *Candidatus* Liberibacter solanacearum (Lso). In order to gain insight on the virulence, metabolism, and culturability of the pathogens within the genus *Liberibacter*, a mini-Tn5 transposon derivative system consisting of a gene specifying resistance to kanamycin, flanked by a 19-base-pair terminal repeat sequence of Tn5, was used for the genome-wide mutagenesis of *L. crescens* BT-1 and created an insertion mutant library. By analyzing the location of insertions using Sanger and Illumina Mi-Seq sequencing, 314 genes are proposed as essential for the culture of *L. crescens* BT-1 on BM-7 medium. Of those genes, 76 are not present in the uncultured *Liberibacter* pathogens and, as a result, suggest molecules necessary for the culturing these pathogens. Those molecules include the aromatic amino acids, several vitamins, histidine, cysteine, lipopolysaccharides, and fatty acids. In addition, the 238 essential genes of *L. crescens* in common with *L. asiaticus* are potential targets for the development of therapeutics against the disease.

## Introduction

Citrus greening disease, also known as Huanglongbing disease (HLB), is a devastating disease of citrus worldwide. A phloem-restricted α-proteobacterium, namely *Candidatus* Liberibacter asiaticus (Las), is the casual agent of the disease (Bové, 2006; Tyler et al., 2009). Las is found in North America and Asia while two other species, *Candidatus* Liberibacter africanus (Laf) and *Candidatus* Liberibacter americanus (Lar), are found in Africa and Brazil respectively. HLB is naturally transmitted by the psyllid *Diaphorina citri* and is capable of infecting all known commercial varieties of citrus. The highest titer of Las is found in the peduncle and is unequally distributed in the phloem of infected plants (Tatineni et al., 2008). Some of the HLB symptoms include yellow shoots, asymmetric blotchy leaf mottle, thicken leaves, veins enlargement, twig dieback, small off-taste fruits, and premature fruit drops. Starch accumulation occurs in the phloem, which damages the phloem and prevents nutrients transport. The infected trees eventually die when the conditions are too severe (Wang and Trivedi, 2013). Currently there are no cures for HLB. Thermotherapy and chemotherapy are recently proposed but still under evaluation (Pagliai et al., 2015; Canales et al., 2016; Fan et al., 2016). Methods on controlling the HLB infection are based on chemical treatments on the psyllid vectors and complete removal of the infected trees (Gottwald et al., 2007). However, these preventative methods are not as efficient as the spreading of the disease.

During the past few years, genomes of several *Liberibacter* species were sequenced (Duan et al., 2009; Lin et al., 2011, 2015; Wulff et al., 2014). Genes involved in transcriptional regulation, major metabolic pathways, secretion/transportation system, motility and signal transduction were predicted as putative virulence factors (Cong et al., 2012; Yan et al., 2013). However, the inability to culture any of the *Liberibacter pathogens* limits our understanding of the mechanism of infection and delays the development of treatments for citrus greening disease. *Liberibacter crescens* BT-1 is a Gram-negative, rod-shaped, α-proteobacterium isolated from mountain papaya (Leonard et al., 2012; Fagen et al., 2014a). It is the closest cultured relative of the causal agents of citrus greening disease (Las, Laf, and Lar), and *Ca*. L. solanacearum (Lso), the causal agent of zebra chip on potato and tomato yellows (Fagen et al., 2014a,b). To date, a plant host for *L. crescens* has not been identified. In addition, *L. crescens* has not experienced far less genome reduction than the *Liberibacter* pathogens. These two observations suggest that *L. crescens* may not be a plant pathogen. *L. crescens* can serve as a model to understand the mechanism of infection and culturing of *Liberibacter* pathogens.

Genome-wide saturation mutagenesis has been used in several studies to identify the essential genes and pathways critical for the survival or pathogenesis of bacteria in culture or in association with a host (Akerley et al., 2002; Forsyth et al., 2002; Langridge et al., 2009; Moule et al., 2014). An essential gene is defined as one whose loss is lethal to an organism under the growth conditions of interest. Identifying the essential gene set allows us to not only understand the nutritional requirement for growth but also suggests potential targets for antimicrobial drug development (Lee et al., 2015).

Transposon mutagenesis is a powerful tool for producing randomized gene mutation in bacterial genomes. It has been utilized for the study of bacterial pathogenesis extensively and has been used to identify virulence factors in different bacteria (Mei et al., 1997; Hava and Camilli, 2002). Bacterial clones containing transposon insertions that significantly reduce fitness for *in vitro* growth will not survive. Using the genomic DNA from the mutant pools, Illumina nucleotide sequencing can be used to amplify from the transposon and sequence into the adjacent target DNA to identify the insertion sites in the genome. The distribution of transposon insertion sites thus provides a map of the genes and other elements essential for *in vitro* culturing, which can also serve as antimicrobial targets for the closely related pathogens (Miesel et al., 2003).

Here, the Tn5 transposon was used to mutagenize *L. crescens* BT-1 in order to predict gene essentiality. We believe that the predicted essential genes will be extremely useful for expanding the capacity of *L. crescens* BT-1 as a model organism to develop treatments for citrus greening disease.

## Materials and methods

### Bacterial strains and culture conditions

*Escherichia coli* donor strain SM17-1λpir carrying pUT-miniTn5Km1 plasmid (de Lorenzo et al., 1990) was cultured on Luria-Bertani (LB) liquid medium supplemented with 25 μg mL^−1^ kanamycin at 37°C with agitation at 250 rpm. *L. crescens* BT-1 was cultured on liquid BM7 medium (Fagen et al., 2014a) at 27°C with moderate agitation (125 rpm).

### Preparation of *L. crescens* BT-1 electrocompetent cell

A single colony of *L. crescens* BT-1 was picked from BM7 agar plate and grown in 2 mL of BM7 medium until OD_600_ reached 0.3–0.4. It was used to inoculate 30 mL of BM7 medium. Cells were incubated on ice for 15 min and harvested by centrifugation (1400 x g, 15 min, 4°C) when OD_600_ reached 0.3–0.4. Cells were washed 2 times with 30 mL of ice-cold 10% glycerol. The competent cells were resuspended in 300 μL of ice-cold 10% glycerol and stored at −80°C in small aliquots.

### Electro-transformation of *L. crescens* BT-1 using EZ-Tn5 transponsome

EZ-Tn5 < KAN-2>Tnp Transposome (20 ng, Epicentre, USA) was mixed with 50 μL of *L. crescens* BT-1 competent cells and transferred to a pre-chilled 0.1 cm gene pulser cuvette (Bio-Rad, USA). Electro-transformation was carried out using Bio-Rad Gene Pulser Xcell™ Electroporation System set to 1.8 kV, 25 μF, and 200 ω. Cells were immediately resuspended into 2 mL of BM7 medium and grown for 24 h. Cells were harvested by centrifugation (1400 x g, 5 min) and spread on BM7 agar plate supplemented with 5 μg mL^−1^ kanamycin. Cells were incubated at 27°C until colonies formed. Individual colonies were picked and inoculated into 2 mL of BM7 medium. Cells were harvested when OD_600_ reached 0.3–0.4 and suspended in 25% glycerol for storage and subsequent DNA extraction.

### Transposon mutagenesis of *L. crescens* BT-1 using pUTminiTn5km1

The pUTminiTn5Km1 transposon was delivered into *L. crescens* BT-1 by bi-parental mating with the *E. coli* donor strain SM17-1λpir carrying pUTminiTn5Km1 plasmid. Ten milliliter of *L. crescens* BT-1 culture (OD_600_ ~ 0.3) and 1 mL of *E. coli* donor culture were harvested by centrifugation. Cells were washed 2 times with 1 mL of 10 mM MgSO_4_ and were resuspended in the same volume of 10 mM MgSO_4_. One microliter of the washed *E. coli* donor cells was added to the 1 mL of the washed *L. crescens* BT-1 cells. The mixture was filtered through a sterile 25 mm-diameter Millipore type HA 0.45 μm filter (EMD Millipore, USA). The filter was transferred to a BM7 agar plate and incubated for 18 h at 27°C. The filter was transferred to 5 mL of BM7 supplemented with 10 mM MgSO_4_ to release cells from the filter and into the medium. Cells were harvested from 200 μL of the cell suspension by centrifugation and spread on BM7 agar plate supplemented with antibiotics (15 μg mL^−1^ nalidixic acid and 5 μg mL^−1^ kanamycin) to select for *L. crescens* BT-1 containing transposon insertions. Transformed cells were then incubated at 27°C until cell layer formed. Cells were scraped off the plate and grown in 5 mL of BM7 medium supplemented with antibiotics (15 μg mL^−1^ nalidixic acid and 5 μg mL^−1^ kanamycin). Next, cells were harvested at OD_600_ 0.3–0.4 and then stored at −80°C in 25% glycerol.

### Genomic DNA extraction of *L. crescens* BT-1 Tn5 mutants

Genomic DNA of Tn5 mutants were extracted using hexadecyltrimethylammonium bromide (CTAB) phenol chloroform extraction method. Cell pellets were resuspended in 567 μL TE buffer (10 mM Tris-HCl pH 8, 1 mM EDTA), 30 μL 10% SDS, and 3 μL proteinase K (20 mg mL^−1^ in TE buffer) and incubated for 1 h at 37°C. Cells were mixed thoroughly after addition of 100 μL 5 M NaCl and 80 μL of CTAB-NaCl (10% CTAB, 0.7 M NaCl). After the mixture was incubated at 65°C for 10 min, 780 μL of chloroform/isoamyl alcohol (24:1, v/v) was added, gently mixed, and centrifuged at 15,000 x g for 5 min. The supernatant was transferred to a new tube followed by addition of an equal volume of phenol/chloroform/isoamyl alcohol (25:24:1, v/v/v) which was then, gently mixed, and centrifuged at 15000 x g for 5 min. To the supernatant, 0.6 volume of isopropanol was added, gently mixed, and stored at −20°C overnight. The DNA pellet was harvested by centrifugation at 15,000 x g for 5 min. The pellet was washed with 700 μL of 70% ethanol and centrifuged. The ethanol was discarded. The pellet was allowed to air dry for 10 min and rehydrated in 100 μL of water. The concentration of DNA was determined with a qubit fluorometer (Life Technologies, USA).

### Determination of EZ-Tn5 *L. crescens* BT-1 insertion site

Single primer polymerase chain reaction (sp-PCR) was used to identify the EZ-Tn5 insertion site (Karlyshev et al., 2000). Primer inv-1 (ATGGCTCATAACACCCCTTGTATTA) or inv-2 (GAACTTTTGCTGAGTTGAAGGATCA) was used in the PCR with GoTaq® G2 DNA Polymerase (Promega, USA). DNA was amplified with 5 min at 95°C followed by 30 cycles of 30 s at 95°C, 30 s at 56°C, and 30 s at 72°C; 30 cycles of 30 s at 95°C, 30 s at 30°C, and 30 s at 72°C; 30 cycles of 30 s at 95°C, 30 s at 56°C, and 2 min at 72°C; and 10 min at 72°C. The PCR products were purified using Qiagen QIAquick PCR purification kit. Sanger sequencing was performed using primer FP1 (ACCTACAACAAAGCTCTCATCAACC, for inv-2 PCR product) or RP1 (GCAATGTAACATCAGAGATTTTGAG, for inv-1 PCR product). The insertion sites were determined by homology to the *L. crescens* genome using NCBI Blastn. A total of 2070 high quality sequences of the EZ-Tn5 insertion sites are provided as Table S1.

### Illumina sequencing of pUTminiTn5km1 *L. crescens* BT-1

Equal amounts of genomic DNA from each batch of pUTminiTn5Km1 *L. crescens* BT-1 mutants were pooled together. Pooled DNA (5 μg) was fragmented to an average size of 300 bp by ultrasonication. The fragments were further size selected to 250–350 bp by gel excision. The fragmented DNA was purified, end repaired, and A-tailed according to Illumina library preparation protocol. Two single-stranded primers Ind_Ad_T (ACACTCTTTCCCTACACGACGCTCTTCCGATC^*^T) and Ind_Ad_B (pGATCGGAAGAGCGGTTCAGCAGGAATGCCGAGACCGATCTC) were annealed together to form the adapter. The adapter was ligated to the fragmented DNA. The DNA was quantified with an Agilent NAD1000 chip and qubit fluorometer. PCR was preformed to enrich the fragments containing the transposon insertion sites using primer RInV3.3 (CAAGCAGAAGACGGCATACGAGATCGGTACACTCTTTCCCTACACGACGCTCTTCCGATCT) and primer MiniTn5_P5_3pr_3 (AATGATACGGCGACCACCGAGATCTACACCTAGGCtGCGGCtGCACTTGTG) with Phusion® High-Fidelity DNA Polymerase (NEB, USA). PCR amplification conditions included 2 min at 98°C; 22 cycles of 30 s at 98°C, 30 s at 65°C, 30 s at 72°C; and 10 min at 72°C. Amplified library was cleaned using Qiagen QIAquick PCR purification kit. DNA fragment library was sequenced using 1 × 50 cycles Illumina flow cell on an Illumina MiSeq sequencer with a custom Tn5 sequencing primer Tn5_ill_seq (CCTAGGCtGCGGCtGCACTTGTG), which is designed such that the first 12 bp of each read is the end 12 bp of the transposon sequence.

### Analysis of the pUTminiTn5km1 sequence data

The 12-bp transposon tag (TATAAGAGTCAG) was trimmed from the raw Illumina sequence data. Reads were mapped to the *L. crescens* BT-1 chromosome (GI: 430799321) using CLC Workbench version 6.5 (CLC bio, USA). A custom script was used to count the number of insertions by gene and is available online (https://github.com/triplett/lai-2015). The total number of high quality sequences obtained from these insertion events was 16,185,251. Raw sequence data can be accessed at NCBI sequence read archive with study accession number SRP065838.

### Statistical analysis

The probability of having 1 insertion in each of the genes was calculated using the poisson distribution equation (Watabe et al., 2014) as follow:

N=ln(1−P)/ln(1−f)

*N* is the number of insertions needed; *P* is the probability of having 1 insertion in each of the genes; *f* is the frequency of insertion, which is the average gene size divided by the genome size. The probability of finding 1 insertion within a particular gene was calculated using neutral base-pair model (Laia et al., 2009):

$$P=1-(1-\frac{X}{G})n$$

*n* is the number of insertions; *P* is the probability of finding 1 insertion within a particular gene; *X* is the length of the gene; *G* is the length of the genome.

### Gene comparison and functional categorization of essential genes

Gene annotations were acquired from NCBI. Essential gene homologs were identified by manual NCBI BLAST searches. Genes were classified into functional groups/subsystems using Rapid Annotation Subsystem Technology (RAST; Overbeek et al., 2014).

## Results

### Tn5 transposon mutagenesis of *L. crescens* BT-1

*L. crescens* BT-1 genome has 1379 open reading frames and 54 RNA-coding genes (Leonard et al., 2012). It has a genome size of 1504659 bp and an average gene length of 895.3 bp. Poisson distribution equation was used to estimate the number of insertions or mutants required to saturate the genome to have at least 1 insertion per gene as seen in Table 1. Two methods were used for the Tn5 transposon mutagenesis of *L. crescens* BT-1: electro-transformation with the commercial EZ-Tn5 Transposome (EZ-Tn5) and bi-parental mating with the *E. coli* donor strain SM17-1λpir harboring pUTminiTn5Km1 plasmid (pUT-Tn5). Approximately 50–100 colonies (2.5 × 10^3^–5 × 10^3^ transformants/μg of transposon DNA) were formed in each successful electro-transformation with the EZ-Tn5 method. A total of 2205 EZ-Tn5 colonies were isolated. Tn5 insertion sites were identified in 2070 (93.9%) EZ-Tn5 isolated mutants using sp-PCR and Sanger sequencing. Of those, 188 unique insertions were mapped to intergenic regions and 1355 unique insertions were mapped to 547 ORFs and 5 RNA-coding genes of the *L. crescens* BT-1 genome (Table S2).

**Table 1:** **Probability estimation of having at least 1 insertion per gene in *L. crescens* mutagenesis with poisson distribution equation**.

	***P***	***N***
	0.100	177
	0.500	1165
	0.900	3869
	0.990	7737
	0.999	11606

**Figure 1:**
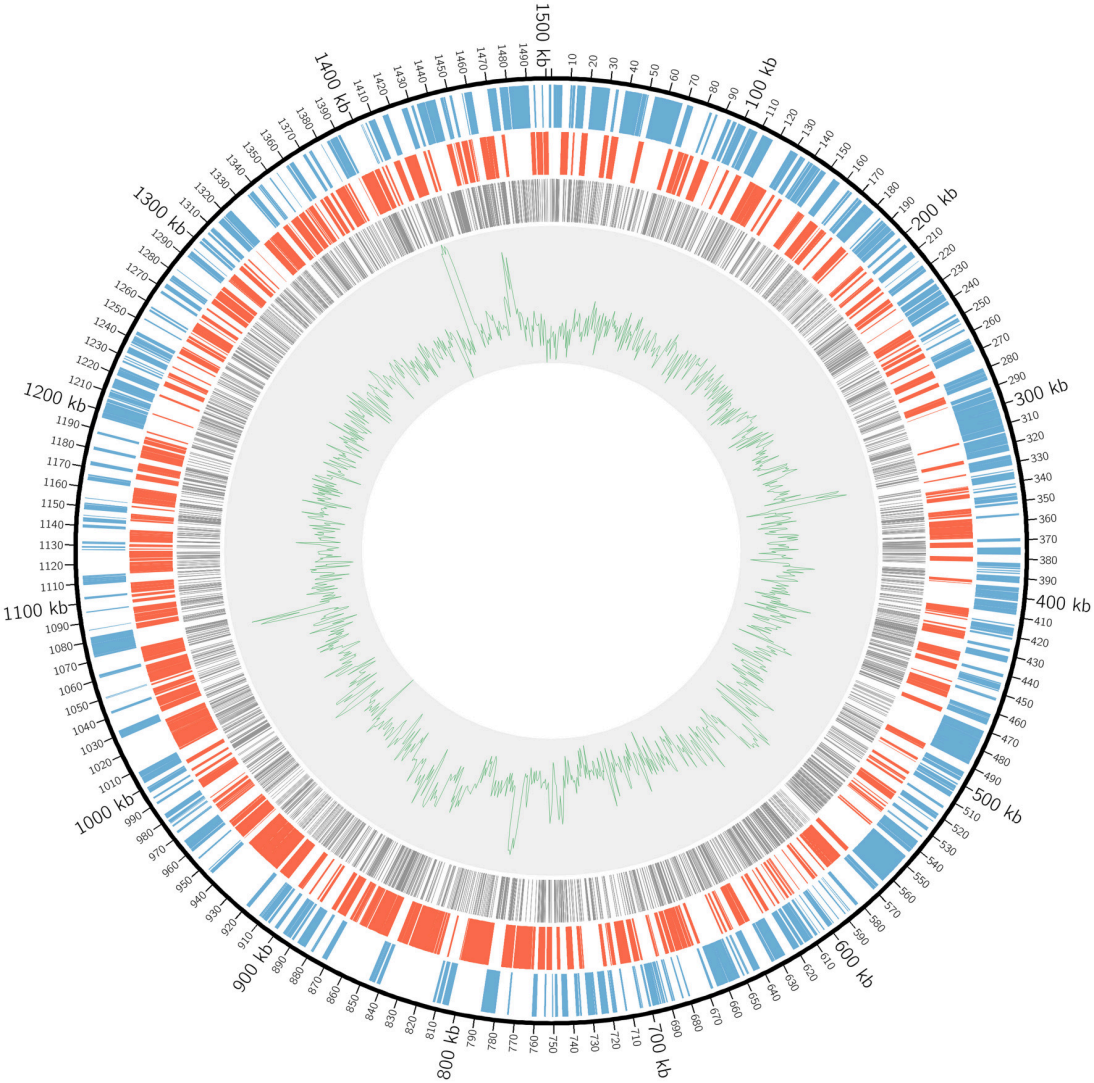
**The distribution of Tn5 insertions in *L. crescens* BT-1 genome**. The black circular line represents the genome sequence with the origin of replication at coordinate zero. The blue bars represent genes transcribed clockwise. The red bars represent genes transcribed counterclockwise. The gray bars represent Tn5 insertions within ORFs. The green line represents the GC plot.

In order to achieve genome-wide saturation mutagenesis of *L. crescens* BT-1, a more robust mutation method was required to generate a larger number of mutants. A thin cell layer was formed on the BM7 agar plate supplemented with nalidixic acid and kanamycin in each successful bi-parental mating with the pUT-Tn5 method. Each batch resulted in 500–1000 colonies as determined by the standard serial dilution plate count method (data not shown). A total of 40 batches of pUT-Tn5 mutants were made independently. Illumina Mi-Seq sequencing analysis identified 4250 unique insertions. Of those 3591 were mapped to 835 ORFs and 11 RNA-coding genes respectively of the *L. crescens* BT-1 genome (Table S3). The distribution of mapped sequence reads across the whole genome is shown in Figure 1.

### Identification of candidate essential genes

Combining both mutation protocols, Tn5 insertions were detected in 994 protein-coding genes and 13 RNA-coding genes from the *L. crescens* genome. Tn5 insertions were not detected in 385 protein-coding and 41 RNA-coding genes, suggesting these genes are essential for the growth of *L. crescens* on BM7 medium (Table S4). A fair number of these essential genes code for small hypothetical proteins. We questioned whether these are truly essential as small gene is less likely to have a mutation than a large gene. Neutral base-pair model allows us to estimate how many genes in this set are truly essential and how many were not hit because of chance alone. It assumes that every base pair in the genome has the same probability of containing an insertion. The gene is less likely to be essential when *P* < 0.5. The neutral base-pair model estimates that 71 protein-coding genes and all 41 RNA-coding genes in the essential gene set were not disrupted because of chance (Table S4), which reduces the essential protein-coding genes set to 314. With 4938 unique insertions within ORFs and 1433 protein- and RNA-coding genes in the genome, there was an average of 3.45 unique insertions per gene. Excluding the 314 protein-coding genes with no insertions, there was an average of 4.41 unique insertions per putatively non-essential gene.

### Functional classification of candidate essential genes

Genes essential for growth make excellent drug target since inhibiting their function will likely lead to lethality. Here, a total of 314 protein-coding genes are proposed as the essential gene set for *L. crescens*. Of the essential protein-coding genes, 74 code for hypothetical genes with no known functions. Most of the annotated essential genes are involved in diverse core cellular processes such as DNA and protein metabolism, transporters, cell division, regulation, and cell wall/membrane biogenesis (Table S4).

The essential gene set was further classified using RAST. Unlike the Genbank annotation pipeline which provides a specific annotation for each gene, the RAST pipeline classifies proteins into functional groups called subsystems. RAST was used previously in the comparative genomics of *Liberibacter* species (Fagen et al., 2014a). A functional comparison was done between the essential gene set and the *L*. *crescens* genome (Table S5). In that analysis, a far great proportion of unclassified genes is seen in the essential gene set (43%) than in the whole genome (28.1%). Also in the essential gene set a higher proportion of genes involved in fatty acid, lipid, and isoprenoid metabolism and a much lower proportion of genes with roles in amino acid, RNA, and carbohydrate metabolism as well as cell wall synthesis, motility, chemotaxis, and stress response.

Of the 314 essential protein-coding genes, 238 have homologs in Las (CEGs-Las^+^). These 238 genes are potentially excellent candidates for drug targeting against Las (Table S4). As expected, only 28 of these 238 genes (12%) are hypothetical as most of these would be expected to play a role in core metabolism or essential transport functions.

The remaining 76 protein-coding essential genes of *L. crescens* have no homologs in Las (CEGs-Las^−^) and these genes should help us understand why *L*. *crescens* is cultured while Las has not yet been cultured (Table S6). Of those, 46 genes (61%) are hypothetical which is expected given that we have not yet solved the riddle of culturing Las.

As expected, homologs of both Lso and Las are highly conserved in the *L*. *crescens* essential protein-coding genes set with a small different. Of the 314 essential protein-coding genes, 242 have homologs and 72 have no homologs in Lso (Tables S7, S8).

**Figure 2:**
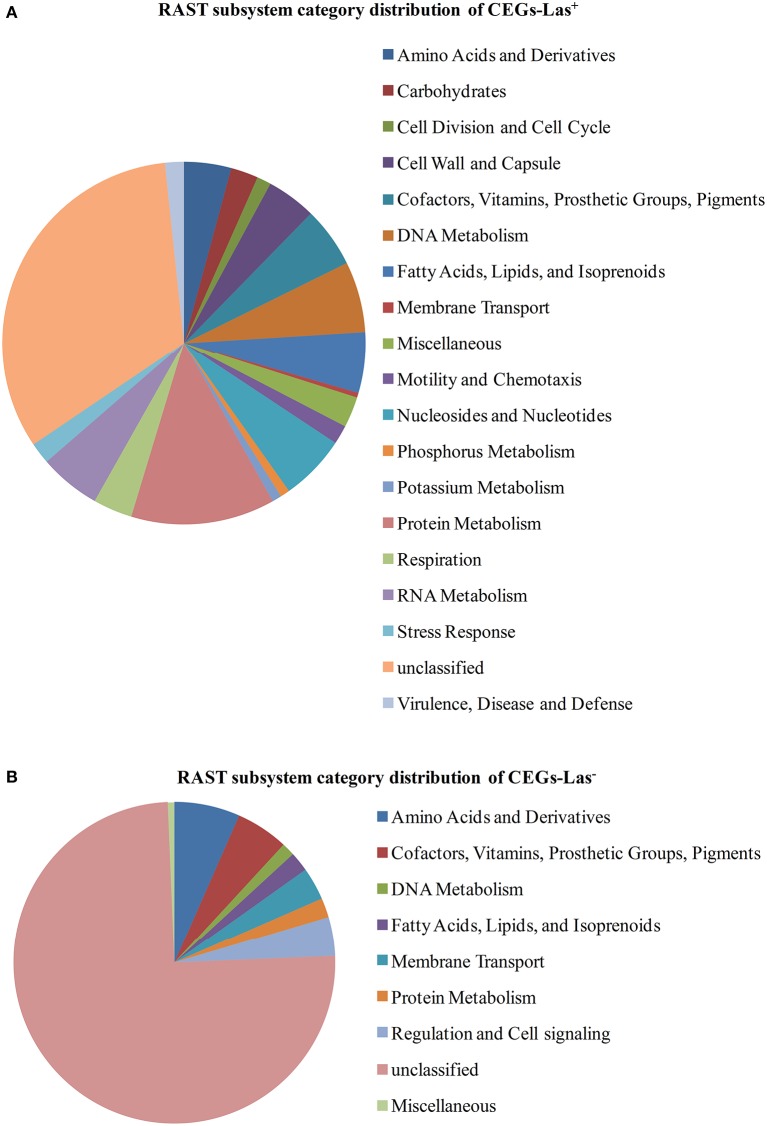
**RAST subsystem category distribution of CEGs**. CEGs were classified into different category using RAST. Unclassified group contains proteins with no known metabolic involvement and putative/hypothetical proteins. **(A)** CEGs-Las^+^. **(B)** CEGs-Las^−^.

The subsystem categories of CEGs-Las^+^ and CEGs-Las^−^ protein sets are divided into functional categories including amino acid synthesis and uptake, cofactor and vitamin synthesis and uptake prosthetic group synthesis, pigment formation, gene regulation (Figure 2). These results suggest that Las may be more reliant on the uptake of available resources from the environment than *L*. *crescens*. The fact that most of the genes are categorized as unclassified suggests that the functions of these genes are still poorly understood.

### Essential gene set and Las gene expression profile comparison

Genes involved in bacterial adaptation to the host environment during infection in plant pathogenic bacteria are known to be up-regulated (Chowdhury et al., 1996). A study by Yan showed the relative expression of 381 Las genes in *planta* versus in psyllid (Yan et al., 2013). Several of these up-regulated Las genes are homologs of *L. crescens* essential genes as shown in Table 2.

**Table 2:** **Essential genes of *L. crescens* with homologs in Las and Lso that are up-regulated in Las in citrus**.

***L. crescens* Essential genes**	**Gene annotation**	**Lso homologs**	**Las homologs**	**Las relative gene expression in *planta* vs. psyllid, (Yan et al., 2013)**
*B488_00550*	3-deoxy-manno-octulosonate cytidylyltransferase	CKC_01160	CLIBASIA_03280	3.29
*B488_00780*	Two component response regulator	CKC_01590	CLIBASIA_01805	1.08
*B488_02170*	Flavodoxin reductases (ferredoxin-NADPH reductases) family 1 protein	CKC_02130	CLIBASIA_01240	2.02
*B488_02560*	Heat shock protein 60 family chaperone GroEL	CKC_04895	CLIBASIA_03720	1.85
*B488_03560*	Retrovirus-related POL polyprotein	CKC_05630	CLIBASIA_02145	1.86
*B488_03900*	Hypothetical protein	CKC_03835	CLIBASIA_04580	2.04
*B488_04630*	CTP synthase	CKC_04370	CLIBASIA_00400	1.51
*B488_06120*	Cystine ABC transporter, permease protein	CKC_03670	CLIBASIA_05075	7.96
*B488_06430*	Phytoene synthase	CKC_04590	CLIBASIA_00220	8.56
*B488_06910*	Signal peptidase I	CKC_03020	CLIBASIA_04190	1.46
*B488_06920*	Holo-(acyl-carrier protein) synthase	CKC_03015	CLIBASIA_04185	1.84
*B488_07560*	Putative uroporphyrinogen-III synthase protein	CKC_03135	CLIBASIA_04685	3.03
*B488_08030*	Thymidylate kinase	CKC_02955	CLIBASIA_00515	3.87
*B488_08410*	Coproporphyrinogen III oxidase, aerobic	CKC_03595	CLIBASIA_04875	2.16
*B488_08500*	Preprotein translocase subunit (SecE)	CKC_05015	CLIBASIA_00140	4.84
*B488_09130*	DNA-binding protein HU-beta	CKC_02845	CLIBASIA_03175	1.6
*B488_09350*	Hypothetical protein	CKC_02735	CLIBASIA_01975	1.32
*B488_09410*	Flagellar basal-body rod modification protein (FlgD)	CKC_02705	CLIBASIA_02035	1.96
*B488_09830*	Kup system potassium uptake protein	CKC_02915	CLIBASIA_03625	1.42
*B488_09880*	RND efflux system, outer membrane lipoprotein CmeC	CKC_04480	CLIBASIA_04145	1.54
*B488_10200*	Inorganic pyrophosphatase	CKC_05220	CLIBASIA_00585	2.2
*B488_10300*	Hypothetical protein	CKC_04695	CLIBASIA_03960	1.28
*B488_10570*	Response regulator	CKC_02580	CLIBASIA_00985	1.61
*B488_10890*	Serine hydroxymethyltransferase	CKC_02395	CLIBASIA_01170	2.32
*B488_10980*	2-Keto-3-deoxy-D-manno-octulosonate-8-phosphate synthase	CKC_02350	CLIBASIA_02780	1.82
*B488_11380*	Hypothetical protein	CKC_00165	CLIBASIA_02385	2.57
*B488_11500*	ABC-type anion transport system, duplicated permease component	CKC_00235	CLIBASIA_02420	7.24
*B488_11710*	RNA polymerase sigma factor (RpoH)	CKC_00305	CLIBASIA_02490	1.37
*B488_11980*	Creatinine amidohydrolase	CKC_00410	CLIBASIA_02600	8.43
*B488_12040*	Chaperone protein (DnaJ)	CKC_02100	CLIBASIA_02625	2.8
*B488_12190*	Transketolase (TktA)	CKC_02045	CLIBASIA_02710	6.24
*B488_12520*	Two-component system response regulator (QseB)	CKC_04710	CLIBASIA_03950	1.74
*B488_12610*	Phosphate transport ATP-binding protein (PstB)	CKC_00595	CLIBASIA_02955	7.28
*B488_13000*	Lipopolysaccharide ABC transporter, ATP-binding protein (LptB)	CKC_00785	CLIBASIA_03155	7.46
*B488_13040*	Integration host factor beta subunit	CKC_00805	CLIBASIA_03175	1.6

*Gene expression data from (Yan et al., 2013)*.

## Discussion

### Functional comparison between the essential gene set and the entire *L. crescens* genome

The much higher proportion of unclassified genes in the essential gene set compared to the whole genome was a surprise. The vast majority of the essential genes were expected to be core metabolism genes that are already well understood but a large number are of unknown function with many of those also with homologs in the two uncultured *Ca*. Liberibacter plant pathogens. Clearly, much of the metabolism of fastidious bacteria that interact with plants and insects is largely unknown. Not surprisingly, the rich medium used for the culture of *L. crescens* in this work, precludes the requirement for many of the genes involved in amino acid, carbohydrate, and RNA metabolism.

### Implications of the *L. crescens* essential gene set for Las culturing

Despite vigorous efforts to culture Las, there is still no reproducible, robust culturing method available. This greatly hinders research on understanding the pathogenesis of this organism as well as limit progress in treating infected citrus trees. *L. crescens* is the sole culturable member of the *Liberibacter* genus (Fagen et al., 2014a). The stream lined genome of Las possesses 356 fewer genes than does that of *L. crescens* genome (Fagen et al., 2014b). The majority of the *L. crescens* essential genes that are unique to *L. crescens* encode hypothetical proteins.

Among the essential genes in *L. crescens* that are absent in Las are genes that code for proteins involved in stress responses. Our hypothesis here is the plant and insect habitats of Las do not expose this pathogen to some of the stresses that *L. crescens* must survive in culture. For example, *B488_09700* encodes protein-L-isoaspartate O-methyltransferase (PIMT). One of the several processes that can damage proteins and affect the cellular functioning is the formation of L-isoaspartyl residues on proteins. PIMT acts as a protein repair enzyme, converting the abnormal L-isoaspartyl residues back to the normal L-aspartyl residues (Clarke, 2003). Oxidative damage is another process that inactive proteins. Reactive oxygen species (ROS) and reactive nitrogen intermediates (RNI) are generated as natural byproducts during cell metabolism and stress responses. These byproducts can damage proteins by oxidizing the methionine residues, forming methionine sulfoxide. *B488_10230* encodes peptide methionine sulfoxide reductase (MsrA), which catalyzes the reduction of methionine sulfoxide residues in proteins to methionine during oxidative damage (Weissbach et al., 2002). Low dosage of anti-oxidants such as ascorbic acid and glutathione can be added to the culture medium to protect the cells (La Scola et al., 2014). Both processes prevent the accumulation of potentially dysfunctional proteins and avoid the unnecessary protein degradation and resynthesis, indicating *L. crescens* has a better mechanism on protein repairing than in *L. asiaticus*.

The functions of another set of genes missing in Las but required for the growth of *L. crescens* in BM7 medium can be replaced by simple additions to the medium such as high levels of folate and aromatic amino acids. Folate biosynthesis is an essential pathway to synthesize folate (an essential cofactor for cell metabolism) in most of the bacteria due to the fact that folate-dependent formylation of the initiator tRNA is a hallmark of bacterial translation (de Crécy-Lagard et al., 2007). However, genes code for 2-amino-4-hydroxy-6-hydroxymethyldihydropteridine pyrophosphokinase (*B488_08090*), a putative dihydroneopterin aldolase (*B488_08100*), and dihydropteroate synthase (*B488_08110*) are missing in Las but present in *L. crescens* and Lso. For the synthesis of aromatic amino acids, the *L. crescens* genome codes for indole-3-glycerol phosphate synthase (*B488_03020*), 2-keto-3-deoxy-D-arabino-heptulosonate-7-phosphate synthase II (*B488_07360*), cyclohexadienyl dehydrogenase (*B488_11240*), and 3-dehydroquinate synthase (*B488_11320*), which are involved in phenylalanine, tyrosine, and tryptophan biosynthesis/shikimate pathway. All of these are essential proteins for the growth of *L. crescens* on BM7 medium but absent in Las.

One of the *L. crescens* essential proteins is nicotinamidase (PncA, B488_07080). The function of PncA is to hydrolyze nicotinamide to nicotinic acid for the production of NAD+, maintaining NAD+ homeostasis (Gazzaniga et al., 2009). Although *L. crescens* has a niacin/nicotinic acid transporter NiaP (B488_08370), it was determined to be non-essential in this study. The result is in agreement to the fact that the composition of BM7 medium does not include nicotinic acid. NAD is an important cofactor that contributes to important biological processes in both prokaryote and eukaryote (Vrablik et al., 2009; Evans et al., 2010). It acts as electron carrier during redox reaction and even involves in posttranslational modifications. Las does not appear to have PncA nor NiaP; indicating nicotinic acid is not required for the growth of the bacterium.

However, the key to culturing Las may still lie within the majority of the genes encoding hypothetical proteins that are essential in *L. crescens* but also found in Las. Improved bacterial genome annotation is required to unravel unknown metabolic pathways that may be required for Las in culture.

### Implications of the *L. crescens* essential gene set for Las inhibition

Inhibition of an essential function for growth is a common means to develop antibiotics against a pathogen of interest (Clatworthy et al., 2007). Since *L. crescens* BT-1 and Las are close relatives, it is not surprising that 238 essential genes of *L. crescens* have homologs in Las. The availability of a list of essential genes provides a significant advantage because it allows us to choose specific targets, such as enzymes and regulators, to introduce a lethal effect on Las. Antibiotics can be designed to target more than one essential gene and thus minimize the development of resistance. Genes involved in cell wall or membrane biosynthesis are important targets for inhibition of the growth of Las. The incomplete synthesis of cell walls or membranes may prevent the bacteria from resisting high osmotic pressure and high sucrose concentrations present within phloem. Several *L. crescens* essential proteins such as UDP-N-acetylglucosamine O-acyltransferase (B488_00500), tetraacyldisaccharide 4′-kinase (B488_02350), and 2-keto-3-deoxy-D-manno-octulosonate-8-phosphate synthase (B488_10980) are involved in lipopolysaccharide biosynthesis. Homolog of *B488_10980* in Las (*CLIBASIA_02780*) was also up-regulated during infection in *planta*, indicating *CLIBASIA_02780* is a promising target for growth inhibition. Effort has been shown to discover inhibitors for the biosynthetic pathway (Shapiro et al., 2013).

Other essential genes with homologs in Las [*B488_01060* and *B488_02950*: enoyl-(acyl-carrier-protein) reductase; *B488_01080*: 3-hydroxydecanoyl-(acyl-carrier-protein) dehydratase; *B488_03630*: phosphate:acyl-ACP acyltransferase PlsX; *B488_10110*: acyl carrier protein AcpP; *B488_10130*: malonyl CoA-acyl carrier protein transacylase; *B488_11350*: acetyl-coenzyme A carboxyl transferase alpha chain] are involved in fatty acid synthesis. In *E. coli*, genes in the fatty acid biosynthetic pathway are essential (Egan and Russell, 1973; Zhang and Cronan, 1998). This pathway is a common target for novel antibacterials triclosan, isoniazid, thiolactomycin, and cerulenin (Heath and Rock, 2004).

### Differences on essential gene set comparison between Las and Lso

Only five of the *L. crescens* essential gene homologs that are present in Las are absent in Lso while nine of the *L. crescens* essential genes found in Lso are absent in Las. All 14 of these genes are highly conserved. The essential genes uniquely missing in Las are involved in folate-dependent formylation of tRNA. The few differences observed in the comparison of the *L. crescens* essential gene set with both Las and Lso suggests that the inability to culture both pathogens may be very similar. Thus, we predict that a medium that can successfully culture Las will culture Lso.

### Increased in *planta* expression of *L. crescens* essential gene homologs in Las using gene expression data from Yan et al. (2013)

In this study, 314 of *L. crescens* essential genes were suggested. A study of the relative expression of 381 Las genes in *planta* versus in psyllid is publicly available. We hypothesized that Las genes that are up-regulated in *planta* and are common in the *L. crescens* essential set are the most promising candidates for drug targeting. Within the 314 *L. crescens* essential genes, 35 Las homologs were up-regulated during the infection in *planta* (Table 2). Genes related to ABC transporter (*CLIBASIA_05075, CLIBASIA_02420, CLIBASIA_02955, CLIBASIA_03155*) were especially up-regulated, up to 7.96-fold. Transporters are known virulence factors in pathogenic bacteria, aiding in uptake of nutrients and metal ions (Li et al., 2012; Yan et al., 2013). Gene encoding lipopolysaccharide ABC transporter (*CLIBASIA_03155*), together with other lipopolysaccharide biosynthesis genes (*CLIBASIA_03280, CLIBASIA_02780*) were also up-regulated. Lipopolysaccharide is an essential component for cell wall synthesis, protecting the cells from the harsh environment. Antimicrobial compounds can be designed to target these genes, controlling HLB disease (Akula et al., 2012). The potential role in pathogenesis of the other genes in Table 2 is unknown. In addition, the up regulation of some of these genes in the citrus may be less important than their down regulation for survival of Las in the psyllid.

## Summary

**Figure 3:**
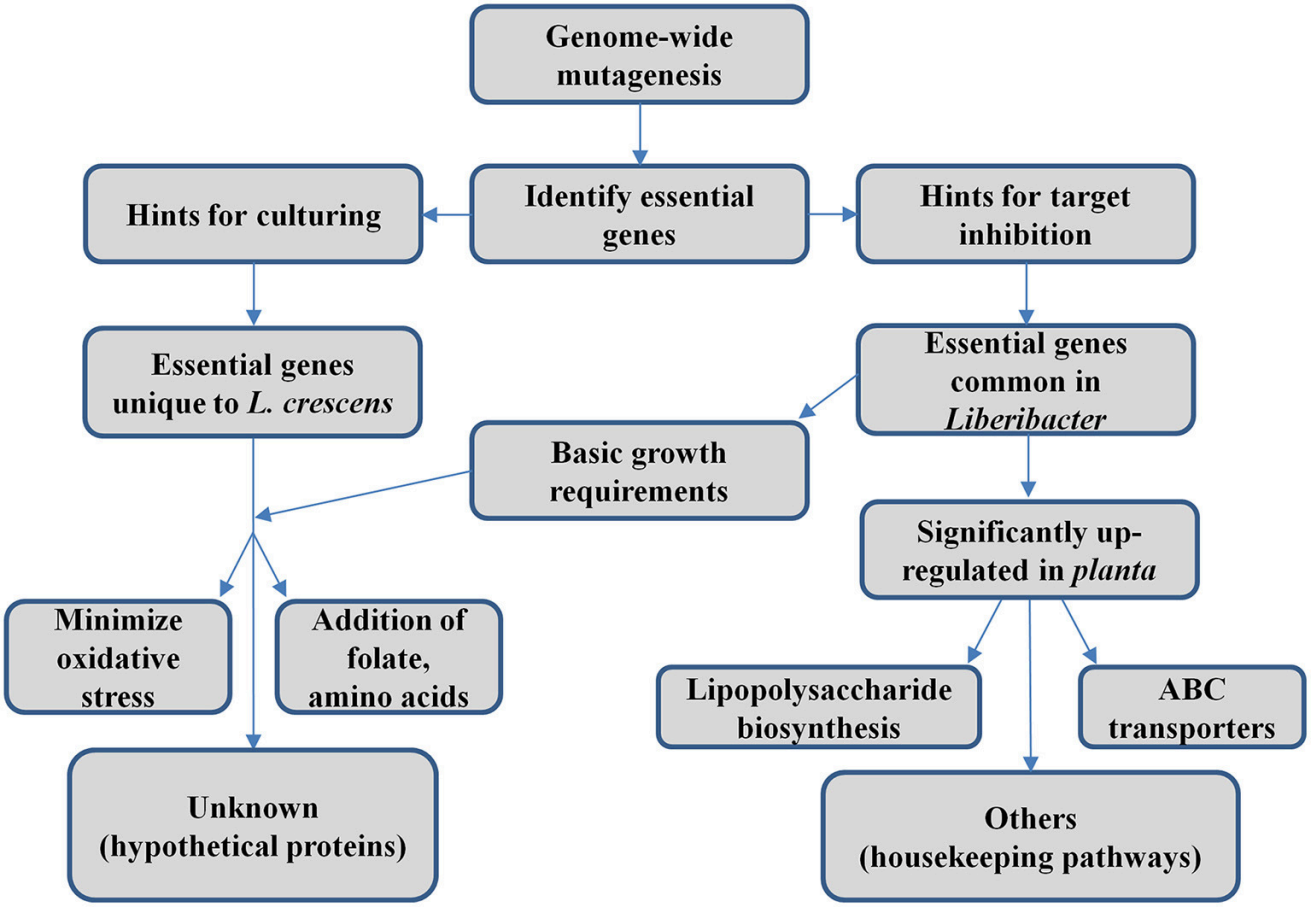
**Conceptual diagram of work summary**.

The essential gene set of *L. crescens* described here provides suggestions for both culturing (essential genes that are unique to *L. crescens*) and inhibition of Las (essential genes that are common in *Liberibacter* pathogens). A general workflow of this study is summarized in Figure 3. However, there are limitations to this approach. First, it only describes those genes that are essential for growth on BM7 medium. Second, some of the putative essential genes may simply have escaped mutagenesis by chance, which would suggest that our mutagenesis did not saturate the genome. Third, some of the insertions may not have interrupted gene function. Nevertheless, the number of genes in our 314 essential gene set is larger than those observed in *E. coli*, with 295 essential genes (Juhas et al., 2014), and *Bacillus subtilis*, with 261 essential genes (Commichau et al., 2013).

Given that the study of the genetics and biochemistry of *L. crescens* is in its infancy, it is not surprising that the current list of essential genes in *L. crescens* is larger compared to those of the model systems of *E. coli* and *B. subtilis*. The essential gene sets of both model systems have reduced in size as our knowledge grows (Juhas et al., 2014). However, the growth conditions and physiology of the three bacteria are very different so that each bacterium uses its own specific set of genes to survive in a particular growth medium. Indeed, some of the essentials genes in *E. coli* are not present in the *L. crescens* genome at all. Of the 295 protein-coding genes in the *E. coli* essential gene set, 39 have no homologs in *L. crescens*.

The gene essentiality is specific to the experimental growth condition and likely differs from those genes required in the natural habitat of *L. crescens*. The results described here provide an approximation of the essential elements for *L. crescens* BT-1 growth *in vitro*.

In summary, beside the nutrients in BM7 that support the growth of *L. crescens*, the study herein suggests folic acid and amino acids such as phenylalanine, tyrosine, and tryptophan should be supplemented when formulating a growth medium for Las. Our results are consistent with the finding as in (Fagen et al., 2014b). The majority of hypothetical proteins in the essential gene set still hinder the culturing of Las. In order to cure HLB, ABC transporter, and lipopolysaccharide biosynthesis genes should be used as the primary targets for developing antimicrobials. Triclosan, isoniazid, thiolactomycin, and cerulenin are novel antimicrobials that can inhibit the fatty acid biosynthetic pathway. More insights are needed from worldwide citrus researchers to fully solve the riddle of HLB.

## Author contributions

All authors listed, have made substantial, direct, and intellectual contribution to the work, and approved it for publication.

### Conflict of interest statement

The authors declare that the research was conducted in the absence of any commercial or financial relationships that could be construed as a potential conflict of interest.
