# Supplementary material for: Identification of the Genes Required for the Culture of Liberibacter crescens, the Closest Cultured Relative of the Liberibacter Plant Pathogens
Source: Front Microbiol. 2016 Apr 20;7:547. doi: 10.3389/fmicb.2016.00547 (PMC4837290; doi:10.3389/fmicb.2016.00547)
Supplement: Supplementary file 1 [file Table1.docx]

**Supplemental Table S1: EZ-Tn5 insertion sites of *L. crescens* BT-1**
>B488_00200|29357..29456|Liberibacter_crescens_BT-1_EZ-Tn5_Transposon_Disruption

GTCATGGAGTGTTTTGTCAGGAATAGGTGATAAAGAACATTCTAATAAAGCCATGGATGCAGTACTAAAAAAATTAGTAGATGAAGAAAATAAGATCATA

>B488_05820|638491..638590|Liberibacter_crescens_BT-1_EZ-Tn5_Transposon_Disruption

GGGATCTCCTGTCCAGGATGCTGTTATGGGCATTGTAAGATTTCCCTGAAAAGAATCTCCACGTTGCAGACTTTGATGAAATTGCTGAAATTTTTTCTGA

>B488_02010|238021..237922|Liberibacter_crescens_BT-1_EZ-Tn5_Transposon_Disruption

GCTTAATATATGCAAAACCAGGATCATCAGGTGCAGCTATAATTGCTTCTCCTGAAGCTTCTGTCTGAACAACAAGATTGTCACCTATATTCTTGAGTCC

>B488_06270|691129..691030|Liberibacter_crescens_BT-1_EZ-Tn5_Transposon_Disruption

GGCTGGACTCATGGCAAAAGGCTTTCATTGGATTTAAACCTTACGCACTGATGCAATCCCTACCAGTCACTTTTGGCGTACAAACAAAAATTCATACCAC

>B488_06680|742037..742136|Liberibacter_crescens_BT-1_EZ-Tn5_Transposon_Disruption

GACCGATACCATCGCCAGGAAGCACAAGTACTTTTTTCATGATGATGCTTCTTTCAGAAAATCTTTTATCAGGGAGAGGGGACAGGCATCACGGAGCGGA

>B488_09500|1022490..1022391|Liberibacter_crescens_BT-1_EZ-Tn5_Transposon_Disruption

GTTATGGATACAGCTATACAAGGAAATGTCAATATACGTAGTTCAATGATAAAAGACATGGATATACAGCTACGTAAGACAAATATAGATGCTTTTTCAG

>Intergenic_Region|1163443..1163385|Liberibacter_crescens_BT-1_EZ-Tn5_Transposon_Disruption

TGTTTAGGGAGGAAATAATTGGATGGCAATTTGTGAAAAATGTTGAATTGCTTTTTTCTGTGCGATGTTGGTAGGGTCAAGGCGCCCTGGACTCATACCA

>Intergenic_Region|1163443..1163344|Liberibacter_crescens_BT-1_EZ-Tn5_Transposon_Disruption

TGTTTAGGGAGGAAATAATTGGATGGCAATTTGTGTAGAAAGTTTAATTTTATTTTCCTTTGTGTATGCTTGACGTTCGTGGTCCTCTGCGAAAAATAAA

>Intergenic_Region|327150..327051|Liberibacter_crescens_BT-1_EZ-Tn5_Transposon_Disruption

GGCGAGGGTTGCGCTCGTTGCGGGACTTAACCCAACATCTCACGACACGAGCTGACGACAGCCATGCAGCACCTGTGTGCGTGCCTCCGAAGAAGAAAAT

>Intergenic_Region|327150..327051|Liberibacter_crescens_BT-1_EZ-Tn5_Transposon_Disruption

GGCGAGGGTTGCGCTCGTTGCGGGACTTAACCCAACATCTCACGACACGAGCTGACGACAGCCATGCAGCACCTGTGTGCGTGCCTCCGAAGAAGAAAAT

>B488_06040|667466..667565|Liberibacter_crescens_BT-1_EZ-Tn5_Transposon_Disruption

GTGTTTATGCAATAAAGAAAGTGGTTTCTATTCCTGTTACAATTAAATGTCGTATAGGTGTAGATGATCAAGTTCCTGAGCTTGCGTTGCGTAATTTAAT

>Intergenic_Region|329143..329044|Liberibacter_crescens_BT-1_EZ-Tn5_Transposon_Disruption

CTAGTACTCCTCGGTATTCGGAGTTTGGTTAGGAGCAGTAAGGCGGTGAGCCCCCATAGCCTATCCAGTGCTCTACCCCCGAGGGTATTCAGTTAACGCT

>B488_08290|906041..906140|Liberibacter_crescens_BT-1_EZ-Tn5_Transposon_Disruption

CATTACAACTGCGTGAAATCGGTGTTCTTCTTACGGCTATCATGGTTGCAGGACGTTCTGGAAGTTCTATTACAGCGGAAATTGGTTCCATGAAAATGCG

>B488_11660|1271714..1271615|Liberibacter_crescens_BT-1_EZ-Tn5_Transposon_Disruption

GGTTCGAATTCATACAGGGTTACAACAGGACCTGGACTTACATTAATAATTTCTCCTTTGACTCCAAAATCCTCAAGGATACTTTCTAGTACTCCAGCCT

>B488_08210|894228..894327|Liberibacter_crescens_BT-1_EZ-Tn5_Transposon_Disruption

CATCAAATTTTAGAAGAAGAAGAAGGTAAAAAAGACATCAATAGAAGACTGGCACTCTATTTTGAACGTAAAGCCCCAACTATCACAAATATTTATGATT

>B488_10550|1149823..1149922|Liberibacter_crescens_BT-1_EZ-Tn5_Transposon_Disruption

ACCATTAACTATTTCAGCTATACCTTTTATAGCGCGATTAATTGAAACTTCCATTCGTGAAGTTAATATTGAGCTTGTTGAAACCGCACGTTCAATGGGA

>B488_06680|741005..741104|Liberibacter_crescens_BT-1_EZ-Tn5_Transposon_Disruption

GAATAGCTTGTGCCATTCCTTCTGTAGTGGCATTTCCACCCAAGTCATAAGTAAGATGATGACCGATACGAATCGTTTGGCGGACTGCAGTATCAATGCG

>B488_12440|1359656..1359575|Liberibacter_crescens_BT-1_EZ-Tn5_Transposon_Disruption

GTAATACAAGGGGTGTTATGCTTCCATTGATGGTTTGGGATCTATTACCATCATTGATGATGGAGGAATTCCTCCTTCTAACCAACCTGAGAGTATAGCT

>B488_12440|1359673..1359574|Liberibacter_crescens_BT-1_EZ-Tn5_Transposon_Disruption

TCTCTGATCTTAGCGGTTGCTTCTATTGATGGTTTGGGATCTATTACCATCATTGATGATGGAGGAATTCCTCCTTCTAACCAACCTGAGAGTATAGCTG

>Intergenic_Region|330816..330915|Liberibacter_crescens_BT-1_EZ-Tn5_Transposon_Disruption

GAATGGATAACCGCTGAAAGCATCTAATCGGGAAACCAACCTGAAAACGAGTATTCCCTATCAGAGCCATGGAAGACTACCACGTTGATAGGCTGGGTGT

>B488_06170|680158..680257|Liberibacter_crescens_BT-1_EZ-Tn5_Transposon_Disruption

CTATCATCCTTTGAAGATGTTAATTCTTCATGCAATATCTGAAGCTCAAGCCATTGTTCTTGTTTGTCTTCCAGGTCTTTGTATAATAATTCAAGTTCAG

>B488_01870|227229..227130|Liberibacter_crescens_BT-1_EZ-Tn5_Transposon_Disruption

AGATAAAATACTCCGAACATATCCCCCATGAATAAGAACTTTTTGAGCATATTCTTCAGTTAAGCGTGCTAATTGATTCAATTTCCCTTTCGGCACTACA

>B488_12300|1345722..1345623|Liberibacter_crescens_BT-1_EZ-Tn5_Transposon_Disruption

ATTGAAAACCAAAGAATTCTGCCTCCTCCAAATATCATTCGATTGGATGATGATGATCCTTATTTTGTGGTAGCAGCAGATAAAGGAACTGCAACATTTT

>Intergenic_Region|216013..216112|Liberibacter_crescens_BT-1_EZ-Tn5_Transposon_Disruption

AGTTAAATCATTGCGGGACCTTCGCTTATCGGTTGATTATGATACTGAATCTGCAGTAAAGTCTGTCATTTAAAATTATATTTCACTTGGCGTGATACTT

>Intergenic_Region|533711..533810|Liberibacter_crescens_BT-1_EZ-Tn5_Transposon_Disruption

GTTTTAAAGGCCTGAACAACCGTTTTGACAGGCTTGAAGGTAAAATTGATTCCGAGTTTAAAGAAAATAGAAATAAACTTGAGACCATTACCGAAAAGAT

>Intergenic_Region|533711..533810|Liberibacter_crescens_BT-1_EZ-Tn5_Transposon_Disruption

GTTTTAAAGGCCTGAACAACCGTTTTGACAGGCTTGAAGGTAAAATTGATTCCGAGTTTAAAGAAAATAGAAATAAACTTGAGACCATTACCGAAAAGAT

>Intergenic_Region|216013..216112|Liberibacter_crescens_BT-1_EZ-Tn5_Transposon_Disruption

AGTTAAATCATTGCGGGACCTTCGCTTATCGGTTGATTATGATACTGAATCTGCAGTAAAGTCTGTCATTTAAAATTATATTTCACTTGCCGAGATACTT

>Intergenic_Region|527686..527736|Liberibacter_crescens_BT-1_EZ-Tn5_Transposon_Disruption

CACCTGAACAGAGCAAAAATTAACAGCTAATAACCCGTTCAGGTGATCCTTCAACTCAGCAAAAGTTCCGGGCCACGATCGTATTTAACGCCTTGGTGTG

>Intergenic_Region|330816..330915|Liberibacter_crescens_BT-1_EZ-Tn5_Transposon_Disruption

GAATGGATAAGCGCTGAAAGCATCTAATCGGGAAACCAACCTGAGAACCAGTATTCCCTATCAAATCCATGGAAGACTACCACGTTGATAGGCTGGGTGT

>B488_05130|554360..554459|Liberibacter_crescens_BT-1_EZ-Tn5_Transposon_Disruption

CTTGTGCCCTGTTGATGACATTACTGACAGAAGGCGGCAATGAAATCAGTCTCCGTGCGGAGTGGCCTTCTCTTATAAGACAGGTGCCAGTGACCAGTGA

>B488_04860|537155..537056|Liberibacter_crescens_BT-1_EZ-Tn5_Transposon_Disruption

CTCATAGCTCCTGAACTTTCTTATATGGACGAGGAGCTTTAATATGGATCCTGAACCATATGCTGTACAACCCTGTTCAGAATAATTATTCGGGAGTAGA

>B488_13570|1477768..1477669|Liberibacter_crescens_BT-1_EZ-Tn5_Transposon_Disruption

GACCAGGTCTGTTTCCATTCAAATTTTTTTGATTCAACTAAAGGAAGTTCAGAACGAATAATTTGTTCACGTGAATATGAAAATAGATAACTTGAATCAA

>B488_07010|778239..778140|Liberibacter_crescens_BT-1_EZ-Tn5_Transposon_Disruption

GTCGAGACATCAAAAGCGTTCTCCGATTGAAGGAACCTTATGGAGACGTTGGTTATTTTATGATCTTCTTGGAGCACCAAATACAGCGGTTCGTATTATT

>Intergenic_Region|328974..329073|Liberibacter_crescens_BT-1_EZ-Tn5_Transposon_Disruption

CTTGTGGCTAGGGGTGAAAGGCCAATCAAACTCGGAGATAGCTGGTTCTCCGCGAAATCTATTTAGGTAGAGCGTTAACTGAATACCCTCGGGGGTAGAG

>Intergenic_Region|328974..329073|Liberibacter_crescens_BT-1_EZ-Tn5_Transposon_Disruption

CTTGTGGCTAGGGGTGAAAGGCCAATCAAACTCGGAGATAGCTGGTTCTCCGCGAAATCTATTTAGGTAGAGCGTTAACTGAATACCCTCGGGGGTAGAG

>B488_08230|896728..896825|Liberibacter_crescens_BT-1_EZ-Tn5_Transposon_Disruption

CCCTGGAATTCCTGAACATGCTCTCCAGGATGTTTTTAAACCATTCTTTTCGACTTGACTCAGCACGTAACCTTAATAAATCCGGCACAGGCCTTCGGAC

>Intergenic_Region|328987..329086|Liberibacter_crescens_BT-1_EZ-Tn5_Transposon_Disruption

GTGAAAGGCCAATCAAACTCGGAGATAGCTGGTTCTCCGCGAAATCTATTTAGGTAGAGCGTTAACTGAATACCCTCGGGGGTAGAGCACTGGATAGGCT

>Intergenic_Region|328987..329086|Liberibacter_crescens_BT-1_EZ-Tn5_Transposon_Disruption

GTGAAAGGCCAATCAAACTCGGAGATAGCTGGTTCTCCGCGAAATCTATTTAGGTAGAGCGTTAACTGAATACCCTCGGGGGTAGAGCACTGGATAGGCT

>B488_09270|1000290..1000388|Liberibacter_crescens_BT-1_EZ-Tn5_Transposon_Disruption

CGCTCACACCCATCTTAAAGACAATTCCATTGCTCTCTTAATCTCTTTATCTTCTGCTGGCAAAGATATACACTCATCAAGCTGCATTTGTATATCCGAA

>B488_09500|1022490..1022391|Liberibacter_crescens_BT-1_EZ-Tn5_Transposon_Disruption

GTTATGGATACAGCTATACAAGGAAATGTCAATATACGTAGTTCAATGATAAAAGACATGGATATACAGCTACGTAAGACAAATATAGATGCTTTTTCAG

>B488_08220|895036..895135|Liberibacter_crescens_BT-1_EZ-Tn5_Transposon_Disruption

ATATAATGATGCCTCATGAATCGGGCCTTTCACTCACAAAAAGTTTGCGTGTTGATAATCCTGTTCCGGTTATTTTACTCACCGCTCTTGATGAAACACA

>B488_04510|505042..504943|Liberibacter_crescens_BT-1_EZ-Tn5_Transposon_Disruption

AGAAATACCTTCTCCATACATGATATCAAATTCAACTTGCTTGAAAGGAGGAGCCATTTTATTCTTAACTACTTTAACTCTTGTCTGATTACCAATAATT

>B488_00200|27829..27730|Liberibacter_crescens_BT-1_EZ-Tn5_Transposon_Disruption

TCATTACTCCAAGGCGTAATTTTATAATCCCGAGAGTTTTGACTCCAAGTAAAACCTGCTCCTTCTGCTGACACATGGAATCCAAAGTTTTCCCCGGAAA

>B488_05130|554303..554268|Liberibacter_crescens_BT-1_EZ-Tn5_Transposon_Disruption

ATGTTCCCCTGTCCTCGTTATTGGTGTATTATTGGTCTCTAATATGACTGTTGCTCGAACTCAAACTTCATTGACAATGGAAACAATCGTCATGGTGTTG

>B488_05130|554360..554459|Liberibacter_crescens_BT-1_EZ-Tn5_Transposon_Disruption

CTTGTGCCCTGTTGATGACATTACTGACAGAAGGCGGCAATGAAATCAGTCTCCGTGCGGAGTGGCCTTCTCTTATAAGACAGGTGCCAGTGACCAGTGA

>B488_12300|1345722..1345662|Liberibacter_crescens_BT-1_EZ-Tn5_Transposon_Disruption

ATTGAAAACCAAAGAATTCTGCCTCCTCCAAATATCATTCGATTGGATGATGATGATCCTTCAACTCAGCAAAAGTTCAGTAAAGAATGTGGACTATTTT

>B488_06040|667466..667565|Liberibacter_crescens_BT-1_EZ-Tn5_Transposon_Disruption

GTGTTTATGCAATAAAGAAAGTGGTTTCTATTCCTGTTACAATTAAATGTCGTATAGGTGTAGATGATCAAGTTCCTGAGCTTGCGTTGCGTAATTTAAT

>B488_06540|724039..724138|Liberibacter_crescens_BT-1_EZ-Tn5_Transposon_Disruption

ACCTGGACTATTGCAAAGCTTTGTAGATGTCTTAACGCAGGAATTTAATACTGCTCCTTCAACATTAAAGAACAAAGCAAGAATTGTCTTCCTGATCAAT

>B488_10820|1179821..1179920|Liberibacter_crescens_BT-1_EZ-Tn5_Transposon_Disruption

GTTCAATACTGTAAAGACCTCAATCTCAGAAACACCTATAATTGGTTTAACAATAACAATCTTTGCAAGCGTAATTAATTTTTTTTGGGGATATTTTCTT

>B488_10680|1165100..1165001|Liberibacter_crescens_BT-1_EZ-Tn5_Transposon_Disruption

GATTTCAGTCGATCATAAAAATCAAATACAACTTCATTAAGAGGTAATTCGTATGATATGATAATTCGGTTGCCAATGTAGCTTATTTCAATTTGGATAC

>Intergenic_Region|329200..329229|Liberibacter_crescens_BT-1_EZ-Tn5_Transposon_Disruption

CTCCAGCTAAGGTCCCCAAGTCATGGCTAATACAAGGGGTGTTATGAGCCATATATCTCTTCTGGAGGGGCACTGCCCTAAGATGAATACAGCGTCCAGG

>Intergenic_Region|989227..989128|Liberibacter_crescens_BT-1_EZ-Tn5_Transposon_Disruption

CTGTTGCTTTGATGTAATAGGTATTATCATGAATATTTCATGAGAATCGGAGACAAGTTGTTGAGAAATGAAATTTTTAATTCTACAACTTGTCCTGTTG

>B488_12020|1312627..1312726|Liberibacter_crescens_BT-1_EZ-Tn5_Transposon_Disruption

GAGCAGAACATTATGTTGTTGATATGGTTATTGATCAAATACAAAGTTTAGTACCCCAAAATAAGGAAGACCTCATCGTAGAAACTTCACTTAATATGAA

>B488_03000|348094..347995|Liberibacter_crescens_BT-1_EZ-Tn5_Transposon_Disruption

ACTCAGGACTACGATAATTACCTCGTACTGTTTCAAACCACTTCTTAACAATTTCATCTTTTGGCGGAGATATCGGATCAATATTGTTCTTAGTCAGCAC

>Intergenic_Region|330816..330915|Liberibacter_crescens_BT-1_EZ-Tn5_Transposon_Disruption

GAATGGATAACCGCTGAAAGCATCTTATCGGGAAACCAACCTGAAAACGAGTATTCCCTATCATAGCCGTGGAAGACTACCACGTTGATATGCTGGGTGT

>B488_00260|38512..38612|Liberibacter_crescens_BT-1_EZ-Tn5_Transposon_Disruption

ATATTCACAGCTGATGCTCCACAATTAGTGAATAATCTTATAACAGAGCGTGCAGATATGGTCGTGGGGGTGCGTCGTTATAAAAAAAACTATATTGATC

>B488_00530|67633..67534|Liberibacter_crescens_BT-1_EZ-Tn5_Transposon_Disruption

GTTCTTTATCGCTACCCGTCATGAAAACTTGTGCACCAATATCGTTCACTATATCGAAAAGCACAGCTCTTCTTTTTTTATCCAGATGCGCAGCAATTTC

>Intergenic_Region|330816..330915|Liberibacter_crescens_BT-1_EZ-Tn5_Transposon_Disruption

GAATGGATAACCGCTGAAAGCATCTAAGCGGGAAACCAACCTGAAAACGAGTATTCCCTATCAGAGCCGTGGAAGACTACCACGTTGATAGGCTGGGTGT

>B488_09250|998098..998197|Liberibacter_crescens_BT-1_EZ-Tn5_Transposon_Disruption

GCTCAATTGATGGCTTATTATCCTCTCTTCTCCTTTGTTTCCATATTATTCTTTATTTCTTTTGGATGGGTAACTGTTAATGCATTATACCACCAAAAAG

>B488_08300|906575..906674|Liberibacter_crescens_BT-1_EZ-Tn5_Transposon_Disruption

ATGTTCCCCTGAACAAAAAAGAATGTGTATTATCTGTCTCTAATATGACTGTTGTTCTGAATAAAAAAACAATATTTAAAAATCTTGATCTTCAGGTATA

>B488_12300|1345722..1345662|Liberibacter_crescens_BT-1_EZ-Tn5_Transposon_Disruption

ATTGAAAACCAAAGAATTCTGCCTCCTCCAAATATCATTCGATTGGATGATGATGATCCTTCAACTCAGCAAAAGTTCTGCCAAGTTTGCCCGTTGTTTT

>B488_10410|1137052..1136953|Liberibacter_crescens_BT-1_EZ-Tn5_Transposon_Disruption

CTTTAGAAGAGAAGGCTTTTGATTTTTATACTTCGTCTTCTGTGGAGGATTCTTGGTTTGGTTATTATCGTAAAAAAATGTTGCACTTTAAAGTTTTTAA

>Intergenic_Region|527686..527736|Liberibacter_crescens_BT-1_EZ-Tn5_Transposon_Disruption

CACCTGAACAGAGCAAAAATTAACAGCTAATAACCCGTTCAGGTGATCCTTCAACTCAGCAAAAGTTCCATGGTTCAGGTGTGATATCAGATCTCAACTT

>Intergenic_Region|97894..97795|Liberibacter_crescens_BT-1_EZ-Tn5_Transposon_Disruption

CCCAATAACCAGCATTCAGCAAACAGAAAAATCCTTTATATAATTAAAGATCTTAAAAAGAAAATTCACGATACCAATATATCAAATCAAAGCAAAAAAA

>B488_12300|1345722..1345662|Liberibacter_crescens_BT-1_EZ-Tn5_Transposon_Disruption

ATTGAAAACCAAAGAATTCTGCCTCCTCCAAATATCATTCGATTGGATGATGATGATCCTTCAACTCAGCAAAAGTTCCAAAGATGGTGGGCTGGTTGAG

>Intergenic_Region|329143..329044|Liberibacter_crescens_BT-1_EZ-Tn5_Transposon_Disruption

CTAGTACTCCTCGGTATTCGGAGTTTGGTTAGGAGCAGTAAGGCGGTGAGCCCCCATAGCCTATCCAGTGCTCTACCCCCGAGGGTATTCAGTTAACGCT

>B488_09250|998098..998196|Liberibacter_crescens_BT-1_EZ-Tn5_Transposon_Disruption

GCTCAATTGATGGCTAATTATCCTCTCTTCTCCTTTGTTTCCATATTATTCTTTATTTCTTTTGGATGGGTAACTAGTTAATGCATTATACCACCAAAAA

>B488_12300|1345722..1345662|Liberibacter_crescens_BT-1_EZ-Tn5_Transposon_Disruption

ATTGAAAACCAAAGAATTCTGCCTCCTCCAAATATCATTCGATTGGATGATGATGATCCTTCAACTCAGCAAAAGTTCCACGCTTAATAGGCTGGGTGTG

>Intergenic_Region|329208..329109|Liberibacter_crescens_BT-1_EZ-Tn5_Transposon_Disruption

TAGCTGGAGATCAGGGTTGTTTCCCTTTCCACGACGGACGTTAGCACCCGCCGTGTGTCTGCCAACTAGTACTCCTCGGTATTCGGAGTTTGGTTAGGAG

>B488_00260|38512..38611|Liberibacter_crescens_BT-1_EZ-Tn5_Transposon_Disruption

ATATTCAACAGCTGATGCTCCACAATTAGTGAATAATCTTATAACAGAGCGTGCAGATATGGTCGTGGGGGTGCGTCGTTATAAAAAAAACTATATTGAT

>Intergenic_Region|330313..330412|Liberibacter_crescens_BT-1_EZ-Tn5_Transposon_Disruption

TGTATGGTGGGTAGTTTGACTGGGGCGGTCGCCTCCGAAAGAGTAACGGAGGCGCGCGTGGGTGGGCTCAGAGCGGTCGGAAATCGCTTGTTGAGTGCAA

>B488_12300|1345714..1345813|Liberibacter_crescens_BT-1_EZ-Tn5_Transposon_Disruption

GTTTTCAATATTATCCGTTAAAGACAATAATGCACGAATATAAATCTTATACGCTTCATGCCCAGCATTAAAAATTTCATTCCGGCTTCCTTCAATGGGC

>B488_03770|435664..435763|Liberibacter_crescens_BT-1_EZ-Tn5_Transposon_Disruption

TGATATAATTCTAACAATATTTTCAATATCAGGAATAAAGCCCATATCAAGCATTCGATCTGCTTCATCTATAACGAGTATTTTAATACCACTCATAAGC

>B488_11490|1245823..1245724|Liberibacter_crescens_BT-1_EZ-Tn5_Transposon_Disruption

TATATATAGTGAGCGTCATTTTCGTTTTGATTAAATTAGTTTTTTATGTATATTTTAAAAGGTGTTTCTATGGTAGAAGCAATGTTTGAAGCTGGCCATC

>B488_11810|1289908..1290007|Liberibacter_crescens_BT-1_EZ-Tn5_Transposon_Disruption

ATATAGTACCATACGTGCTATTAGACCAGGCATTGATAATGTATAATCAGAGACCCAATTGATAAACTCACACATCTTACGCGAAATAGGCAAACAATCA

>Intergenic_Region|330816..330915|Liberibacter_crescens_BT-1_EZ-Tn5_Transposon_Disruption

GAATGGATAACCGCTGAAAGCATCTTATCGGGAAACCAACCTGAAAACGAGTATTCCCTATCATAGCCGTGGAAGACTACCTCGTTGATAGGCTGGGTGT

>B488_03270|377160..377259|Liberibacter_crescens_BT-1_EZ-Tn5_Transposon_Disruption

GTTTAGAGATCTTCTTCGTGCATTCTGTGGTCCAGAACTTGGTGTTTTTATATTGATAGTTTCTGTTATTGGAATAACAAGCTGGATGAATACAGCGCGT

>Intergenic_Region|330816..330912|Liberibacter_crescens_BT-1_EZ-Tn5_Transposon_Disruption

GAATGGATAACCGCTGAAAGCATCTAAGCGGGAAACCAACCTGAAAACGAGTATTCCCTATCAGAGCCGCGGAAGACTACCACGTTGATAGGCTGGGCGC

>B488_12400|1356878..1356779|Liberibacter_crescens_BT-1_EZ-Tn5_Transposon_Disruption

AAGGCATCTTTTCTAGATATTGAGACGATGTTTAAGGTAAATAATGAAGAAAGTCCTATTCTCCGCCAAGCCATTACTCTCTCCTTCTATTTCAAGTATT

>B488_05490|585871..585971|Liberibacter_crescens_BT-1_EZ-Tn5_Transposon_Disruption

GTGGTAAGCTGTTCCGGCGATAAACTCATGATTCAAATCCCTCGTAATAAACCAATAAAATTTAATTAAAAATCGAATAAAACAGGTTAAAATAACAACA

>Intergenic_Region|330816..330915|Liberibacter_crescens_BT-1_EZ-Tn5_Transposon_Disruption

GAATGGATAACCGCTGAAAGCATCTAAGCGGGAAACCAACCTGAAAACGAGTATTCCCTATCAGAGCCGTGGAAGACTACCACGTTGATAGGCTGGGTGT

>B488_01000|116614..116713|Liberibacter_crescens_BT-1_EZ-Tn5_Transposon_Disruption

ATATACTTCTAAAGAAGTTACCGCTTATACTATTAACATTTTAAAAGATGATATCTCTTTAGCTGTTGATATACTTGGAGATATGTTAAGTAATTCTTTA

>Intergenic_Region|330816..330915|Liberibacter_crescens_BT-1_EZ-Tn5_Transposon_Disruption

GAATGGATAACCGCTGAAAGCATCTAAGCGGGGAACCAACCTGAAAACGAGTATTCCCTATCAGAGCCGTGGAAGACTACCACGTTGATAGGCTGGGTGT

>B488_13630|1484856..1484757|Liberibacter_crescens_BT-1_EZ-Tn5_Transposon_Disruption

GAATTAGAATCGTTAGCTATTATATCCCATAATGTTTCAGGCATAGAAACATCAAGTCCTGGAAGGATTATTGCACCATTAGGGTCTTTGGCTATTACTG

>B488_09250|998122..998195|Liberibacter_crescens_BT-1_EZ-Tn5_Transposon_Disruption

ACTGAATCTTTTCTATTATATATGAGCTCTTCTCCTTTGTTTCCATATTATTCTTTATTTCTTTTGGATGGGTAACTGTTAATGCATTATACCACCAAAA

>Intergenic_Region|330818..330916|Liberibacter_crescens_BT-1_EZ-Tn5_Transposon_Disruption

GTATGGATCACCGCTGATGCATCTTATCGGGAAACCAACCTGAAAACGAGTATTCCCTATCATAGCCGTGGAAGACTACCACGTTGATAGGCTGGGTGTG

>Intergenic_Region|897747..897845|Liberibacter_crescens_BT-1_EZ-Tn5_Transposon_Disruption

CAATTAACAACAGAAAAAAACTGACAGATTGATATACCATGATCTATGCTCTATACTTATAGGTGAATAATTCGACTTATAGCAGTTTATAATGTAAAGG

>B488_07060|786566..786503|Liberibacter_crescens_BT-1_EZ-Tn5_Transposon_Disruption

GTGCAAACCCACTCTACCTTTTCACGGTCAGGCAGCGCCCTAAAGAACGGCTTCAGATGATCCTTCAACTCAGCAAAAGTTAAGATCAAACACGTTGTTT

>B488_02220|256304..256403|Liberibacter_crescens_BT-1_EZ-Tn5_Transposon_Disruption

GCTATGACATTTTGCATGAGTGCGCATTGCTCTCTTGATGCACCGGCTGGTTTTTCACAGAGTATTGACCATGCTCCATGTTTTGATTTTACAACTCCTG

>B488_05820|637873..637972|Liberibacter_crescens_BT-1_EZ-Tn5_Transposon_Disruption

ATCATAGACAATGCCGCTACCAACATTCAAAACCGCTTTGTTTTGTCTGAAAAGACTCAGTGTGCGGATAGCAACTGAAAAACACATTGTATTTTGTGGT

>Intergenic_Region|330816..330915|Liberibacter_crescens_BT-1_EZ-Tn5_Transposon_Disruption

GAATGGATAACCGCTGAAAGCATCTAAGCGGGAAACCAACCTGAAAACGAGTATTCCCTATCAGAGCCGTGGAAGACTACCACGTTGATAGGCTGGGTGT

>B488_00260|38512..38611|Liberibacter_crescens_BT-1_EZ-Tn5_Transposon_Disruption

ATATTCAACAGCTGATGCTCCACAATTAGTGAATAATCTTATAACAGAGCGTGCAGATATGGTCGTGGGGGTGCGTCGTTATAAAAAAAACTATATTGAT

>Intergenic_Region|329076..329173|Liberibacter_crescens_BT-1_EZ-Tn5_Transposon_Disruption

GCCTGGATAGGCTATGGGGGCTCACCGCCTTACTGCTCCTAACCAAACTCCGAATACCGAGGAGTACTATTTGGCATACACACGGCTGGTGCTAACGTCC

>B488_08390|916173..916074|Liberibacter_crescens_BT-1_EZ-Tn5_Transposon_Disruption

GATCTGTTGTCGGACAGGTGATGGCTCAACATCCCGAGGTAGATATGGTATCATTCACAGGCTCAAGAAGAGCAGGTGTTGAGGTTGCAAGGGCAGCTTC

>B488_05670|616017..616116|Liberibacter_crescens_BT-1_EZ-Tn5_Transposon_Disruption

ATATTATTGATAGCCAGAATCTTTGGGATTTGGATAGTCAGCTGGAAATGGCTATGGAGGCCTTACGCTGTCCGTCTGGTGATGCAGGTGTACAAGAACT

>B488_04570|514148..514247|Liberibacter_crescens_BT-1_EZ-Tn5_Transposon_Disruption

GACCTGGCTACTACAAAGTAAGTCGATAGTGGTATCGTTTTTTTTTGTTAGCCAGAAGATATCACCAAAAATTTTTATTTTTTTGTTCATATTGCATCAA

>B488_05530|599828..599728|Liberibacter_crescens_BT-1_EZ-Tn5_Transposon_Disruption

GCTATATATAGCAAGACAAAACACATTAGTAAGCTTTGAGACAATAGTTGAGGATAAGCCATCAGAGGGAGTATCAAGAGGGTATAATAAACGTTGTGGC

>Intergenic_Region|330818..330915|Liberibacter_crescens_BT-1_EZ-Tn5_Transposon_Disruption

GGATGGATAACCGCTGAAAGCATCTAAGCGGAAAACCAACCTGAAAACGAGTATTCCCTATCAGAGCCGTGGAAGACTACCACGTTGATAGGCTGGGTGT

>B488_08600|942875..942776|Liberibacter_crescens_BT-1_EZ-Tn5_Transposon_Disruption

GGTTTATTAATGGTATTGGAGGTGAGCGTGATTATAAGAAAGGCTTTGAATGGCTTTCTATTGCCGCTAATCGAGGGAATGTTTTGGCTCATAATAGATT

>B488_12800|1395207..1395108|Liberibacter_crescens_BT-1_EZ-Tn5_Transposon_Disruption

GGATGTTGTTGTTCGTTCAGTCCGTTCAGGTTTGCCATTAATAGATGCGATCAGATTGATTGCCACGCAATCGAAACAGCCTCTCAGAAATGAATTCCGT

>B488_08600|942875..942848|Liberibacter_crescens_BT-1_EZ-Tn5_Transposon_Disruption

GGTTTATTAATGGTATTGGAGGTGAGCGCTCTTCCATGAGGCGATTTGATTTGCTTTCTAGCCACGCTTGCTGATGATTTGCTTTGATTTGAATTCAAGG

>B488_05060|550575..550478|Liberibacter_crescens_BT-1_EZ-Tn5_Transposon_Disruption

GTTGGCGATCCAGTGCTTTTTCTCTTGCCTGTGCCTGTGCCTTTGCCTGTGTCGTTGCCTCTTCTGGATCAGTTTGCCCGGCTGCTGCCTGCAAGCGTTG

>B488_03380|389741..389840|Liberibacter_crescens_BT-1_EZ-Tn5_Transposon_Disruption

ATCTTAAGGGATATGTTCTTGACCTTCGTCTTAATCCTGGAGGCTTTCTAGATCAAGCAATTAGTGTTGCTGATTATTTCTTAGAAAAAGGGGAAATCGT

>Intergenic_Region|330816..330915|Liberibacter_crescens_BT-1_EZ-Tn5_Transposon_Disruption

GAATGGATAACCGCTGAAAGCATCTAAGCGGGAAACCAACCTGAAAACGAGTATTCCCTATCAGAGCCGTGGAAGACTACCACGTTGATAGGCTGGGTGT

>B488_05410|583785..583884|Liberibacter_crescens_BT-1_EZ-Tn5_Transposon_Disruption

ATCAATATCGTGTCTTTGAAGATCTTGATAGGATAAGACTGCAGGTTCAAGGATTGCAGAGGCTTCCTGGAAGGAAGGGGCAGACAGGTCCTCCTCAACA

>B488_09530|1024719..1024818|Liberibacter_crescens_BT-1_EZ-Tn5_Transposon_Disruption

TCCATATCAGCATCTACCAATCGACCGATCCCTTTTTTGATGGAATCTTGCAAAGTATGTATAAAATCTTCCTGTAGTTTAATACGAGAATGTATGGAAC

>B488_00090|11762..11663|Liberibacter_crescens_BT-1_EZ-Tn5_Transposon_Disruption

GTAATAAATAGCTCCCCAAATCAATATCTGTCCGCTCAAACAATGATACAAGGGGTGTTATGAGCCATATTTCATGCATTGGGTATTGAGCTGGCTTCTT

>B488_05140|555712..555613|Liberibacter_crescens_BT-1_EZ-Tn5_Transposon_Disruption

ACATCTGCCAGTGCATTCCTGCCAAGAGGGCGTGCAATCTGATAAATCCCTTTCAGGGAAAAATCAGTGGCAACATCAGGATTATTTCCTGCATAAACAG

>Intergenic_Region|1287836..1287867|Liberibacter_crescens_BT-1_EZ-Tn5_Transposon_Disruption

ATCTGGTGGTCACAGAAAGGATCATGATCCTTCAACTCAGCAAAAGTTCCACACTCAGCAAAAGTTCGCTGAGCGCAGGTCACAAGACAAGTATTAAATG

>B488_03410|393579..393479|Liberibacter_crescens_BT-1_EZ-Tn5_Transposon_Disruption

CCATTATGTGCACCGTCTCGCGCATCATTCATGACAGGCCAACTTATTTCTGAAACGAGGTCTATGATAATGCTGCCGAATTCCATGCCGATAAACCTAC

>B488_05060|548890..548989|Liberibacter_crescens_BT-1_EZ-Tn5_Transposon_Disruption

ATCTCATGCTGTTTCCCGGGACATTCGTGGAGTGATTGCGAAGATTTTACCATCATTACGACGTGTTTTGATAGGAAGCGACAAGGTTGTCGAGTATCAG

>B488_05550|594001..594100|Liberibacter_crescens_BT-1_EZ-Tn5_Transposon_Disruption

GTACTTGTCCCAACCGTATAAAAGCGAATGCACCTCTGTATCGCCAACAGATCCATGCTCTGTTATCTTTATGAGATCGGTGACCAGATCTTTTTGGTAC

>Intergenic_Region|329143..329044|Liberibacter_crescens_BT-1_EZ-Tn5_Transposon_Disruption

CTAGTACTCCTCGGTATTCGGAGTTTGGTTAGGAGCAGTAAGGCGGTGAGCCCCCATAGCCTATCCAGTGCTCTACCCCCGAGGGTATTCAGTTAACGCT

>B488_06680|740522..740621|Liberibacter_crescens_BT-1_EZ-Tn5_Transposon_Disruption

GACCTGGTAATACCAAGAACCGACGACCGCGCCAATCAATGGAGAAGCCTGGTGCTGTCCCATTGGGATTATGCAAAATAGTTGCCGTTTCTGGAAAGAA

>Intergenic_Region|330816..330915|Liberibacter_crescens_BT-1_EZ-Tn5_Transposon_Disruption

GAATGGATAACCGCTGAAAGCATCTAAGCGGGAAACCAACCTGAAAACGAGTATTCCCTATCATAGCCGTGGAAGACTACCACGTTGATAGGCTGGGTGT

>B488_11840|1292689..1292788|Liberibacter_crescens_BT-1_EZ-Tn5_Transposon_Disruption

CACTAATCCTTGTTATTGTGAGCAGCTTTGATTACTACTTTGAGATGATTAGTACAAAATCTAATTCAATTATTGCGCTTAATAGTTTCTCTGCATTGAT

>Intergenic_Region|330816..330915|Liberibacter_crescens_BT-1_EZ-Tn5_Transposon_Disruption

GAATGGATAACCGCTGAAAGCATCTAAGCGGGAAACCAACCTGAAAACGAGTATTCCCTATCAGAGCCGTGGAAGACTACCACGTTGATAGGCTGGGTGT

>B488_01190|140425..140524|Liberibacter_crescens_BT-1_EZ-Tn5_Transposon_Disruption

ACTTATATGTGACTAGAGGGCTGCTTGCTCTTTGTAATGATGCATCAGAAGTTGCTGCGGTTTTGGCCCATGAAATGGCACATATAATTCTTAATCATGG

>Intergenic_Region|330865..330915|Liberibacter_crescens_BT-1_EZ-Tn5_Transposon_Disruption

ATCTGGTGGTCACAGAAAGGATCATGATCCTTCAACTCAGCAGAAGTTCAGTATTCCCTATCAGAGCCGTGGAAGACTACCACGTTGATAGGCTGGGTGT

>B488_01000|116614..116713|Liberibacter_crescens_BT-1_EZ-Tn5_Transposon_Disruption

ATATACTTCTAAAGAAGTTACCGCTTATACTATTAACATTTTAAAAGATGATATCTCTTTAGCTGTTGATATACTTGGAGATATGTTAAGTAATTCTTTA

>B488_13500|1471296..1471248|Liberibacter_crescens_BT-1_EZ-Tn5_Transposon_Disruption

ATCTGGTGGTCACAGAAAGGATCATGATCCTTCAACTCAGCAAAAGTAATGAGAATGAACAAATGATGCATGTAGTCCATTGTTACCAGTAGAGATCCCT

>Intergenic_Region|330816..330915|Liberibacter_crescens_BT-1_EZ-Tn5_Transposon_Disruption

GAATGGATAACCGCTGAAAGCATCTAAGCGGGAAACCAACCTGAAAACGAGTATTCCCTATCAGAGCCGTGGAAGACTACCACGTTGATAGGCTGGGTGT

>B488_12300|1346477..1346378|Liberibacter_crescens_BT-1_EZ-Tn5_Transposon_Disruption

ACCTTGTAGAGGCTTTCAAGATTATTTTTCAAGGTCGAGTAGATGATGATTTATTCAATCGTTTGGTGATTTTATCAGGATTAAGAGTCTATGAAGTTAA

>B488_09240|997881..997980|Liberibacter_crescens_BT-1_EZ-Tn5_Transposon_Disruption

GGTAAAGGTACTTCCGTTACAATAACCTTACCATTAAATGAAAAGATCTAAACAGAAAGAAAAGGAGTCGTTTCTACGTTTTATTTTCCTATCGGTTTAC

>B488_08600|942781..942880|Liberibacter_crescens_BT-1_EZ-Tn5_Transposon_Disruption

ATTCTGAGCCAAAACATTCCCTCGATTAGCGGCAATAGAAAGCCATTCAAAGCCTTTCTTATAATCACGCTCACCTCCAATACCATTAATAAACCAAAGA

>B488_01230|149889..149988|Liberibacter_crescens_BT-1_EZ-Tn5_Transposon_Disruption

GTTTCAGAAGATGCACGGTCAGTATCATCGGGATGATGAATATTTTCTTTAGAAAGAGATTGCAAGGTTTCATTTAATTCACTAATAATATCATTTTTCC

>Intergenic_Region|865933..866013|Liberibacter_crescens_BT-1_EZ-Tn5_Transposon_Disruption

TGTACGAAGTCAGCTGATAGGGAGAAAGCACATGTGATTACCTAATAACATGTTGGCATATTACTTTGATCCTTCAACTCAGCAAAAGTTCCAGTTTCTA

>B488_10700|1167533..1167434|Liberibacter_crescens_BT-1_EZ-Tn5_Transposon_Disruption

AAATTGACTTTCATCAGACATATCAATTTTTAAACGATATTATCCTGGGAAAATTTAAAAAACTACCTGCGTGGAAGAAAATTTCTAAAAACAGGCGTTC

>B488_10620|1157669..1157577|Liberibacter_crescens_BT-1_EZ-Tn5_Transposon_Disruption

GTTGTATATTCCAGTTTCTTTGACTTGTTGTATTGGTGGCTCCCTTCAACTAAAGATTTTTTAAAAGTATCTGAATTAACGAATAAGTTGCGCGTCCCCC

>B488_01000|116614..116713|Liberibacter_crescens_BT-1_EZ-Tn5_Transposon_Disruption

ATATACTTCTAAAGAAGTTACCGCTTATACTATTAACATTTTAAAAGATGATATCTCTTTAGCTGTTGATATACTTGGAGATATGTTAAGTAATTCTTTA

>Intergenic_Region|671978..672075|Liberibacter_crescens_BT-1_EZ-Tn5_Transposon_Disruption

AGCCAAGAGAGTATCAAATTCTCTTAATGATATTTATAACTTTCTATCATGTATTTCGATTATATTCTGTGTATCTGCAACCCATTAAATTTTTCGAATA

>B488_05770|633112..633211|Liberibacter_crescens_BT-1_EZ-Tn5_Transposon_Disruption

TTATAATATAGCTAAAAAGTTATAAAAACTTTATCAGAAAAGATACTTTATTTTATGATGACTACAGCAGTTCAGTCAGCTATTCGTAGTATTTCAATAG

>B488_01230|149897..149798|Liberibacter_crescens_BT-1_EZ-Tn5_Transposon_Disruption

TTCTGAAACAGACCGCTCTGTTCAATTACGCGCTCGTGATAGACAACGCAAGCTCATTTCTAAAATAGACGCTGCATTACAGCGCATAAATGACTCAACT

>B488_09000|686284..686383|Liberibacter_crescens_BT-1_EZ-Tn5_Transposon_Disruption

TGGCATAATCAACACCAACGCCTACTGTCGGCCCGTTGAATGTTTTCTTATTGATCACTGAATTAATGTCCTGAGTGAACTTGGCCTTGCTAAAAGACCA

>B488_12810|1397088..1396989|Liberibacter_crescens_BT-1_EZ-Tn5_Transposon_Disruption

GTTTGAAGCTAATAATAAATTATCTGATTCTTTCGTGACAGAAACACCATCTTCTAGAGCTTTTAAGGTGGTTTCAGATCGATCAAATGTTCGTGATCCT

>B488_05170|558849..558750|Liberibacter_crescens_BT-1_EZ-Tn5_Transposon_Disruption

GCCTTGAATGTGTAAGCAGGTGTGCCCAGAGCCTGCTGAACCTGTGACCCCTGTTGGCCCGCAGAAGGCAACATCGGATTGTTCTGAAGAGCAGACATGA

>B488_01000|116901..117000|Liberibacter_crescens_BT-1_EZ-Tn5_Transposon_Disruption

CTTATATGTCACGTAATTATGTAGCAGACCGTATATTTATAGTCGCTGTTGGAGGAGTTGATCATAATTTTCTTGTGAAGAAAGTAGAAGAATGTTTTAA

>Intergenic_Region|651852..651753|Liberibacter_crescens_BT-1_EZ-Tn5_Transposon_Disruption

ACCCTATTGTGCACCATATTAATAGGTGAATAACGGTAATGGCTTCTTGATTTAAGTAAAGAAAAGTGCAAAATAAATTAAGTGGTTTATACTTTAAAAT

>Intergenic_Region|329143..329044|Liberibacter_crescens_BT-1_EZ-Tn5_Transposon_Disruption

CTAGTACTCCTCGGTATTCGGAGTTTGGTTAGGAGCAGTAAGGCGGTGAGCCCCCATAGCCTATCCAGTGCTCTACCCCCGAGGGTATTCAGTTAACGCT

>B488_13380|1455363..1455264|Liberibacter_crescens_BT-1_EZ-Tn5_Transposon_Disruption

ATTGAAGCCTTAGAGATGGAATTAAAGCTGAGATCAGCAAAAGAAAGTCTTATCAAAGCTAAAGCAAACGCTAATAATGCTTTTAATATGGCTCTTAGAA

>B488_03730|429058..429157|Liberibacter_crescens_BT-1_EZ-Tn5_Transposon_Disruption

GTTATACTCTCAGTGAAGGTGATGCTCTTAAATATGTTGCCTCTTATCCGGATTTGATAGAAGATTTTTTGAGGAAAGGTCTTTCTGATAAACAAGCTAT

>B488_03860|451031..451130|Liberibacter_crescens_BT-1_EZ-Tn5_Transposon_Disruption

TTGTTATATACACTTCGATAAGACCACGTGCATGAAGTTGATAAATCTCATTTCGTAATCTTTTTTCAGGGATGTGATCATCTATAACAATTAAGTTATA

>B488_03730|429058..429158|Liberibacter_crescens_BT-1_EZ-Tn5_Transposon_Disruption

GTTAACTCTCAGTGAAGGTGATGCTCTTAAATATGTTGCCTCTTATCCGGATTTGATTTAAGATTTTTTGAGGAAAGGTCTTTCTGATAAACAAGCTATT

>B488_04890|538552..538651|Liberibacter_crescens_BT-1_EZ-Tn5_Transposon_Disruption

AACCATAACCCAATAACCAATAACCAAAATAAAAATAATAAATCTTCACTTCGTTCAGATTTGTCGGTTTCTGATTTTGAGGATTTTGACAATCACACTG

>B488_05470|593005..592906|Liberibacter_crescens_BT-1_EZ-Tn5_Transposon_Disruption

ATATTAACCTGGGCAATCGTTATTTCAGGACGAACAGAGGTACCTTCTGCAGAAATTATTGATGCAAAAAAAGAACTCATCGAATGGCCTTATGCCAATA

>B488_06040|667468..667565|Liberibacter_crescens_BT-1_EZ-Tn5_Transposon_Disruption

GCGTTTATGCAATAAAGAAAGTGGTTTCTATTCCTGTTACAATTAAATGTCGTATAGGTATAGATGATCAAGTTCCTGAGCTTGCGTTGCGTAATTTAAT

>B488_13130|1426418..1426319|Liberibacter_crescens_BT-1_EZ-Tn5_Transposon_Disruption

GTGTATTCCTGTGCGGATAGTTCCAATATCACGAGGGCCTCCTCGTCCAAGTGCTAAACGAGATAATGCGCGTGCTATATCGGAAGAAGAGCTTAAAATT

>Intergenic_Region|104039..103940|Liberibacter_crescens_BT-1_EZ-Tn5_Transposon_Disruption

GATATAGCTTGTATAAAAATACAGTTAATTTCTTGGAACTTATACAATATAAAAAGAAACTGATCCATTAGTGAGGATTTTATGCAGAAAATTGATTTTA

>B488_05620|609954..609856|Liberibacter_crescens_BT-1_EZ-Tn5_Transposon_Disruption

TTACTAACCCTACTACCATCCGGTCCCACAAATAAGGCAGCAACAACATGGTGATACCGTAAAACAAACCCTAACGCTAACGAAACAATCAGAAGTCTAA

>Intergenic_Region|329143..329044|Liberibacter_crescens_BT-1_EZ-Tn5_Transposon_Disruption

CTAGTACTCCTCGGTATTCGGAGTTTGGTTAGGAGCAGTAAGGCGGTGAGCCCCCATAGCCTATCCAGTGCTCTACCCCCGAGGGTATTCAGTTAACGCT

>B488_05550|601627..601712|Liberibacter_crescens_BT-1_EZ-Tn5_Transposon_Disruption

ATTAAAGATAAAGGATTAAGCGCTATTACGGCCAATAATTATGCCTATAACAAGCTGAACCATGATCTTGGGCTGGATGATCCTTCAACTCAGCAAAAGT

>B488_06680|740530..740431|Liberibacter_crescens_BT-1_EZ-Tn5_Transposon_Disruption

TACCAGGTCCCCCGCAAGAATGTTTGCCAATTCTGGAAACATTCTTGCAACATCAGCCAAGACTATCGCATGGTATTCAGTACCGTTGGTTACTTTTAGG

>B488_05620|609954..609855|Liberibacter_crescens_BT-1_EZ-Tn5_Transposon_Disruption

TTACTAACCCTACTACCATCGGTCCCACAAATAAGGGCAGCAACAACACGGTGACCCGTAAAACAAACCCTAACGCTCACGAAACAATCAGAAGTATAAG

>Intergenic_Region|330816..330915|Liberibacter_crescens_BT-1_EZ-Tn5_Transposon_Disruption

GAATGGATAACCGCTGAAAGCATCTAAGCGGGAAACCAACCTGAAAACGAGTATTCCCTATCAGAGCCGTGGAAGACTACCACGTTGATAAGCTGGGTGT

>B488_01840|224508..224607|Liberibacter_crescens_BT-1_EZ-Tn5_Transposon_Disruption

TCTTTATCCATAAAAGGAATGACGTCACCTACGGATAAGTGTAAAATATTTCTAGGTGTAATTGTCTGTAGTTCTATTCGTGCTTCTATATACACATCAA

>B488_01920|232357..232456|Liberibacter_crescens_BT-1_EZ-Tn5_Transposon_Disruption

ATTGGAAGTGAAGTGGTTATAGCTCTTGAAGATTACCGTTTTCAAGAAGGAATATATGATCAAATTGTTTCTATTGAAATGTTTGAGGCGGTTGGAGAAG

>B488_06020|665474..665574|Liberibacter_crescens_BT-1_EZ-Tn5_Transposon_Disruption

CTCCTACAGTTCCTTTGAAAAAAACATTGGAATGAAGCCAGGGATAATTACAGTCGAGAATCGAAGAGAAACAATACAATAATTCGCAAATAACCGTCCT

>Intergenic_Region|216013..216114|Liberibacter_crescens_BT-1_EZ-Tn5_Transposon_Disruption

AGTTAAATCATTGCGGGACCTTCGCTTATCGGTTGATATGATACTGAATCTGCAGTCAAGTCCGCATCCAAACCTATTTCACTCGGCGCGATACTTAACG

>B488_05540|600839..600740|Liberibacter_crescens_BT-1_EZ-Tn5_Transposon_Disruption

CAGGAAGAGATTGCTAAGACAAGCCGGGATAAAGAAGAATAAGAGAGCAACTGTCATTGCGGGAAGTAATATTATATATAGGCAGACAAAACACATTAGT

>Intergenic_Region|691060..690961|Liberibacter_crescens_BT-1_EZ-Tn5_Transposon_Disruption

CTTTTGGCGTACAAACAAAAATTCATACCACCACCCAGTTATCCAAAACAATCGTGCGTGTACAGTAAGATTTTACTCGATTTAAAGTATATAATAATCA

>Intergenic_Region|642732..642634|Liberibacter_crescens_BT-1_EZ-Tn5_Transposon_Disruption

GTGACATAGAGGCAAAAATATCACATGTCTTCCCATAATTCCATAAATCCCCCCAAGAAACCATGTATTCATCAAATTTTACGAGAACATCATTCTCTCC

>B488_05930|653190..653091|Liberibacter_crescens_BT-1_EZ-Tn5_Transposon_Disruption

GGACAACTGTAGTAATTAATTCTTTTTGAAAACGTTCTGCTACACCTGCTGAAAGTGTTACACGAATAGACTTTTCAGCAGTAACTTGTATATCAGAATT

>B488_04890|538552..538651|Liberibacter_crescens_BT-1_EZ-Tn5_Transposon_Disruption

AACCATAACCCAATAACCAATAACCAAAATAAAAATAATAAATCTTCACTTCGTTCAGATTTGTCGGTTTCTGATTTTGAGGATTTTGACAATCACACTG

>B488_07700|849213..849312|Liberibacter_crescens_BT-1_EZ-Tn5_Transposon_Disruption

GACAGCCTGACCCTTTATATGAATCTTGTTCCAACGTAAAAATTGATTGGCTGCATCCTTAAAAGACCCAAGATTAAGATAGTGGAGCAGCGTAGAACCC

>B488_09530|1025028..1025127|Liberibacter_crescens_BT-1_EZ-Tn5_Transposon_Disruption

GCATCAGTAAAAACTGATACTTTTCCAATTTCCTTTGCACCAGCAGAATCCTTCACCGTGAGCTCTGTCTCTGAGGCCTTAACCCAAACATCATCTTTTA

>B488_11650|1270239..1270140|Liberibacter_crescens_BT-1_EZ-Tn5_Transposon_Disruption

GCCCTATTGTTGAAAGGAAGCGCTATAATTTTTATTGATGGCCGGTATAATCTTCAGGTGGAAGAAGAACTTGATATGACATTATTTTCTAAACTGGATC

>B488_11620|1265760..1265661|Liberibacter_crescens_BT-1_EZ-Tn5_Transposon_Disruption

TCATTGAGGTATCAGAAAGTCCTTATCGATTTTTGGTTAGGCGTGCAGTCCGCAACTGCCGACGGCGCCCTGGTAACGGCATTCGGGGGCACCGTAACCA

>B488_13090|1419847..1419946|Liberibacter_crescens_BT-1_EZ-Tn5_Transposon_Disruption

GTGTCAGGCTGAAGGTTAATAGGCGCGTAGCGTCTGAAATACCGTAGCCAGTTTATGATGATCTTTAATACCCGGCGCGCTTTCCACACCGGAGTTGAAG

>B488_07580|842762..842861|Liberibacter_crescens_BT-1_EZ-Tn5_Transposon_Disruption

GCTCTATGAATGTCACAAAACAGATCTACAAAGCCTTTTCATCCAAATCTTATGATGAAGCAAAAATCTCTGTTCCTGCCAACAGGAAACATGATGACAT

>B488_13110|1422375..1422276|Liberibacter_crescens_BT-1_EZ-Tn5_Transposon_Disruption

CTTTAACGACATGGTGATGGCGGTACGCAATGATGCCGATCGGATCTGCGGCTTTCAGTTCCACCCTGAATCCATTTTAACCACTAAGGGAGCACATTTG

>B488_01840|224508..224607|Liberibacter_crescens_BT-1_EZ-Tn5_Transposon_Disruption

TCTTTATCCATAAAAGGAATGACGTCACCTACGGATAAGTGTAAAATATTTCTAGGTGTAATTGTCTGTAGTTCTATTCGTGCTTCTATATACACATCAA

>B488_07730|852195..852096|Liberibacter_crescens_BT-1_EZ-Tn5_Transposon_Disruption

TCTGGGAACAGATGATGCCAGCCATATTATCAAGGGGAAACTTCCTCAAATCTGTTTGCCTTTTCAGCCTGTTCAACAGTCTGGAGGGATTGGACAACAG

>B488_04880|538099..538198|Liberibacter_crescens_BT-1_EZ-Tn5_Transposon_Disruption

GTATGACATCGATGTGGAGGTGGTGAGATCATCATGAAGTATTATTCGTTCCATATTGGAGACTACCGCACGGATGCGTTTAATCTTCCATGGGATTATC

>B488_06780|751275..751327|Liberibacter_crescens_BT-1_EZ-Tn5_Transposon_Disruption

GTGCAGCTCAGTATACCTGGAGAGTAATAGTCAAGCAAATATTTATGATCCTTCAACTCTTCAAAAGTTCATGATGTATTTGTAGAAGATTGGGTGGAGC

>B488_02030|239064..239163|Liberibacter_crescens_BT-1_EZ-Tn5_Transposon_Disruption

GCTCAAGGTTCAATAGTTGTTTCTGGAGTTTCTGTAAAAGGGAAAAAAGCTTCAGTTTTTGAAGGGATAACAACAAGTGGACGTATTATTAATGGTGCAA

>B488_03090|355169..355087|Liberibacter_crescens_BT-1_EZ-Tn5_Transposon_Disruption

GAACAGGGCAAAGCCATTTTTTATATAAAGGTGAATATGAAAAAAATGCTTTTGATAGGATTTTTTTGATCTCGGGTATTTGAAAAAGGCTCTTGCTAAT

>B488_03400|391705..391804|Liberibacter_crescens_BT-1_EZ-Tn5_Transposon_Disruption

GTATTATGGTTTTAAATCATGATGGGCTTATATGGTTGGGGCGTCGTTGTATAAAAAATAATATCAGAGAGGATGTTGGTTTTTTATGGCAAACGCCTCA

>B488_00130|17230..17329|Liberibacter_crescens_BT-1_EZ-Tn5_Transposon_Disruption

GTCATACACCTGTAGAAACAGAAGCTCTTTTAAAAATCATGGAGTATGGTGCATGGATTGATTTAATAAGATTACATATCCCAGAACATCAAAAAATTTA

>B488_09920|1083364..1083265|Liberibacter_crescens_BT-1_EZ-Tn5_Transposon_Disruption

TAGCAGCACAGAGGATAGAAAACTATCATGTCAATCAAATTCCTAAAGATGATCTTTACCAAGATGAAATAGGGGTCAAGCTTGGTTGTTTTTGGCGAGC

>B488_12300|1345714..1345813|Liberibacter_crescens_BT-1_EZ-Tn5_Transposon_Disruption

GTTTTCAATATTATCCGTTAAAGACAATAATGCACGAATATAAATCTTATACGCTTCATGCCCAGCATTAAAAATTTCATTCCGGCTTCCTTCAATGGGC

>B488_07050|783239..783140|Liberibacter_crescens_BT-1_EZ-Tn5_Transposon_Disruption

GTCATATGGTAGGCGGTCATTGCGGTATTGATCCATGGCGGCTTCGCAGGACTGGGTGAAGTCAGTCTTCAATTCGACCGTAGCCACAGGCAGGCCATTG

>B488_05910|650317..650416|Liberibacter_crescens_BT-1_EZ-Tn5_Transposon_Disruption

GTTGAAGATCACGTTTCCATGTTCCATGGTGTTACTTTGGGTGGTACAGGCAAGGAGCGAGGAGATCGTCATCCAAAGGTTCGTAAAGGAGTTATCATTG

>B488_02990|346316..346415|Liberibacter_crescens_BT-1_EZ-Tn5_Transposon_Disruption

GTTCCATGGTTGCTAATAATCAGGCGCAAGTTATTACTGATTTTCATCAGGATGAGGAAGGTTTTACTCGGATATTGGTGGTGGATTATGGTATGTCGTC

>B488_05910|650317..650416|Liberibacter_crescens_BT-1_EZ-Tn5_Transposon_Disruption

GTTGAAGATCACGTTTCCATGTTCCATGGTGTTACTTTGGGTGGTACAGGCAAGGAGCGAGGAGATCGTCATCCAAAGGTTCGTAAAGGAGTTATCATTG

>B488_08910|968730..968631|Liberibacter_crescens_BT-1_EZ-Tn5_Transposon_Disruption

GGATATGGGCTCTTCAGTGAGAGAGCGTACGACTATTGCGGATTATATAAAAGATGGGCTTGATGTTCTTGCACAACATTGGGGGTACAAGAGCTCATAA

>B488_12300|1345941..1345842|Liberibacter_crescens_BT-1_EZ-Tn5_Transposon_Disruption

GGATTACGGTGGTCAGATCGAGCAGAAGATTATCGTACTGAAGCGTTAGGTCTGGTAAAGGCACAAAGAGTTAAGAATGCAGTGATTGTTCCTGTGGGAG

>B488_06040|667468..667565|Liberibacter_crescens_BT-1_EZ-Tn5_Transposon_Disruption

GCGTTTATGCAATAAAGAAAGTGGTTTCTATTCCTGTTACAATTAAATGTCGTATAGGTATAGATGATCAAGTTCCTGAGCTTGCGTTGCGTAATTTAAT

>Intergenic_Region|216013..216112|Liberibacter_crescens_BT-1_EZ-Tn5_Transposon_Disruption

AGTTAAATCATTGCGGGACCTTCGCTTATCGGTTGATTATGATACTGAATCTGCAGTAAAGTCTGTCATTTAAAATTATATTTCACTTGGCGTGATACTT

>B488_05060|550730..550631|Liberibacter_crescens_BT-1_EZ-Tn5_Transposon_Disruption

GCCCTCCAAAGTGCACACTTCCAGACATGCCTGAAGGAGCCAGGGCGATCTTGTCCGCCTGTTCCATATCAAGCTGTTTGTTCTTCAGGGCTGTTTCTGT

>B488_09670|1042957..1043056|Liberibacter_crescens_BT-1_EZ-Tn5_Transposon_Disruption

GTAACATCTGCAGCTGTCGATATGCAGACCCCGAGATATTTGACCTTCCTGAAACAAAATATTTCCAAATAATGCGATCGCTTTAATATGACCTCTTTTA

>Intergenic_Region|329143..329044|Liberibacter_crescens_BT-1_EZ-Tn5_Transposon_Disruption

CTAGTACTCCTCGGTATTCGGAGTTTGGTTAGGAGCAGTAAGGCGGTGAGCCCCCATAGCCTATCCAGTGCTCTACCCCCGAGGGTATTCAGTTAACGCT

>B488_06710|743610..743709|Liberibacter_crescens_BT-1_EZ-Tn5_Transposon_Disruption

TAGCGATGGTGCTTCAGAATCATTTGATTCACAAACCTGGGCAAGGCTAATCGCTTGCCAGTTTGTTGAAAACTCCACTCTTGATGAATTCTGGTTAACG

>B488_05010|545458..545555|Liberibacter_crescens_BT-1_EZ-Tn5_Transposon_Disruption

CCCCAGAAGGACGTGGATTGGAAGCCAAAGAAGCTATTTCATTTCTTATTCTGTTCTCCCGTCCTTGGATTGGATATTCTCAAGGTATTTTAATGATTCT

>B488_08420|920397..920496|Liberibacter_crescens_BT-1_EZ-Tn5_Transposon_Disruption

AGATAAAACTGTTATAGTTATTGCACATCGCCTCTCTACGTTGATTAAAATGGATCATTTAATCATGATTGATCAAGGTCGTGTTATTGAACAAGGCTCT

>B488_01000|117618..117519|Liberibacter_crescens_BT-1_EZ-Tn5_Transposon_Disruption

GTCAAAGGCAAGGTTTCTATAGGTCCCAAAGCCGAAATAGTTGGATTTTTATCCCAAAAAAAGCGACTCGCTAAATCAGTTATATCTTTACAAGTAATAC

>Intergenic_Region|1373320..1373419|Liberibacter_crescens_BT-1_EZ-Tn5_Transposon_Disruption

CTTATAGACATTACAACTTATAAATAATACAATAGTTTTAAATAAGCTACGTCTCTGAAGAAGTTATCTTTAAAAATTGAAATCAGATTACTGTGTTTAG

>B488_13070|1418597..1418498|Liberibacter_crescens_BT-1_EZ-Tn5_Transposon_Disruption

CCATGAAGGCGCATTTGTACCTTTTGTGACGCTTGGCGATCCCAAGCCCACCCTCTCGCTACGCATTATCGATACGCTCATTGACGCCGGCGCCGATGCA

>B488_07050|783385..783285|Liberibacter_crescens_BT-1_EZ-Tn5_Transposon_Disruption

TCCTTGCGCCCCTTGTTGAACGGCACGAATAAGGTGTTTTCCCCGACCTCTTGGTTGCCATCATGATTTCGGAATCCGACATAGCGAAATGCACCACCGC

>Intergenic_Region|329143..329044|Liberibacter_crescens_BT-1_EZ-Tn5_Transposon_Disruption

CTAGTACTCCTCGGTATTCGGAGTTTGGTTAGGAGCAGTAAGGCGGTGAGCCCCCATAGCCTATCCAGTGCTCTACCCCCGAGGGTGTTCAGTTAACGCT

>B488_04720|528529..528429|Liberibacter_crescens_BT-1_EZ-Tn5_Transposon_Disruption

GCTCTATTGTCATAAGCTGGTCTGTCCAATACCGCTCTTTTCAGGTTCTTTCCTAAGATGTTCTCGAGCCATATTTTGCAAGGCATTGGCATTATTTGAA

>B488_03250|375175..375274|Liberibacter_crescens_BT-1_EZ-Tn5_Transposon_Disruption

AAGATAGGGGTAGAAACTGAACTGCGGAATATTAACTCGTCTGTTTTTTTTGGGAGTGATCCTTCAACTCAGCAAAAGTTCCATAGATTTTATGCAGATG

>Intergenic_Region|329143..329044|Liberibacter_crescens_BT-1_EZ-Tn5_Transposon_Disruption

CTAGTACTCCTCGGTATTCGGAGTTTGGTTAGGAGCAGTAAGGCGGTGAGCCCCCATAGCCTATCCAGTGCTCTACCCCCGAGGGTATTCAGTTAACGCT

>B488_09380|1010996..1010903|Liberibacter_crescens_BT-1_EZ-Tn5_Transposon_Disruption

GGATTGTTACACGTATATATGCACTGGGATTACAAGTCTCCGGGACAATCCTCTCTACTGAGATAGGTTTAAATGCCGCATCAGGAATAGATATCCCCGA

>B488_00320|46152..46251|Liberibacter_crescens_BT-1_EZ-Tn5_Transposon_Disruption

ATGCATTTCGCTGTTTCTGATATACAAGCGAATCGCATTAGGAAGGGTAAGTCAATTCTGGTAGATGGATATGAAGATGAAGTTTTTCCAGCAGATGCTT

>B488_05060|549457..549358|Liberibacter_crescens_BT-1_EZ-Tn5_Transposon_Disruption

ATCACGATCATAACCCATCTCAATCAGATCAGAACGCCTGAGCTTTGTCTGTCTCCCGACAAGAACCGCTTCCTTAATTGTCACGGCTTCAGGGGATATC

>B488_09210|993178..993277|Liberibacter_crescens_BT-1_EZ-Tn5_Transposon_Disruption

ATCTTGAGGTTGCTGTATCTCCTGACGATAATAAACTTGAATTACGCAATGTTTGGCGGTTTGATGAAATGTTTACATCATCTGTTATTATAGACTTTGA

>B488_05060|549457..549358|Liberibacter_crescens_BT-1_EZ-Tn5_Transposon_Disruption

ATCACGATCATAACCCATCTCAATCAGATCAGAACGCCTGAGCTTTGTCTGTCTCCCGACAAGAACCGCTTCCTTAATTGTCACGGCTTCAGGGGATATC

>B488_05120|554102..554201|Liberibacter_crescens_BT-1_EZ-Tn5_Transposon_Disruption

GGCTACGACATTTACCTGTACGGTGTGGTTCGCGAGATCGGATTGTGGGAACAGAATACGGATAAGGTGACTTCGGCAATGAAGCTGTATGATGCAGCGG

>B488_02780|315776..315875|Liberibacter_crescens_BT-1_EZ-Tn5_Transposon_Disruption

CTTCTAGGGAGAGTGAAAATGATAAAAAAACTACATGTAGAACATAGGGATGTTCTTAAGCGACTTCGTGCTCTAATAGTTGAACCTCTTGATTATCAAG

>Intergenic_Region|329143..329047|Liberibacter_crescens_BT-1_EZ-Tn5_Transposon_Disruption

CTAGTACTCCTCGGTATTCGGAGTTTGGTTAGGAGCATGAAGGCTTTGAGCCCCCATAACCTATCGTTGGCTCTACCCCCGAGGGTATTCGGTTAACCCT

>B488_03070|353850..353949|Liberibacter_crescens_BT-1_EZ-Tn5_Transposon_Disruption

CTTGCTGCCTCTCCATACTTTTCTCCATCTTGAACCGGAATATTGAGCAGCTTGCAGATAATTTCTACTGGAAGTTTGAAAGCGTATTCAGCTGCTAGGT

>B488_01210|146088..145989|Liberibacter_crescens_BT-1_EZ-Tn5_Transposon_Disruption

CAATAATCTTCATCTTTCTCGCATTTAATACTCGTTTTTTGCTCTAATATAAATGGATTATTCGTTTTTCCATGACCAAAGTATGGATAATAATAAATAG

>B488_05170|559882..559783|Liberibacter_crescens_BT-1_EZ-Tn5_Transposon_Disruption

GCTATATTCATTGCTCAAAACATGCGAAGCTATGTCAGCCCTTCCCTGTCTGTATTGTACAGCCTTGCGTAAATTATCGATAATAAGCCTGTTGCCTTCA

>B488_09990|1093354..1093453|Liberibacter_crescens_BT-1_EZ-Tn5_Transposon_Disruption

CCCCTACACAGCCCAAAGTAAGTATTTTATATAATGCCTGAACATGATCATCAACATATAGCCAATTACGTATATTTTTACCATCTCCATAAACAGGAAT

>B488_06170|680158..680252|Liberibacter_crescens_BT-1_EZ-Tn5_Transposon_Disruption

CTATCATCCTTTGAAGATGTTAATTCTTCATGCAATATCTGGAGCTCAAGCCATTGTTCTTGTTTGTCTTCCAGGTCTTTGTATAATAATACAAGGGGTG

>B488_03730|429424..429325|Liberibacter_crescens_BT-1_EZ-Tn5_Transposon_Disruption

ACATATTCTCCGCCATCAAAAGCATCATGACGATACCCTTTCTTATAACCTTTATTAATATGATATAAGGAAACGTAATCATTCAGTTTCTTTAAAGCGA

>B488_13390|1456104..1456017|Liberibacter_crescens_BT-1_EZ-Tn5_Transposon_Disruption

GCTGACGCTGGTCCTCGGGAAGTTGTATTTCTTTGCAAGAATCACTGATTGGCACCTATAGATATAGAGTAAACAAAAAAGTCCAGATTTCTTTACCAAA

>Intergenic_Region|249988..250087|Liberibacter_crescens_BT-1_EZ-Tn5_Transposon_Disruption

CATCAGAGTTGAGGGCGTGGAGTTGTTTATTTTATAATATTGTACTGTAAGAGAGGGAGTATGATTGCTTTTTTATGAAGGAGTGGTCGGAGCGACTGGA

>B488_08670|946522..946424|Liberibacter_crescens_BT-1_EZ-Tn5_Transposon_Disruption

GCATTATACATCATTAAGAAATATTTTATTATAAAAATGTGGCATTCTTCCTCAAAAATTACAAAGATTTTATTATGAAGAAAACAATAATATAAAAACT

>B488_12770|1391337..1391434|Liberibacter_crescens_BT-1_EZ-Tn5_Transposon_Disruption

CCATATTGATCCTTTTTCTACATAACCAATACAACTAGCGGGGACAAGTCTCTGATACTGGAGCAGACCTCTCAAATGCTTCCTGCAGCCTCCCAAGGAT

>B488_01000|116661..116715|Liberibacter_crescens_BT-1_EZ-Tn5_Transposon_Disruption

GTACTATTATCTCGATTGGCTTGATATATCAACTCCATTTTGTTCATGATATCTCTTTAGCTGTTGATATACTTGGAGATATGTTAAGTAATTCTTTACT

>Intergenic_Region|801289..801191|Liberibacter_crescens_BT-1_EZ-Tn5_Transposon_Disruption

GTGCAACACTGATGTTTTTTGTATCTTTCATTATAGGATTAAAGAGTCAGCTGGTTTTTTTTCTAAAAAACACTGTTTTTTCTGGTAATGATCAGAACAC

>B488_01000|116614..116713|Liberibacter_crescens_BT-1_EZ-Tn5_Transposon_Disruption

ATATACTTCTAAAGAAGTTACCGCTTATACTATTAACATTTTAAAAGATGATATCTCTTTAGCTGTTGATATACTTGGAGATATGTTAAGTAATTCTTTA

>B488_05040|547388..547487|Liberibacter_crescens_BT-1_EZ-Tn5_Transposon_Disruption

GGTTTCAGGAGACGAGGAAGGCTCTCCTGTTTCCCTGAATGTGACTGTAAGGTTTGTTGATTGATCGGGATTGATATTCCGAAGGTTTTTCGTCCCCTGA

>B488_01000|116614..116713|Liberibacter_crescens_BT-1_EZ-Tn5_Transposon_Disruption

ATATACTTCTAAAGAAGTTACCGCTTATACTATTAACATTTTAAAAGATGATATCTCTTTAGCTGTTGATATACTTGGAGATATGTTAAGTAATTCATTA

>B488_06040|667468..667565|Liberibacter_crescens_BT-1_EZ-Tn5_Transposon_Disruption

GCGTTTATGCAATAAAGAAAGTGGTTTCTATTCCTGTTACAATTAAATGTCGTATAGGTATAGATGATCAAGTTCCTGATCTTGCGTTGCGTAATTTAAT

>Intergenic_Region|330816..330915|Liberibacter_crescens_BT-1_EZ-Tn5_Transposon_Disruption

GAATGGATAACCGCTGAAAGCATCTAAGCGGGAAACCAACCTGAAGACGAGTATTCCCTATCAGAGCCGTGGAAGACTACCACGTTGATAGGCTGGGTGT

>B488_08600|942946..942992|Liberibacter_crescens_BT-1_EZ-Tn5_Transposon_Disruption

CTTTTTCTTTTCTTCTGGTAATTGAGGTAAACTATTATAATACAAGGGGTGCTATGAGCCATAGCCGGTGGGTGCGGGGGGGGTCAAAGGTCTCATGCAA

>Intergenic_Region|330816..330915|Liberibacter_crescens_BT-1_EZ-Tn5_Transposon_Disruption

GAATGGATAACCGCTGAAAGCATCTAAGCGGGAAACCAACCTGAAAACGAGTATTCCCTATCAGAGCCGTGGAAGACTACCACGTTGATAGGCTGGGTGT

>Intergenic_Region|330953..330854|Liberibacter_crescens_BT-1_EZ-Tn5_Transposon_Disruption

TATTAGTACTGGTAAGCTTCACTGGTTACCCAACTTCCACACCCAGCCTATCAACGTGGTAGTCTTCCACGGCTCTGATAGGGAATACTCGTTTTCAGGT

>B488_02050|240541..240455|Liberibacter_crescens_BT-1_EZ-Tn5_Transposon_Disruption

GTATATTCACAGTTAGAATATCACCAACATTAATAGCGCGTGCATCTTTCAACAAAGCAGCTCCGAGAATCTCTCCATAAAGAATAACCACCGCCCTATG

>B488_12300|1345722..1345662|Liberibacter_crescens_BT-1_EZ-Tn5_Transposon_Disruption

ATTGAAAACCAAAGAATTCTGCCTCCTCCAAATATCATTCGATTGGATGATGATGATCCTTCAACTCAGCAAAAGTTCCAGGCAATAATATGCTTTGGTG

>B488_06720|744571..744472|Liberibacter_crescens_BT-1_EZ-Tn5_Transposon_Disruption

CTTGACAATAGGAAAAACATTCCCGCCGACACGGATGCCTGTTGGCTGAAATTTAGAGTCTACAAAATAAGATGATTTGAGGCTTTTTAAACGTGAAGAA

>Intergenic_Region|330816..330915|Liberibacter_crescens_BT-1_EZ-Tn5_Transposon_Disruption

GAATGGATAACCGCTGAAAGCATCTAAGCGGGAAACCAACCTGAAAACGAGTATTCCCTATCAGAGCCGTGGAAGACTACCACGTTGATAGGCTGGGTGT

>B488_08600|942781..942880|Liberibacter_crescens_BT-1_EZ-Tn5_Transposon_Disruption

ATTCTGAGCCAAAACATTCCCTCGATTAGCGGCAATAGAAAGCCATTCAAAGCCTTTCTTATAATCACGCTCACCTCCAACACCATTAATAAACCAAAGA

>B488_08600|942781..942880|Liberibacter_crescens_BT-1_EZ-Tn5_Transposon_Disruption

ATTCTGAGCCAAAACATTCCCTCGATTAGCGGCAATAGAAAGCCATTCAAAGCCTTTCTTATAATCACGCTCACCTCCAATACCATTAATAAACCAAAGA

>B488_01000|116614..116713|Liberibacter_crescens_BT-1_EZ-Tn5_Transposon_Disruption

ATATACTTCTAAAGAAGTTACCGCTTATACTATTAACATTTTAAAAGATGATATCTCTTTAGCTGTTGATATACTTGGAGATATGTTAAGTAATTCTTTA

>B488_02820|321407..321506|Liberibacter_crescens_BT-1_EZ-Tn5_Transposon_Disruption

GGTTTATATTTCTCAAAATTTAGTAACTCTTTTTTCCAAAGCGGAAGAAATTTCTAAAAAAGCTGGAGATAGTTTTGTAACTGTTGAGAGGCTCTTGCTG

>Intergenic_Region|249988..250087|Liberibacter_crescens_BT-1_EZ-Tn5_Transposon_Disruption

CATCAGAGTTGAGGGCGTGGAGTTGTTTATTTTATAATATTGTACTGTAAGAGAGGGAGTATGATTGCTTTTTTATGAAGGAGTGGTCGGAGCGACTGGA

>B488_07570|842770..842672|Liberibacter_crescens_BT-1_EZ-Tn5_Transposon_Disruption

TCATAGAGCTTACTATAAAAATAATTGCTGATACACCGGCAATTACAACCAAAATAAAAGATGTTTTTTTATTTTTCTGGAGCATTGAGGGATATCCTTC

>B488_10170|1112867..1112775|Liberibacter_crescens_BT-1_EZ-Tn5_Transposon_Disruption

ACTATGCACCAGTTATAAAAGTACGTAGTCATTGTTAGTACAATCGTTGGAGCAGGAGCGCTAGTTTATAAAAAAGCTAAAATAGACGCCTATAAAACAG

>B488_05120|553906..553960|Liberibacter_crescens_BT-1_EZ-Tn5_Transposon_Disruption

GATCAGGGACGAAAGGAATTGTGTTCTTGTCTGTCTTCCCCTTGATCCTTCAACTCAGCAAAAGTTCAGGGTTGTGACGAGATTGTTTGGTGAAAATTCT

>B488_07900|866605..866704|Liberibacter_crescens_BT-1_EZ-Tn5_Transposon_Disruption

ACCTATAACCCTTCTTCTGATCAACCGCTGAATCATTGTGATGAGAAGGACAAAAAACCATTTTGTCCTCTTCATTGATCGATTTCTTTATTCTAGCTTC

>B488_03090|355169..355070|Liberibacter_crescens_BT-1_EZ-Tn5_Transposon_Disruption

GAACAGGGCAAAGCCATTTTTTATATAAAGGTGAATATGACACAATGCTTTTGATAGGCTTTGGTTTTATCACGGGTACTTTGCTGAGTCTCTTTGCTAA

>Intergenic_Region|541905..541807|Liberibacter_crescens_BT-1_EZ-Tn5_Transposon_Disruption

GCATAAGACTGTGCGGAATAGTGTTTGGCTTAGGAAGTCTTGCTACTCGGCTAATAAAATCTGATCTTAAATTCTGCCATGAAATGACTACCATAAATAG

>B488_06780|752056..752155|Liberibacter_crescens_BT-1_EZ-Tn5_Transposon_Disruption

GGGTCTTGAGTGTTTCGAAAAGCTGATGGGGCAATTGTCCATCTCGCGCAATGAATACCAATTTATTCAGCTTCGCTTCTTTTGTTCTCGCCATCACCCA

>Intergenic_Region|249996..249897|Liberibacter_crescens_BT-1_EZ-Tn5_Transposon_Disruption

ACTCTGATGATAGTTTTACTATGATGTTTTGAATTTTCCTAGTTTTTAAGATTAATGAGTACTATTATAATATGTTATCTTTCACTTTATGTGTTTACTT

>Intergenic_Region|329143..329044|Liberibacter_crescens_BT-1_EZ-Tn5_Transposon_Disruption

CTAGTACTCCTCGGTATTCGGAGTTTGGTTAGGAGCAGTAAGGCGGTGAGCCCCCATAGCCTATCCAGTGCTCTACCCCCGAGGGTATTCAGTTAACGCT

>B488_02330|267666..267589|Liberibacter_crescens_BT-1_EZ-Tn5_Transposon_Disruption

GGATTAATATATGGCTCTAAAGCACGTAATGTAATTTTAAGAGCACCCTGACCCTGCATTTCTTAATCCTTATTCATACTGTTGTTTATCCCATTATCCC

>B488_13390|1456112..1456013|Liberibacter_crescens_BT-1_EZ-Tn5_Transposon_Disruption

GCTCATGATGGTCCTCGGGATGTTGTATTTCAATTGCAGGAATCACTGATTGGCACCTATAGATATAGAGTAAACGAAAAAGTCCAAATTTCTTTAATAG

>B488_10170|1112871..1112770|Liberibacter_crescens_BT-1_EZ-Tn5_Transposon_Disruption

AATAGCAGCAGTTATAAAAGTAGTAGTCATTGTTAGTACATCGTTGGAGCGGGAGCGCTGTTTATAAAAAGCTAAAATAGACGCCTAAAAAACAGCCTAT

>B488_12300|1345722..1345662|Liberibacter_crescens_BT-1_EZ-Tn5_Transposon_Disruption

ATTGAAAACCAAAGAATTCTGCCTCCTCCAAATATCATTCGATTGGATGATGATGATCCTTCAACTCAGCAAAAGTTCCTGGTTTGTTTCTTTTGGAGTT

>B488_05060|550653..550554|Liberibacter_crescens_BT-1_EZ-Tn5_Transposon_Disruption

TTGTTCTTCAGGGCTGTTTCTGTTGCAAATTTCTGCTGCTCAAAAGCCAACTTCTGCTGACTGTCTGCTGCCCTCTGCTGGCGATCCAGTGCTTTTTCGT

>Intergenic_Region|500685..500586|Liberibacter_crescens_BT-1_EZ-Tn5_Transposon_Disruption

TTACAATTTAGATCAAATATCTTGATAATATAAAATATAATTGCATTATAAGAAAATACAATTTTAACCATTAAAATTATTTGGGAAAAGATAAATGAAA

>B488_13110|1422374..1422473|Liberibacter_crescens_BT-1_EZ-Tn5_Transposon_Disruption

AGTGGGCGTTGACCGTGAGCGTTGCCGGCACTTGACTCCCGACCAGAGAATGATAGCGCGCCACCGGCAGCGGGTTCGGCAGGCCGGCGAACATGCCCTG

>B488_13420|1459557..1459642|Liberibacter_crescens_BT-1_EZ-Tn5_Transposon_Disruption

CTATAACACCAGTCAAGTCTACGAAATTGTCAAAGATTATAATCTTGCAGAAAAGCAGCTTCGGCTAGCAATTTCATATGATCCTTCAACTCAGCAAAAG

>B488_10960|1195075..1194976|Liberibacter_crescens_BT-1_EZ-Tn5_Transposon_Disruption

GGTGAAGAGAGTGTCTTCTTTTACAAAAGCAACTAAACGACCATTATCAGATATTAAAAGATCAGGTTTAGGAGTTGGATGAAGAAAATGAATGCGCTCG

>B488_08620|943848..943749|Liberibacter_crescens_BT-1_EZ-Tn5_Transposon_Disruption

GTGCAACAAGTTGATTAAGTTTTAAAAGAAAAAAGGAGAATTTTTCTTGGATGAGCATGTTTTGATTATATTTTGTGATATGTTGTAGTACATGGAGCAA

>Intergenic_Region|330816..330915|Liberibacter_crescens_BT-1_EZ-Tn5_Transposon_Disruption

GAATGGATAACCGCTGAAAGCATCTAAGCGGGAAACCAACCTGAAAACGAGTATTCCCTATCAGAGCCGTGGAAGACTACCACGTTGATAGGCTGGGTGT

>B488_10170|1112871..1112772|Liberibacter_crescens_BT-1_EZ-Tn5_Transposon_Disruption

AATAGCAGCAGTTATAAAAGTAGTAGTCATTGTTAGTACAATCGTTGGAGCAGGAGCGCTGTTTATAAAAAAGCTAAAATAGACGCCTATAAAACAGCAT

>B488_05460|589784..589685|Liberibacter_crescens_BT-1_EZ-Tn5_Transposon_Disruption

TTATAGAACAGATCGCGGTACCTGATTCACAAGGAGTTTTGCTTCTCAACGAGGCTGCTGAAAAAATGCATTTTTCAGCACGCGGTTATCATCGAATTCT

>B488_13420|1459557..1459642|Liberibacter_crescens_BT-1_EZ-Tn5_Transposon_Disruption

CTATAACACCAGTCAAGTCTACGAAATTCGTCAAAGATTATAATCTTCGCAGAAAAGCAGCTTCGGCTAGCAATTTCATATGATCCTTCAACTCAGCAAA

>B488_07050|783385..783286|Liberibacter_crescens_BT-1_EZ-Tn5_Transposon_Disruption

TCCTTGCGCCCCTTGTTGAACGGCAGGAAGAAGGTGTTTTCCCCGTCCAGCTTGGTTGCCATCATGATTTCGGAATCCGACATAGCGAAATGCACCACCG

>B488_08600|942946..942992|Liberibacter_crescens_BT-1_EZ-Tn5_Transposon_Disruption

CTTTTTCTTTTCTTCTGGTAATTGAGGTAAACTACTATAATACAAGGGGTGCTATGAGCCATAGCCTGTGGCACCATGAGACGCTTAATCCTCAACTGAT

>Intergenic_Region|501749..501649|Liberibacter_crescens_BT-1_EZ-Tn5_Transposon_Disruption

GTGCTGATGGCTGGTGTATAGTAAAAAGTATCTTCCATCATAGAACGCCAATTCCTAACTTAAACAATAAAAATGACTTTTAAAAAAAAAATATCTAAAA

>Intergenic_Region|501741..501840|Liberibacter_crescens_BT-1_EZ-Tn5_Transposon_Disruption

CATCAGCACATATTAACATCATTGATTGATGTTCTACAAACAATAGTCTTAAACATAACGATATCGCTTTTACAAAGAAAGAGATATTTATTAAGTTATA

>B488_11600|1260128..1260029|Liberibacter_crescens_BT-1_EZ-Tn5_Transposon_Disruption

GATTATTATTGACCAATCTTTGACACCTGTGCAGCAGCGTAATTTAGAAAAAATGTGGAACATTAAAGTAATTGACCGTATTGGTTTGATTTTAGAAATT

>B488_13390|1456112..1456013|Liberibacter_crescens_BT-1_EZ-Tn5_Transposon_Disruption

GCTCATGACGGTCCTCGGGATGTTGTATTTCAATTGCAGGAATCACTGATTGGCACCTATACACATACAGTAAACAAAAAAGTCCAAATTTCTTTAATAG

>B488_10170|1112871..1112772|Liberibacter_crescens_BT-1_EZ-Tn5_Transposon_Disruption

AATAGCAGCAGTTATAAAAGTAGTAGTCATTGTTGGTACAATCGTTGGAGCAGGAGCGCTGTTTATAAAAAAGCTAAAATAGACGCCTATAAAACAGCAT

>Intergenic_Region|470212..470113|Liberibacter_crescens_BT-1_EZ-Tn5_Transposon_Disruption

TCACTACACTGTTCCAGGAAACAATAAAAAGCAATAATCGCATGTGATACGCTCCAAAAACAATAAGAAAAGTCTCCATATTTTAATTACAAAATTGAAA

>Intergenic_Region|470204..470303|Liberibacter_crescens_BT-1_EZ-Tn5_Transposon_Disruption

GTGTAGTGATTTATAAAAACAGCTATTAAATATAATTCTCAGTCAAACCTAACGTTTTCCTTGTATTTTTTTTACAAAGCTTGATTCTTGATTGGCAGTA

>Intergenic_Region|330816..330915|Liberibacter_crescens_BT-1_EZ-Tn5_Transposon_Disruption

GAATGGATAACCGCTGAAAGCATCTAAGCGGGAAACCAACCTGAAAACGAGTATTCCCAATCAGAGCCGTGGAAGACTACCACGTTGATAGGCTGGGTGT

>B488_05550|594001..594101|Liberibacter_crescens_BT-1_EZ-Tn5_Transposon_Disruption

GTACTTGTCCCAACCGTATAAAAGCGAATGCCCTCTGTATCGCCACAGATCCTGCTCTGCCTCTCCTGAGATCGGCACCAGATCTCTTCGGACTCCCCGT

>B488_09500|1021783..1021684|Liberibacter_crescens_BT-1_EZ-Tn5_Transposon_Disruption

GCTTAAAGCACTTGTTAATTTATCTGATCACTCTTCTTCTACTATACGTCTTTATGAAGAGCTTGTTAAATTACCTTTCTCAGATATACGTTCTATTGAA

>Intergenic_Region|330945..331044|Liberibacter_crescens_BT-1_EZ-Tn5_Transposon_Disruption

GTACTAATAGCTCGATTGGCTTGATCCTTCAACTCAGCAAAAGTTCCCTTTTTTGCCCTTTATAGACCTGGTGGCTTTTGCGGAGTGACTGCACCCGTTC

>Intergenic_Region|330816..330912|Liberibacter_crescens_BT-1_EZ-Tn5_Transposon_Disruption

GAATGGATAACCGCTGAAAGCATCTAAGCGGGAAACCAACCCGAAAACGAGTATTCCCTATCAGAGCCGTGGAAGACTACCACGTTGATAGGCTGGGCGT

>B488_04700|526803..526902|Liberibacter_crescens_BT-1_EZ-Tn5_Transposon_Disruption

CCTTATAGCCATCTGGAGCAATTCCACAGGCGTCAATTCTCTTGTTATTTCGTGTTCTTCTGTTGTCCTGATTGCAGAAGTTCCTGCTGGCATAATTTCC

>B488_07010|777877..777778|Liberibacter_crescens_BT-1_EZ-Tn5_Transposon_Disruption

GTGCACTGGAACATTCAGTAGAGCGTGTTTTTTCAATTTCGGTGATGAAAGCAGGTTTTTGTATTCATGATGTTGATGCTGTTGATGAAGCAAATCATTG

>B488_08600|942781..942881|Liberibacter_crescens_BT-1_EZ-Tn5_Transposon_Disruption

ATTCTGAGCAAAACATTCCCTCGATTAGCGGCAATAGAAAGCCATTCAAAGCCTTTCTTATAATCACGCTCACCTCCAATACCATTAATAAACCAAAGAC

>B488_08600|942781..942880|Liberibacter_crescens_BT-1_EZ-Tn5_Transposon_Disruption

ATTCTGAGCCAAAACATTCCCTCGATTAGCGGCAATAGAAAGCCATTCAAAGCCTTTCTTATAATCACGCTCACCTCCAATACCATTAATAAACCAAAGA

>B488_13390|1456112..1456013|Liberibacter_crescens_BT-1_EZ-Tn5_Transposon_Disruption

GCTCATGATGGTCTCCGGGATGTTGTATTTCAATTGCAGGAATCACTGATTGGCACCTATAGACATAGAGTAAACGAAAAAGTCCAAATTTCTTTAATAG

>B488_10170|1112871..1112772|Liberibacter_crescens_BT-1_EZ-Tn5_Transposon_Disruption

AATAGCAGCAGTTATAAAAGTAGTAGTCATTGTTAGTACAATCGTTGGAGCAGGAGCGCTGTCCATAAAAAAGCTAAAATAGACGCCTATAAAACAGCAT

>B488_02300|265010..265109|Liberibacter_crescens_BT-1_EZ-Tn5_Transposon_Disruption

AGATAGAGCTTGGTGTATTAGTATTGCAGTTGTTCATCAGGGTCGTCCTGTTGCTGGTGTAATAAATGCATCAGCTCTTGGGCAGGAATTTTATGTTTGT

>B488_06270|691052..691151|Liberibacter_crescens_BT-1_EZ-Tn5_Transposon_Disruption

CGCCAAGAGTGACTGGTAGGGATTGCATCACTGCGTAAGGTTTAAATCCAATGAAAGCCTTTTGCCATGATTCCAGCCAGACCAAGGGCGACAGCGATGA

>B488_07950|871610..871709|Liberibacter_crescens_BT-1_EZ-Tn5_Transposon_Disruption

GTATCGCCATGGCAGCCAGTGGTGCCCCGCTTGGAGTTTTTCTGGTCCTGCGTCGTATGTCTCTTATGGGAGAAGCAACCTCACATGCTATTTTACCGGG

>B488_07590|843561..843462|Liberibacter_crescens_BT-1_EZ-Tn5_Transposon_Disruption

GTATTGTATTATGTTGAAGTATTCTGGATAATTCTTCCTTGAGTAATTCAAGGGGTGTTATGAGCCATAAAAGTTGTTTTTTGAGTTGAATACTTGTTGA

>B488_04830|535315..535216|Liberibacter_crescens_BT-1_EZ-Tn5_Transposon_Disruption

CTTTAAACATGCAAAACTTGAGAGGTATATTGATAAATACATAGTAGATATTAGAAGAGCTCTATTTTCTAAACCGGAAGAGAGAGAGAGTATTCTCGAT

>B488_11210|1221582..1221679|Liberibacter_crescens_BT-1_EZ-Tn5_Transposon_Disruption

TGATACTATTGGTAATACAAAGGATGATCAGACCCATATGCATAAACATAATTATCAACGTGTTCTTGTGGTTACAAACAATTATCATATGTTACGCACC

>B488_00590|73234..73135|Liberibacter_crescens_BT-1_EZ-Tn5_Transposon_Disruption

GTGATACAATGCATTCATTGCACCAGAACGCTCTAATGCCCATAAATCCGCAACATCTTCAATTACAATAATAACTGAATGATCACGTTTTTTATCTGTA

>B488_13370|1451775..1451676|Liberibacter_crescens_BT-1_EZ-Tn5_Transposon_Disruption

TTCTTGTAGTTCAGATTGAAAAACTTCGTTCTCAGGGAGTAAATCCCTGGGTAGCTGTACGTGAAGCTGTAGAACACCGTGTGAGGCCAATTTTTCTAAC

>B488_10170|1112871..1112773|Liberibacter_crescens_BT-1_EZ-Tn5_Transposon_Disruption

AATAGCAGCAGTTATAAAAGTAGTAGTCATTGTTAGTACAATCGTTGGAGCAGGAGCGCTCGTTTATAAAAAAGCTAAAATAGACGCCTATAAAACAGCA

>B488_09990|1093563..1093464|Liberibacter_crescens_BT-1_EZ-Tn5_Transposon_Disruption

CATATGGGTTGCCGGTACTTCTCTCTAACTGTTCTAATAATTATGGTCCCTATCAATTCCCTGAAAAGCTTATTCCTCTAACAATTCTTAATGCCATAAA

>B488_10260|1123431..1123530|Liberibacter_crescens_BT-1_EZ-Tn5_Transposon_Disruption

CTCTTTAAGTCTCGGGAAAAGCTGAAATACATTCATTAAGTCATCATTAAAATATTTTAATTTATCAATGACGGCTCCCATTTGCAGATTTTCATAAACC

>B488_05180|561018..560919|Liberibacter_crescens_BT-1_EZ-Tn5_Transposon_Disruption

ATTCAGTGTTGCAGCATTTCCTCCCAAATCCTGTATATGACCAAGGGCTACTTTTGCCCCTGTTTGCAACTCAGGATGATAGCCAAAATTCCTGAATTCA

>B488_13430|1461169..1461070|Liberibacter_crescens_BT-1_EZ-Tn5_Transposon_Disruption

GGTCATGTATGCTTGCACAGAAACGGCAAAAACTTGCCCTGTTCCCAACATGAGGCTGCCGCTCGCAACCGCTAGTATAAACCCATAATCTAAGGACATA

>B488_10170|1112864..1112772|Liberibacter_crescens_BT-1_EZ-Tn5_Transposon_Disruption

AATCATCCGCATTTATAAACATAGTAGTCATTGTTAGTACGTCGTTGGAGCAGGGGCGCGGTTCCTAAAAAAGCTAAAATAGACGCCTATAAAACAGCAT

>B488_12820|1397304..1397403|Liberibacter_crescens_BT-1_EZ-Tn5_Transposon_Disruption

GCGTTTATCTCATTAATCATTTTACCTGAATTAGCTGCCGCTCCAAAAAGGGCAATATCAAAGGGAATTACCGCAGAAGGCCTCACTCCTAGTGGTTCAC

>B488_02280|262634..262733|Liberibacter_crescens_BT-1_EZ-Tn5_Transposon_Disruption

TGTTTATCCTGAAGCGTTAAATGCGACATATATGGTTGATATGTTTGCTGGTGTAGGTTCTATAGGACTTGAGGCGTTATCTCGAGGTTGTCGTTACGTT

>B488_10910|1186885..1186786|Liberibacter_crescens_BT-1_EZ-Tn5_Transposon_Disruption

CCTCTATTCATAAGGTTGGAGGTCTTGATGTTAATAATTTTATTGAAATCAATAAATTATTACAGCGCCTTGATCGTTTCTGGAATGATCAAATTTTATA

>B488_11180|1217946..1217847|Liberibacter_crescens_BT-1_EZ-Tn5_Transposon_Disruption

CCTACACCTGGTACGAACAACTATCTGGCCATGAAGGTCGCTTATGATAAGCTGACGTCGGCCTATGAAGCATCAGAACAGAAAAAGAAGAATAACAAGA

>B488_03390|390976..391075|Liberibacter_crescens_BT-1_EZ-Tn5_Transposon_Disruption

GACAGGAACACAATACGCTATAAGTCAATTGCCGGAAAATATCACTCTTGCCTTTGCGTCTGGTGGGAATAGCCTGCAACGTTGGGGACAAGAAGCAAGA

>B488_08210|893450..893351|Liberibacter_crescens_BT-1_EZ-Tn5_Transposon_Disruption

CTTGAATGTTTGTTCTACGGATTGTATCTCTTTGCCATTTTTTTCTTGTCCGTAGGCATTGATGTTAAAAAAATCTGTAACCTGAGAAACACCAGAGCCT

>B488_04940|543102..543003|Liberibacter_crescens_BT-1_EZ-Tn5_Transposon_Disruption

TTGTTTCAGATTCTGAAACAGATTTGCTTAATGAAGATACCTCTTCTTTTAAGAACTCAATATTAAATTTACTATTATTTAATGTCTTTAATACTTCTTC

>B488_11040|1203332..1203233|Liberibacter_crescens_BT-1_EZ-Tn5_Transposon_Disruption

GAGTAGCACGTATAGCTGCAACATATCCAGCAGGACCTGATCCAATGATAGCAATATCATAAGTATTTGACATAAGCGCGTCCATTTTTGGGAGTGTTCT

>B488_10960|1194776..1194678|Liberibacter_crescens_BT-1_EZ-Tn5_Transposon_Disruption

GATCTAAAGAGAGAGGTATTAAAAATATTGTAGCAAAAGCTGCAGGGATAACTATGAATGATAGTAACGGTATGGCAGCGAGATTCGGCAAGAAATCCAT

>B488_03250|375240..375141|Liberibacter_crescens_BT-1_EZ-Tn5_Transposon_Disruption

AGAAGGGTCACTCCCAAAAAAAACAGACGAGTTAATATTCCGCAGTTCAGTTTCTACCCCTATCTTCTTCCACATATCTTTTATAAGTGATTGAGTCCCT

>B488_01340|164820..164721|Liberibacter_crescens_BT-1_EZ-Tn5_Transposon_Disruption

CAATAATATCAATCAAATAATGACTGGCATTAGCAACGGCGGAAACCATCATTAGAAAATTTAGTAAAAGAGAAACCCACTTTAAAAATTTAAGAGGCCA

>B488_06930|770947..771046|Liberibacter_crescens_BT-1_EZ-Tn5_Transposon_Disruption

GGCAGAAGGTGGGACTTGACAACTTGAACGAATGATTTCCACTTCATCCCCGTTATTAAGCAGGGTAAAAAGAGGCATTATTCTACCATTTATTTTGGCG

>B488_13030|1414828..1414927|Liberibacter_crescens_BT-1_EZ-Tn5_Transposon_Disruption

GTTTTATTCTTCGCCAAAACACCGCCACATATAGCACGTGTTACAATCTCTGGAACCATAGAAGATAACCCTGAATTATTGAGAGTTCTTAAAAAAGTTG

>B488_07930|869686..869785|Liberibacter_crescens_BT-1_EZ-Tn5_Transposon_Disruption

ACATCAGGGAGCCCATCGAACCGGAAACATCCAGGACCAGTTCAATGGAGACGGGAATGCTTTCTTCCTGTTGTGCTTCCGCAACGCCATTCACCGCAAT

>B488_01440|179009..179066|Liberibacter_crescens_BT-1_EZ-Tn5_Transposon_Disruption

AGTACTTACTGTAATACAAGGGGTGTTATGATCCATAATTATATGAAAGTGGATATCTTGCTAATGAACATCAAAAAATGAACCTTGCTGCTATCGAGGC

>B488_00700|84567..84665|Liberibacter_crescens_BT-1_EZ-Tn5_Transposon_Disruption

AGTACTTACTGTAACGAAAGGAGCCGCTTCGGTTGTTTTTTCAATTAGGTTCGGCTCTTTCTTCAGAGACTCTTGTGCAAGGAATTGAAAATCTTTATCT

>B488_10170|1112871..1112771|Liberibacter_crescens_BT-1_EZ-Tn5_Transposon_Disruption

AATAGCAGCAGTTATAAAAGTATATCATTGTTAGTCCAATCGTTGGACAGGAGCGCCGTTATAAAAAAGCTAAAATAGACGCCTATAAAACAGCATACAC

>B488_12300|1345722..1345662|Liberibacter_crescens_BT-1_EZ-Tn5_Transposon_Disruption

ATTGAAAACCAAAGAATTCTGCCTCCTCCAAATATCATTCGATCGGATGATGACGATCCTTCAACTCAGCAAAAGTTCAGAACTCAAAGTTCCAGAAAGC

>B488_07280|812021..811922|Liberibacter_crescens_BT-1_EZ-Tn5_Transposon_Disruption

TTGTTGTTCTTGTTATCTTTATCGAAATCTATCGTAAAATCTGCTTTTAACAGAACACATTGCTCTTTTTGGCAGAAGTTAACTTCAACATGTAAATTGA

>B488_01440|178968..179064|Liberibacter_crescens_BT-1_EZ-Tn5_Transposon_Disruption

CTTCGGGTTAGCATCTTCTTTATCTGTAATTCGTGTTAGGAGATGAAAGTGGATATCTTAGCTAATGAACATCAAAAAATGAAGCTTCGCTGCTATTGAG

>B488_02720|308485..308584|Liberibacter_crescens_BT-1_EZ-Tn5_Transposon_Disruption

GATATTAGTTTTCTTATCAATGAAAATCAGATCCTTAAAGCAATTATTATTACACATGCACATGAAGATCATTTTGGGGCTATTATTGATTTATGGTCTA

>B488_05410|583659..583754|Liberibacter_crescens_BT-1_EZ-Tn5_Transposon_Disruption

AGCCCCTCCTTGAGTGAGATCTAAGCTCTTATGCGACCAGTGCATCATCATGGAACATATGATGATGCAGGTCTGATGATGTATGCAGAAGATCCTCCTG

>B488_09250|998098..998195|Liberibacter_crescens_BT-1_EZ-Tn5_Transposon_Disruption

GCTCAATTGATGGCTTATTATCCTCTCTTCTCCTTTGTTTCCATATTATTCTTTATTTCTTCCTGGATGGGTAACTCGTTAACGCATTATACCACCAAAA

>B488_13030|1414828..1414927|Liberibacter_crescens_BT-1_EZ-Tn5_Transposon_Disruption

GTTTTATTCTTCGCCAAAACACCGCCACATATAGCACGTGTTACAATCTCTGGAACCATAGAAGATAACCCTGAATTATTGAGAGTTCTTAAAAAAGTTG

>B488_01420|175218..175119|Liberibacter_crescens_BT-1_EZ-Tn5_Transposon_Disruption

CTAAAAACACTTGCAACTTTTCCTGAAGAAGGACAATTAATATCGAGAGGAGAAATATTTCTCCATCGCTCTAGCATTTCCTGTCTTTTTTGTGACACAA

>B488_12250|1339138..1339234|Liberibacter_crescens_BT-1_EZ-Tn5_Transposon_Disruption

TGCACAGGGAGTCCAGACTACCCCGATTAACAAACCGATTAAAATACCGCTAAAAAAACCGTCGTTAGCATTTCTGAATAATCGATGTCCTACATTACAG

>B488_05630|612185..612284|Liberibacter_crescens_BT-1_EZ-Tn5_Transposon_Disruption

CTATCAGGCTATCTTTTCTGTTGATAATTCTGCTCTTTTATTACGTCCTGGGATGACGGCAACAGCGGATATTACCATTAATAAAGTACAGAATAAGTTG

>B488_10170|1112871..1112772|Liberibacter_crescens_BT-1_EZ-Tn5_Transposon_Disruption

AATAGCAGCAGTTATAAAAGTAGTAGTCATTGTTAGTACAATCGTTGGAGCAGGAGCGCTGTTTATAAAAAAGCTAAAATAGACGCCTATAAAACAGCAT

>B488_12840|1400230..1400131|Liberibacter_crescens_BT-1_EZ-Tn5_Transposon_Disruption

ATCTTTATCTTTACAAGAAGACAGACGGGTATCAACGGTTGTTAATTTGTTGGATATTGAGGGTGAGGATCAGGTTACTCTTAAGATTACTGTTGCAGAA

>B488_11650|1268715..1268814|Liberibacter_crescens_BT-1_EZ-Tn5_Transposon_Disruption

CTTCTTCGCTAAGTATCTCAAGAACGATAAGTCCCAAGTCTATGGGACAAAAAGTAAGCGTTTTGAAACCAAGCATTGGTTGATCTCCCCCTTTAATCTC

>B488_10170|1112848..1112773|Liberibacter_crescens_BT-1_EZ-Tn5_Transposon_Disruption

ACTTACACCATGAATTCAAATCATAGTCAATGTTCCTACTATCGTTGGAGCAGGAGCGCTCGTTCATAAAAAAGCTAAAATAGACGCCTATAAAACAGCA

>B488_08420|920399..920494|Liberibacter_crescens_BT-1_EZ-Tn5_Transposon_Disruption

ATATAAAACTGTTATAATATAAGGCACATTTATCTCTCTACGTTGATTAAAATGGATCATTTAATCATGATTGATCAAGGTCGTGTTATTGAACAAGGCT

>B488_10170|1112871..1112772|Liberibacter_crescens_BT-1_EZ-Tn5_Transposon_Disruption

AATAGCAGCAGTTATAAAAGTAGTAGTCATTGTTAGTACAATCGTTGGAGCAGGAGCGCTGTTTATAAAAAAGCTAAAATAGACGCCTATAAAACAGCAT

>B488_05910|650337..650436|Liberibacter_crescens_BT-1_EZ-Tn5_Transposon_Disruption

GTTCCATGGTGTTACTTTGGGTGGTACAGGCAAGGAGCGAGGAGATCGTCATCCAAAGGTTCGTAAAGGAGTTATCATTGGCGCAGCTGCAATAATCCTT

>B488_10960|1194776..1194677|Liberibacter_crescens_BT-1_EZ-Tn5_Transposon_Disruption

GATCTAAAGAGAGAGGTATTAAAAATATTGTAGCAAAAGCTGCAGGGTTAACTATGAATGATAGTAACGGTATGGCAACGAGATTGGCAAGAAATCCATA

>B488_10170|1112871..1112772|Liberibacter_crescens_BT-1_EZ-Tn5_Transposon_Disruption

AATAGCAGCAGTTATAAAAGTAGTAGTCATTGTTAGTACAATCGTTGGAGCAGGAGCGCTGTTTATAAAAAAGCTAAAATAGACGCCTATAAAACAGCAT

>Intergenic_Region|1334379..1334280|Liberibacter_crescens_BT-1_EZ-Tn5_Transposon_Disruption

GATCTAAACAACTCATAATGGGTGACGCATAGAAATATTTTATTTAGGCACCATAAACAAATTTTTGTGATAAGCATAAAAAATTTTTAAAATTAATTCT

>B488_10170|1112871..1112772|Liberibacter_crescens_BT-1_EZ-Tn5_Transposon_Disruption

AATAGCAGCAGTTATAAAAGTAGTAGTCATTGTTAGTACAATCGTTGGAGCAGGAGCGCTGTTTATAAAAAAGCTAAAATAGACGCCTATAAAACAGCAT

>B488_08140|884204..884105|Liberibacter_crescens_BT-1_EZ-Tn5_Transposon_Disruption

CTCCAATGACGTGCTGGCGTACAATGTAGTTTCAATCCCATCATTGACACCTCATCCCACCAGTCGCATTCACTCACAGAACCAGCACCGTATTGACGTA

>B488_13160|1429898..1429985|Liberibacter_crescens_BT-1_EZ-Tn5_Transposon_Disruption

AAGCAATACATCATCTTCAAGAATAAGAGCGGCTTCATGTCCATCAGAAACAAATTTCTCTAAGGCTGCAACATGACTATAATACAAGGGGTGTTATGAG

>B488_11180|1217946..1217845|Liberibacter_crescens_BT-1_EZ-Tn5_Transposon_Disruption

CCTACACCTGGTCGAACACTATCTGGCCATGAAGGTCGCTTATGATAAGCTGACGTCGGCCTATGAAGCATCAGAACAGAAAAAGAAGAATAACAAGATC

>Intergenic_Region|1236364..1236265|Liberibacter_crescens_BT-1_EZ-Tn5_Transposon_Disruption

CCTTAAGCTATTAATTCAGATCAAAAAAGATTAATCCTTTAAAAAAGGACTATTATACAGTATATATTTTCTATCAAGGGTATCCTAAGTTTACATATCA

>B488_05380|580765..580666|Liberibacter_crescens_BT-1_EZ-Tn5_Transposon_Disruption

GACATGAAATTCCCGCAGACAAAGATAAAAGGAATCACAAATGAATAAATTTTATAGACTGTTGGATCTCTTTTCTCATATTCTGCTTATCTTTTATATA

>B488_01600|194010..194111|Liberibacter_crescens_BT-1_EZ-Tn5_Transposon_Disruption

CTCTTGGTATTGATAGAATAGGATCCTTGACATATGCTTCCACCTGAGATAACATTAGGTATTAGTACTACAGCTCGAGTAAGAAATTTTCGGCTTATTG

>B488_13420|1459746..1459850|Liberibacter_crescens_BT-1_EZ-Tn5_Transposon_Disruption

CCCTTACTATGAGGCTTACTTAATCGAGGTGTTCTCTTGGCACGCTTTGGTGAAACGATAAGGCATAGGAGACTCACACGCGCATTGGAGAAAAACCACA

>B488_12830|1399059..1399158|Liberibacter_crescens_BT-1_EZ-Tn5_Transposon_Disruption

AGTATGCTCTGTCTCTTTCAGAACAATTTTATGTCTCTTACGATAATCACTGCTATCATCTTCAATTGAACTTTGCCTTGTTGTGGTACAAGAAGACATT

>B488_04020|466446..466347|Liberibacter_crescens_BT-1_EZ-Tn5_Transposon_Disruption

GTGCTGTACGTGTGATTAATCCAACCGTATCTTTGGTATGAGAAATAGATAAAATACACTGATGAGGATAACATCCCTTCGTTGGTTTAAACCCAACAAC

>B488_13510|1471595..1471496|Liberibacter_crescens_BT-1_EZ-Tn5_Transposon_Disruption

GTTATATTCGGTAGCTTGTTCCATATCACCGAGTTTTAAGCGGACACATTTACCATCTTTTATATCAATAGCAGGGAATAAAATCATATGACTACCATTA

>Intergenic_Region|326469..326370|Liberibacter_crescens_BT-1_EZ-Tn5_Transposon_Disruption

TCTTCACTCACGCGGCATGGCTGGATCAGGGTTGCCCCCATTGTCCAATATTCCCCACTGCTGCCTCCCGTAGGAGTCAGGGCCGTGTCTCAGTCCCAGT

>B488_11650|1268715..1268815|Liberibacter_crescens_BT-1_EZ-Tn5_Transposon_Disruption

CTTCTTCGCTAAGTATCTCAGAACGATAAGTTTCAAGTCTATGGGACAAAAAGTAAGCGTTTTGAAACCAAGCATTGGTTGATCTCCCCCTTTAATCTCT

>Intergenic_Region|386738..386640|Liberibacter_crescens_BT-1_EZ-Tn5_Transposon_Disruption

ATATTTGCTTACTTACCTCAAGAATCAAGTTATTGAGCTCGTTAATAAATATTAATTTGAATACCTTTAACAGAAAATATGACTTTTTCTGCATTCAAAA

>B488_10170|1112871..1112772|Liberibacter_crescens_BT-1_EZ-Tn5_Transposon_Disruption

AATAGCAGCAGTTATAAAAGTAGTAGTCATTGTTAGTACAATCGTTGGAGCAGGAGCGCTGTTTATAAAAAAGCTAAAATAGACGCCTATAAAACAGCAT

>B488_05410|583659..583752|Liberibacter_crescens_BT-1_EZ-Tn5_Transposon_Disruption

AGCCCCTCCTTTGAGTTGAGATCTAAGCTCTTATGCGACCAGTGCATCATCATGGAACATATGATGATGCAGGTCTGATGATGTATGCAGAAGATCCTCC

>Intergenic_Region|583664..583566|Liberibacter_crescens_BT-1_EZ-Tn5_Transposon_Disruption

GGAGGGGCTTTCTTATTTAAAGAAAGCCTTCTTTAAGGCTCCATCACTGCACAGCCCCCTTTCAAGGAAAAGCAACTGTCATCACCAGGTATATCCAACA

>B488_05430|587470..587371|Liberibacter_crescens_BT-1_EZ-Tn5_Transposon_Disruption

CTCTTGATACTCCCTCTGATGGTTGATCCTCTAACATTTTCTCTAGCCTTAATGATGTTTTTAGCCTCGCTAATAGTAGGAGTTCTATACGCAATGATAG

>B488_13390|1456498..1456399|Liberibacter_crescens_BT-1_EZ-Tn5_Transposon_Disruption

TTATTGAACGGTTAGTTGATATAGGTTCTCATGTTAACGTTGGAGATGTATTGGCAAGATTAAATTCTGTGCAACAGGTTTCTGAGGTAGCTTCTGCTGA

>B488_11940|1303539..1303638|Liberibacter_crescens_BT-1_EZ-Tn5_Transposon_Disruption

ATTTATATCAATGCCTTCTGTAATCTTAATCCCATCAACAATAATTCATCACTTTGCTCTTCAGGACTCAAATATTCTTGCTCAACGATACCACGACCTC

>B488_07050|785945..785852|Liberibacter_crescens_BT-1_EZ-Tn5_Transposon_Disruption

GTCTATGTTTGTAGCGCCCGCGCGGTCATAAATCACATCGACCAGAGCTGACATACCCCGTCGGTCGGATTCAAGCACTCGCGTAGCAAGTCTCCGACGG

>B488_00700|84567..84666|Liberibacter_crescens_BT-1_EZ-Tn5_Transposon_Disruption

AGTACTTACTGTAACGAAAGGAGCCGCTTCGGTTGTTTTTTCAATTGGTTCGGCTCTTTCTTCAGAGACTCTTGTGCAAGGAATTGAAAATCTTTATCTG

>B488_00530|67255..67156|Liberibacter_crescens_BT-1_EZ-Tn5_Transposon_Disruption

TACCTAACCTGGCCTCGGAAAATATTTTTTTTGGGAACTTCTCTACATCCGCAATAAGGAGAGATAACTTAGATATCATCTCAGCACGGGTACGCTCTAT

>B488_07770|856123..856044|Liberibacter_crescens_BT-1_EZ-Tn5_Transposon_Disruption

CTGTTGAACATATTTTTAGTTAGTACAACACCCTCTAATATCCCCGGGAATCTCATCGGCCTCTATCAGGAGGCGCCGTCCGCCACGCGCGCCGGTCGTT

>B488_01270|155497..155587|Liberibacter_crescens_BT-1_EZ-Tn5_Transposon_Disruption

CACTTTTATCAGTAAGTGTTTTAACAGAAAAGGACATGATAGATCTTGATGTAGCATTACAAACTGGTGAAGTCGATCGGATAGCCTTATCCCCATTCCA

>B488_05630|612185..612286|Liberibacter_crescens_BT-1_EZ-Tn5_Transposon_Disruption

CTATCAGGCTTCTTTTCTGTTGATAATTCTGCTCTTTTATTACGTCCTGGGATGACGGCAACAGCGGATACACCATTAATAAAGTACAGAATAAGTCGCT

>B488_05140|554929..555028|Liberibacter_crescens_BT-1_EZ-Tn5_Transposon_Disruption

CACCTGCACTCTTTCCTGCGCCTGTAAAAGGTCTGGTTACTGTAGAGGATTTTCAGGATGGCACAGCGGGATCGGCTCGGGTTCTCAGAAACTTCTGGCC

>B488_09220|994284..994383|Liberibacter_crescens_BT-1_EZ-Tn5_Transposon_Disruption

CATCAGAATGTACAACATGTAGTCATAGTCATCATCATTTAAATACAACAATAACACACTCCAAGAGACTCGAAATCATTCAGATACTTATAACAAGTAT

>Intergenic_Region|527686..527736|Liberibacter_crescens_BT-1_EZ-Tn5_Transposon_Disruption

CACCTGAACAGAGCAAAAATTAACAGCTAATAACCCGTTCAGGTGATCCTTCAACTCAGCAAAAGTTCCGCACTCAGTCACTAGCAACGTTCCCTTCTTC

>B488_10170|1112870..1112774|Liberibacter_crescens_BT-1_EZ-Tn5_Transposon_Disruption

AATATACAGCCAGTTATTAAAGTAGTAGTCATTGTTAGTACAATCGTTGGAGCAGGAGCGCTGTTTATAAAAAAGCTAAAATAGACGCCTATAAAACAGC

>B488_05500|596766..596865|Liberibacter_crescens_BT-1_EZ-Tn5_Transposon_Disruption

AATAAGACCTGTTACCAAGAACGCCACCAAAAGGTTTCTTGAAACTTTTACGATAGGGCCGCATATTTCCCAGGATGGAATGATAGGTAACAGCTCTCTA

>B488_05630|612185..612281|Liberibacter_crescens_BT-1_EZ-Tn5_Transposon_Disruption

CTATCAGGCTATCTTTTCTGTTGTATTCTGCTCTTTTATTACGTCCTGGGATGACGGCAACAGCGGATACACCATTAATAAAGTACAGAATAAGCCGCAA

>B488_03600|414348..414248|Liberibacter_crescens_BT-1_EZ-Tn5_Transposon_Disruption

CAACATACCTCCTGCAACACCAGTTGCCGTAGACTAGCACTAGCTAAGAAACCTCCTCCTCCAAAACCCTGTTGCACAGGAGGAGGACTCATCTGAGGAG

>B488_12920|1404457..1404358|Liberibacter_crescens_BT-1_EZ-Tn5_Transposon_Disruption

CTCTAGGTGGGGCTATAAACAATAACTTTAATCTTCTTGCTGCAAAGATGAATAATGCAAGTTCAAAATATCCATCATGGAACCGCATAAGGGTCTTTAG

>Intergenic_Region|894757..894661|Liberibacter_crescens_BT-1_EZ-Tn5_Transposon_Disruption

CTCTATATTACATCGAGTTAATTCTATAATACAAGGAGAGCGTTCTCTAGCCATCATGAGAGGATACAGTGAGTTTCTGAAGTATATCCATAAAATGTTA

>B488_03650|418504..418604|Liberibacter_crescens_BT-1_EZ-Tn5_Transposon_Disruption

TTGTAAAGCTTTCCTCATTTGCTACGTTTCAGATACGCAAAAAAAATCAGCGAGTTGGTCGCAATCCAAGAACAGGGGAAGAACCATCATTTTAAAGCGT

>B488_05410|584772..584872|Liberibacter_crescens_BT-1_EZ-Tn5_Transposon_Disruption

GGTATATTCATTGATCAGCTTGAGCTCTCGCCGGAGACTTTTTCTTCATTCCGGGCTGCTTTGCTTAAGGATTTTTTTAGAGTTGGTAAGGATAGCAGGT

>Intergenic_Region|894757..894658|Liberibacter_crescens_BT-1_EZ-Tn5_Transposon_Disruption

CTCTATATTACATCGAGTTAATTCTATAAAACAAAGATATTGCATTGAAAAATTGAGAGGATACAGTGAGTTTCTGAAGTATATCCATAAAATGTTATTT

>B488_05910|650337..650441|Liberibacter_crescens_BT-1_EZ-Tn5_Transposon_Disruption

GTTCCATGGTGTTACTTTGGGTGGTACAGGCAAGGAGCAGGAAAACGTCCTCTAAGGTCCCTCGAGGAGCATCACTGGCGCAGCTCAAAATTCCTGGCAA

>B488_03730|427400..427301|Liberibacter_crescens_BT-1_EZ-Tn5_Transposon_Disruption

ATTATTGCCAATAATAATATCAGATTTTGGGCCTCCGATCACATTTTCAATAATCGCCCCTCTGGCAATAGAGAGATTTTTTTTTCGGCCACCAAGATCA

>B488_09080|978598..978499|Liberibacter_crescens_BT-1_EZ-Tn5_Transposon_Disruption

GTTTAGGCATGGTTAGTACAAGGGGTGATCTACCGCTTCCCTTTCTGTTTCAGGAGAAGGGATTGTGAATTCAGGATAGACACGAAAGGTCAATCCCCCC

>B488_05180|561018..560919|Liberibacter_crescens_BT-1_EZ-Tn5_Transposon_Disruption

ATTCAGTGTTGCAGCATTTCCTCCCAAATCCTGTATATGACCAAGGGCTACTTTTGCCCCTGTTTGCAACTCAGGATGATAGCCAAAATTCCTGAATTCA

>B488_12330|1351733..1351666|Liberibacter_crescens_BT-1_EZ-Tn5_Transposon_Disruption

GTGCTGCATTATGACGAAACAAAAGGCACTCTTGCAGAGAATACCTCGTCGTGGCATCAGAGGCTCCCGCGCCGGCCAGTCCGCATCATCGTCGAACAGG

>B488_09380|1011005..1010933|Liberibacter_crescens_BT-1_EZ-Tn5_Transposon_Disruption

GTGCATACGGGATTGTTACACGTATATATGCACTGGGATTACAATTCTCCGGGACAATTATTTCTACAATGATCCTTCAACTCAGCAAAAGTTCCACAGC

>B488_08300|908258..908357|Liberibacter_crescens_BT-1_EZ-Tn5_Transposon_Disruption

CTGTTGATAATATAACAGTAGCAATCGCCAATGCACGTGAAAGTTCTGCTTCTATAAAAAGCATAACAGAGCAGCTTTCTCAAAAACAGAACGAAATGAC

>Intergenic_Region|591151..591251|Liberibacter_crescens_BT-1_EZ-Tn5_Transposon_Disruption

GGACTGTTATCGTATTTTTGCAGAGTTTTATTTTATATTCATGTTTTTGTTTAATTTTTGATACAATAAGGCTTGTTAAGGAGAAAAAGAGTGCACGATA

>B488_09470|1019191..1019092|Liberibacter_crescens_BT-1_EZ-Tn5_Transposon_Disruption

CTCTAATGATGTAGAGCCAATTCGTGTATTTTCTGATGGACGTGATCCGGAGATAAAAGGGGAAGTATTTCCTTTGCCACGTCGGGAACGCAGAATTCTG

>B488_01150|135544..135445|Liberibacter_crescens_BT-1_EZ-Tn5_Transposon_Disruption

CCTTAAGGTAATTATAAAATTCTTTTACGTATTCAATTGAAACAATTTTATGAGGAGCAAGGCCTCTTTTTAAGAGGGCTCTCGTTACAACTCCAGTTCC

>B488_08300|907429..907526|Liberibacter_crescens_BT-1_EZ-Tn5_Transposon_Disruption

ACATACTGGTTCTCCAGTCATGAGGAATCCAAAGGACCAACACACAACCTTATCATTCGTATTCCAGGTTCTACTAATGGCCTATCGGTTGGTTCTCCCG

>B488_10970|1195859..1195761|Liberibacter_crescens_BT-1_EZ-Tn5_Transposon_Disruption

CCATTATGCTCTTGAAGTTGAGGGAGATTCAATGAATGGAGTTGGTATCTTAGATAGTGATACTCGTTATTATATGCAGCTGTGAAACTGCAAATTCAGG

>B488_07960|873256..873287|Liberibacter_crescens_BT-1_EZ-Tn5_Transposon_Disruption

CCCCAACCTATCTCGATATGTTTAAACATAATACAAGGGGTGTTATGAGCCATAATCCCAGATTTGGTTGCTACTTATGTTTTGAGCATTCTCGGGAAAT

>B488_03730|427400..427301|Liberibacter_crescens_BT-1_EZ-Tn5_Transposon_Disruption

ATTATTGCCAATAATAATATCAGATTTTGGGCCTCCGATCACATTTTCAATAATCGCCCCTCTGGCAATAGAGAGATTTTTTTTTCGGCCACCAAGATCA

>B488_04680|525324..525224|Liberibacter_crescens_BT-1_EZ-Tn5_Transposon_Disruption

GGCAAGGCATCATGGGACAGGCTTGCTGCCGAGGACTGGAAGCTGATCTTCCTTGATGCCCTGCGAAAGGAAATGCGCATTGTTCCCAATCTTGAAGGAA

>B488_01210|145407..145505|Liberibacter_crescens_BT-1_EZ-Tn5_Transposon_Disruption

GCTCTGGAGCGCTCAATCTTTTTCCCTATCTTCTCTGATAATTTAGAAAAAATAAAAAAAAGTAAGAAAAAAATAATCATTGGTTTCAGTGATGTAACAG

>B488_05430|587470..587371|Liberibacter_crescens_BT-1_EZ-Tn5_Transposon_Disruption

CTCTTGATACTCCCTCTGATGGTTGATCCTCTAACATTTTCTCTAGCCTTAATGATGTTTTTAGCCTCGCTAATAGTAGGAGTTCTATACGCAATGATAG

>B488_11940|1304289..1304386|Liberibacter_crescens_BT-1_EZ-Tn5_Transposon_Disruption

GCCTTCAAGAATAGACCCGATACTCTTCGGCTCCATAAGAGAAGGAGTACCTCCGCCAAAAAAAACGCTCGGTAATCGTTCTAGGACCACTTAATTCGTC

>B488_01150|135544..135445|Liberibacter_crescens_BT-1_EZ-Tn5_Transposon_Disruption

CCTTAAGGTAATTATAAAATTCTTTTACGTATTCAATTGAAACAATTTTATGAGGAGCAAGGCCTCTTTTTAAGAGGGCTCTCGTTACAACTCCAGTTCC

>Intergenic_Region|1105172..1105073|Liberibacter_crescens_BT-1_EZ-Tn5_Transposon_Disruption

GTATAATCCATACATAAACATATTTTTGTCATATAAGAAATTATATTTAAGATTAATAATTTATTAAAAGGATTTTATAGACTATAATGACGGGTCTTCA

>B488_09220|994284..994383|Liberibacter_crescens_BT-1_EZ-Tn5_Transposon_Disruption

CATCAGAATGTACAACATGTAGTCATAGTCATCATCATTTAAATACAACAATAACACACTCCAAGAGACTCGAAATCATTCAGATACTTATAACAAGTAT

>B488_02000|237410..237310|Liberibacter_crescens_BT-1_EZ-Tn5_Transposon_Disruption

GACATACCAGTCGCTGCATAGAAAGAGCTCTCATAGGTTAATTTTCCTTCAATTATATCTGCATTTTTGTAATTTCGAGATAAGCGGAAACAAGCTTATC

>Intergenic_Region|675675..675578|Liberibacter_crescens_BT-1_EZ-Tn5_Transposon_Disruption

GCTTTATACCTATCATGGATCTCCCATAGTTTAATTAAACAATTTCAAATATTCATCACCACCATTCTTCTATTTTTCCAGCATCTTATTCGCAACAGCA

>Intergenic_Region|386738..386639|Liberibacter_crescens_BT-1_EZ-Tn5_Transposon_Disruption

ATATTTGCTTACTTACCTCAAGAATCAAGTTATTGAGCTGTTAATAAATATTAATTTGAATACCTTTAACAGAAAATATGACTTTTTCTGCATTCAAAAA

>B488_13510|1471794..1471894|Liberibacter_crescens_BT-1_EZ-Tn5_Transposon_Disruption

GGCGTAGCACGTGTAATACATGGGTGGTCCGGAGCCAAAACCCTGAGATGGTTTATTCTGCTTGTAAACTTTTTCCCGGAAGAGTTGTCATCAGCATCGA

>B488_01430|176902..177001|Liberibacter_crescens_BT-1_EZ-Tn5_Transposon_Disruption

ACCTCAGATATTGATATTCTAGATACTCTTTATTTAAGAGGGTTGGTTCCGATGGTCGTGGCAATCTCTAGCTCCTTTCTTTTAGGTGTAGGGTTGTTTT

>B488_03100|356527..356428|Liberibacter_crescens_BT-1_EZ-Tn5_Transposon_Disruption

GAATTACTCACTGGTATGAAAATACTTTGGTAATCTTCAATAGAGTTACTAGCATAACTTTTTTGAAAAAAAAGGAAAAATATTATGAGTATAGCATGTA

>B488_05170|559700..559601|Liberibacter_crescens_BT-1_EZ-Tn5_Transposon_Disruption

GATGAGGAACAAGGACATTGATAAGTGCCTGCGCTGCCTGATAATTGCTTGATTCAGGGCCTGTTTGTATGCCGTATTGCGCAAGTTTGCCTTTTACGCC

>B488_10390|1135519..1135420|Liberibacter_crescens_BT-1_EZ-Tn5_Transposon_Disruption

GTTGTAAGGACAGATAGGGGTGATTTTATACTTGATAATATGCACGATGATATATTATTGTGGTTAAAGACTGAGTATAAATATATAAAGAGACAATCTC

>B488_04940|543102..543004|Liberibacter_crescens_BT-1_EZ-Tn5_Transposon_Disruption

TTGTTTCAGATTCTGAAACAGATTTGCTTAATGAAGATACCTCTCTCTTCCCAGAACTCAATATTAAATTTACTATTATTTAAAGTCTTTAATACTTCTT

>B488_08370|913731..913830|Liberibacter_crescens_BT-1_EZ-Tn5_Transposon_Disruption

TTTATCGACTCCATATGCTGCAATAGCATATCCTGGAATTTGGAATAGAACACTTAAAAGGAGAAAATGATATCCCCGTAAAAAACCAATCATATGTTGT

>Intergenic_Region|1236364..1236265|Liberibacter_crescens_BT-1_EZ-Tn5_Transposon_Disruption

CCTTAAGCTATTAATTCAGATCAAAAAAGATTAATCCTTTAAAAAAGGACTATTATACAGTATATATTTTCTATCAAGGGTATCCTAAGTTTACATATCA

>B488_05160|557059..556960|Liberibacter_crescens_BT-1_EZ-Tn5_Transposon_Disruption

CTATAGTTCAGTGTGCCATAAGGCGTCACCTGATTGACATTGCTTAAGGCAGATCCCGCCATTCCCGAGGCAATATTGCCTGCAAACTGCGCTCCTGCTA

>B488_05670|616155..616058|Liberibacter_crescens_BT-1_EZ-Tn5_Transposon_Disruption

AACCTGCATAACGCTACACGACGCTTTTCTCCACCAGAAAGTTTCTTGTACACCTGCATCACCAGACGGACAGCGTAAGGCCTCCATAGCCATTTTCCAG

>B488_07970|873508..873607|Liberibacter_crescens_BT-1_EZ-Tn5_Transposon_Disruption

GGTTTAAGCAACTCAATATGCTCTAATGCTTCTGAAAGGGAAAAGTGACTCTTGTGCACCTGATACTGCAAGGCATCAATGATCAATATATCCAGATTTT

>B488_03690|422799..422898|Liberibacter_crescens_BT-1_EZ-Tn5_Transposon_Disruption

ATGTAAAAATATCCTTAACGTTTAAAGATGATACATCTACATTCATTCCAGCACCAGGTTTTATGACATCTGCAACGATAATTCCAATTATTAATGCTAA

>B488_01970|235156..235058|Liberibacter_crescens_BT-1_EZ-Tn5_Transposon_Disruption

ACTGAATCGGTAACGTCTAATACAAGGGCTGTAAGCTTCCATAACTAGAACAGATTTCAAATTCTCTAAGTCATTGCTACTCCGAGCTGAAAGGACAAGT

>B488_11130|1212460..1212557|Liberibacter_crescens_BT-1_EZ-Tn5_Transposon_Disruption

GTATAATGATGGCATTGATATTTCTGTCCCTTCGGTACCGCTATTAAAGCAGCAGAAAAAGGAGTTGTTATTTATGTTGGGAATAGTCTCAAAAAATCGG

>B488_07970|873508..873608|Liberibacter_crescens_BT-1_EZ-Tn5_Transposon_Disruption

GGTTTAAGCAACTCAATATGCTCTATGCTTCTGAAAGGGAAAAGTGACTCTTGTGCACCTGATACTGCAAGGCATCAATGATCAATATATCCAGATTTTG

>B488_12920|1404531..1404431|Liberibacter_crescens_BT-1_EZ-Tn5_Transposon_Disruption

ATCTGGAGCCACGGCTATAGAATATGGTTCGATCGCAGCTCTCCGTTCAGTCGCCTTAATCGCCGGTGCAACAGCTCCGGCGGGGCTATAAACAATAACT

>B488_05550|594001..594060|Liberibacter_crescens_BT-1_EZ-Tn5_Transposon_Disruption

GTACTTGTCCCAACCGTATAAAAGCGATGCACCTCTGTATCGCCAACAGATCCACGCTCCGCCTCCCCTGAGATCGGCACCAGATCCTTCCCGGCCTTCC

>Intergenic_Region|287008..286910|Liberibacter_crescens_BT-1_EZ-Tn5_Transposon_Disruption

CTACATCACCCACCAAGTTTAAATTCCCTACTTTTAATAACCCCATTATGTTATATCGCAAAACTATATTAAATAAAAAATCTATTAGTCCGAAAATAAT

>B488_11140|1213347..1213443|Liberibacter_crescens_BT-1_EZ-Tn5_Transposon_Disruption

CTGCTATGCTTATACTTTACTGGCATCAGGTTATATCGACTTGGTTATCGAAGCAGACCTTAAACCCTACGATATAGGAGCTGTTATTCCTTTAATTCGA

>B488_01270|155497..155596|Liberibacter_crescens_BT-1_EZ-Tn5_Transposon_Disruption

CACTTTTATCAGTAAGTGTTTTAACAGAAAAGGACATGATAGATCTTGATGTAGCATTACAAACTGGTGAAGTTGATTGGATAGCCTTATCTTTCATTCA

>B488_11970|1306872..1306971|Liberibacter_crescens_BT-1_EZ-Tn5_Transposon_Disruption

GTCGAGCGAACCTGTTAGAAGGAATTGAACCAACAGATCTAGAGCGATTACGACTTTTATTTGATGATCTAGAGAAAAAAGAGGTTCTCATAGAAATGCT

>B488_04870|537714..537615|Liberibacter_crescens_BT-1_EZ-Tn5_Transposon_Disruption

CTTCTGTACCGATAAAGACAAAGACAAGCACAGCTCCAACAATCTCAAAAAACAACATCGGCTTCTCCTCCATTATCCTGTCAAACCAGTCTCTTAAAAA

>Intergenic_Region|105859..105958|Liberibacter_crescens_BT-1_EZ-Tn5_Transposon_Disruption

ATTTAGTACAGACTGGTTAAATAGTATATAAGATCGGTATATAAAATTATCTTTTTATTTAACAATGAAAAATTATCCTGAAATTATATGAATCTTACCT

>B488_10170|1112870..1112773|Liberibacter_crescens_BT-1_EZ-Tn5_Transposon_Disruption

ACTATACAGCATTTATTCAAGTACGTAGTCTTGTTAGTACATCGTTGGAGCAGGGAGCGCTGTTCATAAAAAAGCTAAAATAGACGCCTATAAAACAGCA

>B488_00130|17230..17329|Liberibacter_crescens_BT-1_EZ-Tn5_Transposon_Disruption

GTCATACACCTGTAGAAACAGAAGCTCTTTTTAATCATGGAGTATGGTGCATAGATTGGCCAATAAGATTACATATCCCACACCAACTAAACATTTAATC

>B488_11150|1214918..1214819|Liberibacter_crescens_BT-1_EZ-Tn5_Transposon_Disruption

ATATCACGGTAGATTGTTCCCATAATATTAAGAGGAACAGTAAGCTGGATAAGTAATGCATTTATCAGAACAAAATCACCTACAGTTTGTTGTCCAAGTT

>B488_10170|1112862..1112773|Liberibacter_crescens_BT-1_EZ-Tn5_Transposon_Disruption

CCTATACGCTAGTTATAAAAGTAGTAAAAGTTGTTAGTACTATCGTTGGAGCAGGAGCGCTGTTTATAAAAAAGCTAAAATAGACGCCTATAAAACAGCA

>Intergenic_Region|329965..330063|Liberibacter_crescens_BT-1_EZ-Tn5_Transposon_Disruption

CTGAATTGAAGCCCCAGTAAACGGCGGCCGTAACTATAACGGTCCTAAGGTAGCGAAATTTCCTTGTCGGGTAAGTTCCGACCTGCACGAATGGCGTAAC

>Intergenic_Region|329973..329874|Liberibacter_crescens_BT-1_EZ-Tn5_Transposon_Disruption

TCAATTCAGAGCTTGCACTCCTCCTCTTAACCTTCCAGCACCGGGCAGGCGTCAGGCCCTATACATCGTCTTAATGACTTAGCAGAGCCCTGTGTTTTTG

>B488_03380|389751..389837|Liberibacter_crescens_BT-1_EZ-Tn5_Transposon_Disruption

ATCTTAGCTATATATATTTACTAGACTTCGTCTTAATCCTGGAGGCTTTCTAGATCAAGCAATTAGTGTTTGCTGATTATTTCTTAGAAAAAGGGGAAAT

>Intergenic_Region|329091..328993|Liberibacter_crescens_BT-1_EZ-Tn5_Transposon_Disruption

CCCATAGCCTATCCGTGGCTCTACCCCCGAGGGTCTTCAGTTAACGCTCTACCTAAATAGATTTCGCGGAGAACCAGCTATCTCCGAGTTCCGATCGGCC

>B488_05920|651371..651470|Liberibacter_crescens_BT-1_EZ-Tn5_Transposon_Disruption

GCACTTGTACTAAATAGGGCAAGCTTGGTTACTCTTTCTGGAGCTTGGCGTAAGATTTCAAATGCAACATATCCTCCCATAGAAAATGCTGCTATTGAAA

>B488_10170|1112871..1112772|Liberibacter_crescens_BT-1_EZ-Tn5_Transposon_Disruption

AATAGCAGCAGTTATAAAAGTAGTAGTCATTGTTAGTACAATCGTTGGAGCAGGAGCGCTGTTTATAAAAAAGCTAAAATAGACGCCTATAAAACAGCAT

>B488_05320|574992..574893|Liberibacter_crescens_BT-1_EZ-Tn5_Transposon_Disruption

CCTTTACTCGTTGTACAAGGAGAAGGATCTCCTCTTCGTTTAAGTATTTTTGGGCCTTTAATGAGCAAGAACATATCTTATCGAATATCTAAAGACATAC

>B488_07750|853740..853839|Liberibacter_crescens_BT-1_EZ-Tn5_Transposon_Disruption

GCTGTGCATCGTCTTCATCCGGGATACGATGAATCACGGCCTGACACTTCAATTCCTCGAGGGGATAACGGTACATTTCCCTGAGAACATGGCGATTCAA

>B488_13240|1438260..1438359|Liberibacter_crescens_BT-1_EZ-Tn5_Transposon_Disruption

TCTTTGGACTGGGAGAAACTGTTGATGACCGCGTTGATATGTTAGTAACCCTTTCTAACTTAAAAAGTCCACCTGAAAGCGTACCTATTAACATGCTTAT

>B488_10960|1194776..1194678|Liberibacter_crescens_BT-1_EZ-Tn5_Transposon_Disruption

GATCTAAAGAGAGAGGTATTAAAAATATTGTAGCAAAAGCTGCAGGGATAACTATGAATGATAGTAACGGTATGGCAGCGAGATCCGGCAAGAAATCCAT

>B488_03660|419051..419150|Liberibacter_crescens_BT-1_EZ-Tn5_Transposon_Disruption

CTGTACAGGCAATGCTTTTGCAGGAGCGGTTCAGTTTGAGCGTATAAAAGCAAAGGAGAAACCTGATTTTATCCCGTCAAGGCTGTTTATTTATTATAAT

>B488_12780|1393148..1393107|Liberibacter_crescens_BT-1_EZ-Tn5_Transposon_Disruption

TTGTAAATATAGGTATAGGTGGTTCAGATCTTGATCCTTCAACTCAGCAAAAGTTCAGGACTGTTATTTCCCATCTTGCTGTTGTAACTTATATTATAAA

>B488_10680|1164219..1164120|Liberibacter_crescens_BT-1_EZ-Tn5_Transposon_Disruption

GCCTTGACCTGTTTTAGCTGAAATAAGGATAGAATCTTCTGAATCAATTCCTATTACATCTGTGATTTGTTTTTTTACACGATCGGGTTCCGCAGCAGGG

>Intergenic_Region|1048526..1048427|Liberibacter_crescens_BT-1_EZ-Tn5_Transposon_Disruption

GACTAGTAGTTGCTATGATTTATTTTCGACGTAATAATTAAGTGTTGTAAAATTAGATCGAGTTAATAATTAATTAAGAATTAAACTAAACAGAGGTTTT

>B488_06090|673033..672927|Liberibacter_crescens_BT-1_EZ-Tn5_Transposon_Disruption

TTTCTTTCTCTTTAATGCTCTTACCAACTACCTGGTGTTAGTGGTGACAGCGCGCACCAGTCCAGTACTATTCTGGTCGCAGGAGGATAGGGCCGCGCGT

>B488_06090|673025..673124|Liberibacter_crescens_BT-1_EZ-Tn5_Transposon_Disruption

GAAATGAAATAGCCAATCCACAAATCACCAGATTTTCTACCTGTCGTTTCCGTTGTAGAAAAAATAGCAACGTTACAGCCAGTGCAGAACAAATGCCACC

>B488_08140|883892..883991|Liberibacter_crescens_BT-1_EZ-Tn5_Transposon_Disruption

ATCCAGCGCTAATCCAAAGCGCTTCACCGCTATCATTTTTTGGACCAAAACGTAAAACTCCTCCAGCAGCAACCGTGGAAGAATTGCCTAAAATTGATGT

>B488_11600|1259871..1259970|Liberibacter_crescens_BT-1_EZ-Tn5_Transposon_Disruption

GCTTCGATCTGGGTCTCTCCTGGACCACCCAGGAAACCAGTACCTCCTCGTTGCCGCTCAAGATGCGTCCAACTACGAACAAGACGACCTTTTTGATAAT

>B488_01390|170223..170322|Liberibacter_crescens_BT-1_EZ-Tn5_Transposon_Disruption

GTGATGTTTAAGTTGTTCTTTTTGTGTTTAAGTGTAACTATAGTTTCGGGTTAGTTTTTCATGTATTGCCATTCCTTGAAAGAAAAGAAAGCTTTTATTT

>B488_02780|315883..315982|Liberibacter_crescens_BT-1_EZ-Tn5_Transposon_Disruption

GAATAGGCTTGTTAAGCAGATTGCTGAAGATATGGCATTTCCACGTATGTTCGATATACGCTCCGTCTCCGGAAGGTCCCCCGAGTTATATGCTACAGAA

>B488_11490|1246874..1246775|Liberibacter_crescens_BT-1_EZ-Tn5_Transposon_Disruption

ATGTTGGTCTAGCAATTAAAGATCAGAATTTGCCAGCTGTAGGGTATGCTTTGTTAACAATGTTGTTAGTAATTATTTTTTATGATCAGTTGTTATTTCG

>B488_00790|95837..95936|Liberibacter_crescens_BT-1_EZ-Tn5_Transposon_Disruption

GTGTCAATCTGTAAAAATTGATGTTATTGTGATTGATGTGAATATTTTTGATGCTTTTGAAATATGTGAAAGAATAAAGTCTTATTATTCTATTCCAATT

>B488_11630|1266962..1266863|Liberibacter_crescens_BT-1_EZ-Tn5_Transposon_Disruption

GCTTTGGGAGGTGCAGGAGGCGCATTATTCAGTAAAATGTCAGAGAAAAATCGTCAGTGTTATGAAAGAGATCGTTATAATCGCCGTTATTTGGTTCCTT

>B488_03660|419059..418960|Liberibacter_crescens_BT-1_EZ-Tn5_Transposon_Disruption

CCTGTACAGGAACCAATTTTTCCCTGATCTAGACAGGAAAAGAAGGACAATTATCAATTCTTCGGTGGTAAAACCTGTAAAACAGATAAAGAAGCACTAT

>B488_03660|419051..419150|Liberibacter_crescens_BT-1_EZ-Tn5_Transposon_Disruption

CTGTACAGGCAATGCTTTTGCAGGAGCGGTTCAGTTTGAGCGTATAAAAGCAAAGGAGAAACCTGATTTTATCCCGTCAAGGCTGTTTATTTATTATAAT

>B488_07710|850382..850481|Liberibacter_crescens_BT-1_EZ-Tn5_Transposon_Disruption

GCCCAAGAATCTGCACGGAATCAAGGAAATTTTCTTGTGTACAATGAAGAACCAAATATCGGACACGTGAACTAAACCCTTTTATACTGCGATAGATATG

>Intergenic_Region|894757..894658|Liberibacter_crescens_BT-1_EZ-Tn5_Transposon_Disruption

CTCTATATTACATCGAGTTAATTCTATAAAACAAAGATATGGCATTGAAAACTTGGGAGGATACAGTGAGTTTCGGAAGTATATCCATAAAATGTTATTT

>Intergenic_Region|894749..894848|Liberibacter_crescens_BT-1_EZ-Tn5_Transposon_Disruption

AATATAGAGAGCCCTCTATACAATAAAATTGTATCAATAGAGAAATAAAAATTTGAAAAAACCTTGTAATATCATCCTTTTTCTATAGGGAATTACAGTT

>B488_04700|526441..526342|Liberibacter_crescens_BT-1_EZ-Tn5_Transposon_Disruption

GCTCTACAGTTACCTATCTTTCACGCTACACCCTCAGGGCAGCATTGGGATTGGCGGCTTCTGCTGATGATGATGGAACCTATGCAAGATATAACAATGG

>B488_01970|235156..235057|Liberibacter_crescens_BT-1_EZ-Tn5_Transposon_Disruption

ACTGAATCGGTAACGTCTAGTACAAAGACTTTATGCTTCTCTCCTAGAACAGATTTCAAATTCTCTAAGTCATTGCTACTCCGAGCTGAAAGGACAAGTA

>B488_04700|526433..526532|Liberibacter_crescens_BT-1_EZ-Tn5_Transposon_Disruption

CTGTAGAGCCGATAGAATGATTGTAATTTTTTCTGTTGCCCATGGCTTCATCAGGAGGAGCAGAAAGGCTGTTTCTTTCACGATGCCCATCCTTGTGAGA

>B488_07670|848029..848128|Liberibacter_crescens_BT-1_EZ-Tn5_Transposon_Disruption

CTATTATTCCACGATTAAAACCACATACGTTTTAACAAGGAATCTTTTTTAACAAATTGATGCCAAAGCGCTACAAGTATATGCAAACCAATGACATAAT

>B488_07660|848037..847938|Liberibacter_crescens_BT-1_EZ-Tn5_Transposon_Disruption

GAATAATAGCTTAAAAAGCGATTGTATGATCTCATGGGATTTGATCGTATTACAGGTGATCGAATAGATGGTGTTATCGTTTCTTGCAAGATTTCCATTC

>B488_07040|782278..782188|Liberibacter_crescens_BT-1_EZ-Tn5_Transposon_Disruption

TAATACAATTGCCCGCCCTCTATCCATACGACAGGGTTTCCCTCATCCTTTTCAATCACAGTGTGGTCACTACCATTCTGAATTGAAACTAGCCTCTTAA

>B488_11590|1258590..1258689|Liberibacter_crescens_BT-1_EZ-Tn5_Transposon_Disruption

AGATTAATGAGCCTCATAATGTAGGAGCTATTTTACGTTCTGCTGTTGCTTTTGAATGCGGTGCTGTTATTACTACAAGACGCTATAGTCCTTCTGAATC

>B488_11590|1258598..1258499|Liberibacter_crescens_BT-1_EZ-Tn5_Transposon_Disruption

CATTAATCTGATCCAATACAATTAACAGTTTGCAGTTTTTGATTGAATCAATATCTCTTGATGGCAAAGGATGTGTTTCTAGCATGACTCCCTGATGAGT

>B488_05430|587045..586946|Liberibacter_crescens_BT-1_EZ-Tn5_Transposon_Disruption

CTTATTAATACCTCTTATTGTAGCTGTTATAATGACGCCATCCTTGAGCAGTATAAAGCATTAACAAGATAAGAATCATCATATTTTTTAAGTAAAATTG

>B488_05430|587037..587136|Liberibacter_crescens_BT-1_EZ-Tn5_Transposon_Disruption

ATTAATAAGAGCACCATCAAAAGGTTTCTTAAAACATTTATGATAGGGCGGCATACTTCCCAGGATGGAATGATAGGTAAGCGTTCTCTATCATCGTCAT

>B488_04500|502668..502569|Liberibacter_crescens_BT-1_EZ-Tn5_Transposon_Disruption

CTTGTTGATACCATTATTCTGATCTTTTCAGTATCTGTCATTAATTTTGTAAAATTTTCACCAGAATTATAGCTATCAAAAAAAGATGGAGATAATTTCA

>B488_12840|1399359..1399260|Liberibacter_crescens_BT-1_EZ-Tn5_Transposon_Disruption

GGTATGGATATTCCAAAGGCACAGCGACCTTATCAAGGTACAATTGGCTTCATATACAAATAAGATAGGACATGTAATGAACAATATACTTAGAAAACTG

>B488_04730|529209..529176|Liberibacter_crescens_BT-1_EZ-Tn5_Transposon_Disruption

CTTCCAGTAAAACCAACGGCTCTACAGAAGACGGGTCCCCCCTGCGTCAAATCGTAAAAAAGGTTAGGGGCGGCGTAGGAATTACAATGTTCTGAGCGTA

>B488_00930|109951..110049|Liberibacter_crescens_BT-1_EZ-Tn5_Transposon_Disruption

GTTATTAATAGATCACGCTTTGCCTTTGCGTTTAGCAGGTAAAAAGCCTGCAAAAAAGAAAGATTTGTTCGATAATCCAGAAGCTACTAATGCTGCTAAG

>B488_00930|109959..109860|Liberibacter_crescens_BT-1_EZ-Tn5_Transposon_Disruption

ATTAATAACTCTGGCATCTATTCCTTCTACACTTATTCTATTTGAAGCCCCTCACCGTCTTAAAGAAACCCTAAAAGATTGTTCAGAAATATTAGGAGGG

>Intergenic_Region|973130..973030|Liberibacter_crescens_BT-1_EZ-Tn5_Transposon_Disruption

CTCTTATGGTGTGAGGGCGACCAAAAAGGCTCCTTGAGCTCGAATAATTCCGCTCGTTTTAAGCGCCTACTGCTGGTCACGGGTTTTGTGCCTCCCGCCA

>Intergenic_Region|973122..973221|Liberibacter_crescens_BT-1_EZ-Tn5_Transposon_Disruption

CCATAAGAGAGGTATGTTTAACGCATAAAAATGCGTCTCTAGAAGAAGCTTTCAGAAGAACTATAATTGTTTTTGTTATTTATATCTTAATTTTTACTTG

>B488_09440|1015501..1015573|Liberibacter_crescens_BT-1_EZ-Tn5_Transposon_Disruption

ATTAATTCCTTTGATATTATTGTCCTCTAAAAATTTTTTAAGAGCACTATCAAAGGCCTTACCAGCCAATGAAACCCCCTAAAATAAACCAACACGGCTC

>B488_09440|1015509..1015410|Liberibacter_crescens_BT-1_EZ-Tn5_Transposon_Disruption

GGAATTAATGGTGAAACAGAACCTTCTGCTATGAATGGTGCTCAGATGCTTGATTTTATAAATCAACTGGAAAAATCTTTTGCAGATGACAAGTATTGGT

>B488_09670|1043376..1043306|Liberibacter_crescens_BT-1_EZ-Tn5_Transposon_Disruption

GCCCTTGGTGATGGGGCTCCAAAAAATTCTTTTCAGGCTTCCAAAATATACATCTGAAATCGCTAATACAAGGGGCGCCTCGAGCCACATGATACAGTTT

>B488_06720|745911..745813|Liberibacter_crescens_BT-1_EZ-Tn5_Transposon_Disruption

CTTCCCTGCATATAAAAAATAACAGAAATAAGACTCACTGCAATAATCCAGAAAATCAGTTGAAGCAATAGCTTTCTGTTCTCCAATGAAAGAAGAAGAC

>B488_06730|745903..746002|Liberibacter_crescens_BT-1_EZ-Tn5_Transposon_Disruption

GCAGGGAAGAGGGCTATAAAAGCTTTGATAATCATATGATGCCACGACAAGGTAATTTTTACCTCGGCAACTTCGGTTGTAACCTTTGCTATTTCCTTGC

>B488_05910|650218..650317|Liberibacter_crescens_BT-1_EZ-Tn5_Transposon_Disruption

TGTATACTGTACGGGGTAGATATTCATCCAGCTGCAAAGATTGGTAGCGGTATCATGCTTGACCATGCAACAGGGCTTGTAATCGGTGAAACTGCTGTTG

>Intergenic_Region|174735..174638|Liberibacter_crescens_BT-1_EZ-Tn5_Transposon_Disruption

ATATTATAGTATAATACAAAAAACAGTACTCGAGTATTTATAAGATACAGCTGCTCTATAAAATTTTTCAAGATCTATACAGATTTCTAATCTACAACAT

>B488_10960|1194493..1194394|Liberibacter_crescens_BT-1_EZ-Tn5_Transposon_Disruption

AGCTATAGTCGCTGCAAACGACATTTGAAAACTAGGCCCCATGACTTCTGAAGGTGTTATAAAAAGGATAACAAGAGCAGTCAATGCAATATTATGTAAA

>B488_10960|1194485..1194584|Liberibacter_crescens_BT-1_EZ-Tn5_Transposon_Disruption

ACTATAGCTCTTGTAGCAAGCTATACAAAGTGGGAAAACAAAGCTATCTCTCACCCTCTTCTCAAGACATTCCGTTATGGAATTATAATTACTGGCATAA

>B488_07290|813464..813563|Liberibacter_crescens_BT-1_EZ-Tn5_Transposon_Disruption

AGGTATAATACAGCGAGATCTCTTGGTTATAGTGGACCTCCTCACGGTCTTCTTGATGTTGAAACAAATCTTAAATATGCCGTAAAATATCTTCGTGGAG

>B488_05550|593927..593828|Liberibacter_crescens_BT-1_EZ-Tn5_Transposon_Disruption

CTTATGAGCAGGATGAACAACAGGATGATCTTCTGCATACATCATCAGAAATGTTCCATGATGATGCAATGATTGCATAAGAGCTTGGATCTCACTCAAG

>B488_05550|593919..594018|Liberibacter_crescens_BT-1_EZ-Tn5_Transposon_Disruption

GCTCATAAGAATTCTTGCCATAACTCTTCTGAAATTCACGATCGATCACAGTATCGATATAACCGTCTATTTCTACTGCTGAGTACTTGTCCCAACCGTA

>B488_05870|646524..646623|Liberibacter_crescens_BT-1_EZ-Tn5_Transposon_Disruption

GCATAACGTATAATACGGGTTTTCCCATGATGACTACCCTGTGTGTGCGGCGGAGGAGTGGTATCAAATGCTAGAACCTTAAGTCCAGCTTGTGCAGCAT

>B488_05870|646532..646434|Liberibacter_crescens_BT-1_EZ-Tn5_Transposon_Disruption

ACGTTATGCCTATGCTGAAGGAGAAAAATATGTGCCTATGCTCCTTCAACTCCGCACACGTTCAGAAGAAAGTCATGCATGATAGCGGCGAAAAATTGCT

>B488_13420|1458985..1459085|Liberibacter_crescens_BT-1_EZ-Tn5_Transposon_Disruption

CTTATAAGTCTCCACGGCCATATATCTGCAACAAGCGCAAGCGTCTTGAGGCGTTGGCCGAACCGGGTGAAATTCTCAAAAACATCGGATTGTATGATAC

>B488_04020|466446..466347|Liberibacter_crescens_BT-1_EZ-Tn5_Transposon_Disruption

GTGCTGTACGTGTGATTAATCCAACCGTATCTTTGGTATGAGAAATAGATAAAATACACTGATGAGGATAACATCCCTTCGTTGGTTTAAACCCAACAAC

>B488_06810|755794..755695|Liberibacter_crescens_BT-1_EZ-Tn5_Transposon_Disruption

CCTTAATACACTGGGGGCAGGATTTCAACTTGCCAGTCATGATCTTGATATTCGTGGAGCAGGTAATCTTCTTGGCGAAGAACAATCTGGGCATATAAAG

>B488_07970|873511..873412|Liberibacter_crescens_BT-1_EZ-Tn5_Transposon_Disruption

AACCTAAACAGGCCATCCTGACCCATATGCATATTTATATGGATTATGATACCCTTTTATGGGAAACGCCCGCTCATGTAAAGCCTGCCTTTGATGGAAT

>B488_01870|228217..228316|Liberibacter_crescens_BT-1_EZ-Tn5_Transposon_Disruption

GCACATAAGGTGTGGAATTTAGAGGAATCACAACTTGTTTCTCGTCTAAAGTTACTTATTTCTCGATTTGAGGAGACACGGGATTTTCGCCTTGTAGGGG

>B488_01870|228226..228127|Liberibacter_crescens_BT-1_EZ-Tn5_Transposon_Disruption

CCTTATGTGCTAATCGGGAAATAGAAGCGAGTGGATCTACAGGAGGATAACGCCCCTCTTCTGCTAGAGAACGCTTTAAAACAATATGACCATCAAGAAT

>B488_07040|782284..782252|Liberibacter_crescens_BT-1_EZ-Tn5_Transposon_Disruption

TATGCGAATTGCCCCCCTGATCCATAAACGGGAATACAAGGGGTGTTATGAGCCATCTTGTGGCTCGCCTTTTTTTTAATGGTAACCACCTTGTACTCAA

>B488_07040|782276..782375|Liberibacter_crescens_BT-1_EZ-Tn5_Transposon_Disruption

ATTCGCATATGCCTCAAATTACATGCACGAAGGTGAATCGGTGTTGCTCGGCAGGAAGGGTACGATTGATCGCCCTATGTATGTGAATGATAAATTCTGG

>B488_06090|673153..673054|Liberibacter_crescens_BT-1_EZ-Tn5_Transposon_Disruption

CCCTTACATTACCAGTATCAGCTTTTTGTGGTGGCATTTGTTCTGCACTGGCTGTAACGTTGCTATTTTTTCTACAACGGAAACGACAGGTAGAAAATCT

>B488_06090|673145..673244|Liberibacter_crescens_BT-1_EZ-Tn5_Transposon_Disruption

ATGTAAGGGTTCCCAACCACTCACCAAAGCGGGTGATAACCAATACTGCTCCTGTTGATGCGCCAAAAGAAAGACCGAAAAGGAATGGATCTGCCAAATC

>B488_09670|1043376..1043306|Liberibacter_crescens_BT-1_EZ-Tn5_Transposon_Disruption

GCCCTTGGTGATGGGGCTCCAAAAAATTCCTTTTCAGGCTTTTAAAATATACATTGAAATTGCTAATACAAGGGGTGTTATGAGCCATCAATTGGCTGGC

>B488_06260|690615..690547|Liberibacter_crescens_BT-1_EZ-Tn5_Transposon_Disruption

ACTTAAACTACAACAGCCTTGAGGCAACACGCAACCGAATTAAGCATTTTCAGCCTTTATTAATACAAGGGGTCGTTATGAGCCATCAACCATGGGGTGA

>B488_05160|557292..557193|Liberibacter_crescens_BT-1_EZ-Tn5_Transposon_Disruption

GAAGAGGGTTCATACGCCCGAACAGGGCATTGGCATAATCCTGCGTATTGCCCGTGTCCAGCTTCAGCGAGCCTGCCCCCTGACTGGCAACATTGCCCAA

>B488_02030|238922..238823|Liberibacter_crescens_BT-1_EZ-Tn5_Transposon_Disruption

AACTATAACAGCAGCAATATTTTTTGCTCCTAACTTACCTCCTTGTGTAGATATACCAAGATTCTGCAACATAGCGCGGACTGATTGTTCTGTAAAAGGT

>B488_08350|911566..911486|Liberibacter_crescens_BT-1_EZ-Tn5_Transposon_Disruption

ATATAGCTCTGATCTACAGAGCCTCTGGATTAATAGTGTAATAGTCTGTGTTGTCTTCAAGTTTACGCGTGCTAAGATCGACTCCCGTAATAGTACGACG

>B488_13090|1420201..1420102|Liberibacter_crescens_BT-1_EZ-Tn5_Transposon_Disruption

GAGCTATATCGATGCGCTGCGCCATGCCCTGCCGCTCGAGTGCCGCATCTGGAAGGCGCTGGATATGTGCCAGGCCCTTCTTCCGCGCGATCTGACCCAG

>Intergenic_Region|973122..973221|Liberibacter_crescens_BT-1_EZ-Tn5_Transposon_Disruption

CCATAAGAGAGGTATGTTTAACGCATAAAAATGCGTCTCTAGAAGAAGCTTTCAGAAGAACTATAATTGTTTTTGTTATTTATATCTTAATTTTTACTTG

>B488_04920|541099..541000|Liberibacter_crescens_BT-1_EZ-Tn5_Transposon_Disruption

GTTCAAGATAGGCACTAATATAAAAGCAGGGAGACGTAATAAGACCGTAATAAAACATGATAAGAGGGATAAGCAACCGCCCAAGCCTTCCATTGCCATC

>B488_04920|541091..541190|Liberibacter_crescens_BT-1_EZ-Tn5_Transposon_Disruption

ATCTTGAACAGAATAGAGATATGTACTACGAAAATTTGCTGTCAGTATCACGAGATGGTAATTGGCAGGGGTGGATTGAGTTTTTTTTGAAAGCAGTAGA

>B488_00540|68252..68351|Liberibacter_crescens_BT-1_EZ-Tn5_Transposon_Disruption

GAATATGACTATGTACTGTACAGATTTCATCTTTTGTTACTCCTTTTATAGCCATGAGTTGTAAGTGAATAGGCATAAAATACTCACCAATAATATGAAA

>B488_00540|68049..67950|Liberibacter_crescens_BT-1_EZ-Tn5_Transposon_Disruption

CTTTATAATTATTTCACGTGATAAAAAATGGGCTGAACCAAACCCATCTGAAAAAATAATAACTTCTTTGCTTTTTAATCTACGAAATATACCTTCAGCA

>B488_02320|266150..266056|Liberibacter_crescens_BT-1_EZ-Tn5_Transposon_Disruption

CTAGAATAATCAGGGAAAATACCTACAACATCAATATTATTGGTATCCTGAAATCTCAAACTTGTTAAATCTTCTAGCTCGCCACCTAATACAAGGGGTG

>B488_08150|884962..885061|Liberibacter_crescens_BT-1_EZ-Tn5_Transposon_Disruption

CTTCATTCCCTCATCTATCGACCAAGAGGATCAGAAACCGTCCAGGATGATCATACCGGAAAAACCGTTATTTTACCAACATTTTCCATAAACTATCTGA

>B488_08150|884970..884871|Liberibacter_crescens_BT-1_EZ-Tn5_Transposon_Disruption

GGAATGAAGGGTACGCGCACCAGAGGCACCACGGGCTCTAAGTGCTTGAGCGGCTTTTCCGGTAAAAGCTGCAAAGATAACATCACCATCAATATGTTCG

>B488_02010|237469..237568|Liberibacter_crescens_BT-1_EZ-Tn5_Transposon_Disruption

GCTTATAAGCGTGCACGTGTGGAATTTTCTGATTTGCTTTATCAGCTGGAACGAAGTGCTGGTGTCAAGGATCTTTCAAATCAAGCAATTATTCCAGAAG

>Intergenic_Region|79194..79293|Liberibacter_crescens_BT-1_EZ-Tn5_Transposon_Disruption

GCACCCGACAGGATTCGAACCTGTGACCTTTGGAATCGGAATCCAACACTCTATCCAGCTGAGCTACGGGTGCTGATTCTTATAAATAGAAAAATATTTT

>Intergenic_Region|79202..79103|Liberibacter_crescens_BT-1_EZ-Tn5_Transposon_Disruption

GTCGGGTGCGCCACAACATCTAGACAAAATAAGTTTGCAAAAATATTTTTTCCCTTTGGGAATAAATTGAGAAAGAAAACTACCGCTCCAGAAACAATAA

>B488_05540|597023..596972|Liberibacter_crescens_BT-1_EZ-Tn5_Transposon_Disruption

CTCTTGACTACCCTTTTGATCTTCTGTCTTATTTACCCCCTTCAGCCGCTCTCCCATACAAGGGGGCGCCACAGCCACCAACTCCACTTTTGTGGCTCAA

>B488_05540|600884..600785|Liberibacter_crescens_BT-1_EZ-Tn5_Transposon_Disruption

AGTCAAGAGCGTATAATAAACAATTGTGCCCCATCGATTGGTATTCAGGAAGAGATTGCTAAGACAAACCGGGATAAAGAAGAATAAGAGAGCAACTGTC

>B488_01990|236695..236794|Liberibacter_crescens_BT-1_EZ-Tn5_Transposon_Disruption

GGTCATATCTGAAAACATTGCTAATGCTCGTTCTACAGGAAGCTCTCCGGGATCTGATCCTTATCGACGAAAAACTATTTCTTTTGGACAAATTATGAAT

>B488_05100|553253..553154|Liberibacter_crescens_BT-1_EZ-Tn5_Transposon_Disruption

TCACGGTACAGCTCAACCATAACGACAGGCTCTACAGGTGCAGAAGATTTAGTCCTGCCATTCCCATTCTCCTTGCCAGTGACATCAGCATTTTCTGTAT

>B488_04410|494625..494559|Liberibacter_crescens_BT-1_EZ-Tn5_Transposon_Disruption

ATTGAATGCCGAACGGCATGCTCTAAAGAAATAAAGAAATTTTTATCAGGATAAACAGAAATTGATCCTTCAACTCAGCAAAAGTTCCAAGAGAATAGAA

>B488_03250|374984..374885|Liberibacter_crescens_BT-1_EZ-Tn5_Transposon_Disruption

ACATAGGCTTCTGGAGCCGGTAAAATATTGCACGTAGGACGACCACTCGGACCATAACCTTCATCCACTATTAATGAGCGATCTATCGCTATGGATAATG

>B488_05190|562434..562336|Liberibacter_crescens_BT-1_EZ-Tn5_Transposon_Disruption

ACATTAAGGTTGCCAGTAATATTAACAGTTCCCTTCTGACATATCCAGCTTCGTTTCTATTGTTTTGCCATCGGGTGAGCGTTTCTCAATCACAAGAAAG

>B488_06540|723806..723767|Liberibacter_crescens_BT-1_EZ-Tn5_Transposon_Disruption

CCTTTTTATACTTGCATAAAGTGCATACTTTTATCCTTTAACTCAACCAAAGTTTCCCCACTTCAGACTCTTCGCCTCGATTCATCCTTCCACCAAAAAA

>B488_09870|1074947..1074848|Liberibacter_crescens_BT-1_EZ-Tn5_Transposon_Disruption

ATTGGCACCACTAGCAAGATTTACTGCAAAACCAGAAGCAGGGCGTCCGTTATAAAAGGAATTGTTTCCGTAAGTTTCTTGTCCTATTTCAATTCTTGCA

>B488_10010|1095127..1095226|Liberibacter_crescens_BT-1_EZ-Tn5_Transposon_Disruption

CATATAGGGTGTGATATTAATCTAGAATGTAAAGGTCTGAAGTATATTGATCGACGTATAAAAATTATTGCTGGTGATTGTAACATTGAAGATTTACATT

>B488_00280|39769..39868|Liberibacter_crescens_BT-1_EZ-Tn5_Transposon_Disruption

CTTTTGTTCCCGCTCTTTAATTTTCTGTAGTGAAATAATTTTTGATTGAAAAGGAGTGATTGATTGACAAGTAAGGCGGTTGCTGTTGAAAATGATATAA

>B488_03730|430187..430088|Liberibacter_crescens_BT-1_EZ-Tn5_Transposon_Disruption

GGATTAATGCATGTCATGCAGATCAGCATGAGTATGCAGTCCTTGCGATATAATCATATCTTCTAATTCGAAGTCAGTATCAAGATTTTCTTCATAAGAA

>B488_05410|585307..585208|Liberibacter_crescens_BT-1_EZ-Tn5_Transposon_Disruption

GGGATGGACGCAAGGGGTGGTTTGAAAAAGGCGATAGCGTCATGTGGAAATCTCTGGGAGAATGTATGAAGGACATGGATCCTAAGCTCGAGGATATGAT

>B488_06540|723399..723498|Liberibacter_crescens_BT-1_EZ-Tn5_Transposon_Disruption

CTGAAGAGCTATCTGCTTTTTATAATGTAAAGCGCAACAATCATCTCACGACTGATTGGAGACGAAAGAAAAGTGTGATGCAAGAGAAGTTAGTCGAAGC

>B488_13270|1441126..1441226|Liberibacter_crescens_BT-1_EZ-Tn5_Transposon_Disruption

GGTTGGGGAACACTGGAACCATATTTGCTTGTAATCATGTTAATGTAACACCAGATATCTTATGTATCTCTAAAGGTATTACAGGCGGAGCGCTTCCTTT

>Intergenic_Region|329143..329044|Liberibacter_crescens_BT-1_EZ-Tn5_Transposon_Disruption

CTAGTACTCCTCGGTATTCGGAGTTTGGTTAGGAGCAGTAAGGCGGTGAGCCCCCATAGCCTATCCAGTGCTCTACCCCCGAGGGTATTCAGTTAACGCT

>B488_09870|1074296..1074197|Liberibacter_crescens_BT-1_EZ-Tn5_Transposon_Disruption

GGACTCCATGTATATGAGGTGCTCAAGATTATCAAGCGAATCCTCAATGATTTTGGTTACCGATTTCTCTACTGTTTCTGCGCTAGCACCATTATATTGA

>B488_07830|861187..861088|Liberibacter_crescens_BT-1_EZ-Tn5_Transposon_Disruption

CTGCCGGTCTGGATGTTCGCACGGCTCTCGGGATGCATGGTGTGCCGATGGTTGCCGATAAATCCTTTGCCATGCTTGATTATCTTGATCGTGAACTGAT

>B488_07290|813155..813058|Liberibacter_crescens_BT-1_EZ-Tn5_Transposon_Disruption

CTTTATGATCATCAGTATTTTCGCGCTACATGAGTCGTAGATAACATTAAAAGTTGTTGGTATCAATTTTCCTACAACTGTAAAAGGTGCTTTCTGTGAG

>Intergenic_Region|470204..470292|Liberibacter_crescens_BT-1_EZ-Tn5_Transposon_Disruption

GTGTAGTGATTTATAAAAACAGCTATTAAATATAATTCTCAGTCAAACCTAACGTTTTCCTTGTATTTTTTTTACAAAGCTTGATCCTTCAACTCAGCAA

>B488_09800|1060174..1060273|Liberibacter_crescens_BT-1_EZ-Tn5_Transposon_Disruption

CCTCCACCCATTGTAAACCGATCTCCAATTGGAACCAAACCAATAACTGGTTGATGAAGCTCTTCAATAAGTTTCATATCAGAAAAGATATCAGTATCTC

>B488_03970|459552..459651|Liberibacter_crescens_BT-1_EZ-Tn5_Transposon_Disruption

GTCAAGGATTGCTCCGATATCACAGGAAAGAGGGCTTTTATTGCAGATTAATGTCTGACATTTTTTTGACGACGACAGGGTTTCCGGAAGCTCCGTTATA

>B488_05430|587037..587134|Liberibacter_crescens_BT-1_EZ-Tn5_Transposon_Disruption

ATTAATAAGAGCACCATCAAAAGGTTTCTTAAAACATTTATGATAGGGCGGCATACTTCCCAGGATGGAATGATAGGTAAGCGTTCTCTATCATCGTCCT

>B488_05540|600884..600785|Liberibacter_crescens_BT-1_EZ-Tn5_Transposon_Disruption

AGTCAAGAGCGTATAATAAACAATTGTGCCCCATCGATTGGTATTCAGGAAGAGATTGCTAAGACAAACCGGGATAAAGAAGAATAAGAGAGCAACTGTC

>B488_12840|1400230..1400131|Liberibacter_crescens_BT-1_EZ-Tn5_Transposon_Disruption

ATCTTTATCTTTACAAGAAGACAGACGGGTATCAACGGTTGTTAATTTGTTGGATATTGAGGGTGAGGATCAGGTTACTCTTAAGATTACTGTTGCAGAA

>B488_00790|96722..96821|Liberibacter_crescens_BT-1_EZ-Tn5_Transposon_Disruption

TGGTAATACAGCAGGTGATAATTTATTGTGTGAATTTGCTTCTAGGGTTTGTGCGGTAGCCTGTTTTGATGATATCATTTTTCGTTATGAAGAGGATGGA

>B488_08300|906610..906709|Liberibacter_crescens_BT-1_EZ-Tn5_Transposon_Disruption

GTCTCTAATATGACTGTTGTTCTGAATAAAAAAACAATATTTAAAAATCTTGATCTTCAGGTATACCGGGGTGAAATTATTGGCATCATAGGAGCTTCCG

>B488_05690|621401..621500|Liberibacter_crescens_BT-1_EZ-Tn5_Transposon_Disruption

ATTTATGGGCTCAACCAATTATTTTCTAAAGTTCCTTTAATCGGTACCATTCTTGGAAATGGTCAGGATCATGGTCTCATAGGGATAACATTTAAGTTAT

>B488_04500|502668..502569|Liberibacter_crescens_BT-1_EZ-Tn5_Transposon_Disruption

CTTGTTGATACCATTATTCTGATCTTTTCAGTATCTGTCATTAATTTTGTAAAATTTTCACCAGAATTATAGCTATCAAAAAAAGATGGAGATAATTTCA

>B488_04360|490301..490400|Liberibacter_crescens_BT-1_EZ-Tn5_Transposon_Disruption

TGCTAATCCTGTGCGATCTGCTCCTCCTTTGCAATGTATAAGTAGGGGTTTTGGAGAATTTTTCATCATTTCTATAAGTGTATTTATTTCTTTTGTTGTT

>Intergenic_Region|433538..433452|Liberibacter_crescens_BT-1_EZ-Tn5_Transposon_Disruption

GCTCCTTAGGAGTTCCAGAAGAACCATAATTCAGAGGTTTCTCAGGGTTAAAACCATGCTGCCCTTCATTTTCATCAGGACGAGGAGGAAGATGATTGAG

>B488_03250|375308..375407|Liberibacter_crescens_BT-1_EZ-Tn5_Transposon_Disruption

ACCCTGAAGTATATATGCAAGTATGGACTTGTGAAAAGATTCCTTCTCCTGCAACTTCTTGGCACGGTGAAAATATTTCCCGTTATTGTAATCAGCGATA

>B488_01190|140426..140525|Liberibacter_crescens_BT-1_EZ-Tn5_Transposon_Disruption

CTTATATGTGACTAGAGGGCTGCTTGCTCTTTGTAATGATGCATCAGAAGTTGCTGCGGTTTTGGCCCATGAAATGGCACATATAATTCTTAATCATGGT

>B488_10010|1095167..1095073|Liberibacter_crescens_BT-1_EZ-Tn5_Transposon_Disruption

GTTGTTTCCGACCTTTACATTCTAGATTATTATCACACCCTATATGCACTGTGCTTTTCTTACAAACCTTGCCCATATTTCTAGAGATCCCCCATTTTGC

>B488_04930|541660..541759|Liberibacter_crescens_BT-1_EZ-Tn5_Transposon_Disruption

CTGTTATTCAGGGTTAGAGCAGAATCCTTGAATAAGGAAAGCCCATTTTCTGAAAACTATTTTTTAAAAAAACATATTATTTTTTATTTTTTAAAGAAGA

>B488_07760|855549..855648|Liberibacter_crescens_BT-1_EZ-Tn5_Transposon_Disruption

TCCTTGTTCTGAAGGACTGTTGGCAAAGCTGTTGACCAAACCACGCATTGGTGCCGGAAAAAATACCGGAAAGGCTGTTTGTACAGTATTTGTTCGGTTA

>Intergenic_Region|470204..470292|Liberibacter_crescens_BT-1_EZ-Tn5_Transposon_Disruption

GTGTACTGATTTATAAAAACAGCTATTAAATATAATTCTCAGTCAAACCTAACGTTTTCCTTGTATTTTTTTTACAAAGCTTGATCCTTCAACTCAGCAA

>B488_11700|1276344..1276443|Liberibacter_crescens_BT-1_EZ-Tn5_Transposon_Disruption

ATCTAAGCCTTGAGAAAAAGAGCTTATGCAAATCAATTCAAAGAAATCTTCTATCAAGATATCATTAAAAGTTGATAACTTGGCTAAAGGAAGAATTGAC

>B488_05410|585307..585208|Liberibacter_crescens_BT-1_EZ-Tn5_Transposon_Disruption

GGGATGGACGCAAGGGGTGGTTTGAAAAAGGCGATAGCGTCATGTGGAAATCTCTGGGAGAATGTATGAAGGACATGGATCCTAAGCTCGAGGATATGAT

>B488_00280|39769..39868|Liberibacter_crescens_BT-1_EZ-Tn5_Transposon_Disruption

CTTTTGTTCCCGCTCTTTAATTTTCTGTAGTGAAATAATTTTTGATTGAAAAGGAGTGATTGATTGACAAGTAAGGCGGTTGCTGTTGAAAATGATATAA

>Intergenic_Region|470204..470292|Liberibacter_crescens_BT-1_EZ-Tn5_Transposon_Disruption

GTGTAGTCCTTTATAAAAACAGCTATTAAATATAATTCTCAGTCAAACCTAACGTTTTCCTTGTATTTTTTTTACAAATCTTGATCCTTCATCTCATCTT

>B488_11630|1266962..1266863|Liberibacter_crescens_BT-1_EZ-Tn5_Transposon_Disruption

GCTTTGGGAGGTGCAGGAGGCGCATTATTCAGTAAAATGTCAGAGAAAAATCGTCAGTGTTATGAAAGAGATCGTTATAATCGCCGTTATTTGGTTCCTT

>B488_11630|1266962..1266863|Liberibacter_crescens_BT-1_EZ-Tn5_Transposon_Disruption

GCTTTGGGAGGTGCAGGAGGCGCATTATTCAGTAAAATGTCAGAGAAAAATCGTCAGTGTTATGAAAGAGATCGTTATAATCGCCGTTATTTGGTTCCTT

>B488_01910|231453..231354|Liberibacter_crescens_BT-1_EZ-Tn5_Transposon_Disruption

AGTCAAAGATGTGACAAGCTGCTTTTTTTTCTGAGCATCATAATAATCAATCCAAACCCCAAGTTTGTCGTTCTGTAAATCAAAACGAAACTTATAATTC

>B488_07630|846539..846440|Liberibacter_crescens_BT-1_EZ-Tn5_Transposon_Disruption

GGCGTATGGAGTGTAAAATCATTGTCCATTCCAAGATTAATAGCGGTTTGAAGCGCTTGTATGGTCTCATTACGGCAATCCGGATAACCGGAGTAATCTG

>B488_07630|846539..846440|Liberibacter_crescens_BT-1_EZ-Tn5_Transposon_Disruption

GGCGTATGGAGTGTAAAATCATTGTCCATTCCAAGATTAATAGCGGTTTGAAGCGCTTGTATGGTCTCATTACGGCAATCCGGATAACCGGAGTAATCTG

>B488_00790|96722..96794|Liberibacter_crescens_BT-1_EZ-Tn5_Transposon_Disruption

TGGTAATACAGCAGGTGATAATTTATTGTGTGAATTTGCTTCTAGGGTTTGTGCGGTAGCCTGTTTTGATGATCCTTCAACTCACCAAAAGTTCCACAAA

>B488_00280|39798..39868|Liberibacter_crescens_BT-1_EZ-Tn5_Transposon_Disruption

GTGCTGATCCTTCAACTCAGCTTAAGTTCGTGAGATAATTTTTGATTGAAAAGGAGTGATTGATTGACAAGTAAGGCGGTTGCTGTTGAAAAAGATATAA

>B488_12400|1356455..1356356|Liberibacter_crescens_BT-1_EZ-Tn5_Transposon_Disruption

GCGTATTATTGGTGCATCGCATGCTGGTTGGCGAGGAGTTTTATCTGGTATACTTGAAAATACTGTAGAAAAGATAGTAAGTCTTGGTGCCATTCAGGAA

>B488_12400|1356455..1356356|Liberibacter_crescens_BT-1_EZ-Tn5_Transposon_Disruption

GCGTATTATTGGTGCATCGCATGCTGGTTGGCGAGGAGTTTTATCTGGTATACTTGAAAATACTGTAGAAAAGATAGTAAGTCTTGGTGCCATTCAGGAA

>B488_08300|906610..906709|Liberibacter_crescens_BT-1_EZ-Tn5_Transposon_Disruption

GTCTCTAATATGACTGTTGTTCTGAATAAAAAAACAATATTTAAAAATCTTGATCTTCAGGTATACCGGGGTGAAATTATTGGCATCATAGGAGCTTCCG

>Intergenic_Region|39769..39869|Liberibacter_crescens_BT-1_EZ-Tn5_Transposon_Disruption

CTTTTGTTATTGCACTTTAATTTTCTGTAGTGAAATAATTTTGATTGAAAAGGAGTGATTGATTGACAAGGATGGCGGTTGCTGTTGAAAATGATATAAG

>B488_11970|1306976..1306877|Liberibacter_crescens_BT-1_EZ-Tn5_Transposon_Disruption

GATGAAGCATTTCTATGAGAACCTCTTTTTTCTCTAGATCATCAAATAAAAGTCGTAATCGCTCTAGATCTGTTGGTTCAATTCCTTCTAACAGGTTCGC

>B488_08210|894093..893994|Liberibacter_crescens_BT-1_EZ-Tn5_Transposon_Disruption

GAATAAAGTTGTCTGGAGTGTTAAAATAAGATGTGAACGTTTCTTTAAGAAGATTTTCTGTGACAGTGTCTGGTTTAATTCCCTTTGCTTCCAGCATAAA

>B488_09010|976269..976170|Liberibacter_crescens_BT-1_EZ-Tn5_Transposon_Disruption

AGGTAAGTTTCTAAAATGGCTGCTTCATTAAAACGAACAAAGCAGCCATTATATTTTGGTTTATTTACATTCTGGAAGACTAAGAAGATTATTACTGAGA

>Intergenic_Region|470204..470292|Liberibacter_crescens_BT-1_EZ-Tn5_Transposon_Disruption

GTGTAGTGATTTAAAAAAACAGCTATTAAATATAATTCTCAGTCAAACCTAACGTTTTCCTTGTATTTTTTTTACAAAGCTTGATCCTTCAACTCAGCAA

>B488_10010|1095127..1095226|Liberibacter_crescens_BT-1_EZ-Tn5_Transposon_Disruption

CATATAGGGTGTGATATTAATCTAGAATGTAAAGGTCTGAAGTATATTGATCGACGTATAAAAATTATTGCTGGTGATTGTAACATTGAAGATTTACATT

>B488_13570|1477793..1477892|Liberibacter_crescens_BT-1_EZ-Tn5_Transposon_Disruption

GCCTACTCATCTTCCTGTGTATAAAGAATTGTCCAATGGAGATGGATCTATTTACCCAGAAGTTACTAATTCTTTGGCAGGAGTTCGCGGAAAAACTGTA

>B488_13570|1477793..1477892|Liberibacter_crescens_BT-1_EZ-Tn5_Transposon_Disruption

GCCTACTCATCTTCCTGTGTATAAAGAATTGTCCAATGGAGATGGATCTATTTACCCAGAAGTTACTAATTCTTTGGCAGGAGTTCGCGGAAAAACTGTA

>B488_09580|1031694..1031595|Liberibacter_crescens_BT-1_EZ-Tn5_Transposon_Disruption

ATTCCTACTCCTGCAATAAGGCGATTCCGACGTGGAAAATCACGGGCACGTGGTATCCAACCAAAAGGCATCTCACTGATTGCTAAACCTACACCATTCC

>B488_09580|1031694..1031595|Liberibacter_crescens_BT-1_EZ-Tn5_Transposon_Disruption

ATTCCTACTCCGGGGTAAGGCGATTCCTACGTGGAAAATCACGGGCACGTGGTATCCTACCAATAGGCATCTCACTGATTGCTAAACCTACACCATTCCG

>B488_09580|1031509..1031584|Liberibacter_crescens_BT-1_EZ-Tn5_Transposon_Disruption

GTATACGATTGCTCCTCCAGCTCACCTTAATTTCAGGACAATCGTTGCAATGGCAGGTGGTTTAGATTGTTCATATCCACCAGAAAACATCAGTCTTTTA

>Intergenic_Region|738575..738476|Liberibacter_crescens_BT-1_EZ-Tn5_Transposon_Disruption

GGTTATAATTATAGACAAAATCCAGAAACTTAAAATATAACGAGTTGTAGGCTTTATATTATAAAAAGCCTACAACTCAATAAAATCATAATATAACGTT

>B488_09580|1031493..1031586|Liberibacter_crescens_BT-1_EZ-Tn5_Transposon_Disruption

AGAGCCCCTGCGCATCAAGCAAGCCTTAATAGCGGGACAATCGTTGCTTGGCAGGTGGTTTACATTGTTCATATCCACCAGAAAACATCAGTCTTTTAGA

>B488_05430|587037..587134|Liberibacter_crescens_BT-1_EZ-Tn5_Transposon_Disruption

ATTAATAAGAGCACCATCAAAAGGTTTCTTAAAACATTTATGATAGGGCGGCATACTTCCCAGGATGGAATGATAGGTAAGCGTTCTCTATCATCGTCCT

>B488_08570|940057..939957|Liberibacter_crescens_BT-1_EZ-Tn5_Transposon_Disruption

GCTTTACAGGCTTCTGAATCTAACGATAAGCTTCTCAGGGGAAGATATCGGATTTAGGTCTTCTACTTAATATTGCTCTTGCTCAGCGGGTTCAGGAATT

>B488_08570|940057..939958|Liberibacter_crescens_BT-1_EZ-Tn5_Transposon_Disruption

GCTTTACAGGCTTCTGAATCTAAGGATAAGCAAAATCAGGGGAAGATATCTGATTTAGGTCGTCAACTTAATATTGCTCTTGCTCAGCGGGTTCAGGAAT

>Intergenic_Region|1446866..1446965|Liberibacter_crescens_BT-1_EZ-Tn5_Transposon_Disruption

GCTCTATACTCACTTAGAGAATATAATTTCTCTACGATACAAAGTCACAAATGCCTTATTTTTTACATAGACATCACATGTTTATCTTATAAGTTATCGT

>B488_03250|374984..374885|Liberibacter_crescens_BT-1_EZ-Tn5_Transposon_Disruption

ACATAGGCTTCTGGAGCCGGTAAAATATTGCACGTAGGACGACCACTCGGACCATAACCTTCATCCACTATTAATGAGCGATCTATCGCTATGGATAATG

>B488_03250|374984..374885|Liberibacter_crescens_BT-1_EZ-Tn5_Transposon_Disruption

ACATAGGCTTCTGGAGCCGGTAAAATATTGCACGTAGGACGACCACTCGGACCATAACCTTCATCCACTATTAATGAGCGATCTATCGCTATGGATAATG

>B488_07030|780032..780131|Liberibacter_crescens_BT-1_EZ-Tn5_Transposon_Disruption

ATACATAAGATACCGCTTTGTGCAAAATATGGTTGGATATCAAAATGACAGAGTTCGCAGGTTCGGCTGCATCACAAGCTGATTTTATCTGGAAGAATGC

>Intergenic_Region|1155633..1155732|Liberibacter_crescens_BT-1_EZ-Tn5_Transposon_Disruption

ACATAACTCATTATGAACCTTCATATCAAAAAAAGTTCCAGGTGCAATTGATAATTAAAAAAAATTAACGATCTTTAACATAGTCTTTCACAAATGTTGC

>B488_07290|813106..813055|Liberibacter_crescens_BT-1_EZ-Tn5_Transposon_Disruption

ACATAACTCATTATGATCCTTCCGTCAGCGAAGTTCCATGAGCTTATGAGTTGTTGGTATCAATTTTCCTACAACTGTAAAAGGTGCTTTCTGTGAGGAT

>B488_07830|861187..861088|Liberibacter_crescens_BT-1_EZ-Tn5_Transposon_Disruption

CTGCCGGTCTGGATGTTCCCACGGCTCTCGGGATGCATGGTGTGCCGATGGTTGCCGATAAATCCTTTGCCATGCTTGATTATCTTGATCGTGAACTGAT

>B488_05870|646022..646121|Liberibacter_crescens_BT-1_EZ-Tn5_Transposon_Disruption

GGTGAATACCTTGCGAATCGCTGTAATAGGCCATTGTGGAAGAAGAAATTTTATCCATGTTCCAGTGCTGATTACTGCTTTGTGTCCCCGAAAACGACCA

>Intergenic_Region|470204..470292|Liberibacter_crescens_BT-1_EZ-Tn5_Transposon_Disruption

GTGTAGTGCTTTATAAAAACAGCTATTAAATATAATTCTCAGTCAAACCTAACGTTTTCCTTGTATTTTTTTTACAAAGCTTGATCCTTCACCTCAGCAA

>B488_08250|898290..898389|Liberibacter_crescens_BT-1_EZ-Tn5_Transposon_Disruption

ATTATGGGTTCCCCATCGACCTCATCGTCAAGGACAAAAATCAGAAGGCGGGATTCCACTGCGTATGCATACCCATTACAAACCTTCAGGAGATCAGCCA

>Intergenic_Region|431490..431391|Liberibacter_crescens_BT-1_EZ-Tn5_Transposon_Disruption

TTTTAAGATATCCCTTAAAATCAAAATTGATCTTCCTATGCTCTTTATGACCATAGTTATTATAATGCCAAATACCATAATCAGAAATGGCTTGTTTAGT

>B488_03770|435762..435664|Liberibacter_crescens_BT-1_EZ-Tn5_Transposon_Disruption

CTTATGAGTCGGTATTAAAATACTCGTTATAGATGAAGCAGATCGAATGCTTGATATGGGCTTTATTCCTGATATTGAAAATATTGTTAGAATTATATCA

>B488_12270|1340333..1340235|Liberibacter_crescens_BT-1_EZ-Tn5_Transposon_Disruption

ATTCGTGCACGTTGTGGTGGTCATCAAGGTCATGTATTTGAGGATGGGCCCCAGCCTACAGGTTTACGCTATTGCAATAATGGTGCAGTGTTAACTTTTA

>B488_08300|907231..907301|Liberibacter_crescens_BT-1_EZ-Tn5_Transposon_Disruption

GTCTAGGATTGATCCTTCATCTCAGCAAAAGTTATTATTATACCATTATTGCGCAAGGAGGCATTCAGGATATATTAAATGTTGATGAACCTTCAATACA

>B488_03240|372478..372577|Liberibacter_crescens_BT-1_EZ-Tn5_Transposon_Disruption

GATTAAGAGATGCAAGTGGATTTTGGAATACCATTTGTATGGAACGACGTATTGTACGTAATGATGAGCGATCAAGGGTTAGTATGTTACATCCATTTAT

>B488_11960|1305881..1305980|Liberibacter_crescens_BT-1_EZ-Tn5_Transposon_Disruption

TTGGAATACTGACGAATAAAAGACACTTCACGCATTTGATCTTTTCTTCTACCTGATAAACGCATAATTTACTCCTCAGTAAAGTCTTGAATATTTGTAA

>B488_13130|1425532..1425631|Liberibacter_crescens_BT-1_EZ-Tn5_Transposon_Disruption

GTGTTGCTGTATGCGAACAAACAGAAGATCCTGCAGAAGCCAAAAAACGTGGTGCAAAATCAGTTGTACGTCGTGATGTAGTCCGTCTTGTAACCCCCGG

>B488_13130|1425532..1425631|Liberibacter_crescens_BT-1_EZ-Tn5_Transposon_Disruption

GTGTTGCTGTATGCGAACAAACAGAAGATCCTGCAGAAGCCAAAAAACGTGGTGCAAAATCAGTTGTACGTCGTGATGTAGTCCGTCTTGTAACCCCCGG

>B488_01800|221385..221287|Liberibacter_crescens_BT-1_EZ-Tn5_Transposon_Disruption

CTATATATATTCATCACCTTGTACTGTAAGCAGCAATCTCACGATCTGTTAAATTTCTATTTGACATAGTACGTCGTTTAGAATATTCCAGAAAATCTAT

>B488_04720|528638..528736|Liberibacter_crescens_BT-1_EZ-Tn5_Transposon_Disruption

GTTCTTGGGATACCTAAGAACCCTCTGAAGTGCACGTTTATTGTTAATCTTGAAGCAAGAGTTAATGGCGGGAAGATAGTTATTTATCGTATTTTGGAGG

>B488_11260|1226085..1226184|Liberibacter_crescens_BT-1_EZ-Tn5_Transposon_Disruption

CTGGTAAGCCCTGATATATAACTTTTCTTACCTCAACTATATAAACCCCAGCAAATCCAGGGCACAGTATACGCCCAACTTTTTCAAATAAATATGGAAA

>B488_09800|1060279..1060376|Liberibacter_crescens_BT-1_EZ-Tn5_Transposon_Disruption

GTGTGATACTGCGTTGGTTCATCGTTAAATGAAGGACCAAACCATTGGCATTCCCTACAGAGTGCGATACACCATCAGAGGTTATATGCGCGGAAGAATG

>Intergenic_Region|540358..540259|Liberibacter_crescens_BT-1_EZ-Tn5_Transposon_Disruption

GTATTGTTTTATAGATAATTCTTAATTCTTCAGCTGTTAAGAATCTATTGACTTTTTTTATTTTCTTTTCATCCCACCCCTTCTTTCAATTCAAGACCAT

>Intergenic_Region|208768..208669|Liberibacter_crescens_BT-1_EZ-Tn5_Transposon_Disruption

GCCATCTACTGTTAAAAACTACACTTTAAATCTTAAATTCCTATCGATTGATTAAAAAAAGAAAGCTTGTCAAAAAATACTCTTTATTGATTAGAAACCT

>B488_06370|698701..698602|Liberibacter_crescens_BT-1_EZ-Tn5_Transposon_Disruption

GCCTTCATACGATCATAAGTTTGTTGAGATACTTCCTGTGTAAGGTCAGTACGCCGTATACGTACATCCTCAATCATCAATCCCAATTTTTCTGCATCTC

>B488_08420|919833..919932|Liberibacter_crescens_BT-1_EZ-Tn5_Transposon_Disruption

CTTTAAAGATGTAACCTTTCGGTATAATAAAAATAAAATTGGCTTGGAAAACATTAATATCTTTATTCCTGCTGGAATGAAAATTGGATTAATAGGCCGT

>B488_09990|1093684..1093585|Liberibacter_crescens_BT-1_EZ-Tn5_Transposon_Disruption

GATGAGGTCTATGGTTCCTTTGATAAAGGTTTATGCCAGGAGACAACAGTTTATAATCCATCATCTCCTTATTCTGCTTCTAAGGCACCAACTGATCATT

>B488_06100|674272..674173|Liberibacter_crescens_BT-1_EZ-Tn5_Transposon_Disruption

GGATTAACTGGCATCAGTGGTTGGTATAAAATGACACCGGAATTCCGTAAGAAAATGGGAACGATTCCAGAACTCTCACCTAAATATCCATCATTGGAAA

>B488_09900|1081147..1081048|Liberibacter_crescens_BT-1_EZ-Tn5_Transposon_Disruption

GATTTAAGACATGTCTGTGAAAGAATATATTTTTCTTTTAATCTTTTTGCTATCTCTCTCGGTGAGCGACTAGTGTTATTAGAATAACTAGTAAAATGCA

>B488_05400|582978..582895|Liberibacter_crescens_BT-1_EZ-Tn5_Transposon_Disruption

ATTCTAATGTTTGTTATGATCAGTGGCCCTTCTCTTGTTCTTTTGGCTTCCTTCAAAACAATTCTTGCATCCATGTTTCTGATCCTTCAACTCAGCAAAA

>B488_12270|1340622..1340523|Liberibacter_crescens_BT-1_EZ-Tn5_Transposon_Disruption

GCTGATATGGTAGAAAAAATGACGTTTTCTGATAAAGAGTGGAAAAGCAAACTTACAAAGCAACAATATGCTATTTTACGTCAGGAAGGGACGGAATATC

>B488_09000|686684..686783|Liberibacter_crescens_BT-1_EZ-Tn5_Transposon_Disruption

ACCTAGAACACCGCCGTATGCGCCTTCCCAAGAGAAATTACCAGAATTCGAACCCGTGGGCTGTGACGCAGCATGAGGAATATTATCTACATTATTCACG

>B488_04720|528637..528736|Liberibacter_crescens_BT-1_EZ-Tn5_Transposon_Disruption

ATTCTTGAGATACCTAAGAACCCTCTGAAGTGCACGTTTATTGTTAATCTTGAAGCAAGAGTTAATGGCGGGAAGATAGTTATTTATCGTATTTTGGAGG

>B488_01190|140426..140525|Liberibacter_crescens_BT-1_EZ-Tn5_Transposon_Disruption

CTTATATGTGACTAGAGGGCTGCTTGCTCTTTGTAATGATGCATCAGAAGTTGCTGCGGTTTTGGCCCATGAAATGGCACATATAATTCTTAATCATGGT

>B488_11180|1218588..1218489|Liberibacter_crescens_BT-1_EZ-Tn5_Transposon_Disruption

GATACATCCAGGATGATTATGACCCGTAGTTCATTGCAGGAAGCAACTGATGCTGCTACTTTAACGGCTGCTTCAGCAATTTTTAAAAAAGCAAACACTA

>B488_10540|1149236..1149335|Liberibacter_crescens_BT-1_EZ-Tn5_Transposon_Disruption

GGTTGCTGAATGGCCTACCAATTTACAAGCTTCCAGAAAAACTCATCTCTAAATTTCATGATCCTTCAACTCAGCAAAAGTTAAGAGTTATACGTGTTTC

>B488_12130|1326078..1325979|Liberibacter_crescens_BT-1_EZ-Tn5_Transposon_Disruption

GAGTAACATTACGTGTTGGGGATGTTTTTGCAACTGGCATGGAACAGATACCTTCAAGCTCAGAAGGGAATGGATTGTATAGGATTACTGTATCTCCTAA

>B488_03810|441336..441237|Liberibacter_crescens_BT-1_EZ-Tn5_Transposon_Disruption

GAACTCCTGTTGCAAGATGCGCTCCAATAATACCAGACAAAATATCTCCTGAACCAGCAGTTGCTAAATTAGAAGATGCATTGGTATTAATTAGAACACG

>B488_08260|900365..900464|Liberibacter_crescens_BT-1_EZ-Tn5_Transposon_Disruption

CCTCTGATGTTATTCACTGTCTGTATAACATGAAACACATAAGCAGGAGTAACAGATATGAATAATCTCATTCCGCATCATTTTAAAATGTTTATTGCCC

>B488_13270|1440674..1440772|Liberibacter_crescens_BT-1_EZ-Tn5_Transposon_Disruption

CTCAAGGGCTGATTAGAATTCGCTCCCCCAGGCCTTTCATATGTCTTCTTTTCTGATAGCGGCTCAACTTCAGTAGAGGTAGCTCTAAAAATGGCCCTGG

>B488_06930|771750..771849|Liberibacter_crescens_BT-1_EZ-Tn5_Transposon_Disruption

CTTCAGGATTAATATAACTAAATGCAAGTTCTTCCAGTTCTTCACGCATGTCTTGCATGCCCATGCGTCCAGCTAACGGAGCATATATATCCATCGTTTC

>B488_03730|429056..429155|Liberibacter_crescens_BT-1_EZ-Tn5_Transposon_Disruption

ACGTTATACTCTCAGTGAAGGTGATGCTCTTAAATATGTTGCCTCTTATCCGGATTTGATAGAAGATTTTTTGAGGAAAGGTCTTTCTGATAAACAAGCT

>B488_01190|140426..140525|Liberibacter_crescens_BT-1_EZ-Tn5_Transposon_Disruption

CTTATATGTGACTAGAGGGCTGCTTGCTCTTTGTAATGATGCATCAGAAGTTGCTGCGGTTTTGGCCCATGAAATGGCACATATAATTCTTAATCATGGT

>B488_10700|1167361..1167413|Liberibacter_crescens_BT-1_EZ-Tn5_Transposon_Disruption

TTCTGAAAGTGAGCGTAAACGTGATCCTTCAACTCAACAAAAGTTCCAGAAAAAGATACTGCAGAAGGTTCCATAATTTCAATAATTTCTTTCATTATCA

>B488_06930|771533..771630|Liberibacter_crescens_BT-1_EZ-Tn5_Transposon_Disruption

ATACGAAAACCATAGACATCACAAAGTTGTTCAAATGATAATGATTTCGATTGCATTTTGCAGAAAATAGAATAAGCCCTCTTTTGTGATCCTTCAACTC

>B488_01810|222137..222236|Liberibacter_crescens_BT-1_EZ-Tn5_Transposon_Disruption

ACCCGGTATCTGAGAAAAGTTGTCTTTTAATGATAGTGTAATAAAGATACTAATAACAATTATTTTTAAGAATATTTTTGTAAAATCAATGAGATTATTA

>B488_12280|1342344..1342443|Liberibacter_crescens_BT-1_EZ-Tn5_Transposon_Disruption

GATCTATTCTGGAGGACACAGAATTGGTTGTCACCTATTCCAGCATTATGATATGACAACATTAAGTGACAGGAGTATAAGAAACCAAATACAGATGTTC

>B488_10060|1100851..1100752|Liberibacter_crescens_BT-1_EZ-Tn5_Transposon_Disruption

GGTTATGATGCTAAACAGATAGGGAAATATTGCAGTTCTTTTCCAGATGATTATTATGAAATTACAGATTACAATCAAATATCTAAAGTTATGCATGACA

>B488_07840|862636..862670|Liberibacter_crescens_BT-1_EZ-Tn5_Transposon_Disruption

CCCCGATACTGACTGGCCAGCTGTTTCCCTGATCCTTCAACTCAGCAAAAGTTCCAGCGCCGCCGGTCCGGGGGGCCGCTCTCCCTAGTCTTGGGGATTT

>Intergenic_Region|531982..531883|Liberibacter_crescens_BT-1_EZ-Tn5_Transposon_Disruption

CTCTTGTACAACACAAAAACCAAAAAACTATAAAACAGCAATACCCATAGTCCCCTGTTGCAAAAAGCCATAAACTGGAGACGGGAGATGACTTACTGTA

>B488_05390|582619..582520|Liberibacter_crescens_BT-1_EZ-Tn5_Transposon_Disruption

GGCCTTAAGTACACTCTTGATATTTTAATAGTGCTATGTTTTTACAATAAAACATTACATATTTGAATAAAAAGGCCCTTCCCCTCCTTGCGGTGGCACC

>B488_05200|562785..562884|Liberibacter_crescens_BT-1_EZ-Tn5_Transposon_Disruption

TTATAAAACAGTTTCCGCACTGGAAACAGTAAACCGACCCGCGTTAACGGATCGGCTTGGTGCATGATTTTTATACCACACTTATTAGGAGAGGGAAAGA

>B488_08400|916853..916785|Liberibacter_crescens_BT-1_EZ-Tn5_Transposon_Disruption

AGTATATAGGACCAGTCACGGCTCATGGAGCTTATCAATCTGATGAATAAAGGAAATAATGATTGATGGT

>Intergenic_Region|393931..393832|Liberibacter_crescens_BT-1_EZ-Tn5_Transposon_Disruption

CTATAAAGATATCCCGTGGCTGGTGAAAGAATGTGGAAAAAAATGAAAGTATTTTTCTTAAATCATTCGGTATATAAGGAAGATTAAGACAGGAAAAGCC

>B488_11700|1277278..1277377|Liberibacter_crescens_BT-1_EZ-Tn5_Transposon_Disruption

GCATTACATGCACACTTCCTGGCTTTTAATCACCCTTGCAATAATAAACGCATGAAATTTGAAGTACCTATCGAAAAAGACATGCTAAACATAATAGAAA

>Intergenic_Region|330836..330737|Liberibacter_crescens_BT-1_EZ-Tn5_Transposon_Disruption

GCTTTCAGCGGTTATCCATTCCGTATGTAGCTACCCTGCTATGCCCTTGGCAGGACAACAGGTCCACCAGAGATACGTCCATCCCGGTCCTCTCGTACTA

>B488_05630|611611..611710|Liberibacter_crescens_BT-1_EZ-Tn5_Transposon_Disruption

ATGTTGGGCAGGTTATTGCCCAAATTGATACACAGAAGATACAGGCTGATGTTCATGCTATGCGAGCAAAATTAAATCAAGCCGCAGCCAATTTGTTAAA

>B488_09990|1094125..1094026|Liberibacter_crescens_BT-1_EZ-Tn5_Transposon_Disruption

CGGTATTCCATTGCTGCCCCTCAATTTTAATTGAGGTTTATCATGCGAGTAATTATAACGGGCGGTGCAGGTTTCATTGGTTCAGCGGTATGTCGATATT

>B488_10710|1168745..1168844|Liberibacter_crescens_BT-1_EZ-Tn5_Transposon_Disruption

ATATAAAGAGCATCAATAATTTCAGGATCTACCAATGAATTTAATTTCATCCATATACTAGCTAAACATCCAGAATGAGCATATTCGATCTCATCTTGAA

>B488_05920|651019..650920|Liberibacter_crescens_BT-1_EZ-Tn5_Transposon_Disruption

GTGTGGGCATATACCTCCCTTAGAAAAACCACAGGAAACAAATGAAGTTCTACGACAATGGTTTTATCCTGTTTCACAAACATAACCGCTGACTTGGCGG

>B488_01840|224843..224744|Liberibacter_crescens_BT-1_EZ-Tn5_Transposon_Disruption

CTTCGAAGGCTCTGCTACTTTTAAGGATCTTATCCAGAAAGAAAATCAGGAACATAACGAATTTTCAGCAGCAATTAATATGGAGATAATATTAGATGGA

>B488_10710|1169260..1169359|Liberibacter_crescens_BT-1_EZ-Tn5_Transposon_Disruption

ATCATTAGAAGTTCGATATAATGTTTGCTTAATCACAAGAACATCAGGATCTTGTGCAGCTTGACGCAAAAACTGTACAACCATATCAAAAGATTCATAT

>B488_07740|852816..852915|Liberibacter_crescens_BT-1_EZ-Tn5_Transposon_Disruption

GCATACGGCTGGCCATTTCCTGCTGATAGGCTTGTGCCTGAGCAGCATTATGACTCATCATCCGCTGCGCATTGCGATTCTCGATACGGTCCTGTTCCTG

>B488_05410|583713..583614|Liberibacter_crescens_BT-1_EZ-Tn5_Transposon_Disruption

GTTCCATGATGATGCACTGGTTGCATAAGAGCTCTTAGATCTCACTCAAGGAGGGCTTTCTTATTCAAAGAGAGCCTTCCTTAAGGCTCAATCACTGCAC

>B488_09990|1093684..1093585|Liberibacter_crescens_BT-1_EZ-Tn5_Transposon_Disruption

GATGAGGTCTATGGTTCCTTTGATAAAGGTTTATGCCAGGAGACAACAGTTTATAATCCATCATCTCCTTATTCTGCTTCTAAGGCAGCAGCTGATCATT

>B488_06290|692056..691957|Liberibacter_crescens_BT-1_EZ-Tn5_Transposon_Disruption

CTTATAGGCTTGTGCAGAAGGTTGGGTTACCGGTTTTGAGCCCGCAAGAACCAATCCTGCCTTAATCGTAAACCGGAAACGGGATTTTGATTTCTATTTC

>B488_07670|848104..848005|Liberibacter_crescens_BT-1_EZ-Tn5_Transposon_Disruption

TTGTAGCGCTTTGGCATCAATTTGTTAAAAAAGATTCCTTGTTAAAACGTATGTGGTTTTAATCGTGGAATAATAGCTTAAAAAGCGATTGTATGATCTC

>B488_11970|1306251..1306152|Liberibacter_crescens_BT-1_EZ-Tn5_Transposon_Disruption

GATAATAGTCGTGATAGGTTACGTGATCCTAAAGGTTCACCTGTTTCCAAATAACTTTCAACTATTCGACGAAAAACTTCTCGATCACGATCGTCTAATG

>B488_11960|1305881..1305980|Liberibacter_crescens_BT-1_EZ-Tn5_Transposon_Disruption

TTGGAATACTGACGAATAAAAGACACTTCACGCATTTGATCTTTTCTTCTACCTGATAAACGCATAATTTACTCCTCAGTAAAGTCTTGAATATTTGTAA

>B488_01190|140579..140480|Liberibacter_crescens_BT-1_EZ-Tn5_Transposon_Disruption

ATCTGACATAGCCAATTGATTAAAGCTTTTTTGTTGTTCTTGTTGACGCTCAACACCATGATTAAGAATTATATGTGCCATTTCATGGGCCAAAACCGCA

>B488_10710|1168544..1168642|Liberibacter_crescens_BT-1_EZ-Tn5_Transposon_Disruption

CATATCAGCAGACCCTATATAGGCAATAGCCTCTTCAGAAGGCAAACCATAACCGTTACCAAAACAAAAAATACGACTATGTTCGAGGAAACGACCCACT

>B488_04870|537714..537615|Liberibacter_crescens_BT-1_EZ-Tn5_Transposon_Disruption

CTTCTGTACCGATAAAGACAAAGACAAGCACAGCTCCAACAATCTCAAAAAACAACATCGGCTTCTCCTCCATTATCCTGTCAAACCAGTCTCTTAAAAA

>Intergenic_Region|502210..502111|Liberibacter_crescens_BT-1_EZ-Tn5_Transposon_Disruption

ATGTTATAATAATCAATCTTGTTATTAAACTCGTTTTCATGTACTTATGAAATTCTTAACTTCAGGCACAAAAACTGTCTGAAGTTTTATTTCATATGCT

>B488_11180|1218588..1218489|Liberibacter_crescens_BT-1_EZ-Tn5_Transposon_Disruption

GATACATCCAGGATGATTATGACCCGTAGTTCATTGCAGGAAGCAACTGATGCTGCTACTTTAACGGCTGCTTCAGCAATTTTTAAAAAAGCAAACACTA

>B488_05380|580675..580576|Liberibacter_crescens_BT-1_EZ-Tn5_Transposon_Disruption

CTTTTATATATTCCAAAACGATGCTTTAGCGAATAATCAAATAATTGGTGTCGTTGCCCCTAAAAATGGACAATATGCAGTTCTTGGTAAACAAATTTTT

>B488_05090|552208..552109|Liberibacter_crescens_BT-1_EZ-Tn5_Transposon_Disruption

CCCTTATCCAGCTCCTCACGGTTTGTGTTCGACATGGAAGTGATAAAAGTTTGTAATTGTGTTGCCATTTCATTTCTCCTTTATGAGGCATTTTTTTCTA

>B488_11960|1305881..1305980|Liberibacter_crescens_BT-1_EZ-Tn5_Transposon_Disruption

TTGGAATACTGACGAATAAAAGACACTTCACGCATTTGATCTTTTCTTCTACCTGATAAACGCATAATTTACTCCTCAGTAAAGTCTTGAATATTTGTAA

>B488_07060|786591..786503|Liberibacter_crescens_BT-1_EZ-Tn5_Transposon_Disruption

GTGCTTGAAAGGGCGCCACATATCCGTGCAAACCCACTCTACCTTTTCACGGTCAGGCAGCGCCCTAAAGAACGGCTTCAGATGATCCTTCAACCAGCAA

>B488_09000|686684..686783|Liberibacter_crescens_BT-1_EZ-Tn5_Transposon_Disruption

ACCTAGAACACCGCCGTATGCGCCTTCCCAAGAGAAATTACCAGAATTCGAACCCGTGGGCTGTGACGCAGCATGAGGAATATTATCTACATTATTCACG

>B488_05910|650520..650421|Liberibacter_crescens_BT-1_EZ-Tn5_Transposon_Disruption

CGTCACTATGCTATAAGGTGGAACATCATGAAGAACAACACTTCCAGCTGCTATACGAGCCCCTGTACCTATTTCGATATTGCCAAGGATTATTGCAGCT

>B488_08360|913143..913240|Liberibacter_crescens_BT-1_EZ-Tn5_Transposon_Disruption

GTGCTTCAAATTGAAATTAGTTTTTTAGGGTTCCCAACGATTTAAAGAATAAGGCATCAATACGCCAACGACCCCGCCACTGGCTCCTGAAGCAATACGC

>B488_05920|651019..650920|Liberibacter_crescens_BT-1_EZ-Tn5_Transposon_Disruption

GTGTGGGCATATACCTCCCTTAGAAAAACCACAGGAAACAAATGAAGTTCTACGACAATGGTTTTATCCTGTTTCACAAACATAACCGCTGACTTGGCGG

>B488_00540|68391..68292|Liberibacter_crescens_BT-1_EZ-Tn5_Transposon_Disruption

ACGTGTAACAGAGATACATCAATTATTACCAGAAACTTCGTTTCATATTATTGGTGAGTATTTTATGCCTATTCACTTACAACTCATGGCTATAAAAGGA

>B488_07060|786304..786228|Liberibacter_crescens_BT-1_EZ-Tn5_Transposon_Disruption

GCGCACGAAAGGTCATACCAAGACACTGTTGCCGGATAACGTCCACCTCACCCCGGGTTGCCCGACGATTATGATCCTTCAAAGTCTTGAAAATTCAGTA

>B488_11960|1305881..1305980|Liberibacter_crescens_BT-1_EZ-Tn5_Transposon_Disruption

TTGGAATACTGACGAATAAAAGACACTTCACGCATTTGATCTTTTCTTCTACCTGATAAACGCATAATTTACTCCTCAGTAAAGTCTTGAATATTTGTAA

>B488_11960|1305883..1305980|Liberibacter_crescens_BT-1_EZ-Tn5_Transposon_Disruption

TAGGAATACAGACGAATAAAAGACACTTCACGCATTTGATCTTTTCTTCTACCTGATAAACGCATAATTTACTCCTCAGTAAAGTCTTGAATATTTGTAA

>B488_11960|1305881..1305980|Liberibacter_crescens_BT-1_EZ-Tn5_Transposon_Disruption

TTGGAATACTGACGAATAAAAGACACTTCACGCATTTGATCTTTTCTTCTACCTGATAAACGCATAATTTACTCCTCAGTAAAGTCTTGAATATTTGTAA

>B488_11960|1305881..1305980|Liberibacter_crescens_BT-1_EZ-Tn5_Transposon_Disruption

TTGGAATACTGACGAATAAAAGACACTTCACGCATTTGATCTTTTCTTCTACCTGATAAACGCATAATTTACTCCTCAGTAAAGTCTTGAATATTTGTAA

>B488_08600|943527..943626|Liberibacter_crescens_BT-1_EZ-Tn5_Transposon_Disruption

GATTAATCGTATTGATATAACTTCTTGGAACAACAGAAAAATTAACAAAAGGGGGAGCCATCAATGCATTATTGGCGTTAATTTGCTCTGTCACCGCAAA

>B488_03240|373665..373566|Liberibacter_crescens_BT-1_EZ-Tn5_Transposon_Disruption

TCCCTACTCTCTCTGTTCTTAACTTAACAAGTTCTCTGTATATAAAAGGAAAATGGAAGCCTATAATTCAAAATATCAATTTTGAAATTAAGCCTGGAGA

>B488_12170|1330384..1330285|Liberibacter_crescens_BT-1_EZ-Tn5_Transposon_Disruption

TGCCTATACTGGTCGTTCGCTGCAACTAGAACTTAATATGATTAAAAAATATCTGGATACAGCTCATAGGCCAATTATTGCCATTATTGGTGGTTCTAAA

>B488_13160|1429898..1429985|Liberibacter_crescens_BT-1_EZ-Tn5_Transposon_Disruption

AAGCAATACATCATCTTCAAGAATAAGAGCGGCTTCTGTCCATCAGAAACAAATTTCTCTAAGGCTGCAACATGACTATAATACAAGGGGCGTTATGAGC

>B488_06310|694068..693969|Liberibacter_crescens_BT-1_EZ-Tn5_Transposon_Disruption

ACTCTCATATCATGAAAACAGCCAATAGCAGGATAAAGATTTTTTTCTTCCTTTCGAATGAGATACGCAATCCCAGAAGGGATAGGAATAATACCCCTAT

>Intergenic_Region|670320..670417|Liberibacter_crescens_BT-1_EZ-Tn5_Transposon_Disruption

GAAATAGACTTTCCTCGCGCATAGAGACTCGAGTCTCTATTAATAGTCAAAAGGTGAGGGATAGACTTGAATTTTTATTCCGCTCCTTGAGTTCAGTTTT

>B488_05630|612155..612086|Liberibacter_crescens_BT-1_EZ-Tn5_Transposon_Disruption

ATAATGCACTGCATTAATCACAGCTGGAAAACGAATTTTTGGATAAGCTGCGACTGTAAAATGATCCTTCAACTCAGCAAAAGTTCCATGACGTCGTAGC

>B488_07750|853740..853839|Liberibacter_crescens_BT-1_EZ-Tn5_Transposon_Disruption

GCTGTGCATCGTCTTCCTCCGGGATACCATGAACCACGGCCTGACACTTCAATTCCTCGAGGGGATAACGGTACATTTCCCTGAGAACATGGCGATTCAA

>B488_05440|588135..588036|Liberibacter_crescens_BT-1_EZ-Tn5_Transposon_Disruption

CTCCTACACTACACGGATCTAAAGGACGATGATAGAGAACGGTTACCTACCATTTCATCCTGGGAAATATGCCGCCCTATCATAAATGTTTTAAGAAACC

>B488_02030|238785..238720|Liberibacter_crescens_BT-1_EZ-Tn5_Transposon_Disruption

CAACAAGACCATAACCAATCAATTGATTACTTCGAGCTGCTTGCAAAGAAGCAACATCTTTGATCCTTCAACTCAGCAAAAGTTCCAGATTTTTTGGGGT

>Intergenic_Region|345541..345640|Liberibacter_crescens_BT-1_EZ-Tn5_Transposon_Disruption

GCTTACCATAGATGGTGGTATTTATTTTCGAAATGTCTCGTAATTTGAGATATCATTTTTTTCCAGTATTTATATTGCATAGGATTTCTTGATAGGAATT

>B488_07750|853740..853839|Liberibacter_crescens_BT-1_EZ-Tn5_Transposon_Disruption

GCTGTGCATCGTCTTCATCCGGGATACGATGAATCACGGCCTGACACTTCAATTCCTCGAGGGGATAACGGTACATTTCCCTGAGAACATGGCGATTCAA

>B488_02340|267731..267830|Liberibacter_crescens_BT-1_EZ-Tn5_Transposon_Disruption

ATATGAAGCACCATGAGAGTTTTTCAAAAATTGGAGGTCTTCTTTTTGATAAAGACGGTACCCTCTTTTCATATGATACAAGTTGGCTGGTTGTTAATAA

>B488_05180|560308..560407|Liberibacter_crescens_BT-1_EZ-Tn5_Transposon_Disruption

GTTGTAAAGGGAGCCATAGCAGAGGGAAAGGCAGCTTACGAAGGACATCCGATTGATAACTTTTCCAAAGACTACCAGAGTGTTTCGCAGGGCTGGGATC

>B488_01840|224843..224744|Liberibacter_crescens_BT-1_EZ-Tn5_Transposon_Disruption

CTTCGAAGGCTCTGCTACTTTTAAGGATCTTATCCAGAAAGAAAATCAGGAACATAACGAATTTTCAGCAGCAATTAATATGGAGATAATATTAGATGGA

>B488_00930|109587..109686|Liberibacter_crescens_BT-1_EZ-Tn5_Transposon_Disruption

TGTATAGAGTTGTTTACGAGGCATACCTGTAATTCGTGCTGCAGTGCTTGCAGCTTTTGCTTTTGACATCTTCTGGTATAATGAATATAAAAGCTTGTCT

>B488_00540|68391..68292|Liberibacter_crescens_BT-1_EZ-Tn5_Transposon_Disruption

ACGTGTAGCAGAGATACATCAATTATTACCAGAAACTTCGTTTCATATTATTGGTGAGTATTTTATGCCTATTCACTTACAACTCATGGCTATAAAAGGA

>B488_09990|1093210..1093307|Liberibacter_crescens_BT-1_EZ-Tn5_Transposon_Disruption

GGCATGTCCTGGACGATCCTTTACAAATTTAATCAAATCAGCATGCGGATAGGATGTTGGTATTATAACATCTAACAGTCTACAAATAGAAACAATAACA

>B488_04360|490309..490210|Liberibacter_crescens_BT-1_EZ-Tn5_Transposon_Disruption

GGATTAACATCTGCGCTCTATTTAATGACAATTTTATCCCACAGTGCTTCACATGCTGAAAATCAACTTTCCTTTATTTATGGATACCTTCCATTACTTT

>B488_03240|372221..372122|Liberibacter_crescens_BT-1_EZ-Tn5_Transposon_Disruption

GGTATGCAATCTCCTCATGGAACTACAAGAAAGCCTTGGCATATCGTATATATTCATTACACACGACATGTCAGTTACAGAAAAAATTAGCCACCGTGTC

>Intergenic_Region|670320..670419|Liberibacter_crescens_BT-1_EZ-Tn5_Transposon_Disruption

GAAATAGACTTCCTTGCGCATAGAGACTTGAGTTCTATTAATAGTCAAAAGGTGAGGGATAGACTTGAATTTTTATTCTGCTCCTTGAGTTCAGTTTTTC

>B488_05200|562785..562884|Liberibacter_crescens_BT-1_EZ-Tn5_Transposon_Disruption

TTATAAAACAGTTTCCGCACTGGAAACAGTAAACCGACCCGCGTTAACGGATCGGCTTGGTGCATGATTTTTATACCACACTTATTAGGAGAGGGAAAGA

>B488_05180|560308..560407|Liberibacter_crescens_BT-1_EZ-Tn5_Transposon_Disruption

GTTGTAAAGGGAGCCATAGCAGAGGGAAAGGCAGCTTACGAAGGACATCCGATTGATAACTTTTCCAAAGACTACCAGAGTGTTTCGCAGGGCTGGGATC

>B488_03140|361821..361722|Liberibacter_crescens_BT-1_EZ-Tn5_Transposon_Disruption

ATTTATACCATCCTTTATCGAATTACACGATGCAACATATATCGCATCAACAAGCTGCACTTTATATCACAGCAGGAGGAATATTTTTAATTACCTTAAT

>B488_01190|140426..140525|Liberibacter_crescens_BT-1_EZ-Tn5_Transposon_Disruption

CTTAAATGTGACTAGAGGGCTGCTTGCTCTTTGTAATGATGCATCAGAAATTGCTGCGGTTTTGGCCCATGAAATGGCACATATAATTCTTAATCATGGT

>B488_13640|1490344..1490245|Liberibacter_crescens_BT-1_EZ-Tn5_Transposon_Disruption

GTATATTCTATAGAAGAAGGTATGCTTGGATATTTTATAATATCTATAAAAGATGAAATTAATTTTTCAGATTCTTTTTTAGGCCAGAGACGTGTATTGT

>B488_04010|464605..464506|Liberibacter_crescens_BT-1_EZ-Tn5_Transposon_Disruption

TCTTTACTCCTTGGCGTAAAAACGCAGTCCAGCCAATATAAAGTTTTTCAAACATAAGTGGTGGAGCTTCACCTTTCTTCCAAGAATAAGCATTTCCTTG

>B488_12250|1338929..1338830|Liberibacter_crescens_BT-1_EZ-Tn5_Transposon_Disruption

CTATTGGGTTCATGGTTTTAGAGGGAGATGCGCAATTCTTTTCAGTCTCGAACGATTCATCTTCTACGATGATAGAAAAATCTTTTGGTTCTAAGGTCTC

>B488_13500|1470850..1470773|Liberibacter_crescens_BT-1_EZ-Tn5_Transposon_Disruption

TTCCAGAGCCGTAATCAATAACTGTGACGTGCATTATTTATTCCATTAAATTTACTGGATTTACATCATGTTGATCCTTCTACTCTACTTAAGTTCTCAG

>B488_00930|109586..109685|Liberibacter_crescens_BT-1_EZ-Tn5_Transposon_Disruption

TGTGATAGAGTTGTTTACGAGGCATACCTGTAATTCGTGCTGCAGTGCTTGCAGCTTTTGCTTTTGACATCTTCTGGTATAATGAATATAAAAGCTTGTC

>B488_09870|1074564..1074622|Liberibacter_crescens_BT-1_EZ-Tn5_Transposon_Disruption

GGTATAGGAGATGTTTCAATCTTTGGATCTGGCTATGCCATGCGTATCTGGCTTGATCCTTCAACTCAGCAAAAGTTAAAACCCTTTCCAACCTTGTCAA

>B488_12460|1361433..1361334|Liberibacter_crescens_BT-1_EZ-Tn5_Transposon_Disruption

GCATAAGGAAGCCCCCCTATTAAATTTCGATAAATTGGTTCAATGCGGCCTGACATATCATGTGCTATTGTTCTTTGTGAAAGTTGTAAAATAATAAGAT

>Intergenic_Region|387792..387693|Liberibacter_crescens_BT-1_EZ-Tn5_Transposon_Disruption

CATATACACCTAGCAATTTCTTCCATAACATTCGCTTACCAATTAACACAAATCTAAAATCCAGAATTGCATTACGTCCAAAACAAATTCATTTTTTATC

>B488_05550|594569..594470|Liberibacter_crescens_BT-1_EZ-Tn5_Transposon_Disruption

ATCTTGATCCTATGCTTCTTTTACGTATCAAACAAAAGAATTATCTGGAATTGAAGCTTCCTACGGCTTGCCATTCAAAAAACATGGAAAAAGGTTTCCA

>Intergenic_Region|1024519..1024420|Liberibacter_crescens_BT-1_EZ-Tn5_Transposon_Disruption

GTTTCGTTTTCTAAGCTTGAGACAAAAAATAATAACAATAGTTATTTGAATATACTTTGTATAGAAAAAATTCTTATTTTATGCTATAATGCAGGATAAA

>B488_04510|504653..504554|Liberibacter_crescens_BT-1_EZ-Tn5_Transposon_Disruption

CATCTACTGCTCCTGAACGTATTAATGTATCCGTTATCTCTAAAGCCTGCTCGCCTGTATCTGGCTGTGAGACCAACAAATTTTGCAAATCAACACCAAG

>B488_06680|741151..741253|Liberibacter_crescens_BT-1_EZ-Tn5_Transposon_Disruption

CTCAGAAACATGGCGGCGGGATACGCGTCCTTTTCCTGCCATGGTTGGAGCACTGCCATGAACAGGCTCAAAATAACACATGCTACTTCTATATTGGCGC

>B488_02900|336736..336835|Liberibacter_crescens_BT-1_EZ-Tn5_Transposon_Disruption

ATGGACGTCTATTAGGTCAGGCTTTTGGTAATCTTATAAAAAATGCAGTAGAATCAATCCGAACTGTTCCTTCTCATGAAGAAAGAATCGAAAAAAAGAT

>B488_11680|1274780..1274681|Liberibacter_crescens_BT-1_EZ-Tn5_Transposon_Disruption

GATAAGATTAGATGGTAAGCGTGTCAAATCAGGAGATCGTGTCAACCTTGATCAGGTAATACGTATTCCTCCCCTTGATTTCCTTTCTAACAACAAGAAC

>B488_07610|845475..845376|Liberibacter_crescens_BT-1_EZ-Tn5_Transposon_Disruption

CTGCTACTCTCCAGACAATATCATCTTCAAGCTCTCTATTTTCTCCGTCTATAGAAGCAAGATCCCGGAGAACAGCGATGACTTCAAGTCTTTGTTGAAT

>B488_10540|1149237..1149336|Liberibacter_crescens_BT-1_EZ-Tn5_Transposon_Disruption

GTTGCTGAATGGCCTACCAATTTACAAGCTTCCAGAAAAACTCATCTCTAAATTTCATGATCCTTCAACTCAGCAAAAGTTCCAAGTTATACGTGTTTCT

>B488_00540|68518..68617|Liberibacter_crescens_BT-1_EZ-Tn5_Transposon_Disruption

GTATCAGAGTTGGCACCAAAATCACCCTGAAATGCGATTTTATTTTGTATTTTTTTCATACGACAATAGCTCTGATTAAGAATCACATTGAGATAATAAA

>B488_07710|850079..850178|Liberibacter_crescens_BT-1_EZ-Tn5_Transposon_Disruption

GTGTAATATACGGATAACGCTGAATAATACTTCTTGCCAGCTGGATCACGGCCTTTCGCTGTTCCAGGGGATAGGGTGTAAAATGATATTCATCACCTTC

>B488_09870|1074564..1074622|Liberibacter_crescens_BT-1_EZ-Tn5_Transposon_Disruption

GGGTATAGGAGATGTTTCAATCTTTGGATCTGGCTATGCCATGCGTATCTGGCTTGATCCTTCAACTCAGCAAAAGTCTAAGCCTTCTTATCTATTTTAT

>B488_02020|238625..238526|Liberibacter_crescens_BT-1_EZ-Tn5_Transposon_Disruption

GTGCCATCTATCATAACGGTTCCAGAGATTATTATCCCAGTGTCACTGTTTCGTACACGAATCACGTCACCGACACTAGCATCAGTTAAAGAAATGCCAG

>B488_09450|1017246..1017345|Liberibacter_crescens_BT-1_EZ-Tn5_Transposon_Disruption

CCATAAGGCATCTCTGACCTGTATCAGAAGAAGTACTCTCTAAAAGCTTATGAAATATATTGGCATTCTCGGCACGTTCTTTAGCGACAACTGTTATTCC

>Intergenic_Region|540356..540260|Liberibacter_crescens_BT-1_EZ-Tn5_Transposon_Disruption

GTGATTGTTTTATAGATAATTCTTAATTCTTCAGCTGTTAAGAATCTATTGACTTTTTTTATTTTCTTTTCATCCCACCCCTTCTTTCAATTCAAGACCA

>B488_11520|1251060..1250961|Liberibacter_crescens_BT-1_EZ-Tn5_Transposon_Disruption

CTTCATCACGAAGACGTTGGATAAAATAGAGGACAGCACTACGGGGAGCTAACACAAAATATTTCTTTTGTTGTGTAAAAAATCGATCAAAACCAGCATT

>B488_09870|1075762..1075682|Liberibacter_crescens_BT-1_EZ-Tn5_Transposon_Disruption

GTCATTAACGATCCTTTGTCTTCTTGAGGTATAAAAGAAGAAGGGATACGTTTAAATATCCATAAACACATGACACATATTTCTTGATACGCGTTATTTA

>B488_06750|748723..748624|Liberibacter_crescens_BT-1_EZ-Tn5_Transposon_Disruption

GTATAGACAATACTTCATCCTTGATTAAATTAATTTTTTTTTCCGACGTATAAATAAAATAAGGATATTCCGTTTTACAATCCCTTGTGACTAATTCAGG

>B488_10610|1156383..1156321|Liberibacter_crescens_BT-1_EZ-Tn5_Transposon_Disruption

GCTGTACTCCGGGACAAATAGATAAGATTCTCTCTGTTTTTCCTGAAACAGGAATTGATCCTTCAACCCAGCAAAAGTTAAGTAAAAGTACTGGTGTAAT

>B488_05190|562467..562369|Liberibacter_crescens_BT-1_EZ-Tn5_Transposon_Disruption

ATAATCCTTCCATTAACTATAAGGTCTGCCAGTAACATTAAGGTTGCCAGTAATATTAACAGTTCCCTTTGACATATCCAGCTTCGTTTCTATTGTTTTG

>B488_05090|552208..552109|Liberibacter_crescens_BT-1_EZ-Tn5_Transposon_Disruption

CCCTTATCCAGCTCCTCACGGTTTGTGTTCGACATGGAAGTGATAAAAGTTTGTAATTGTGTTGCCATTTCATTTCTCCTTTATGAGGCATTTTTTTCTA

>B488_05630|611611..611710|Liberibacter_crescens_BT-1_EZ-Tn5_Transposon_Disruption

ATGTTGGGCAGGTTATTGCCCAAATTGATACACAGAAGATACAGGCTGATGTTCATGCTATGCGAGCAAAATTAAATCAAGCCGCAGCCAATTTGTTAAA

>Intergenic_Region|470204..470292|Liberibacter_crescens_BT-1_EZ-Tn5_Transposon_Disruption

GTGTAGTGATTTATAAAAACAGCTATTAAATATAATTCTCAGTCAAACCTAACGTTTTCCTTGTATTTTTTTTACAAAGCTTGATCCTTCAACTCAGCAA

>B488_07830|861187..861088|Liberibacter_crescens_BT-1_EZ-Tn5_Transposon_Disruption

CTGCCGGTCTGGATGTTCCCACGGCTCTCGGGATGCATGGTGTGCCAATGGTTGCCGATGAATCCTTTGGCATGCTTGATTATCTTGATCGTGAACTGAT

>B488_06090|673145..673244|Liberibacter_crescens_BT-1_EZ-Tn5_Transposon_Disruption

ATGTCAGGGTCCCCAACCACTCACCAAAGCGGGTGATAACCAATACTGCTCCTGTTGATGCGCCAAAAGAAAGACCGAAAAGGAATGGATCTGCCAAATC

>B488_01910|231041..230942|Liberibacter_crescens_BT-1_EZ-Tn5_Transposon_Disruption

CCTAGATCCTTAGCATGAAAACTAACTAGCCTCCCTGGAATTGACCCGGCGGGAAGTGGCAACACAAGATAATACAGATTGTAATAAAAAACATTTTTTT

>B488_03100|356527..356428|Liberibacter_crescens_BT-1_EZ-Tn5_Transposon_Disruption

GAATTACTCACTGGTATGAAAATACTTTGGTAATCTTCAATAGAGTTACTAGCATAACTTTTTTGAAAAAAAAGGAAAAATATTATGAGTATAGCATGTA

>Intergenic_Region|1412388..1412487|Liberibacter_crescens_BT-1_EZ-Tn5_Transposon_Disruption

CCTTTGTATCAATAAGCTCTATTAACACCATCCATATAGTTATTGAATATCTATTACTACTTTAACGATATATTGCTTTAATATTTAATACAAGGAATTA

>B488_07060|786304..786205|Liberibacter_crescens_BT-1_EZ-Tn5_Transposon_Disruption

GCGCACGAAAGGTCATACCAAGACACTGTTGCCGGATAACGTCCACCAGACGCCGGGTTGCCCGACGTTTATCATCCATAAAACTCATCTCCTGCATGAA

>Intergenic_Region|329744..329645|Liberibacter_crescens_BT-1_EZ-Tn5_Transposon_Disruption

TGCTCTACCTGACCACCTGGGTCGGTTTCGGGTACGGTCTATACGGCGGAGCTATTTCCTGGAACCACTTCAAAGCATTTTCAATCCATTAAGAAAATAC

>B488_12280|1342344..1342443|Liberibacter_crescens_BT-1_EZ-Tn5_Transposon_Disruption

GATCTATTCTGGAGGACACAGAATTGGTTGTCACCTATTCCAGCATTATGATATGACAACATTAAGTGACAGGAGTATAAGAAACCAAATACAGATGTTC

>B488_09280|1001814..1001916|Liberibacter_crescens_BT-1_EZ-Tn5_Transposon_Disruption

GATATGTAGGTCGTAATGCAATGCTATGATCCTTCAACTCAGCAAAAGTTCCATCCTGCGCATAAAGAAGACTCCGTTAGGATACCCAAACTACCTTAAA

>B488_12120|1325313..1325214|Liberibacter_crescens_BT-1_EZ-Tn5_Transposon_Disruption

GTTGAAGATGTTCTCTATGGCTCTTTGTCTTGAAGTTATGGCAATTCTTTCACCCTTGGGTTTACAGATGATTATAGATCAGGCTGTACTTAGCGCGGAT

>B488_09900|1081147..1081048|Liberibacter_crescens_BT-1_EZ-Tn5_Transposon_Disruption

GATTTAAGACATGTCTGTGAAAGAATATATTTTTCTTTTAATCTTTTTGCTATCTCTCTCGGTGAGCGACTAGTGTTATTAGAATAACTAGTAAAATGCA

>B488_11140|1212822..1212921|Liberibacter_crescens_BT-1_EZ-Tn5_Transposon_Disruption

AGCTGATACGGCAAAAGCAGAAACTTTACCGCGTTTTCGGAAAAAAAATGCAGTAATCAATAAACAACAATCTGGATTTGATCCTGTAACAGAGTGCGAT

>B488_03720|425921..425822|Liberibacter_crescens_BT-1_EZ-Tn5_Transposon_Disruption

CCCTTTACACCTATTTTTATAATATTAAGTTTTGCAATTCATCCAGTGCTTGGATTATTAACAATTATTAGTGCAGTAATTTTAATAACAATCACTCTCT

>Intergenic_Region|387792..387724|Liberibacter_crescens_BT-1_EZ-Tn5_Transposon_Disruption

CATATACACCTAGCAATTTCTTCCATAACATTCGCTTACCAATTAACACAAATCTAAAATCCAAAATTGAAATTGATTTTTTTTGATTCATTTTTTATCA

>Intergenic_Region|329143..329044|Liberibacter_crescens_BT-1_EZ-Tn5_Transposon_Disruption

CTAGTACTCCTCGGTATTCGGAGTTTGGTTAGGAGCAGTAAGGCGGTGAGCCCCCATAGCCTATCCAGTGCTCTACCCCCGAGGGTATTCAGTTAACGCT

>B488_05530|599962..599863|Liberibacter_crescens_BT-1_EZ-Tn5_Transposon_Disruption

AAAGAAGACAGAACATCAAAGGAGTAGTCAAGAGCGTATAATAAACAATTGTACCCCATCGATTGGTATTCAGGAAGAGATTGCCAAGACAAACCGGGAT

>B488_05190|562187..562286|Liberibacter_crescens_BT-1_EZ-Tn5_Transposon_Disruption

GTTTTAGGATCCATAATATCAAAGGGTGATCTTTCTGGCTCTGTTAACCTTATCGTTCAACAAGGGCCATCTGGTGGTAACTGGCAGATAGTGTTCAAGG

>B488_01210|145669..145768|Liberibacter_crescens_BT-1_EZ-Tn5_Transposon_Disruption

TTCATATAATGGGCTTATTCCTTTAAATTCTTATGCTAAATCTGGAGTTTCAGGTTCATTGGGAGGAGGGAATCTAACATTGATTCAATCTTTATTCTCT

>Intergenic_Region|670320..670419|Liberibacter_crescens_BT-1_EZ-Tn5_Transposon_Disruption

GAAATATACTTCCTTGCGCATAAAGACTTGAGTTCTATTAATAGTCAAAAGGTGAGGGATAGACTTGAATTTTTATTCTGCTCCTTGAGTTCAGTTTTTC

>B488_11970|1306251..1306152|Liberibacter_crescens_BT-1_EZ-Tn5_Transposon_Disruption

GATAATAGTCGTGATAGGTTACGTGATCCTAAAGGTTCACCTGTTTCCAAATAACTTTCAACTATTCGACGAAAAACTTCTCGATCACGATCGTCTAATG

>B488_11620|1265089..1264990|Liberibacter_crescens_BT-1_EZ-Tn5_Transposon_Disruption

GGCGGAGGAAGTTCTGTACGTGGCTATAAATACAATGAAATTTCTCCACGTAATGCTTTAGGACAAGCAATTGGAGGGAATTTTTTTATAACTTTGTCAT

>B488_02690|305754..305848|Liberibacter_crescens_BT-1_EZ-Tn5_Transposon_Disruption

CTGGTAAGCGTTAATGGGTGTTTTTAAAGTGATTTCTATTACTGCTCTTTTTGCAGGGATTGGTATTATTCTTTCAGCCTCTTATGGTTTATGGGTTTAC

>B488_09160|988088..988146|Liberibacter_crescens_BT-1_EZ-Tn5_Transposon_Disruption

GAGTTGGGGTTGCCCTTCATTCGTGCTGCAAATAACGGTATAAACCCTCCTGATGGCTGATGAACCATAATACGAGCGTTAGGCAACACATAACGATGAC

>B488_12120|1325313..1325216|Liberibacter_crescens_BT-1_EZ-Tn5_Transposon_Disruption

GTTGAAGATGTTCTCTATGGCTCTTTGTCTTGAAGTTATGGCAATTCTTTCACCCTTGGGTTTACAGATGATTATAGATCAGGCTGTAGTTAGCGCGGGT

>B488_10910|1186873..1186774|Liberibacter_crescens_BT-1_EZ-Tn5_Transposon_Disruption

AGGTTGGAGGTCTTGATGTTAATAATTTTATTGAAATCAATAAATTATTACAGCGCCTTGATCGTTTCTGGAATGATCAAATTTTATACCGCCTTTAAAC

>B488_04410|494625..494559|Liberibacter_crescens_BT-1_EZ-Tn5_Transposon_Disruption

ATTGAATGCCGAACGGCATGCTCTAAAGAAATAAAGAAATTTTTATCAGGATAAACAGAAATTGATCCTTCAACTCAGCAAAAGTTCCAAGGGCTGCTGA

>B488_04690|525898..525997|Liberibacter_crescens_BT-1_EZ-Tn5_Transposon_Disruption

CTTCCTGTTCATGATCAATGCCCCATTGCATTTCCTTGTTGATGAAACTTTCCGCTGTTATTCCTGTTAAACGTTCAGAAACAAGTTCGGCTTTATAGTT

>B488_05390|582619..582520|Liberibacter_crescens_BT-1_EZ-Tn5_Transposon_Disruption

GGCCTTAAGTACACTCTTGATATTTTAATAGTGCTATGTTTTTACAATAAAACATTACATATTTGAATAAAAAGGCCCTTCCCCTCCTTGCGGTGGCACC

>Intergenic_Region|669994..670093|Liberibacter_crescens_BT-1_EZ-Tn5_Transposon_Disruption

CCTTCTAACTGTAAGATGCAGATCACGTATAGCAATTATGTCAACATAGAATTTAGATACTGCTTTTAAAGAGAGGACTTCATTTCTTTCTTTTGACGAG

>Intergenic_Region|669994..670093|Liberibacter_crescens_BT-1_EZ-Tn5_Transposon_Disruption

CCTTCTAACTGTAAGATGCAGATCACGTATAGCAATTATGTCAACATAGAATTTAGATACTGCTTTTAAAGAGAGGACTTCATTTCTTTCTTTTGACGAG

>B488_05530|599962..599863|Liberibacter_crescens_BT-1_EZ-Tn5_Transposon_Disruption

AAAGAAGACAGAACATCAAAGGAGTAGTCAAGAGCGTATAATAAACAATTGTACCCCATCGATTGGTATTCAGGAAGAGATTGCCAAGACAAACCGGGAT

>B488_08420|919833..919932|Liberibacter_crescens_BT-1_EZ-Tn5_Transposon_Disruption

CTTTAAAGATGTAACCTTTCGGTATAATAAAAATAAAATTGGCTTGGAAAACATTAATATCTTTATTCCTGCTGGAATGAAAATTGGATTAATAGGCCGT

>Intergenic_Region|669994..670093|Liberibacter_crescens_BT-1_EZ-Tn5_Transposon_Disruption

CCTTCTAACTGTAAGATGCAGATCACGTATAGCAATTATGTCAACATAGAATTTAGATACTGCTTTTAAAGAGAGGACTTCATTTCTTTCTTTTGACGAG

>B488_10710|1168544..1168642|Liberibacter_crescens_BT-1_EZ-Tn5_Transposon_Disruption

CATATCAGCAGACCCTATATAGGCAATAGCCTCTTCAGAAGGCAAACCATAACCGTTACCAAAACAAAAAATACGACTATGTTCGAGGAAACGACCCACT

>B488_01270|156273..156174|Liberibacter_crescens_BT-1_EZ-Tn5_Transposon_Disruption

GGTCAGTAGCATCCTCAGTGACTACACAATAAAGTCCCCAGGAAAGCGAAAGCCTCCGTGCTGTCTGCATAATAGGAGACAAAGCCAAAATAGGTATTTG

>Intergenic_Region|330836..330737|Liberibacter_crescens_BT-1_EZ-Tn5_Transposon_Disruption

GCTTTCAGCGGTTATCCATTCCGTATGTAGCTACCCTGCTATGCCCTTGGCAGGACAACAGGTCCACCAGAGATACGTCCATCCCGGTCCTCTCGTACTA

>B488_09220|993766..993852|Liberibacter_crescens_BT-1_EZ-Tn5_Transposon_Disruption

TTGTTATACAAGTCTCGTGCATGCAAAGCAGCCATTTGGAGGTATATTAACAGAACAACAATATTATTCGTTGATCCTTCAACTCACATGAAGTTCCACA

>B488_13500|1470850..1470773|Liberibacter_crescens_BT-1_EZ-Tn5_Transposon_Disruption

TTCCAGAGCCGTAATCAATAACTGTGACGTGCATTATTTATTCCATTAAATTTACTGGATTTACATCATGTTGATCCTTCAACTCAGCAAAAGTTATGCT

>B488_08350|911589..911688|Liberibacter_crescens_BT-1_EZ-Tn5_Transposon_Disruption

GGTGAACAGATTACCGAGACTCCTGTCATCCTTCAGTATATCGCCGACCAGGCGCCTGAAAAGAAGCTCGCACCTGCTCATGGTACGTTAGCACGTGTAC

>B488_02900|336736..336836|Liberibacter_crescens_BT-1_EZ-Tn5_Transposon_Disruption

ATGGACGTCTATTATGTCAGGCTTTTGGTAATCTTATAAAAAATGCTCTGAATCAATCCGAACTGTTCATTCTCTTCAAGAAAGAATTAAAAAAAAGATT

>B488_01910|231077..231176|Liberibacter_crescens_BT-1_EZ-Tn5_Transposon_Disruption

GAGCTATATTAGCTGATTATGGTCTGGATAGATATATTAAAGATATAATACTTGTCACTATGCCACGTGTTTTGGGTTATGTATTTAATCCTATCAGTTT

>B488_06680|742021..742087|Liberibacter_crescens_BT-1_EZ-Tn5_Transposon_Disruption

GCCGTACAGACATCTTGACCGATACCATCGCCAGGAAGCACAAGTACTTTTTTCATGATGATCCTTCAACTCAGCAAAAGTTAAGACTCCGCGGGTCGAT

>B488_04690|525898..525997|Liberibacter_crescens_BT-1_EZ-Tn5_Transposon_Disruption

CTTCCTGTTCATGATCAATGCCCCATTGCATTTCCTTGTTGATGAAACTTTCCGCTGTTATTCCTGCTAACCGTTCAAATTCAAGTTCGGCTTTATAGTT

>Intergenic_Region|1157984..1158082|Liberibacter_crescens_BT-1_EZ-Tn5_Transposon_Disruption

GGAGATAGAGCAATCATTATATTGATAAAATTCCGCAGACTGGTTTAAAAAAACCTTTTTATAGATTGTATAAGAAAGTACAAGCAACTATCATTGCTAA

>B488_11150|1215463..1215503|Liberibacter_crescens_BT-1_EZ-Tn5_Transposon_Disruption

TTCGCTATAGCGCGTGCCATTATCAAGAACCCACCGATTTTGATCCTTCAACTCAGCAAAAGTTAAGATTGTGATTTACAGAAGGAAATATACCTAATGC

>B488_05410|584600..584699|Liberibacter_crescens_BT-1_EZ-Tn5_Transposon_Disruption

GGATATTATTGCCGTCACCTGCTTTCAGCGTATCATTACCCTCGCCGCCAATGATCAGGTCATCGCCATTGCCTCCGGCCAATTTATCGTTGCCCTGTGT

>B488_12160|1329444..1329345|Liberibacter_crescens_BT-1_EZ-Tn5_Transposon_Disruption

CTACAGAGATGTTACGTCGTGATTATCGTGAGATGTTATTTAGTTCTAAAGAAGCTATGAAATTTATTTCAGGAGTTATTCTGTATGAAGAAACTCTTTC

>B488_11070|1206760..1206661|Liberibacter_crescens_BT-1_EZ-Tn5_Transposon_Disruption

CTTTTCAGACAGCTTTGGAAATGGCACAAGGTGCATTAATTTTTAGTCATGCAACTTTTGCAATTTCTGCTACAGGTATTGCTGGTCCTGGAGGAGGCTC

>B488_13270|1440674..1440773|Liberibacter_crescens_BT-1_EZ-Tn5_Transposon_Disruption

CTCAAGGGCTGATTAGAATTGCTCCCCCAGGCCTTTCATATGTCTTCTTTTCTGATAGCGGCTCAACTTCAGTAGAGGTAGCTCTAAAAATGGCCCTGGG

>B488_08600|943527..943626|Liberibacter_crescens_BT-1_EZ-Tn5_Transposon_Disruption

GATTAATCGTATTGATATAACTTCTTGGAACAACAGAAAAATTAACAAAAGGGGGAGCCATCAATGCATTATTGGCGTTAATTTGCTCTGTCACCGCAAA

>B488_01780|218866..218922|Liberibacter_crescens_BT-1_EZ-Tn5_Transposon_Disruption

GTAATAGCTGAATTAAATACAGATACTCAGAAAATTCAAGAAACAATATATGATCCTTCAACTCAGCAAAAGTTAAGATAGCATGTTGCTAATAACATTC

>B488_13370|1452149..1452050|Liberibacter_crescens_BT-1_EZ-Tn5_Transposon_Disruption

CACTATATCCATTTCAGCCGGAGTTATAGATTCAACATTGCCAGATACTATCTTAAAGCAGATAGGAGAAGAAGTAAAAACTTTTCAGAACAAATTACCT

>B488_07980|874070..873971|Liberibacter_crescens_BT-1_EZ-Tn5_Transposon_Disruption

TTCCAGGATAACCCTGGAGGGCGATGCAACAACGGTTATTATTGATACAGGTCCTGATTTCCGCGCCCAAATGATCCGTGAGAATGTCTCTGATATTGAT

>B488_01780|218866..218922|Liberibacter_crescens_BT-1_EZ-Tn5_Transposon_Disruption

GTAATAGCTGAATTAAATACAGATACTCAGAAAATTCAAGAAACAATATATGATCCTTCAACTCAGCAAAAGTTCCATCTGTTATTGGAAAATTCACAAA

>Intergenic_Region|393931..393832|Liberibacter_crescens_BT-1_EZ-Tn5_Transposon_Disruption

CTATAAAGATATCCCGTGGCTGGTGAAAGAATGTGGAAAAAAATGAAAGTATTTTTCTTAAATCATTCGGTATATAAGGAAGATTAAGACAGGAAAAGCC

>B488_05900|649265..649363|Liberibacter_crescens_BT-1_EZ-Tn5_Transposon_Disruption

CCCTATACCTGTATTCTCCAGATGTAGGTTCAATAAGAATTGTTTTTCCAGGTATTATTTTTCTAGCTTTTTCAAGGGCTAGGATCATTGCCACTCCTAT

>Intergenic_Region|329614..329713|Liberibacter_crescens_BT-1_EZ-Tn5_Transposon_Disruption

GGCCTGGCGGTAGTGACGGATCACGTAAATTGTATTTTCTTAATGGATTGAAAATGCTTTGAAGTGGTTCCAGGAAATAGCTCCGCCGTATAGACCGTAC

>B488_00030|4360..4459|Liberibacter_crescens_BT-1_EZ-Tn5_Transposon_Disruption

CAATGGTAGCAGCTCATTCAGTACTTGTTCCATTACAATGTGAGTTTTTTGCTTTAGAAGGTCTTAGTCAGCTTTTAGAAACAGTTAATGAAGTTCGAGA

>B488_07300|815104..815203|Liberibacter_crescens_BT-1_EZ-Tn5_Transposon_Disruption

ACCTATATGTGTCCTTGATCCTTCAACTCAGCAAAAGTTCCTTATTAATCCTGTGGTTTCTGTGCTGTGTCGAATGGCTTTCGAAATTGCTGCGTTTAGT

>B488_09960|1089199..1089150|Liberibacter_crescens_BT-1_EZ-Tn5_Transposon_Disruption

ATTCGAGGCAGCCCATATTACAGAAGAAGCACGTAAAGTTGATCCTTCAACTCAGCAAAAGTTCCACGTGGTTGGTGGGGCTATTTTGCTCTGTCCTCCC

>B488_06100|673756..673855|Liberibacter_crescens_BT-1_EZ-Tn5_Transposon_Disruption

AGCTAGACATCATATTGTCCATGACATTACGCCCACCTGCTGCTTCAATAATTGCAGTTGGCATTGCGTACACGCCGCTGGTAAAAGGTTTGTCTTCACC

>B488_02720|308752..308653|Liberibacter_crescens_BT-1_EZ-Tn5_Transposon_Disruption

GAATAGAATGATTAACTTGAACTGCTTCAATTGAAAAAGGACCTATATTAATACAATCACCAGCCTTAAAAGAAGTTATAGGAATATCTATTGGAATACG

>B488_01600|193388..193487|Liberibacter_crescens_BT-1_EZ-Tn5_Transposon_Disruption

GCGCATGCTCCTATTCCAGGAAATCCAGAACCTGTTGTAATAATTTGGCCAGAAAAAGCTATTCCTTTTATTGTTACAGATAAACCAGATGTTTTATTAC

>B488_06930|771750..771849|Liberibacter_crescens_BT-1_EZ-Tn5_Transposon_Disruption

CTTCAGGATTAATATAACTAAATGCAAGTTCTTCCAGTTCTTCACGCATGTCTTGCATGCCCATGCGTCCAGCTAACGGAGCATATATATCCATCGTTTC

>B488_10370|1133242..1133341|Liberibacter_crescens_BT-1_EZ-Tn5_Transposon_Disruption

GGGGTACAACACGCTTAGGTCCTAATCCTCCTATATGTAGATGAGTAGAAGATGAACCAGGGCCACCACCATAAACAAACGTTATAGGGCGTTTTTCACT

>Intergenic_Region|235868..235769|Liberibacter_crescens_BT-1_EZ-Tn5_Transposon_Disruption

ATGTAACTCATACCCCATTGATTTTACTGAGTTTTTTCTTGGATTTGGTTGCACCTTAAAATAGGAAGGGTATTTACTCATCACGCAGTTCCATCTTCAT

>Intergenic_Region|330629..330530|Liberibacter_crescens_BT-1_EZ-Tn5_Transposon_Disruption

CCCTTGGGACCTGCTTCAGCCCCAGGATGCGATGAGCCGACATCGAGGTGCCAAACAACCCCGTCGATATGGACTCTTGGGGGTCATCAGCCTGTTATCC

>Intergenic_Region|433059..433158|Liberibacter_crescens_BT-1_EZ-Tn5_Transposon_Disruption

CCTTATGTTCAGCCTCAAACTCCTCCTAGCGGCGCTACGGACACAGGTTTGGGGACTTCTGTTCAGCAGCAAAAATCTGAGGGGACTCCTGTTCAGCAGC

>B488_03730|428528..428627|Liberibacter_crescens_BT-1_EZ-Tn5_Transposon_Disruption

CTATGGGCATCAAGAGCATAGGACGATAGACTTTAATGGGCGTTTATATTTTGAGCAGCATCGGGATAAGTATACTGATATTTTGAAAAAAATAGAAGAA

>B488_05430|587020..587119|Liberibacter_crescens_BT-1_EZ-Tn5_Transposon_Disruption

CAGCTACAATAAGAGGTATTAATAAGAGCACCATCAAAAGGTTTCTTAAAACATTTATGATAGGGCGGCATACTTCCCAGGATGGAATGATAGGTAAGCG

>Intergenic_Region|235868..235769|Liberibacter_crescens_BT-1_EZ-Tn5_Transposon_Disruption

ATGTAACTCATACCCCATTGATTTTACTGAGTTTTTTCTTGGATTTGGTTGCACCTTAAAATAGGAAGGGTATTTACTCATCACGCAGTTCCATCTTCAT

>B488_07050|785811..785910|Liberibacter_crescens_BT-1_EZ-Tn5_Transposon_Disruption

GTGCAACAGGCTGTGGTTCGTGCTCTTTCCAGCCATCAGAAACTTGCTACGCAAGTGCTTAAATCCGACCGACAGGGTATGTCAGCTCTGGTCGATGTGA

>B488_06570|726352..726253|Liberibacter_crescens_BT-1_EZ-Tn5_Transposon_Disruption

TACATAATCTCTCCATGTTGAATGCTGCCAAAGACATGATCGAAACTGAAGAGTTACCAAAGCAACGTAAGGCTCCTCTAGCCAATCTCCTCGAAAATAT

>B488_09230|994929..995028|Liberibacter_crescens_BT-1_EZ-Tn5_Transposon_Disruption

GTTGTAAATCCTCTTCTTCAGAAGCTGCTGAACGGACAAAATAACCTGACTTTTGTACAATAGATCTCTTTGCTCCTATAAGTTCCGCAAACTTTTCAGA

>B488_07050|785811..785910|Liberibacter_crescens_BT-1_EZ-Tn5_Transposon_Disruption

GTGCAACAGGCTGTGGTTCGTGCTCTTTCCAGCCATCAGAAACTTGCTACGCAAGTGCTTAAATCCGACCGACAGGGTATGTCAGCTCTGGTCGATGTGA

>B488_07940|870748..870847|Liberibacter_crescens_BT-1_EZ-Tn5_Transposon_Disruption

CCTTTACTCTGGTTTTGATCGCCATGCTATGGTCTGTCGCCTATTGGGCAAAGGCCTGACGATGACGAAAGCCATTACCCTGCATGACCTGACGCTTGCC

>B488_00200|29561..29660|Liberibacter_crescens_BT-1_EZ-Tn5_Transposon_Disruption

TGCGTGGGTAGTTATGGCTCTTATTGAGCTTGGTCGGTATGATGAGGCTTGGAAATGTTTTGCAATGTTAATGCCAATAAATCATTCTCTTACATATGAA

>Intergenic_Region|418388..418289|Liberibacter_crescens_BT-1_EZ-Tn5_Transposon_Disruption

GGTTACAGACTTACCCATTATAGATCCTTGCCTATATTATTCAACCTTGAAACATCTGAAGAAGAAGTTGCTATACACAGAAACCCTTATAAACTTATTG

>B488_05090|552611..552710|Liberibacter_crescens_BT-1_EZ-Tn5_Transposon_Disruption

TGGCACAGGTGGGCAAAGTGGCGGTTTTTCTGGTGGTGTTACAACGGCACCAAAAGCAGGAACGCCAAGGACTATTTCTCTTTCCCAACTCGAAAACGTC

>B488_04390|493001..493100|Liberibacter_crescens_BT-1_EZ-Tn5_Transposon_Disruption

CATTAAGGTTGAAAATATTATTTATAGGGAATTCCCAAGCTATATGCCAATTTCCTGAAACAGAACACAGCCTGTCTGAGCACATTGGTGCAGCAGCTTT

>B488_06570|726352..726253|Liberibacter_crescens_BT-1_EZ-Tn5_Transposon_Disruption

TACATAATCTCTCCATGTTGAATGCTGCCAAAGACATGATCGAAACTGAAGAGTTACCAAAGCAACGTAAGGCTCCTCTAGCCAATCTCCTCGAAAATAT

>B488_04790|532234..532135|Liberibacter_crescens_BT-1_EZ-Tn5_Transposon_Disruption

TAGGTATAGTCAGCAATGAACAAGCCTTAAAACCAATAACTAAAGTAGTTGATGAGAACGCCTGGCCTCCTGAATATAGGAATGCTTTGGATTCTTTATT

>B488_04390|493001..493100|Liberibacter_crescens_BT-1_EZ-Tn5_Transposon_Disruption

CATTAAGGTTGAAAATATTATTTATAGGGAATTCCCAAGCTATATGCCAATTTCCTGAAACAGAACACAGCCTGTCTGAGCACATTGGTGCAGCAGCTTT

>Intergenic_Region|1469977..1470072|Liberibacter_crescens_BT-1_EZ-Tn5_Transposon_Disruption

CTTAAAGACTTAATTAGCAATCTCATAAAATTATATATATGTAATAGATACTTAATATATCACTCGTTATACATCGTATTCTTTTAAACGAGATTATTTA

>B488_09870|1074564..1074622|Liberibacter_crescens_BT-1_EZ-Tn5_Transposon_Disruption

GGTATAGGAGATGTTTCAATCTTTGGATCTGGCTATGCCATGCGTATCTGGCTTGATCCTTCAACTCAGCAAAAGTTCCATATGGATATTCCACCATCAC

>B488_06170|681881..681782|Liberibacter_crescens_BT-1_EZ-Tn5_Transposon_Disruption

ACGTAATGGTTCTGGGAAATCTACTTTATTAAAAATTGCAGTTGGAGTCTTAGAGACATACTCTGGTGAAGTTTTTCGTCATCCTTCTGCCACTATCGGA

>B488_03370|389099..389000|Liberibacter_crescens_BT-1_EZ-Tn5_Transposon_Disruption

CCTATAAATATACCAGCCAAAACAAAAGAAACCCTACCTATCATCCTCTCCCTTTCTAAAAGCATTCTTGCTCCACCATGCACGTGAATCAATAGGCTTT

>B488_05910|650234..650135|Liberibacter_crescens_BT-1_EZ-Tn5_Transposon_Disruption

ACCCCGTACAGTATACAACTGCGACTTTGAAGGAGCAAGGCATCATCCTCTTCTCCAGCTAGCCATAAAACATGCGCCAAACGGTGAGTTTGCAGTGCAT

>B488_13240|1438260..1438359|Liberibacter_crescens_BT-1_EZ-Tn5_Transposon_Disruption

TCTTTGGACTGGGAGAAACTGTTGATGACCGCGTTGATATGTTAGTAACCCTTTCTAACTTAAAAAGTCCACCTGAAAGCGTACCTATTAACATGCTTAT

>B488_10900|1185304..1185403|Liberibacter_crescens_BT-1_EZ-Tn5_Transposon_Disruption

GGCTTGCGGGGTATAGAACTATTTGGTGCTAACGAGTTTCCATCTAGATTATAAATATCTTCTCTAAGTTGTACAACATGATCTTTGGAAGACCATGCAG

>B488_10810|1178716..1178815|Liberibacter_crescens_BT-1_EZ-Tn5_Transposon_Disruption

GGTCTAACATTACGAACAATGCATATCAAAAATGGCATAGCAGAGGTACGTGTTGGAGCGACTTTGCTCAGCGATTCCAATCCCGATAAAGAAGAAACCG

>B488_11600|1259916..1260015|Liberibacter_crescens_BT-1_EZ-Tn5_Transposon_Disruption

CCTCGTTGCCGCTCAAGATGCGTCCAACTACGAACAAGACGACCTTTTTGATAATTAAGATGAGCTAATTCTACCTGTAGAGCTCCTTCTTTAGTCAAAG

>B488_06810|758459..758359|Liberibacter_crescens_BT-1_EZ-Tn5_Transposon_Disruption

GGATTGTCTTCCCTATGATCGAATATCACCTTCAAGGCATATTTCTGCAAGCCGTTTGTCAACGTTTTTTCATTTACTTTCCTATAAAAAAAAGCAAAAA

>B488_12170|1330284..1330383|Liberibacter_crescens_BT-1_EZ-Tn5_Transposon_Disruption

CTTTAGAACCACCAATAATGGCAATAATTGGCCTATGAGCTGTATCCAGATATTTTTTAATCATATTAAGTTCTAGTTGCAGCGAACGACCAGTATAGGC

>B488_07780|856579..856480|Liberibacter_crescens_BT-1_EZ-Tn5_Transposon_Disruption

ATATATGATTACGGCGAATATCCTCTGTGTTTCGTCTTCTTCCCATAACAAGGATATCCATTTGCTGTATTACGGGCGACTTCCGGCGCTGCGTCCTGAT

>B488_03270|377540..377441|Liberibacter_crescens_BT-1_EZ-Tn5_Transposon_Disruption

GGGTATAATACCCTATAAGGATAAACTTGGAAGAAAGTAGTTCCATCATGTAAAAGACGTCCCCAAGTAGGAAAATCAGAAGGGAAACCAAATCCTAAAA

>B488_08580|941144..941045|Liberibacter_crescens_BT-1_EZ-Tn5_Transposon_Disruption

GTCTTGATGAGACAAGGGATACATGGCGCTACCTTGTCGGTCTTCTTGTCTTTTTAGGTCTTTTAGGAACTTTTTGGGGATTAATTGATACGATAACTTC

>B488_01400|172871..172772|Liberibacter_crescens_BT-1_EZ-Tn5_Transposon_Disruption

ACACAACATAGCAGTACCAAACCAACCAATCATTGCCGCACCTATACCGGATGTTATGTGATCATAACCGGGGGCAATATCAGTAACAAGCGGCCCAAGA

>B488_05900|648991..649059|Liberibacter_crescens_BT-1_EZ-Tn5_Transposon_Disruption

GATAGTACTCGTGCCAGGTTACGTGATCCTTCTTCTCCTTGAATGTCCTTCCAAATTTCTTCAGCTGTTGTTTGGCGATGAATTTCTGGATTGGCAGGGT

>B488_12860|1401622..1401721|Liberibacter_crescens_BT-1_EZ-Tn5_Transposon_Disruption

CTCTTCATTAGTATAATTATGGTTAACATTTTTTGATTAAATACTAATTAATTAATCCATTAAGTGCTATTTTAAATATAACGCTGTCCGGATAGGATAT

>B488_03730|429930..429831|Liberibacter_crescens_BT-1_EZ-Tn5_Transposon_Disruption

ATCTCTCCCTTTGCTGCGAATGACCAAAACGCACATAATGTTTGTAGCCGTAATCCTCAATAGATTCATGATCTTTATAATTTTCATTATAATCTTTGAG

>B488_13270|1440491..1440590|Liberibacter_crescens_BT-1_EZ-Tn5_Transposon_Disruption

GTACAAAAGATGCCTATCTTATTGACGAAGATGGTTTTTTAATTTTCGATGCAATCTCCTCGTGGTGGGTTATTACACATGGACATCGTCATCCAACTAT

>Intergenic_Region|1032561..1032462|Liberibacter_crescens_BT-1_EZ-Tn5_Transposon_Disruption

ACTGGAAATAGTTTAAGGAGAACTTTTATCACGATGATAATTTGGTGCTGAACCAGATTATTAGGAATTATTTAAGTTTATGCAGATCAAGCTCATTTTC

>B488_01690|202575..202674|Liberibacter_crescens_BT-1_EZ-Tn5_Transposon_Disruption

GAAGAATAGATTGGTTTGAGCAATAAACCATGTAAATGCAAAGGATGGGCTTGGGGTGTAGGGTTGTCAATATTCAGTACGTAGGTCTTTCCTATTTTCA

>B488_10810|1178716..1178815|Liberibacter_crescens_BT-1_EZ-Tn5_Transposon_Disruption

GGTCTAACATTACGAACAATGCATATCAAAAATGGCATAGCAGAGGTACGTGTTGGAGCGACTTTGCTCAGCGATTCCAATCCCGATAAAGAAGAAACCG

>B488_08580|941144..941045|Liberibacter_crescens_BT-1_EZ-Tn5_Transposon_Disruption

GTCTTGATGAGACAAGGGATACATGGCGCTACCTTGTCGGTCTTCTTGTCTTTTTAGGTCTTTTAGGAACTTTTTGGGGATTAATTGATACGATAACTTC

>B488_10520|1146410..1146509|Liberibacter_crescens_BT-1_EZ-Tn5_Transposon_Disruption

GGGTTATGGTATCAATGATCCCTTCAAAAACCCATTGACGTGTATCTGCATAACTATCATCATAATTTGTTGAAAGAACTTGCGTGTCTGAACCGGAGGG

>B488_04540|510932..510833|Liberibacter_crescens_BT-1_EZ-Tn5_Transposon_Disruption

ATTTAATAGATTGGGAGAAACAACAATAGCAGCAGAAATATCAATAACAGACCCTTTATTAACTTCCTTTGTAACAACATTGGCAACATCAGCAATAATA

>B488_00360|49767..49696|Liberibacter_crescens_BT-1_EZ-Tn5_Transposon_Disruption

AAACAGTATTCTTCAAAATTGAGTGCACATCTGCAATTAAACACCCAATCTCTGTCATAGGAACCACATGATCCTTCAACTCAGCAGAAGTTCAAGTTTG

>B488_13300|1444031..1443932|Liberibacter_crescens_BT-1_EZ-Tn5_Transposon_Disruption

GTTCAAGGTGTTATTGAAGATGCACTTTTTGCATTAACAAAAGAAACTGTGACGGTATATGGAGCAGGTCGTACAGATTCTGGAGTACATGCTCTAGGAC

>B488_03370|389099..389000|Liberibacter_crescens_BT-1_EZ-Tn5_Transposon_Disruption

CCTATAAATATACCAGCCAAAACAAAAGAAACCCTACCTATCATCCTCTCCCTTTCTAAAAGCATTCTTGCTCCACCATGCACGTGAATCAATAGGCTTT

>Intergenic_Region|778604..778703|Liberibacter_crescens_BT-1_EZ-Tn5_Transposon_Disruption

GCATAAGAGACTTCGTCATAAAGCGGTTTATACTCCTGAAAAAAGAATTTTCTTGCCGCGAACCTGATGGAAGCACATAAAATCCTTCAAAACACAACTT

>B488_01560|188652..188751|Liberibacter_crescens_BT-1_EZ-Tn5_Transposon_Disruption

ACGCATGACAGAGCAACGTAAGTTAATAGCGCGTATTTTAGAAGAATCTGAAGATCATCCAGATGTCGAGGAGCTTTATAACCGATCAGTAGCTTTTAAT

>B488_06610|734465..734366|Liberibacter_crescens_BT-1_EZ-Tn5_Transposon_Disruption

CACTAATAGCTGTGGAACCGTGACAGAAGTATCTATTCCATGTATGTTTTCTATATATCCAAAATCAGAGTACAGCTTCAAGAAAGGACGTACAACAGAA

>B488_00930|110093..110192|Liberibacter_crescens_BT-1_EZ-Tn5_Transposon_Disruption

GTATAGCAAGATATACAAGATGATATCCAGGATCAGATATTAAAGGTGTTCCAGAATCTGAAACCAAAGCTATTGAAGCTTTTTCCTGTAAAGCATCAAG

>B488_10760|1174181..1174280|Liberibacter_crescens_BT-1_EZ-Tn5_Transposon_Disruption

CTCTTAGATCACCCGTACGTATTTGACGACTTATTTCATATGTAGCGTTTTCAATAATTTGTTCTCCAATAAGAGCTATAGAAGTCTCAAATATAGCAAA

>B488_04500|502497..502398|Liberibacter_crescens_BT-1_EZ-Tn5_Transposon_Disruption

CGTAGAGAACTGACAACGGCCAGCAAGAGAACCATAACAATAATAATCATAAAATAATAATCTGTCTTTACTCTATCACCGTTGTGGAGGCCATAATCAA

>B488_04540|510932..510833|Liberibacter_crescens_BT-1_EZ-Tn5_Transposon_Disruption

ATTTAATAGATTGTGAGAAACAACAATAGCAGCAGAAATATCAATAACAGACCCTTTATTAACTTCCTTTGTAACAACATTGGCAACATCAGCAATAATA

>B488_06570|726686..726587|Liberibacter_crescens_BT-1_EZ-Tn5_Transposon_Disruption

CATGTTGCCAGAGACTCAACAGAAGAAGTACAAGCAATTATAAATGAAATTGAATGTCTGCGCTATAAGGAGCATCCCTTGAGTCGTATGGCAATCCTCG

>B488_02070|241582..241681|Liberibacter_crescens_BT-1_EZ-Tn5_Transposon_Disruption

GATTTGGTGTTGGCACAACAGTTTTTATATGATGTGTTAAATATACCAGCTGATTCATCTGTTGCTACATGGATTATACGTACTTTTGGGGTTTTGACTG

>B488_06360|697110..697013|Liberibacter_crescens_BT-1_EZ-Tn5_Transposon_Disruption

CCCCCCAAGGCCCTCCATTATCGCCACTTTGATTGCTCCAAGGCATTAATACCTCTTAAAAGTTTACTTCATTTAATATTGATAATCGTTAAAATAATTA

>B488_02150|249346..249445|Liberibacter_crescens_BT-1_EZ-Tn5_Transposon_Disruption

ATATAATTGTACTTCATTACAAGATTTTTTGTTGCTGGTTTTTATCTTGCTAAAGAGATCTTTCGCGTTTGTTAGATTGTTAGAGTTTGTATCTTCTAGG

>B488_01950|233653..233752|Liberibacter_crescens_BT-1_EZ-Tn5_Transposon_Disruption

ATCCTAACCATGTATTCATATGGTACGGAGTCATTTTAGCCATTCTTATGCTTGGGAGCATGTTAGGCTTTTATCAAATTTTTTCTCCTCTATTTTATGT

>Intergenic_Region|12253..12352|Liberibacter_crescens_BT-1_EZ-Tn5_Transposon_Disruption

CAGTTAAAATATTATGATGAAGTTTATTTTCTTTCGGTTGAAAATATTTCATCACTCCTTTTGGAGGAGACACTAGATAAATACCATGTTATTACATCCA

>B488_12790|1394080..1393981|Liberibacter_crescens_BT-1_EZ-Tn5_Transposon_Disruption

TTGCAGAAGAGATCGCATCACAATCTGCTCCTCTTGCAGAGGAGATTCTTATTACTACAGCTGAGTTATCATTTTTACCTGAAAGACAAACGGCTTTTGA

>B488_08250|898799..898700|Liberibacter_crescens_BT-1_EZ-Tn5_Transposon_Disruption

GTATATGCTTCAACAGAACCTATGCCGTATATGCAGGAAACGGAAGCAATAACAATACAGTCATCCCGTTCAAGGAGAGAACGTGTTGCAGAATGTCGCA

>B488_07050|782800..782701|Liberibacter_crescens_BT-1_EZ-Tn5_Transposon_Disruption

TCATAGAACTTTGTATCACCGAGCTTCCAACCCTGGTCTACCAGACGGTCGATGATGTATTGCTGGAAGTGTTTTTCCTGATGTGCTGATGCTCGCATTT

>B488_05170|558954..559053|Liberibacter_crescens_BT-1_EZ-Tn5_Transposon_Disruption

GCGTACCACTTTCGCAAATGGCAAATTTCAAAGACTATCTTCTTGATCTACAGACGGCGGCTTCTTCTCATGACCTCAATAGAGCTAATGCGGCTATCGA

>B488_03660|419051..419150|Liberibacter_crescens_BT-1_EZ-Tn5_Transposon_Disruption

CTGTACAGGCAATGCTTTTGCAGGAGCGGTTCAGTTTGAGCGTATAAAAGCAAAGGAGAAACCTGATTTTATCCCGTCAAGGCTGTTTATTTATTATAAT

>B488_03860|450215..450310|Liberibacter_crescens_BT-1_EZ-Tn5_Transposon_Disruption

ATCCTAAATTTGGTATGTGTTGTACGTGCGGATCCCAGATAAAAAAAGAATCATGTTGTGTTTTATCATTTCTAAGAAAAAATACGGGTATACTTGATCC

>B488_09220|993766..993852|Liberibacter_crescens_BT-1_EZ-Tn5_Transposon_Disruption

TTGTTATACAAGTCTCGTGCATGCAAAGCAGCCATTTGGAGGTATATTAACAGAACAACAATATTATTCGTTGATCCTTCAACTCAGCAAAAGTTACAGA

>Intergenic_Region|418388..418289|Liberibacter_crescens_BT-1_EZ-Tn5_Transposon_Disruption

GGTTACAGACTTACCCATTATAGATCCTTGCCTATATTATTCAACCTTGAAACATCTGAAGAAGAAGTTGCTATACACAGAAACCCTTATAAACTTATTG

>Intergenic_Region|687000..686946|Liberibacter_crescens_BT-1_EZ-Tn5_Transposon_Disruption

TTATTATTCAGACAGGACGCCATATGATCCTTCAACTCATCAAAAGTTCACGTCTTGGTTAATTTCTATAGAAGAACAATATACATTCAACATATTTAAA

>B488_03110|357422..357521|Liberibacter_crescens_BT-1_EZ-Tn5_Transposon_Disruption

ACTTTATATAGTGAATACTAGTCTTTTAATAAAATACTTTCTTAATTTTTAACTTGATAGTGTATTATTTTTCCCATGTTGTAAATCGTTGGCCATTGAT

>B488_11420|1239650..1239749|Liberibacter_crescens_BT-1_EZ-Tn5_Transposon_Disruption

ATGCAGGACAATTAGAAGAAGCAATTACAACCATTGACCGAGCTCAGACTCCTGATAGACCTAACTGGGAATTAATGTCTGCAAAAGGTTCTATTTTAGA

>B488_11420|1239650..1239749|Liberibacter_crescens_BT-1_EZ-Tn5_Transposon_Disruption

ATGCAGGACAATTAGAAGAAGCAATTACAACCATTGACCGAGCTCAGACTCCTGATAGACCTAACTGGGAATTAATGTCTGCAAAAGGTTCTATTTTAGA

>B488_12820|1397712..1397811|Liberibacter_crescens_BT-1_EZ-Tn5_Transposon_Disruption

GCAGCTAAAAGTGAAAGATTTTCGGCACAACGAACAAGCAAACGATCAACGAAAACCTCATCAAGTCGATCAGGAGAATAAATAGCATCTGATATTCCTT

>Intergenic_Region|330549..330648|Liberibacter_crescens_BT-1_EZ-Tn5_Transposon_Disruption

CCCAAGAGTCCATATCGACGGGGTTGTTTGGCACCTCGATGTCGGCTCATCGCATCCTGGGGCTGAAGCAGGTCCCAAGGGTTTGGCTGTTCGCCAATTA

>B488_00460|59117..59216|Liberibacter_crescens_BT-1_EZ-Tn5_Transposon_Disruption

GTCTTAAGGTATATAGTGTTAAAAGCAAGCATCAATTCCTAACTTTACCTGAAGCTTTTTTCATGGGATTATATGAAATACATGATATTGTTAAAGCTAC

>Intergenic_Region|1219442..1219345|Liberibacter_crescens_BT-1_EZ-Tn5_Transposon_Disruption

ATGTATGATATAAGGAATCATCTTTATTGCGAAGAAATTGATTTAATCGCGATAAAGGTATATTGTTTTTTTTTACTTTTTTTGAAGTACTTTTCAAATC

>B488_11860|1295521..1295620|Liberibacter_crescens_BT-1_EZ-Tn5_Transposon_Disruption

GTTTAATTCAGAAGTATCAAAACCCGCAACAGCCATTGTTATACGATAAAGATTTTCATCTGTACGCTCTATGTTGTATGGTGGATACGTAACAGACTGA

>B488_07030|781363..781462|Liberibacter_crescens_BT-1_EZ-Tn5_Transposon_Disruption

GACCTGTGGACTTCAATCCGTAACGAAGGTAAAAAACGCCGCATTGTCAGTGATGACCAACGAGCCCAGATTTTGGAGGTCTACGCCGCTGGGGAAACCA

>B488_08600|943527..943626|Liberibacter_crescens_BT-1_EZ-Tn5_Transposon_Disruption

GATTAATCGTATTGATATAACTTCTTGGAACAACAGAAAAATTAACAAAAGGGGGAGCCATCAATGCATTATTGGTGTTAATTTGCTCTGTCACCGCAAA

>Intergenic_Region|500558..500657|Liberibacter_crescens_BT-1_EZ-Tn5_Transposon_Disruption

CTTTAACACGTACCATAGTATGTAAGTATTTCATTTATCTTTTCCCAAATAATTTTAATGGTTAAAATTGTATTTTCTTATAATGCAATTATATTTTATA

>Intergenic_Region|339189..339249|Liberibacter_crescens_BT-1_EZ-Tn5_Transposon_Disruption

GGCCAGATCAGACACAAAGTTCTGCCCGGCCTGCTTTATCAGAAATAACTGTATGATCCTTCAACTCAGCAAAAGTTCCACAGATGTTCACTAGCTAGGC

>B488_10370|1132670..1132751|Liberibacter_crescens_BT-1_EZ-Tn5_Transposon_Disruption

GTTAATAGCCTTTCTGCAAAGCCTTAGCATAAGGACCTAATGCAAACTGCTCCACATTACTCAAAAAAATCTTTTGATCCTTCAACTCAGCAAAAGTTAA

>B488_04010|465593..465494|Liberibacter_crescens_BT-1_EZ-Tn5_Transposon_Disruption

GTTTTAAATTGAAAAAACAATTCTTCTGTATAATGGTCTCTACCAGGATATTGAAGTTTAAAAGCACCCAAAGAATACTCATCCATCAACCAATCATAAC

>B488_08350|911375..911474|Liberibacter_crescens_BT-1_EZ-Tn5_Transposon_Disruption

GCATTACATCTTTACTCATCTTAAGGAAGCACATTCAATGAAGCTCTATTTTACTCCTGGTTCATGCTCACTTGCCCCTCATATTGCATTACAGGAATCT

>B488_09390|1011457..1011556|Liberibacter_crescens_BT-1_EZ-Tn5_Transposon_Disruption

ATATTGAAGTCCATAACATCCCCTTTTGAATCCCGCTTTAATGCTTGATGAGAGACCATATCCCAATGGCTTCCTAATCTAAGGATACGCAACACACCAT

>B488_09870|1074564..1074622|Liberibacter_crescens_BT-1_EZ-Tn5_Transposon_Disruption

GGTATAGGAGATGTTTCAATCTTTGGATCTGGCTATGCCATGCGTATCTGGCTTGATCCTTCAACTCAGCAAAAGTTCTAATTTCTTTACTTACAAAAGT

>B488_05630|611832..611931|Liberibacter_crescens_BT-1_EZ-Tn5_Transposon_Disruption

GCTTTGGAACGCGCTAATAAAGCAACAGTACAGGCTGCACAAGCCGATCTTAAGCTATCTGAAATTAATCTCAATAAAACTCGAATTATTTCGCCAATCA

>B488_03950|458694..458793|Liberibacter_crescens_BT-1_EZ-Tn5_Transposon_Disruption

ATTGAAAACAGTAACTGAATTCATAGGATCATCATCAAAATAGAACATACTGGTCATATTGCCATTAATAATTGCAAAAGCAATATTTTTAGTGTTAGCA

>B488_05040|547381..547282|Liberibacter_crescens_BT-1_EZ-Tn5_Transposon_Disruption

ACAGAAGGCAACAAAGACGCAACAATCTTCATATATAAACCAGGAGCTTCTTCACGAACACGCGCAATAACCTGTATGCCTTCCGTTTCAAAATCACAGC

>B488_11900|1300408..1300371|Liberibacter_crescens_BT-1_EZ-Tn5_Transposon_Disruption

GTTGTAGGGTTCCCAAATTACGTAATAATTGTCCGCGTTGATCCTTCCCTGCGCGCCAGTTCGCTTCGCAAACATAAATGTTGCCTAGTCCAGCGACCAA

>Intergenic_Region|1032561..1032462|Liberibacter_crescens_BT-1_EZ-Tn5_Transposon_Disruption

ACTGGAAATAGTTTAAGGAGAACTTTTATCACGATGATAATTTGGTGCTGAACCAGATTATTAGGAATTATTTAAGTTTATGCAGATCAAGCTCATTTTC

>B488_09580|1031362..1031461|Liberibacter_crescens_BT-1_EZ-Tn5_Transposon_Disruption

CCCTACAATAGGAGTCGTCGGAGCTCGTAACGCATCAATAGGAGGATTAAAATTTACAACTTTTATATGCCAAGGAATTATTAAAGCTGGTTATGTTATT

>B488_06290|692083..692129|Liberibacter_crescens_BT-1_EZ-Tn5_Transposon_Disruption

ATTCAAGGCTCCGCACATTACTATAAAGGCGCCTAAAGATGATCCATCAACTCTTACAGTCTTGTGAAGCAGAACCTTTATCATCTGCAATCCTGTCTGG

>Intergenic_Region|974380..974281|Liberibacter_crescens_BT-1_EZ-Tn5_Transposon_Disruption

GTGCACGGGCATCAACAATATGTACATGAGTACTGGCGAAGACCGCCAAGCCATCATTACGGTTATTATAGAAGATAACTATAGTATTGATTTCTCAATT

>B488_06570|726352..726253|Liberibacter_crescens_BT-1_EZ-Tn5_Transposon_Disruption

TACATAATCTCTCCATGTTGAATGCTGCCAAAGACATGATCGAAACTGAAGAGTTACCAAAGCAACGTAAGGCTCCTCTAGCCAATCTCCTCGAAAATAT

>B488_13110|1422787..1422688|Liberibacter_crescens_BT-1_EZ-Tn5_Transposon_Disruption

CGTTTACCTATAACCTGGTGGATCAGCTGCGTGCCAATGGTCACCGGGTAGTGATTTACCGCAACCGGCTACCGGCTGCGCTCATCACGCGGGTGCTGTC

>B488_04540|512151..512250|Liberibacter_crescens_BT-1_EZ-Tn5_Transposon_Disruption

ATTTATATGCATGCAACGCCACAGGTAAATCTCTTTAAGAGATCTATGCGGGCTTTAAGCCATGGCTGTATTCGTTTATCTGATCCACGAGCGATGGCTG

>Intergenic_Region|329076..329172|Liberibacter_crescens_BT-1_EZ-Tn5_Transposon_Disruption

GCGCTGGATAGACTTTGGGGGCTCACCGCCTTACTGCTCCTAACCAAACTCCCAATACCGACGAGTACTACTTGGCATACACCCGGCGGGTGCTAACGTC

>B488_06480|718510..718412|Liberibacter_crescens_BT-1_EZ-Tn5_Transposon_Disruption

GTTTAACATCTTCTTGGCTTTATCAAGAGCCTTCGCAGGACTATCCCATCCCATTCCATATTTAACATTTAAGTTCTTCGGAAAATTCTTTTGCTAGCTT

>B488_01560|188652..188751|Liberibacter_crescens_BT-1_EZ-Tn5_Transposon_Disruption

ACGCATGACAGAGCAACGTAAGTTAATAGCGCGTATTTTAGAAGAATCTGAAGATCATCCAGATGTCGAGGAGCTTTATAACCGATCAGTAGCTTTTAAT

>B488_09960|1089199..1089150|Liberibacter_crescens_BT-1_EZ-Tn5_Transposon_Disruption

ATTCGAGGCAGCCCATATTACAGAAGAAGCACGTAAAGTTGATCCTTCAACTCAGCAAAAGTTCCATAAAATGATTAAGTACCATGGTTTAAATCAAAAT

>B488_10240|1119285..1119384|Liberibacter_crescens_BT-1_EZ-Tn5_Transposon_Disruption

CCGTAATATATCCCGTTCCTTTTATAGGCATAGGTGTTTGAATAGTATTCACAACTTCTTTTTTATTGACGTCAATAACAGAAACTATTCCATCTCCTCC

>B488_09960|1089199..1089150|Liberibacter_crescens_BT-1_EZ-Tn5_Transposon_Disruption

ATTCGAGGCAGCCCATATTACAGAAGAAGCACGTAAAGTTGATCCTTCAACTCATCAAAAGTTCCAAGAGGAAGCTGATTATTTGGTTTTGTATTTTAAT

>B488_13370|1454210..1454309|Liberibacter_crescens_BT-1_EZ-Tn5_Transposon_Disruption

ATTACGTATTTTAAACCAATAATTTTGTACATTAGATGGTTTTACATTAGGCTTAAGTTCTACAAAAACAACAGTTCCTCCTGCTTTTGTCAAAGAACGG

>B488_11700|1276983..1276884|Liberibacter_crescens_BT-1_EZ-Tn5_Transposon_Disruption

ACGCCCTAAGGGTGCGTCAATACTTCCAGATATTGTAGAAAGGTTACCCCAAACAATAGCATAATATGAGCGCTCTAGTCCCATGGTAAGACCGTGGTCA

>B488_01290|159194..159095|Liberibacter_crescens_BT-1_EZ-Tn5_Transposon_Disruption

ACCAACCACTGTCATAATGCATGGTGTAGATACAAAACGTTTTTATCCTACAAAAAATAAAACATATGATCGACATGTCATAGGAATGCCTGATGATATT

>B488_07300|815685..815586|Liberibacter_crescens_BT-1_EZ-Tn5_Transposon_Disruption

TTCTCGATCACTCCACGCATTTTGTCAACAATATCATTTAAGTTTAATTTGTTTGTCATCGATGTAAGATTTTCATTTTTTGAAATACTAAATGCATTTG

>B488_12080|1320449..1320548|Liberibacter_crescens_BT-1_EZ-Tn5_Transposon_Disruption

TTATAGCACGAATAGATGAAATAACTCCTGAAGTAGAGGCTATGGCAAGAGAAGCTGCTTCAGAAATGTCATGTGGTGGTATGAAAACAAAAATTGATGC

>B488_13130|1427632..1427731|Liberibacter_crescens_BT-1_EZ-Tn5_Transposon_Disruption

AGATAGGACCAGGTATCGCTAACCATTCCTATGGAATACAGGTAGCAAAATTAGCAGGATTACCACTTTCAACAATCGATCGAGCATATCATATCTTAGA

>B488_07600|844636..844562|Liberibacter_crescens_BT-1_EZ-Tn5_Transposon_Disruption

CTTGATAACGGCTGTTTCAATTGACCTGCTTCCAATGCTGCCCTGGCAGTCGTTATGAGCGACCTACATGGCCCCCTGAATACCCTCAAGACCCTAGCTT

>B488_02360|269506..269605|Liberibacter_crescens_BT-1_EZ-Tn5_Transposon_Disruption

GGTAAAGTTCCTGCGCGTTTCTCTGCCTCTAAAGATGCTGCTGCATCGGCGTAAGGAGCTTGTCGAGCAACACTCCAGTAACATAGCTCGTGGAGTGGAA

>B488_08880|966548..966647|Liberibacter_crescens_BT-1_EZ-Tn5_Transposon_Disruption

GCAGCAAGGCAGGCCTTCAGCTGACTGAGTTGAAGGGGCAATCGTTCATATCGGGCAATCCGATAAGCAAGAGCATGCTGATACGCTGTTTCTGGTGGTT

>B488_11590|1258500..1258401|Liberibacter_crescens_BT-1_EZ-Tn5_Transposon_Disruption

GTAGTGTCTCGACCAAGTTGTTCATCTATTGCTTTTGAAGAAATTATTTTAACAGGGAAATGATTGTCATCACTTGGGGTCCATTTTAAACGTAACATAG

>B488_11700|1276983..1276884|Liberibacter_crescens_BT-1_EZ-Tn5_Transposon_Disruption

ACGCCCTAAGGGTGCGTCAATACTTCCAGATATTGTAGAAAGGTTACCCCAAACAATAGCATAATATGAGCGCTCTAGTCCCATGGTAAGACCGTGGTCA

>B488_05060|550292..550239|Liberibacter_crescens_BT-1_EZ-Tn5_Transposon_Disruption

CTTTTGTGCCACTCCCCAATCCCGTATTGACTGTCACATCCATGTCCGTATTCCTCACCTTTGTTCTCTTCAACATCTTCTGCCTCCTCCTGTTCTTCGT

>B488_09530|1025102..1025201|Liberibacter_crescens_BT-1_EZ-Tn5_Transposon_Disruption

GGCCTTAACCCAAACATCATCTTTTACTCTGAGATAATTAGCAACCGCTACAGGTGTTCCTGGTGCTCCAGATCCTCCAGATGTTCCAGCAGCTATCGTG

>B488_13430|1460470..1460372|Liberibacter_crescens_BT-1_EZ-Tn5_Transposon_Disruption

GAGTAGCGGCATCATAGCTATAGCCCGTATCAAATCAATGCTGATCATCAACAATAAGTGATCCTCCAACTCAGCAAAAGTTCAAAAGGAATAGGCTTCC

>B488_03660|419051..419150|Liberibacter_crescens_BT-1_EZ-Tn5_Transposon_Disruption

CTGTACAGGCAATGCTTTTGCAGGAGCGGTTCAGTTTGAGCGTATAAAAGCAAAGGAGAAACCTGATTTTATCCCGTCAAGGCTGTTTATTTATTATAAT

>B488_00010|1651..1552|Liberibacter_crescens_BT-1_EZ-Tn5_Transposon_Disruption

CTGCTGGACCTTTTTTAAGATTTAACATACGAAATTGTATCCCTGCTACATCAGAAACACGACCCATTAAACCATCTAAAGCATCTATTTCCCGAACAAG

>B488_07060|786304..786228|Liberibacter_crescens_BT-1_EZ-Tn5_Transposon_Disruption

GCGCACGAAAGGTCATACCAAGACACTGTTGCCGGATAACGTCCACCAGACGCCGGGTTGCCCGACGTTTATGATCCTTCAACTTCATCTCCGGTCTGAA

>B488_06930|771533..771619|Liberibacter_crescens_BT-1_EZ-Tn5_Transposon_Disruption

ATACAAAACCATAGACATCATAAAGTTGTTCAAATGAGGTGATTTCGATTGCATTTTGCAGAAAATAAAATAAGCCCTCTTTTGTGATCCGTCAAGTCAG

>B488_05060|550292..550193|Liberibacter_crescens_BT-1_EZ-Tn5_Transposon_Disruption

CTTTTGTGCCACTCCCCAATCCCGTATTGACTGTCACATCCATGTCCGTATTCCAGACACGCGGATCAATCTTGACCCACTGTTTGCGAAGCCTTACTGT

>Intergenic_Region|974380..974281|Liberibacter_crescens_BT-1_EZ-Tn5_Transposon_Disruption

GTGCACGGGCATCAACAATATGTACATGAGTACTGGCGAAGACCGCCAAGCCATCATTACGGTTATTATAGAAGATAACTATAGTATTGATTTCTCAATT

>B488_05430|587094..587193|Liberibacter_crescens_BT-1_EZ-Tn5_Transposon_Disruption

TCCCAGGATGGAATGATAGGTAAGCGTTCTCTATCATCGTCATTTAGATCTGTGTAGTATAGGAGACAATGTCTCCAGAGGGTATAGCCCAATATTGCTC

>B488_10810|1179396..1179495|Liberibacter_crescens_BT-1_EZ-Tn5_Transposon_Disruption

TATCTCTGCTGTTCAATTTCATCCCGAATCAATCATGACACTTGGAGACAATGCTGGTATGCTTATGATAAGAAATATAGTCTCTAACATCACAATAAAA

>B488_12840|1399943..1399844|Liberibacter_crescens_BT-1_EZ-Tn5_Transposon_Disruption

CTCTTGAGCAAGCTCGTGTAATTCGTACTCTAGCTGAGCCAACTTTGACTGCTGTTTCAGGTCAAAGTGCTAGTTTTAGGTCTGGTGGTGAGCGTTTATA

>B488_08350|911375..911474|Liberibacter_crescens_BT-1_EZ-Tn5_Transposon_Disruption

GCATTACATCTTTACTCATCTTAAGGAAGCACATTCGTTGAAGCTCTATTTTACTCCTGGTTCATGCTCACTTGCCCCTCATATTGCATTACAGGAATCT

>B488_09450|1016239..1016338|Liberibacter_crescens_BT-1_EZ-Tn5_Transposon_Disruption

GCATTGCTCCAACCAGCCTATTGAATTCTGTCCATATCGCAGCAAATTGGTAAAAGTATCTATTCCAGCTGTAGAATCGAATTTAATATCAACATTCATT

>B488_05170|558477..558378|Liberibacter_crescens_BT-1_EZ-Tn5_Transposon_Disruption

CCTTTCGAAGCCGCGTAGTTTTTAAGTCCTGTCCAGTTTGAGGCACCACCACCATCATGATTCTGGAAAAAACCACCTGAAATACCGTTAACATCCCACT

>B488_05910|650520..650421|Liberibacter_crescens_BT-1_EZ-Tn5_Transposon_Disruption

CGTCACTATGCTATAAGGTGGAACATCATGAAGAACAACACTTCCAGCTGCTATACGAGCCCCTGTACCTATTTCGATATTGCCAAGGATTATTGCAGCT

>B488_11940|1304303..1304402|Liberibacter_crescens_BT-1_EZ-Tn5_Transposon_Disruption

ACCCGATACTCTTCGGCTCCATAAGAGAAGGAGTACCTCCGCCAAAAAAAATGCTGGTAATTGTTCTAGGACCACTTAATTGTCGCATCCGAGTCATTTC

>Intergenic_Region|546247..546148|Liberibacter_crescens_BT-1_EZ-Tn5_Transposon_Disruption

GTCTTACAGTGTCAAAGGTAAGGTACTTGGGGGTTCACCTAACATTAAAGTTAGCGATAGAATCTATGAATTAACCCAGTTAGAAGTTGAAAAGCTTACT

>Intergenic_Region|432279..432378|Liberibacter_crescens_BT-1_EZ-Tn5_Transposon_Disruption

GATAAGGATACTGTTCAGAAGCAAGATTCTGCGGATAAATCTGTTAAGGTCGAAACTCCTCCTAGCGAAGTTGTTGCTTCTACGGAGACTTCTGATAAGA

>B488_10060|1101188..1101089|Liberibacter_crescens_BT-1_EZ-Tn5_Transposon_Disruption

ACTTAATGCCCTCCCTAGGGTTTTTTTTTCTGAACTATCACCAATATTATATACAAATCCAAAGGCATCTATACAAAGAGCTTATTATACTTTAACAGGG

>B488_11140|1213312..1213213|Liberibacter_crescens_BT-1_EZ-Tn5_Transposon_Disruption

CTACAAGCCTGTAGTATTCTAATTCGTTGGGAGAAAATAGGGAAGGGCTGGTTGTAAAAATTATAGCATTGGAAAGAGATGAGCATGTGCGTACTTTAAT

>B488_11900|1300188..1300089|Liberibacter_crescens_BT-1_EZ-Tn5_Transposon_Disruption

TAACACGGTACAGCAATGCTTCCTTTTCAAGATACAGTATAACGTGATCCTGCAACTCGTCAAAAGTTCGTGTCTGATCAGTTTTTTCTAAGCCATCTTT

>B488_05170|558477..558378|Liberibacter_crescens_BT-1_EZ-Tn5_Transposon_Disruption

CCTTTCGAAGCCGCGTAGTTTTTAAGTCCTGTCCAGTTTGAGGCACCACCACCATCATGATTCTGGAAAAAACCACCTGAAATACCGTTAACATCCCACT

>B488_11140|1213543..1213444|Liberibacter_crescens_BT-1_EZ-Tn5_Transposon_Disruption

GAGCTTCATGGTATACCTCTTTTGATCCTTAAACTCAAAAAAAGTTCCCTTGTTCTGCTCGTTCACCTTTCCAGTTCGTTATAATACCACCTGCTTGTTC

>Intergenic_Region|1013306..1013405|Liberibacter_crescens_BT-1_EZ-Tn5_Transposon_Disruption

TAACAAGCGATTGAAACATATTTTTTATACCTCTTCATGAGGGAAGGTATGTCCTGTAAAATAGATTACAGGAATAAAGATTAGAATGATAAACGATCAA

>Intergenic_Region|437314..437402|Liberibacter_crescens_BT-1_EZ-Tn5_Transposon_Disruption

CCATTAGGTCATCGTGTAAAATAAGATAAGATATTGTTATAAGAAATGTAATTGTTAGTGCCGTGAGTAAGCAACAGCTTATCTTGATCCTTCAACTCAG

>B488_13130|1425366..1425465|Liberibacter_crescens_BT-1_EZ-Tn5_Transposon_Disruption

GGTGAGTTTTATGAACTATTTTTTGATGATGCCTTAGAAGCTTCTCGTTCTCTTGGAATTGCTTTAACAAAACGTGGTCAACATCTGGGTCAAGATATTC

>B488_13420|1458984..1459083|Liberibacter_crescens_BT-1_EZ-Tn5_Transposon_Disruption

GCTTATAAGTCTCCACGGCCAATATATCTGCAACAAGCGCAAGCGTCTTGAGGCGTTGGCCGAACCGGGTGAAATTCTCAAAAACATCGGATTGTATGAT

>B488_09220|993766..993852|Liberibacter_crescens_BT-1_EZ-Tn5_Transposon_Disruption

TTGTTATACAAGTCTCGTGCATGCAAAGCAGCCATTTGGAGGTATATTAACAGAACAACAATATTATTCGTTGATCCTTCAACTCAGCAAAAGTTCCAAA

>B488_04720|528521..528422|Liberibacter_crescens_BT-1_EZ-Tn5_Transposon_Disruption

GTCATAAGCTGGTCTGTAAAAATACCGTCAACTTCAGGTTCTTTCCTAAGATGTTCACGAGCCATATTTTGCAAGGCATTGGCATTATTTGAATTGATAT

>B488_03820|444092..443993|Liberibacter_crescens_BT-1_EZ-Tn5_Transposon_Disruption

TTTCTATAGACCAAAAAAATACTTCACACAATCCACGTTCTACTGTTGGTACGACAACTGAAATCTATGATTACATGCGATTACTTTTTGCTCGTATAGG

>B488_08300|907868..907967|Liberibacter_crescens_BT-1_EZ-Tn5_Transposon_Disruption

ATGCTCGTCAGCCCTTGACAAAGACTGTTCATAATGTTGAAATTTTTTCAAAAGCCTTGGCAGATAATGCAGACAATATTGACCAGTTATTAAAGAGTGT

>B488_09390|1012063..1011964|Liberibacter_crescens_BT-1_EZ-Tn5_Transposon_Disruption

GGACTACAGTTGCATCCAGTAATCTTCGTATTGGAGAAATATTGGTTATCGTTGGTTCAGGAGAGAAACCGAGTTTTCCAGGAGAAGAGATTCGTGAACC

>B488_05440|588150..588201|Liberibacter_crescens_BT-1_EZ-Tn5_Transposon_Disruption

GTGTATAGCCCAATATTGGTCCAGGAGAGACGCTATAAATAACAGTGATCCTTCAACTCAGCAAAAGTTAAAATTCACTCAGTAGAATTAGAATACGGCC

>Intergenic_Region|851197..851098|Liberibacter_crescens_BT-1_EZ-Tn5_Transposon_Disruption

ATGATACGGTTGAGAACCCGTGTCTCTCCTGTCATAAAACACAACACAGCTTTTGAACACTCTTTTCATAACAGGATTTTCCCGCAAGGAAAAGAGTGTT

>B488_11210|1221307..1221393|Liberibacter_crescens_BT-1_EZ-Tn5_Transposon_Disruption

GTCTTTGGTACACTATTTTTAGGTGGATTTTTATGGTTTTCTATATTAATTATGAACATGGACATACCAGATGATCCTTCAACTAACCAAAAGTTAGTAC

>B488_13590|1479930..1479831|Liberibacter_crescens_BT-1_EZ-Tn5_Transposon_Disruption

GTACCTCCAAACATACCTGTCAATATAATCACACCTTTTCCTTCATCTACATTTTCTATGGATTTTATCATATCTTGACGACAACGTTCTAAATCATCCG

>B488_00700|84213..84114|Liberibacter_crescens_BT-1_EZ-Tn5_Transposon_Disruption

AACCCATGCTATAAGCATCGTATCCATGACATGATTGATATCGAAGGTTTTTGGCGTAGCTATCTTATTACTTTTACTAGCAAACAATATCTTTTGAGCT

>B488_11990|1308536..1308437|Liberibacter_crescens_BT-1_EZ-Tn5_Transposon_Disruption

GTGTTGGCAATGGTGGTGCAAAATGATCACTGGAAAGTTTATGGATAAGTTCTTGAGGATTAATTCCCCATTCACGAAATATAATGCCATACACTAATGC

>B488_11810|1288888..1288987|Liberibacter_crescens_BT-1_EZ-Tn5_Transposon_Disruption

ATCAGGACATTGGAATCGATAACCACATACCCGGCATAAAGTTAAAGGAGCATAACCACGCCGATTTAGAAAAAGAAGGGTTTGCTCTCCTTTCTTCAAG

>B488_05160|557049..557148|Liberibacter_crescens_BT-1_EZ-Tn5_Transposon_Disruption

CTGAACTATAGCCAGACAGGAATGACAAGCATTACAGATCCCTATACGGGGAAACAGATCAGCATTCCCCAATATACAGCGACACAGACATTATCGCCGC

>B488_05230|564645..564744|Liberibacter_crescens_BT-1_EZ-Tn5_Transposon_Disruption

GTATATCATTGATAACATTTAAGAGGTGTTTCCCACTATCATGGATATCTTTGGCGTATTCATTATACTTTGTTGACCCTAATGGTCCAAACATTTGATC

>B488_12830|1399106..1399074|Liberibacter_crescens_BT-1_EZ-Tn5_Transposon_Disruption

GATTATCGTAAGAGACATAAAATTGTTCTGAAA

>B488_07980|874070..873994|Liberibacter_crescens_BT-1_EZ-Tn5_Transposon_Disruption

TTCCAGGATAACCCTGGAGGGCGATGCAACAACGGTTATTATTGATACAGGTCCTGATTCCCGCGCCCAAATGATCCTTCAACTCAGCAAAAGTTCGACG

>B488_13280|1441931..1442030|Liberibacter_crescens_BT-1_EZ-Tn5_Transposon_Disruption

CTCAACCCCTGATCAACTTTTACCACCATCAGCTCCTTTTCTTGCACACAAACTTGGACTTGTAAAATCAGGAGCTATTGATTTATCAGGAGCTTGCTCA

>B488_07310|816097..816196|Liberibacter_crescens_BT-1_EZ-Tn5_Transposon_Disruption

GCTTTAATGCTATAACCATGATTATAGAGCAGTATCTTAATTCCTTTTAGAAAATCCATATCTTCCAGTCGATAATAATGGTGACCATCGCTTTTCTTTA

>Intergenic_Region|229459..229360|Liberibacter_crescens_BT-1_EZ-Tn5_Transposon_Disruption

GGCTTAGACCATCACCAAGAACGCTATGGCTGGCATTAACGTAATAAAAACGCTTAAAAACTTTTTCTTTGTCCTCCTCAAGAATTCCAATCCCTGATCT

>B488_12810|1396872..1396971|Liberibacter_crescens_BT-1_EZ-Tn5_Transposon_Disruption

GCTTACAATGCTACTAATTTCTCCACGCGATTCTTCTAAATCATATTGAGAAAGCTGTGAAACATCAATCATGTCAATGAGAGTTTCAAATATTTCTGCT

>Intergenic_Region|500558..500657|Liberibacter_crescens_BT-1_EZ-Tn5_Transposon_Disruption

CTTTAACACGTACCATAGTATGTAAGTATTTCATTTATCTTTTCCCAAATAATTTTAATGGTTAAAATTGTATTTTCTTATAATGCAATTATATTTTATA

>B488_07690|849100..849001|Liberibacter_crescens_BT-1_EZ-Tn5_Transposon_Disruption

GTTATCAGGAGTGCAAAGAATTTTTCTTCTCGCAGGGAGTTTTATTCTTTTCACTCTGTTGGCATGGGTTTTGGGTATCAGGAAAGGTAAAAAAACAGCG

>B488_03240|372915..373014|Liberibacter_crescens_BT-1_EZ-Tn5_Transposon_Disruption

GTTGATAACAATGCTTGTTTATAATGATGAGACTTAGAAGTGGGTGCGTTTTCTACTATATTTCCTTCATACATGACAGCTATATGATCAGCAACTTCTG

>B488_07050|782800..782701|Liberibacter_crescens_BT-1_EZ-Tn5_Transposon_Disruption

TCATAGAACTTTGTATCACCGAGCTTCCAACCCTGGTCTACCAGACGGTCGATGATGTATTGCTGGAAGTGTTTTTCCTGATGTGCTGATGCTCGCATTT

>B488_09390|1012063..1011964|Liberibacter_crescens_BT-1_EZ-Tn5_Transposon_Disruption

GGACTACAGTTGCATCCAGTAATCTTCGTATTGGAGAAATATTGGTTATCGTTGGTTCAGGAGAGAAACCGAGTTTTCCAGGAGAAGAGATTCGTGAACC

>B488_02360|269506..269605|Liberibacter_crescens_BT-1_EZ-Tn5_Transposon_Disruption

GGTAAAGTTCCTGCGCGTTTCTCTGCCTCTAAAGATGCTGCTGCATCGGCGCAAGGAGCTTGCCGAGCAACACTCCAGTAACATAGCTCGTGGAGTGGAA

>Intergenic_Region|328247..328276|Liberibacter_crescens_BT-1_EZ-Tn5_Transposon_Disruption

GCTGTGGGGAGCTGCGATAAGCTTTGATCCTTCAACTCAGCAAAAGTTCCACCTTCACTCAGCAAAAGTTCAGTTGTTACTGAGATTTGATTAAGTTCTT

>B488_13570|1478871..1478970|Liberibacter_crescens_BT-1_EZ-Tn5_Transposon_Disruption

TGCTCATGGTGGTTCTCTTAAACCACAAAATATTATGAATAAAACAACAAATAAAATTATAGGTGCTCACTTCATTTTATCATTGCCTGCAGTGAGTTTA

>B488_06680|741151..741250|Liberibacter_crescens_BT-1_EZ-Tn5_Transposon_Disruption

CTCAGAAACATGGCGGCGGGATTAGCGCGTCCTTCTCCTGCCATGGTTGGAGCACTGCCATGAACAGGCTCAAAATAACACATGCTACTTCCAATATTGG

>B488_11720|1279172..1279073|Liberibacter_crescens_BT-1_EZ-Tn5_Transposon_Disruption

GGAGAGGCAATTATTTTAGATCAAGCGTTATCATCTGAAGGTACTGTTTCTACAGGAATATCTGAAAAGGCTGATGAAAGCCATGGAAACCCTGTACATT

>B488_11140|1213229..1213328|Liberibacter_crescens_BT-1_EZ-Tn5_Transposon_Disruption

GCTCATCTCTTTCCAATGCTATAATTTTTACAACCAGCCCTTCCCTATTTTCTCCCAACGAATTAGAATACTACAGGCTTGTAGAAAACTGTTGTATACT

>Intergenic_Region|957809..957908|Liberibacter_crescens_BT-1_EZ-Tn5_Transposon_Disruption

TTTTTCTACTCCCCTTCCTTCCAGAAAAGATACACAAAAACCTGAAATCATATTCAAACTTCTCAATAAACTCCATTTTTTGTATGAAAAATTACTAACA

>Intergenic_Region|432279..432378|Liberibacter_crescens_BT-1_EZ-Tn5_Transposon_Disruption

GATAAGGATACTGTTCAGAAGCAAGATTCTGCGGATAAATCTGTTAAGGTCGAAACTCCTCCTAGCGAAGATGTTGCTTCTACGGAGACTTCTGATAAGA

>Intergenic_Region|865745..865646|Liberibacter_crescens_BT-1_EZ-Tn5_Transposon_Disruption

CTTAAGTCCCATTGATAATGATCGATGGGACTTTTTCTTTGAGAGAAGGAGTATGTGTTTTTAGATTATGTGGTGTTTTCTCTTTTTTTATATCTTGCTT

>Intergenic_Region|432279..432378|Liberibacter_crescens_BT-1_EZ-Tn5_Transposon_Disruption

GATGAGGATACTGTTCAGAAGCAAGATTCTGCGGATAAATCTGTTAAGGTCGAAACTCCTCCTAGCGAAGTTGTTGCTTCTACGGAGACTTCTGATAAGA

>B488_04740|529568..529667|Liberibacter_crescens_BT-1_EZ-Tn5_Transposon_Disruption

CCAATGAGCGCTGTCATCAAAGTATCAGCAATAGCCGATAAAGAGGCTAGTTACTGTGACCTTTAGGCTTGTTGGGGCCTTTAGTCGTATCTTGGCTCAA

>B488_00090|11929..12028|Liberibacter_crescens_BT-1_EZ-Tn5_Transposon_Disruption

GGCTTATGAAGCATCAGAACAGAAAAAGTTGAAGAATAAGATCGTAAAAAAATATGTTATTGCAGAATTTTTAACCGATAATGATGACAGTACGAATGAT

>B488_00200|26729..26828|Liberibacter_crescens_BT-1_EZ-Tn5_Transposon_Disruption

GTGTATAGTCGATAATATAAAAAGCGAAACGAACAGACGCTTTTTTATTGGTAGAGGTCTTACTATTTCTAATGCTGCTGCTTTTGATAATGATGCCGAG

>B488_10900|1185304..1185389|Liberibacter_crescens_BT-1_EZ-Tn5_Transposon_Disruption

GGCTTGCGGGGTATAGAACTATTTGGTGCTAACGAGTTTCCATCTAGATTATAAATATCTTCTCTAAGTTGTACAACATGATCTTTCAAATACCCAAAAG

>B488_03680|420716..420815|Liberibacter_crescens_BT-1_EZ-Tn5_Transposon_Disruption

GTAAGAGGGAATATGAAATGTCAAAAATCTTAGTAACAGGAGCGAATGGACAATTAGGTCGCTTAGTAATGCATCGTCTTTTACAAAGCATGTCAGCTGA

>B488_08900|967991..968090|Liberibacter_crescens_BT-1_EZ-Tn5_Transposon_Disruption

GCTCTCGGGTCATCGTGATAAAATGCTCTCCTGGCTTTATCAGATGACCATAGCCAATGGTTGGAAGACCTGCAGTGTCCTTGTAGACATGCGCATCAAA

>B488_11140|1213312..1213213|Liberibacter_crescens_BT-1_EZ-Tn5_Transposon_Disruption

CTACAAGCCTGTAGTATTCTAATTCGTTGGGAGAAAATAGGGAAGGGCTGGTTGTAAAAATTATAGCATTGGAAAGAGATGAGCATGTGCGTACTTTAAT

>B488_08340|910818..910759|Liberibacter_crescens_BT-1_EZ-Tn5_Transposon_Disruption

CAATAAAGTAAATAATATTACATTTTTTATTAAATGCATCTTCATAATGTGATCCTTCAACTCAGCAAAAGTTCAGTCTGTCTTTTGATTTCATATTAAG

>Intergenic_Region|865737..865836|Liberibacter_crescens_BT-1_EZ-Tn5_Transposon_Disruption

GGACTTACCAGAGATCATAAAAGCTGTTTTTTAAACTGTTTTATGCAGGCTCTAATATCGTATATGACCACGATATGTTCTTGTTCTTCTTTCGTACACC

>B488_08580|941144..941045|Liberibacter_crescens_BT-1_EZ-Tn5_Transposon_Disruption

GTCTTGATGAGACAAGGGATACATGGCGCTACCTTGTCGGTCTTCTTGTCTTTTTAGGTCTTTTAGGAACTTTTTGGGGATTAATTGATACGATAACTTC

>B488_06090|673427..673526|Liberibacter_crescens_BT-1_EZ-Tn5_Transposon_Disruption

ATGTTGTACTTAACAACATTATCACGAATAGCATCGTTAATGCCAAAAAGCAGATAAGAAAATAATTGTACCGTGATGTCATGGAGTTGCAACTTTATAA

>B488_00320|46285..46186|Liberibacter_crescens_BT-1_EZ-Tn5_Transposon_Disruption

CCTATAGCAACAAGACTTCCCTGCATTGTAACATAAGCATCTGCTGGAAAAACTTCATCTTCATATCCATCTACCAAAATTGACTTACCCTTCCTAATGC

>B488_02520|287606..287508|Liberibacter_crescens_BT-1_EZ-Tn5_Transposon_Disruption

CAGTAAAGGAATAAAATATGTAGGAGAGACCAGAGCTTATTACCCCTAATAGTAAAAAGAAATTATTATCCAAAAATAAATATCAAAAAACTGATATATT

>B488_02150|249337..249436|Liberibacter_crescens_BT-1_EZ-Tn5_Transposon_Disruption

CCCTAAGATATATAATTGTACTTCATTACAAGATTTTTTGTTGCTGGTTTTTATCTTGCTAAAGAGATCTTTCGCGTTTGTTAGATTGTTAGAGTTTGTA

>B488_08300|908258..908356|Liberibacter_crescens_BT-1_EZ-Tn5_Transposon_Disruption

CTGTTGATAATATAACAGTAGCAATCGCCAATGCACGTGAAAGTTCTGCTCCTATAAAAAGCATAACAGAGCAGCTCTACTCAAAAACAGAACGAAATGA

>B488_01410|174252..174175|Liberibacter_crescens_BT-1_EZ-Tn5_Transposon_Disruption

GCTAACCAGCCACCTGTAAAGATGCATTTTCAACCAGGATCACAACTAACACTTGATAACGCTTTAAAAATGATCCTTCAACTCAGCAAAAGTTCCATTG

>B488_07730|851277..851376|Liberibacter_crescens_BT-1_EZ-Tn5_Transposon_Disruption

TTCCTGGACTGCCTTGGCAAGATAGGGAATGATCACTGTAGGATTTGGCGACAAATAATCATCAACCTTATTGACCATCATTGGATCAATCTTCTCAAGA

>Intergenic_Region|688064..688164|Liberibacter_crescens_BT-1_EZ-Tn5_Transposon_Disruption

GTGTAGCAACGACACCAAAGATTACGAAAATAATTTCGATTTTTTACGTCTTCTTGCTGCGCTTATGGTTTTTTGCAGCCACCAATTTGTTTTAGATGGG

>Intergenic_Region|326878..326779|Liberibacter_crescens_BT-1_EZ-Tn5_Transposon_Disruption

GCAGTAAACTGCCCAACAGCTAGCACTCATCGTTTACAGCGTGGACTACCAGGGTATCTAATCCTGTTTGCTCCCCACGCTTTCGCACCTCAGCGTCAGT

>B488_10810|1179396..1179495|Liberibacter_crescens_BT-1_EZ-Tn5_Transposon_Disruption

TATCTCTGCTGTTCAATTTCATCCCGAATCAATCATGACACTTGGAGACAATGCTGGTATGCTTATGATAAGAAATATAGTCTCTAACATCACAATAAAA

>B488_06810|757927..757828|Liberibacter_crescens_BT-1_EZ-Tn5_Transposon_Disruption

TTCCATATCTGAAGGACGTCGCTATCCTGGAATGGAACATTGGTTACCTTTATTCTATGAAAATTTAGAAACCACTTTTTTTTATCTGTCAGATTTTTAT

>B488_06810|756711..756810|Liberibacter_crescens_BT-1_EZ-Tn5_Transposon_Disruption

CATCTCCACAAATTAATCTGTCCATAGGTCGACCGCTGGCAAGATCATTTCGAACAGCTTCAATAGCATTCAGTTGATCTTCTGTTTCATCATAAGGAAA

>B488_11130|1212413..1212512|Liberibacter_crescens_BT-1_EZ-Tn5_Transposon_Disruption

GTTACAGGTACTATAGTTAATTCTTTTGGGAAAAATAGTGCTGGACAGTATAATGATGGCATTGATATTTCTGTCCCTTCAGGTACCGCTATTAAAGCAG

>Intergenic_Region|671207..671306|Liberibacter_crescens_BT-1_EZ-Tn5_Transposon_Disruption

TTATATAACCGCATATTAACTTTTAAAATACCTGAGTTATTTTTTAAATAACTTACAAGTAAATATAGAATCCCTTTATAACATTCTTTTAGATATAAAA

>B488_10270|1123976..1124075|Liberibacter_crescens_BT-1_EZ-Tn5_Transposon_Disruption

AACATGATCTGATATTTCCATAACCACCGACATATCATGTTCAATCAACATTACCGAAGTACCCATATCTTTACAAATACCTACCAATAATTCATTGAGA

>Intergenic_Region|330816..330915|Liberibacter_crescens_BT-1_EZ-Tn5_Transposon_Disruption

GAATGGATGACCGCTGAAAGCATCTAAGCGGGAAGCCAACCTGAAAACGAGTATTCCCTATCAGAGCCGTGGAAGACTACCACGTTGATAGGCTGGGTGT

>B488_12070|1319944..1319846|Liberibacter_crescens_BT-1_EZ-Tn5_Transposon_Disruption

GATCTATAAGAAGCGAGGAACCAATTTTTACTACAATACGTTTATAATCAGTCAGAGAGAATTCTTTCATTCATGATTTATTCTCTCTGGAGAATGACAG

>B488_12840|1399312..1399411|Liberibacter_crescens_BT-1_EZ-Tn5_Transposon_Disruption

GCCAATTGTACCTTGATAAGGTCGCTGTGCCTTTGGAATATCCATACCACTTCTATAAACTTTATGAACACGATTTAAGAAAAAAGAAGTAGGATCACTT

>B488_06110|674729..674828|Liberibacter_crescens_BT-1_EZ-Tn5_Transposon_Disruption

TGTAGTACTGCCACCACGGTGATACCGCGTTGTTGGACCAATGTCAGTAGTTCCTTGCGTCTAGCGGGATCCAGATGGTTTGTTGGTTCGTCTAGCAATA

>B488_13250|1439307..1439208|Liberibacter_crescens_BT-1_EZ-Tn5_Transposon_Disruption

GTACAAGAACATTCTCATGTCTGTGAAGATCTTCTGTAAAGCCATAACCATTCGCACCATAAATACCAGTAGCGTGAGCTTCATCAATGAAAAGGAAGCC

>B488_13250|1439307..1439208|Liberibacter_crescens_BT-1_EZ-Tn5_Transposon_Disruption

GTACAAGAACATTCTCATGTCTGTGAAGATCTTCTGTAAAGCCATAACCATTCGCACCATAAATACCAGTAGCGTGAGCTTCATCAATGAAAAGGAAGCC

>B488_07050|785595..785694|Liberibacter_crescens_BT-1_EZ-Tn5_Transposon_Disruption

GGTGCAGGAGGCGGTGGCGTCGCAGGTGACAAGCCGAAATTCGTCAAAGAGATTATCGAACGGCTCAACAGCCTGTTTGGTGAAGCTACCCCGATTCAGG

>B488_13590|1479933..1480032|Liberibacter_crescens_BT-1_EZ-Tn5_Transposon_Disruption

CTTCTAATCTTTCTATTTCTGTTATGAAACCTGGTTATATTGAAGTAATCGCAGGCATAAATTTACCAATGCTTATAAAGCTTATTGGTGTCAGAGGTGA

>B488_12830|1398731..1398830|Liberibacter_crescens_BT-1_EZ-Tn5_Transposon_Disruption

CTAATATGTTGTCCGTCCATTGCCCACACTCGTGGAGTAGAGAAGGTCTAATTGAAAAATAGCTCATGCGTACCACTTTAACATTCTCATCGCCATCAGA

>B488_13270|1441618..1441519|Liberibacter_crescens_BT-1_EZ-Tn5_Transposon_Disruption

ATGTAGTACAATAAGGAGGCATTAAATAGATTATGTTCCCAAGAGGACGAATAAGAAGATTTTTTTTTCGAAAGAAAGTGCGGAGTTTTAATCCGACATC

>B488_01520|186875..186776|Liberibacter_crescens_BT-1_EZ-Tn5_Transposon_Disruption

GTTTATACTGTTGTGGGTTTCTCTATCACAAATTACCAAAAAAGTAAAAATATTTGGACGCATTAGCCTTAGTCGAGCAATGAATAAAAAGCGAAAGGAA

>B488_00200|27823..27922|Liberibacter_crescens_BT-1_EZ-Tn5_Transposon_Disruption

GTAATGATCCTGTTATTAATCTTTCAGGTCAAGCATTTTTCATCTACGATAAACAGAATTCTTCTGTGTTTACACCTCTTCCCGCAATTTCTAATAGTAA

>B488_07280|812021..811922|Liberibacter_crescens_BT-1_EZ-Tn5_Transposon_Disruption

TTGTTGTTCTTGTTATCTTTATCGAAATCTATCGTAAAATCTGCTTTTAACAGAACACATTGCTCTTTTTGGCAGAAGTTAACTTCAACATGTAAATTGA

>B488_00200|27823..27922|Liberibacter_crescens_BT-1_EZ-Tn5_Transposon_Disruption

GTAATGATCCTGTTATTGATCCTTCAGGTCAACCAATGTTCATCTACGATAAACAGAATTCTTCTGTGTTTACACCTCTTCCCGCAATTTCTAATAGTAA

>B488_08830|961372..961471|Liberibacter_crescens_BT-1_EZ-Tn5_Transposon_Disruption

ATACTGCTCTCAGTGCCAAGGAACGTTTTACAGCATTACAATCTGGACAAATAGATGTGTTAAGCCGCAATACAACCTGGACATTAAGCCGCGATACCTC

>B488_03860|450215..450310|Liberibacter_crescens_BT-1_EZ-Tn5_Transposon_Disruption

ATCCTAAATTTGGTATGTGTTGTACGTGCGGATCCCAGATAAAAAAAGAATCATGTTGTGTTTTATCATTTCTAAGAAAAAATACGGGTATACTTGATCC

>Intergenic_Region|684926..684844|Liberibacter_crescens_BT-1_EZ-Tn5_Transposon_Disruption

ACTTTACTCCGTCCTTGCCCCCTTATTGCAATTCTGATCGTATATCTTTAACCTTCATAAGAAATAATAAGATTGATTTAAAGTAGGTGTTGATTAAATA

>B488_05870|646181..646279|Liberibacter_crescens_BT-1_EZ-Tn5_Transposon_Disruption

GAATAGCTGTTGGCAATTCGACTTGCTTTGCAAGGCGAATATAGGCTGTAACCGCTTTTTCGCTATACAAAAAACCTGCATTTGCTTCATAAATTCCGAT

>B488_03060|352692..352617|Liberibacter_crescens_BT-1_EZ-Tn5_Transposon_Disruption

TATTACAAATGATAGTTATGCCCTTGGTCATGATTTCTATTCTTAATGCCGTTGCCAAGCTGCACGATGATCCTTCAACTCAGAAAAAGTTCCAGTTTTT

>B488_08830|961380..961281|Liberibacter_crescens_BT-1_EZ-Tn5_Transposon_Disruption

GAGCAGTATATTTTACCTTACAAGGATCATTAAAAATTGCACAAGCAATAGCTTTGCAAAAATCAACATCAAACCCTTGCCAATTTCCGTTTGAGTCAGG

>Intergenic_Region|1493450..1493549|Liberibacter_crescens_BT-1_EZ-Tn5_Transposon_Disruption

CTCTAATGTCTTTAAAGCATAAACTTTTATTTTAAGCTTCATTTCTTATACTTTTAAGAAAATTTCTGTTTCAAACATTTATTCTTTCAATTCATTTTAT

>B488_12840|1400345..1400246|Liberibacter_crescens_BT-1_EZ-Tn5_Transposon_Disruption

ATATTGTATTACATGGAACTGTTCGCACTATTCAGGATTCTCAAAAAGCAGTTGATTTGGCTAATGCTTTTATTAAAGGAGGAGAAGCAACTACTCAAAC

>Intergenic_Region|1330883..1330982|Liberibacter_crescens_BT-1_EZ-Tn5_Transposon_Disruption

ATATTACCTTGCATAGATAGAATAAAAACCTCTTATAAAAAAATCCAGATGACTCTGGATGAGTAATGAATACTCTGCTGTACCTTAATAGAAAAACTGG

>B488_05170|558041..557942|Liberibacter_crescens_BT-1_EZ-Tn5_Transposon_Disruption

TCCTTATGCCGTTAAACAGGTTACCAATACTACTACCTATGCCCGCCCATGATGAGGTATTACCCTGATTGATGTTGCCGATCTGATCCATGAGCTGTTT

>B488_01030|122759..122858|Liberibacter_crescens_BT-1_EZ-Tn5_Transposon_Disruption

GGCATGAGTACTTTACATCCGATCTCAAGAAGTTTATCTGCAACTATTAGATCTTCCGTCCTATAAGGGAAGACCTCCCATCCGTCCTTGTGTAATATGC

>B488_12990|1411190..1411091|Liberibacter_crescens_BT-1_EZ-Tn5_Transposon_Disruption

GCTTATTTCTATCTATAAAAGATTGAGTAGTTTTGAAAAAACATTAGAAAACAAAACAAGTCATTAAACAAGTTTTTGAGAGAAAAAAGCTTTTTATGAT

>B488_03250|375064..374965|Liberibacter_crescens_BT-1_EZ-Tn5_Transposon_Disruption

CCTTAACCCAGCCCGCTTTATCAAGAAGATTATTAGCTCCTTCAATATCCTGTTTCAAACACCAATCATTCGCTGTAGAAACATAGGCTTCTGGAGCCGG

>B488_09390|1013292..1013391|Liberibacter_crescens_BT-1_EZ-Tn5_Transposon_Disruption

CTTCAAAAAAGCGCTAACAAGCGATTGAAACATATTTTTTATACCTCTTCATGAGGGAAGGTATGTCCTGTAAAATAGATTACAGGAATAAAGATTAGAA

>B488_06280|691624..691560|Liberibacter_crescens_BT-1_EZ-Tn5_Transposon_Disruption

AGTGACTTCTGAACCTCTCGAACACAAACAGATAAAAGTCCAGGGGTTAATAAATGATCCTTCAACTCAGCAAAAGTTCCATTCGAAATGAGAAAGCTTA

>B488_06300|693204..693105|Liberibacter_crescens_BT-1_EZ-Tn5_Transposon_Disruption

GCCCAGAAAGAAATGTTTGCAGCAAAACTCCTTTCTGTTATATCAAGAGATTCTATTGTTATACGCAAGGACCATATGTCATGAATGCATATTGCCAAGC

>B488_03950|458481..458382|Liberibacter_crescens_BT-1_EZ-Tn5_Transposon_Disruption

GGATTACCAAGATCTCCATTTCTTCAAATGCACAATAGTCCTTTTCTATGAAAAATACTTTCATATTTCCTTAAACAACAAATTATATGATATATATATC

>B488_07640|847343..847245|Liberibacter_crescens_BT-1_EZ-Tn5_Transposon_Disruption

CAAACAGATGCCGATGAAATAACTGTTGTGGCTCATTCCATGGGAAGCTATCTCTTGATGGAAGCTTTAAGGACACTGTCATTCGCAGGGAAAGACCGGG

>B488_05530|600068..599969|Liberibacter_crescens_BT-1_EZ-Tn5_Transposon_Disruption

GTGTAGTATAGGAGACAATATCTCCAGAGGCTATAGCCCAATATTGCTCCAGGAAAGAGGCTACAAATAACATTGAACATGGTATTAAAGAACGGCTGAA

>B488_08300|907483..907383|Liberibacter_crescens_BT-1_EZ-Tn5_Transposon_Disruption

TGATAAGGTTGTGTGTTGGTCCTTTGGATTCCTCATGACTGGAGAACCAGTATGTAAAGATAACCCCAGGCTTAGTATAAAACTGTGAATATGCCTACAT

>B488_05730|625596..625497|Liberibacter_crescens_BT-1_EZ-Tn5_Transposon_Disruption

AAATAGCGACTGTGAATAACAAGTTTACTCAAGGAATAAATTCTTCAAAAACAAGTTTTAGTCAGGATATAACAAATATAAGCAGCAAGTTGAGCCAGGA

>Intergenic_Region|463950..464049|Liberibacter_crescens_BT-1_EZ-Tn5_Transposon_Disruption

CTATAGAAGTCTTTTTAAATTTTTTGTATTTTGATATAATTTCTACTTTATTTAATAGGCCAATAAACTAAATAATTCGTTTTTTAGTAAGTATAATTTT

>B488_13120|1423430..1423518|Liberibacter_crescens_BT-1_EZ-Tn5_Transposon_Disruption

GGTATCAGGGGCGTATTTCATCGAGCTTTCCGGCGAAGCGCCTAACAGGGTGAACTCATTATCCTGCATAAAAAACATATACGGGCTGGAGACGAAAACG

>B488_10820|1180368..1180269|Liberibacter_crescens_BT-1_EZ-Tn5_Transposon_Disruption

GTATTGCTATCATTGCTCCTGGTGTAGAATTCTCTATAGCTTTTTCCAAACGATCACAAATTTTATGTGCGTTTGCAACTGTCATATCAGAGCGTACAAC

>B488_08900|968121..968220|Liberibacter_crescens_BT-1_EZ-Tn5_Transposon_Disruption

CTGCTGTGATGTTTTAAAATTGTTTTTGCTGTTTCCAGACATAATTTCTTTCCCTCTCCCAATAAGTGTGGTATAAAAATTATGAACCTTACGCAAATAA

>Intergenic_Region|328974..329070|Liberibacter_crescens_BT-1_EZ-Tn5_Transposon_Disruption

CTTGTGGCTAGGGGTGAAAGGCCAATCAAACTCGGAGATAGCTGGTTCTCCGCGAAATCTATTTAGGTAGAGCGTTAACTGAATACCCTCGGGGGTAAAG

>B488_10610|1157348..1157249|Liberibacter_crescens_BT-1_EZ-Tn5_Transposon_Disruption

GCTTTATTTTTAGGAATTGAAAGTTTTATAATTTTAATTTTAGTCCTTTATACAGCAATACAATCTTCTGAAAATTTCATAGCACTTGTTAATTCTAGGA

>B488_05410|584425..584524|Liberibacter_crescens_BT-1_EZ-Tn5_Transposon_Disruption

CCCTTACCCCCTTCAACAATAAATTTCTGATTTTCAAGCATAAATCTTTTATCGTCGTGCATCTCCGCTGGAGAAGTAATAATGATTTTGTCATTACCTT

>B488_02000|237210..237144|Liberibacter_crescens_BT-1_EZ-Tn5_Transposon_Disruption

CCCCTGCAGAACACCAGAGCACATGGCTTCTGCCTCTCCATGGTTACTGCTCAAATCTTTGAACATATCCTGCAAGAGAGATAAAAAACCCTTGTTACAA

>B488_12800|1395058..1394959|Liberibacter_crescens_BT-1_EZ-Tn5_Transposon_Disruption

CTCGTATGGTACGTTATATGCCTATTCAGGAGGTGTCTTTTTTTGCAACAGTTATTAACATTCAAGCTCAATCAGGGGGTAATCTTTCTGAAGCGTTATC

>B488_01810|221870..221969|Liberibacter_crescens_BT-1_EZ-Tn5_Transposon_Disruption

GCTTGATACCGCTTTTATCATTCTGTTTTTTGCTCTATTAAACGCAATAGATCGTTGGCGACTTTTAACTAAAGGGTTCCCTTGATGTAGTTTTGTTTCT

>B488_00930|110042..109958|Liberibacter_crescens_BT-1_EZ-Tn5_Transposon_Disruption

GCATTAGTAGCTTCTGGATTATCGAACAAATCTTTCTTTTTTGCTTGCTTTTTACCTGCTAAACGCAAGGCAAACCGGAATCTATCAACGTAGCAGTGAA

>B488_10250|1122545..1122644|Liberibacter_crescens_BT-1_EZ-Tn5_Transposon_Disruption

GATTAAGGTTAGCCATAAAGTGATCAGACGGAATGATTATCTCCACTTCTCGCTTTAAACCTTCTGCAACGCTCTCAATAACTTGCATTTTAACCCCTTC

>B488_04940|543177..543080|Liberibacter_crescens_BT-1_EZ-Tn5_Transposon_Disruption

GTTTAACACGCATTAAAATGGTTTTTAAATCTTCAAAATACTTTTGCGATGCACAACATATTCAGCAAGGGATCCCGTTTCAGATTCTGAAACAGATCTG

>B488_02200|255214..255115|Liberibacter_crescens_BT-1_EZ-Tn5_Transposon_Disruption

AGCAGGTACTATGGATGTAGTACTTGCTTCAGGCTGGCCTGGAGTGATGCTTCAACTCACCAAAAGTTCTGGACTGGAAGGTGATTTTAATCGCAAGGAG

>B488_06240|688811..688712|Liberibacter_crescens_BT-1_EZ-Tn5_Transposon_Disruption

GAGGTAAAGACATTTCGAATATTAGGTTTTGGTACCCTATTTTAAAAAATATAGCTGAGCTAATAAAAAAGAACAACATAACGTATATTGGTTTTTTTGT

>B488_05840|641768..641867|Liberibacter_crescens_BT-1_EZ-Tn5_Transposon_Disruption

GGAGAGACTATCCAATATGTTGGAAAATTCATTGAGGCAAACGGTGGAACGCTTTTTATTGAAGAACCCAGTGAATTGCCCCTTGAAACGCAAAAAAAAT

>B488_05410|584433..584334|Liberibacter_crescens_BT-1_EZ-Tn5_Transposon_Disruption

GGGTAAGGGAGATGATATTATTACAGTCAATGATCGTTTCTTGGATCCATTGAAAATATCTTTTTGTTGTAATGAAGCCCCTCCGAGAAATGTAACATTT

>B488_05720|624654..624753|Liberibacter_crescens_BT-1_EZ-Tn5_Transposon_Disruption

GTCCTGTATACTTTAACATGTTTTCATATTTGAAAAAATTTACTCCTGTATTAACACCGAAATATGGTCCTGACCATAACTGTTTATCACTGAAATTGCT

>B488_09390|1013300..1013201|Liberibacter_crescens_BT-1_EZ-Tn5_Transposon_Disruption

TTTTTGAAGATCAATAAAAATAATCAAGATATTGTTTTTGCTCTAGGTATTATTTTTGTTATATGTATTTTGTTTTTACCTATACCTTCATTCCTTATTG

>B488_08250|900353..900253|Liberibacter_crescens_BT-1_EZ-Tn5_Transposon_Disruption

GTGTATGATGGTTCTTTTTATTCAATGGTTTTGAATTTTTTGATTTCTCCCTTATTCGGGCCGCTTCTTCAAAATTAAGATTGCTCGATGCTTCTTGCAT

>B488_05470|592034..592133|Liberibacter_crescens_BT-1_EZ-Tn5_Transposon_Disruption

AAGTTGTCCATAGAAAGTTGTAGAATACGATGATGCTTGTTTGTAATAAAGGATTGCCTGCTTTTTATCTTTTAATTCAGAAGCTCTGCCCAGCCAATAG

>B488_01510|185634..185733|Liberibacter_crescens_BT-1_EZ-Tn5_Transposon_Disruption

TAATAAGGCTCCAGCAGATCATGGGATACGGTCTCTTGATGATACTCAAGAACAATCAGCTGATTCTTTATCTGCGCATTCAGAAGATTCGTTACAGAAA

>B488_05720|624662..624624|Liberibacter_crescens_BT-1_EZ-Tn5_Transposon_Disruption

ATACAGGACAGGGTATGAATAACAACTATTCTAATACAAGGGGGTGTATGAGCCATAAAGCCTTATGTGTGATAAACGTTATATGGGCGGGGCTGGGTTT

>B488_05720|624670..624752|Liberibacter_crescens_BT-1_EZ-Tn5_Transposon_Disruption

TTATGACAGTGGTTGGTCATTTTATCATATTTGAAAAATTTTACTCCTTTATTAACACCGAAATATGGTCCTGACCATACCTGTTTATCACTGAAATTGC

>B488_13470|1469154..1469219|Liberibacter_crescens_BT-1_EZ-Tn5_Transposon_Disruption

CTCCAGTAGGCCCTATTAACATTATATTCTTTGGCGTCACTTCATCACGCAAATTCTCAGGTAATTGATGGATGGTCCAAATGAATGAAGGAGATTAATA

>B488_01800|221387..221288|Liberibacter_crescens_BT-1_EZ-Tn5_Transposon_Disruption

CTTATATATCCATCACCTGCTAACTGTAAGCAAGCAATCTCACGATCTGTTAAATTTCTATTTGACATAGTACGTCGTTTAGAAAATTCCAGAAAATCTA

>B488_05170|557977..558028|Liberibacter_crescens_BT-1_EZ-Tn5_Transposon_Disruption

GGGTAATACCTCATCATGGGCGGGCATAGGTAGTAGTATTGTATACCTGTTTTTCTCTAGTAATTCAACCAGTCCCTAAATCGTGAGGGGTTAATGCCTA

>B488_04940|543589..543493|Liberibacter_crescens_BT-1_EZ-Tn5_Transposon_Disruption

ACTATCGGTCTTCCATTAATATCAATCGCTTTTCCATCTATAATATTTTTGATATATTTATTTATCTGTTTGATCTCTGACATTTCATATTTAGTACATC

>B488_10960|1195378..1195477|Liberibacter_crescens_BT-1_EZ-Tn5_Transposon_Disruption

ATGTAATGTTCGACTATTAATAACTCCGAAAATTTTACGTAAAAGCGGCTCATTAGAAATCATCATTATCAAGTCGCCAAAGAAAAATGAAAAACCAACT

>B488_00200|27823..27765|Liberibacter_crescens_BT-1_EZ-Tn5_Transposon_Disruption

CTCCAAGGCGTAATTTTATAATCCCGAGAGTTTTGACTCCAATTAACACCTGATCCTTCACCTCAGTAAGGCTCAGTCCCTTCCAGTTTCTCTCTGTCTT

>B488_03070|354056..353957|Liberibacter_crescens_BT-1_EZ-Tn5_Transposon_Disruption

AGCTTTTACTGCACAGCGTGTAGAATCTCTTCGTGAAACAATACAGAACATATCAAATCGTTTGATTAACAATCTAGTGAACCAGTTAAAGAAACACAAA

>Intergenic_Region|975653..975747|Liberibacter_crescens_BT-1_EZ-Tn5_Transposon_Disruption

TAGCTCTGTTGCCCTCCGTCTGGATGGGCACTCATGGCCAAAGGCTTACATTGGCTGTAATTCACCCTTCTCAACGCCTCTATAGTAAATTACACGGAAA

>B488_05720|624654..624753|Liberibacter_crescens_BT-1_EZ-Tn5_Transposon_Disruption

GTCCTGTATAGTTTAACATGTTTTCATATTTGAAAAAATTTACTCCTGTATTAACACCGAAATATGGTCCTGACCATAGCTGTTTATCACTGAAATTGCT

>B488_09490|1020140..1020239|Liberibacter_crescens_BT-1_EZ-Tn5_Transposon_Disruption

CATATCAACTATACCAGATAGATTATCAACATCCTGATTTTCATCCTTTCCTATAACACAGTATGCAGATGTATTCTTTTTATTCCATATCTTATTATTT

>B488_02000|237199..237100|Liberibacter_crescens_BT-1_EZ-Tn5_Transposon_Disruption

CTCTAAGGTTACTGCTCATATCTTTGAACATATCCTGCAAGAGAGATAAAAAACCCAAATTAGAATCCTGTTGAATAGTGTTTTTATCTAAAAAACTATC

>B488_03860|450694..450595|Liberibacter_crescens_BT-1_EZ-Tn5_Transposon_Disruption

GTGTGGGTTTATTTGACGAGAAAAATTTTAATAAAGGTTATGGAGAAGAAAATGATCTTTGTTGTAAAATAGAAAAAGCAGGATGGCATAATATTTTCCT

>Intergenic_Region|329143..329044|Liberibacter_crescens_BT-1_EZ-Tn5_Transposon_Disruption

CTAGTACTCCTCGGTATTCGGAGTTTGGTTAGGAGCAGTAAGGCGGTGAGCCCCCATAGCCTATCCAGTGCTCTACCCCCGAGGGTATTCAGTTAACGCT

>B488_11960|1305770..1305869|Liberibacter_crescens_BT-1_EZ-Tn5_Transposon_Disruption

ATCCAACCTCGACCACCATGACGCAACCAAGGGGGGACTTTTTCTTCAAGTGAAGCAGTGATCAATACATGAGTTTGACCAAATTTGATTAAACAAGAAC

>B488_11360|1234435..1234336|Liberibacter_crescens_BT-1_EZ-Tn5_Transposon_Disruption

ATATGATACAGGGCGTTCTGATTTTTCACTGTTCTTAGAACAGCCTGTTACTATTATTGTTATAGCTATTATAAAAATAATACGATTGTATATCAATATA

>B488_00260|38706..38607|Liberibacter_crescens_BT-1_EZ-Tn5_Transposon_Disruption

GCTCTATACCCGGAAAATATGTCCGTAAATTCATCCCTAAACATAATACGATACAGTTTATTAAAAAAAAGATTTCCTAAAAGGTGACCTTTTCGATCAA

>Intergenic_Region|562561..562462|Liberibacter_crescens_BT-1_EZ-Tn5_Transposon_Disruption

CATCAGGATATCCTTCTGAAAAGGGTCGTGCACGGTGTGACGGAAGAAGGATCAGTATAGGTATATCCGCAGCATTGCCATACTCCATCAAGCGATGATC

>B488_06300|693204..693105|Liberibacter_crescens_BT-1_EZ-Tn5_Transposon_Disruption

GCCCAGAAAGAAATGTTTGCAGCAAAACTCCTTTCTGTTATATCAAGAGATTCTATTGTTATACGCAAGGACCATATGTCATGAATGCATATTGCCAAGC

>B488_05410|584433..584334|Liberibacter_crescens_BT-1_EZ-Tn5_Transposon_Disruption

GGGTAAGGGAGATGATATTATTACAGTCAATGATCGTTTCTTGGATCCATTGAAAATATCTTTTTGTTGTAATGAAGCCCCTCCGAGAAATGTAACATTT

>B488_09330|1008430..1008331|Liberibacter_crescens_BT-1_EZ-Tn5_Transposon_Disruption

GCTTTAGATATAATGGGTATACTTTTAATAACTCCCTCTTCTTTTACTTCAAAAACGAGTTCATCATGTACCTGGAGCAACATTTGTGCTGAAATATTAT

>B488_02990|346570..346471|Liberibacter_crescens_BT-1_EZ-Tn5_Transposon_Disruption

CGTTTAAGGTCATATTTTGTGTAACACGTGTAAGATTCGTTTCAATTGACATAATTTCAGGAGAAAGCTTTTTTATTAAAGGCAAACATAACATTGATAA

>B488_02040|240178..240079|Liberibacter_crescens_BT-1_EZ-Tn5_Transposon_Disruption

TGCTTGACGTGCATTAAGTTTGATTAAAATAGAGGAAGCTGTTTCAGGATTAATTTTTTCTAATTGGGCAGCAGCAGCAGCTGAAGTCATCTTTTTATAG

>B488_11070|1206830..1206731|Liberibacter_crescens_BT-1_EZ-Tn5_Transposon_Disruption

TATTCTAATAGAGCAAAAATGCGATTTCTTGGAGTTAGCCAAAAAACTTTGGAAACTTGGGGAGCAGTCTCTTTTCAGACAGCTTTGGAAATGGCACAAG

>Intergenic_Region|541668..541568|Liberibacter_crescens_BT-1_EZ-Tn5_Transposon_Disruption

GAATAACAGGTGTGTTAATCTTCTCATCAGAACGGCTGTCAACGGTGATCCTTCAACTCAGCAAAAGTTCAATTAGCGGGACGACAAATCATTTTGTCGG

>B488_01340|165656..165755|Liberibacter_crescens_BT-1_EZ-Tn5_Transposon_Disruption

GTTTAACCCCTATCATTATCATTTCTACGTATATTGCTATGCATTTTCATCAACCTCTTGTAGATAATACGCTTAGCCTTGTAGATGAAAGGCTTGGTTT

>B488_06280|691624..691560|Liberibacter_crescens_BT-1_EZ-Tn5_Transposon_Disruption

AGTGACTTCTGAACCTCTCGAACACAAACAGATAAAAGTCCAGGGGGTAATAGATGATCCTTCAACTCAGCAATAGTTCACTCATGTGCTGGTGAATGTT

>B488_03250|375064..374965|Liberibacter_crescens_BT-1_EZ-Tn5_Transposon_Disruption

CCTTAACCCAGCCCGCTTTATCAAGAAGATTATTAGCTCCTTCAATATCCTGTTTCAAACACCAATCATTCGCTGTAGAAACATAGGCTTCTGGAGCCGG

>B488_01820|222857..222956|Liberibacter_crescens_BT-1_EZ-Tn5_Transposon_Disruption

GACGTATACTTGTAGAGTTAAGTATATATTCTCGGAGCTCCGTTGAAGTGCTGTAAAGAGCTGTTATTAGAGTTTCAATTGGAATGTTATTGAGTAAAGT

>Intergenic_Region|406017..406116|Liberibacter_crescens_BT-1_EZ-Tn5_Transposon_Disruption

GATTTATAGATAAAACAACTTTTTATTAATCAATATAAAATTCGTAATCAAGTATTAAAAATTCTAATTTTCTAGAAGTATTAGATCTTTTATCTTTCTA

>B488_07760|854482..854383|Liberibacter_crescens_BT-1_EZ-Tn5_Transposon_Disruption

CAACAGGCTCTGATAATGGACTGCCCTTTACAGCCGTGTATCTTTCAGGATTTCAGAATATTTCGACACCTGGACAAATCAATCGTAGCAGTTTGGCGCA

>B488_13340|1447546..1447643|Liberibacter_crescens_BT-1_EZ-Tn5_Transposon_Disruption

GTAAAGCATAGTCTAAAAGTTAATGTGTATGATATGAATGCTATTGGCGAAATACTTGACCTTGGCATATCTCTTGGATTTAATTCTTCCTATGACATTT

>B488_13510|1471925..1471826|Liberibacter_crescens_BT-1_EZ-Tn5_Transposon_Disruption

TCCTTCTGTTGATACTTTTCCATTTTTTGCATCGATGCTGATGACAACTCTTCCGGGAAAAAGTTTACAAGCAGAATAAACCATCTCAGGGTTATTTACT

>B488_12150|1328089..1328188|Liberibacter_crescens_BT-1_EZ-Tn5_Transposon_Disruption

ATATAACACTGATGCCTCATGCGATAATTTTTTTGCTAATTCTCGACCATCACCATCAAAATAGGAAGAAGTTTCTTCCACAAGATCCATACCTTCTGAA

>B488_01760|213988..214087|Liberibacter_crescens_BT-1_EZ-Tn5_Transposon_Disruption

GTGCATTGAAGGTAAAAGGCGTTTTATTATCTATTTCTTGGTTTAGGTGATCAATTGTATTTATTTTCATTGATTCAAATTCAGTAGATTGTTGTGATTG

>B488_11250|1225309..1225210|Liberibacter_crescens_BT-1_EZ-Tn5_Transposon_Disruption

GGTTTAAAGTTAGTCAATTCCAGTACAAATGTAGTTATGACTCGTACTTTCTCTAAGGCCTATGGTTTGGCTGCATTGCGCATTGGTTGGCTGTATACGA

>B488_02910|337496..337397|Liberibacter_crescens_BT-1_EZ-Tn5_Transposon_Disruption

GCTCCATGAACACTTACCAGCAATCGATCTGTTTCAAAGGGCTTTTCTATATAATCAAACGCTCCTCGTTTTATCGCTGAAACTGCTGTCTCAATATTCC

>B488_06040|667155..667056|Liberibacter_crescens_BT-1_EZ-Tn5_Transposon_Disruption

ATCTAGCATTGGTGCAACTGCAAAAACCTTATATAGATACGATGAAGTATTTGCTTGATCAGAGTGTTTAACTTCTTGATATATAAACATACTTAAGTAT

>B488_11960|1305770..1305869|Liberibacter_crescens_BT-1_EZ-Tn5_Transposon_Disruption

ATCCAACCTCGACCACCATGACGCAACCAAGGGGGGACTTTTTCTTCAAGTGAAGCAGTGATCAATACATGAGTTTGACCAAATTTGATTAAACAAGAAC

>B488_06040|667781..667880|Liberibacter_crescens_BT-1_EZ-Tn5_Transposon_Disruption

GAGTAATGTTAGGCCGGGCATCATATCAGAATAGTACAATACTAACTTCTGTTGATCAATATTTTATAAATCCTTTAACAGGTTCTCATCCTGTTATTGG

>B488_11150|1215065..1215164|Liberibacter_crescens_BT-1_EZ-Tn5_Transposon_Disruption

AACCTATTCTTAAAAACATGAGCTTCGAAATTCCTTCTGGACATACAGTCGCGATTGTTGGGAAATCAGGAGCTGGAAAATCAACTATTTCTAGGCTTCT

>B488_13710|1498257..1498356|Liberibacter_crescens_BT-1_EZ-Tn5_Transposon_Disruption

CCTCATGATTGATCCAATTGATTTTTCTCAAATATATACACAAAAAATTTTAGAAGAAAAATCTCTTAATACGAAAAACAATACATCCCATTAAGATTAT

>B488_03930|455597..455696|Liberibacter_crescens_BT-1_EZ-Tn5_Transposon_Disruption

CTCTTCGGCTGCGGTGATTTGAATATGATTAATATGATGATCCTTCCATTCAGGTTCATTTCGAGCATTAGCAAATCGTAACGCCATTAGATTTTGTACA

>B488_11120|1210807..1210906|Liberibacter_crescens_BT-1_EZ-Tn5_Transposon_Disruption

GCACAAGGTATTGTTGACTCAACCTTATTTAATGCTTTTGAAAAAACTCCACGCTCTATTTTTGCACCTCCACATTTTATTGATGTGGTATGGTCTAATC

>B488_13270|1441605..1441704|Liberibacter_crescens_BT-1_EZ-Tn5_Transposon_Disruption

TTATTGTACTACATTAGCCGAGCTTATTCAAACTTATGAAGCAATTAATGAAGCAGCTGATATCGTAGAGACTATTAAATGATGAAACCTTCTAGTCGTA

>B488_11120|1210807..1210906|Liberibacter_crescens_BT-1_EZ-Tn5_Transposon_Disruption

GCACAAGGTATTGTTGACTCAACCTTATTTAATGCTTTTGAAAAAACTCCACGCTCTATTTTTGCACCTCCACATTTTATTGATGTGGTATGGTCTAATC

>B488_12300|1347512..1347609|Liberibacter_crescens_BT-1_EZ-Tn5_Transposon_Disruption

GTCATGAATGGTGTTTCTTGACAAAACCTAAACACAAAAACCTCAGGATCAGTTAAAATACCGAGTCCTAGTTTGTTCATTACTTCTTCAGTTTCAATGA

>B488_12250|1339148..1339247|Liberibacter_crescens_BT-1_EZ-Tn5_Transposon_Disruption

GTCCAGACTACCCCGATTAACAAACCGATTAAAATACCGCTAAAAAAACCGTCGTTAGCATTTCTGAATAATCGATGTCCTACATTAGAGAAACGTTGTG

>B488_13280|1442284..1442383|Liberibacter_crescens_BT-1_EZ-Tn5_Transposon_Disruption

CCCGACCATTTAGCAAGGATATGCAACCTTACGAATCACTTATGACTGTACATAATGGACAAATGATATTTTCCAAAGCTGTAGAAATGATGGTTGAATC

>B488_00720|86576..86675|Liberibacter_crescens_BT-1_EZ-Tn5_Transposon_Disruption

CAATAAACCTATATATGAAGAGCAAGAGCTGCTTTGATAAAGTCATCTAGATTTCCATCTAGTACATCTAAAGGTGCTGTATTTTCTACGCCTGTTCTCA

>B488_05580|605036..604937|Liberibacter_crescens_BT-1_EZ-Tn5_Transposon_Disruption

GATTTGGAGCAATGACTCCTGAGCCTGCTCGTGGTTATAATCGTCCATATTACTACGATCCTAAATACCATCCATACCATCCTAAACGTATTATTTCTTC

>B488_11590|1258344..1258443|Liberibacter_crescens_BT-1_EZ-Tn5_Transposon_Disruption

CTGCAGCCCTCAACAATCCAGAAAGAAAAATCATTCAACTTTCAGCAACTCAAAATGCTATGTTACGTTTAAAATGGACCCCAAGTGATGACAATCATTT

>B488_05180|561459..561360|Liberibacter_crescens_BT-1_EZ-Tn5_Transposon_Disruption

AGCAAACCCTGTCATATTGGTCGGCGCAAGAGCACCTATTCCTCTGATAAAATTTTGTCCAGTTTTTCCATAAGCAACCGTATCGGCTGCAGCTTTTTCA

>B488_00090|12043..12142|Liberibacter_crescens_BT-1_EZ-Tn5_Transposon_Disruption

CCTCTGTAAAGAGATGAAACAGAAGGGGATACGCATATTTACATATGCATATGGTGATCCTTCAACTCAGCAAAAGTTCCTTGCAGTATCCTGCGCTACT

>B488_03820|443897..443798|Liberibacter_crescens_BT-1_EZ-Tn5_Transposon_Disruption

TTTATATACTTGCACCTATAATACGTAACCGAAAAGGAGAATATAAAAAAGAACTCTCAGAATTATCTAAAAAGGGATTCCAACGTGTTAAAATTGACGA

>B488_13290|1442985..1442886|Liberibacter_crescens_BT-1_EZ-Tn5_Transposon_Disruption

GTGTAAGAGGTAGTGTTGGTATAGGTGAAATTCCCAATGGTTTTGGGATCGAAGTAGAGCTGCAAATTAGTTTACCTGGACTTGATCGCAATATCGCTCA

>B488_03730|427876..427777|Liberibacter_crescens_BT-1_EZ-Tn5_Transposon_Disruption

ATATAATACTGAGCAGCATAATCAGAAATAGCTTGTTTATCAGAAAGACCTTTTTTCACAACCTCTGCCATCAAATCCAGATGAGATGCAAGAAACGCAA

>B488_13240|1438260..1438359|Liberibacter_crescens_BT-1_EZ-Tn5_Transposon_Disruption

TCTTTGGACTGGGAGAAACTGTTGATGACCGCGTTGATATGTTAGTAACCCTTTCTAACTTAAAAAGTCCACCTGAAAGCGTACCTATTAACATGCTTAT

>Intergenic_Region|236208..236109|Liberibacter_crescens_BT-1_EZ-Tn5_Transposon_Disruption

AACTAGAGCTTTAATCTTGTGCAGAACTTACCCAAAAATCAGCGAAAAGGCTTCTCATTAAAAAGAGCTTTTCTTTATCAGTAACGTAACTATTTCTTGA

>B488_08300|907203..907302|Liberibacter_crescens_BT-1_EZ-Tn5_Transposon_Disruption

TTTATATACTATTTGTGATCGTATTGCTGTTATTAATAAAACCATTATTGCAGAAGGAGGCATTCAGGATATATTAAATGTTGATGAACCTTCGATACAA

>B488_11950|1304613..1304712|Liberibacter_crescens_BT-1_EZ-Tn5_Transposon_Disruption

AGCTCTATGTGAAAGCGGTTCCTCACGTTGACCTACTATAAAGGCATTTTTTTCTTCCAATGTCATTTCACCAAAAGTACGACTATGCCCTTTTGGTTGA

>Intergenic_Region|1026016..1025922|Liberibacter_crescens_BT-1_EZ-Tn5_Transposon_Disruption

ATAAGATACTCGGCATGTCCCCCCTTATTTCTTAACTATAATTTTAATTTTATATTCAAGGGACATATAATTATGAGTAGTATTTTAACCAATGATCCTT

>B488_13420|1459144..1459243|Liberibacter_crescens_BT-1_EZ-Tn5_Transposon_Disruption

GGCAATGACCCTGAAAATACAGCTTTTATTAACGCTGCATTTGTACTTTATTCGCTCGGATGTGGAAAGGTGTCAGTGCCTTATCTTGACGAGTTCTATC

>B488_13130|1427462..1427558|Liberibacter_crescens_BT-1_EZ-Tn5_Transposon_Disruption

CCTTTCTATAGCATGGGCAGCCATAGAACATTTACATGAAATTAACTTTTGTCGAGGCCTATTAGCCACTCATTTCCATGAACTGTCAACTCTATCAAAA

>B488_03600|413936..413837|Liberibacter_crescens_BT-1_EZ-Tn5_Transposon_Disruption

GCATTATTGATCATCTCTTCTGCATTGGAATCCCGTGGACCATCAGCGCTGCGAATACGCCTAAAAAGATCTTCTATTAAGCGACGCTCTTCATTATTTA

>B488_07820|859862..859961|Liberibacter_crescens_BT-1_EZ-Tn5_Transposon_Disruption

GTGTGTGATGGTGGAATATCCATATGCGGAACGAAATCGCTCATTTTTGGATAGGCCTTCGCATAGTCTGCCATGCTTTGTGGCTGGAATGGATATGGCC

>B488_07620|845912..845813|Liberibacter_crescens_BT-1_EZ-Tn5_Transposon_Disruption

TTTCAACACTTGGCTTTTCAAGGCCATCAATATCATTGAGAGATTGATGATCAAGAAGAGCAAGGACATGTGACATAGAACTTTCAATATTAAAAAAATC

>B488_07830|862265..862364|Liberibacter_crescens_BT-1_EZ-Tn5_Transposon_Disruption

CCTCTGCATCGCTCACCAGTCTTCTGATTGTTCCGGCAAATTCCTGTTCTTTTTCTTTTTTTCCTGTCATTGTCATGATCTAAATCACTTTCCTTGGATA

>B488_11490|1246874..1246774|Liberibacter_crescens_BT-1_EZ-Tn5_Transposon_Disruption

ATGTTGGTCTAGCAATTAAAGATCAAATTTGCCAGCTGTAGGGTATGCTTTGTTAACAATGTTGTTAGTAATTATTTTTTATGATCAGTTGTTATTTCGA

>B488_06540|724108..724009|Liberibacter_crescens_BT-1_EZ-Tn5_Transposon_Disruption

CTTTAATGTTGAAGGAGCAGTATTAAATTCCTGCGTTAAGACATCTACAAAGCTTTGCAATAGTCCAGGTGAGTAGCATTTTTTAAATACAAGAGGTGCA

>B488_03510|402523..402622|Liberibacter_crescens_BT-1_EZ-Tn5_Transposon_Disruption

GAAAGCAGCTTGTGCTAGTTTTAAAGCCATTGACCAAAAATATCCTAAAGCTTCTAATAGTATTAAGAGTAAGAGCTTATCTGAACAAAAACGTTTATCT

>B488_10370|1132798..1132699|Liberibacter_crescens_BT-1_EZ-Tn5_Transposon_Disruption

ACTTATGCCGCTATCGCCTGGTATCATAATATGTTGCCAGGAAAACATGAAGATCAAAAGATTTTTTTGAGTAATGTGGAGCAGTTTGCATTAGGTCCTT

>B488_02050|240929..241014|Liberibacter_crescens_BT-1_EZ-Tn5_Transposon_Disruption

GTTCAATGGACATACAACAACCCCCAATAGGTCAACAGGCAATAGATTTGCTGTCACCACTTTAATATTCATTAGGGGAGTAGAAATTACGAAGTAGATA

>B488_07940|870940..870841|Liberibacter_crescens_BT-1_EZ-Tn5_Transposon_Disruption

ACTTTTTCCTGCCCCGTTTGGTCCGGCAATGGCTGAAAGCTCTCCGGCAAGAAAGGAGCCATTGACATGATGGACTGCAGGCTGCCGTAAATAGGCAAGC

>B488_12250|1339148..1339247|Liberibacter_crescens_BT-1_EZ-Tn5_Transposon_Disruption

GTCCAGACTACCCCGATTAACAAACCGATTAAAATACCGCTAAAAAAACCGTCGTTAGCATTTCTGAATAATCGATGTCCTACATTAGAGAAACGTTGTG

>B488_08910|968564..968663|Liberibacter_crescens_BT-1_EZ-Tn5_Transposon_Disruption

ATACAATGCTGCACATAAGTGTCAAGTAGATATTCCGTTCGAACGAGTCAAGTACCAGAGTTGTTTTTTATGAGCTCTTGTACCCCCAATGTTGTGCAAG

>B488_03890|452419..452320|Liberibacter_crescens_BT-1_EZ-Tn5_Transposon_Disruption

TCTGCATACCCAAAAAGCATTAACGCAATACTTGTGAGAAAAATTCCAACCATCAATTAATTCGTCAACTGGATGATAAATACTCTCTATGAACCATAGT

>B488_13710|1498179..1498133|Liberibacter_crescens_BT-1_EZ-Tn5_Transposon_Disruption

AAGTAGAACAGGTAGTATATGTTCTTGTGGAAAGTCTGTAATACGAATTGATCCGCGTTCTATTGATTAGTTTTCTGACCATTCTAAAGTGGTTATTCGT

>B488_01420|175997..175898|Liberibacter_crescens_BT-1_EZ-Tn5_Transposon_Disruption

CACTAGGGCCAAACAAGGCTATATGTTCCCCAGGAAAGACTGAAAGATTAAATTTATTAAATAAAAGCCTGTCGATTTTATTATACCTAAAGCAAATATC

>B488_05840|641768..641867|Liberibacter_crescens_BT-1_EZ-Tn5_Transposon_Disruption

GGAGAGACTATCCAATATGTTGGAAAATTCATTGAGGCAAACGGTGGAACGCTTTTTATTGAAGAACCCAGTGAATTGCCCCTTGAAACGCAAAAAAAAT

>B488_09870|1075906..1076005|Liberibacter_crescens_BT-1_EZ-Tn5_Transposon_Disruption

ATGCTATGTTATTTAATAAATTAAAAGATTGTTTTACATGTAGATTAACTGCACAATTAATTGCCCAACATGCTATGCAGTATTTTTCAAGTATACGTGA

>B488_06100|674398..674497|Liberibacter_crescens_BT-1_EZ-Tn5_Transposon_Disruption

ATTCTGATGCCTGTGCAGACATCAAAATTCCAAACCCCAAGAAAAAAGCTAAAAGTCTAAGAACAACAACACAAATGATAGATAGTAAATAATCTATTAA

>B488_09390|1012562..1012661|Liberibacter_crescens_BT-1_EZ-Tn5_Transposon_Disruption

AGGCAGGAACCTGTGATACAAGACCATCTCCTACTGATAATCTTACAAAGACATCGGCTGCTTGACTAATAGGCATATCATGCCGAAAATAACCAATAAG

>B488_07690|849017..849116|Liberibacter_crescens_BT-1_EZ-Tn5_Transposon_Disruption

TTCCTGATACCCAAAACCCATGCCAACAGAGTGAAAAGAATAAAACTCCCTGCGAGAAGAAAAATTCTTTGCACTCCTGATAACAGCATCATCGCAGATA

>B488_12330|1352089..1352188|Liberibacter_crescens_BT-1_EZ-Tn5_Transposon_Disruption

GTATATGGCACAATAGCATCAAGATAACGTATATGAAGTGATAAGCTTTTCATGAACAATGTATAGAATTGATTATCTGCTCCTTTCATAATAACTGGCG

>B488_11950|1304613..1304712|Liberibacter_crescens_BT-1_EZ-Tn5_Transposon_Disruption

AGCTCTATGTGAAAGCGGTTCCTCACGTTGACCTACTATAAAGGCATTTTTTTCTTCCAATGTCATTTCACCAAAAGTACGACTATGCCCTTTTGGTTGA

>B488_11840|1292688..1292787|Liberibacter_crescens_BT-1_EZ-Tn5_Transposon_Disruption

GCACTAATCCTTGTTATTGTGAGCAGCTTTGATTACTACTTTGAGATGATTAGTACAAAATCTAATTCAATTATTGCGCTTAATAGTTTCTCTGCATTGA

>B488_01420|175997..175898|Liberibacter_crescens_BT-1_EZ-Tn5_Transposon_Disruption

CACTAGGGCCAAACAAGGCTATATGTTCCCCAGGAAAGACTGAAAGATTAAATTTATTAAATAAAAGCCTGTCGATTTTATTATACCTAAAGCAAATATC

>B488_13250|1438630..1438729|Liberibacter_crescens_BT-1_EZ-Tn5_Transposon_Disruption

AAATGACACTCAATCAACTTGTTCTTTATAACAAAAAACTGCAACAGCTCGAGAAGAAAGAACGTCTACGTATATTAACCAAAACAAAAGGGATTGATTT

>Intergenic_Region|386801..386703|Liberibacter_crescens_BT-1_EZ-Tn5_Transposon_Disruption

GCTATACCCTCTCTATCGTACCGTCTTATCTTCTTTAAATAGAATAAAGAAACAAGTCCGTGGTATATTTGCTTACTTACCTCAAGAATCAAGTTATTGA

>Intergenic_Region|329143..329044|Liberibacter_crescens_BT-1_EZ-Tn5_Transposon_Disruption

CTAGTACTCCTCGGTATTCGGAGTTTGGTTAGGAGCAGTAAGGCGGTGAGCCCCCATAGCCTATCCAGTGCTCTACCCCCGAGGGTATTCAGTTAACGCT

>B488_11590|1258344..1258443|Liberibacter_crescens_BT-1_EZ-Tn5_Transposon_Disruption

CTGCAGCCCTCAACAATCCAGAAAGAAAAATCATTCAACTTTCAGCAACTCAAAATGCTATGTTACGTTTAAAATGGACCCCAAGTGATGACAATCATTT

>B488_03930|455681..455780|Liberibacter_crescens_BT-1_EZ-Tn5_Transposon_Disruption

CATTAGATTTTGTACAGTTTCTTTGCCAAGATAATGATCAATACGGAAAATTTGTTCTTCATTGAAAACTTTTCCAATAGTTTTATTGAGAATTTTTGCG

>B488_07730|851914..851815|Liberibacter_crescens_BT-1_EZ-Tn5_Transposon_Disruption

CTTTAAGTGAGTTCATGAGAATACTCTTGTCCACTCAAAATATGCAGGCGCTGCAACAGCTTCTTTTAAATTCTGGTGTGAGCCTGTATCATGGAAATAA

>B488_05840|641669..641570|Liberibacter_crescens_BT-1_EZ-Tn5_Transposon_Disruption

ATCCAGAAGCATGTATGGCTCGAGCAAGCATCTGTTTTCCAACGCCTGATTCCCCTTCAAGGATAACAGGAATAGAATACTCAGAAGCTTTTTTTGCAAG

>B488_06100|674398..674497|Liberibacter_crescens_BT-1_EZ-Tn5_Transposon_Disruption

ATTCTGATGCCTGTGCAGACATCAAAATTCCAAACCCCAAGAAAAAAGCTAAAAGTCTAAGAACAACAACACAAATGATAGATAGTAAATAATCTATTAA

>B488_03730|427876..427777|Liberibacter_crescens_BT-1_EZ-Tn5_Transposon_Disruption

ATATAATACTGAGCAGCATAATCAGAAATAGCTTGTTTATCAGAAAGACCTTTTTTCACAACCTCTGCCATCAAATCCAGATGAGATGCAAGAAACGCAA

>B488_09030|976637..976538|Liberibacter_crescens_BT-1_EZ-Tn5_Transposon_Disruption

CCTTGGAATAGTTTAAAGGAGAATGATTGATAGGCGAGAAGTAGATAAACGCAAGAAAATCACAACTTTAGAATGTAAATATGTAATTACGATTGTCCTG

>B488_06540|723819..723720|Liberibacter_crescens_BT-1_EZ-Tn5_Transposon_Disruption

CCGTTATGGAGCCCCTTTTTATACTTGCATAAAGTGCATACTTTTATTCCTTTCTTGTCAGCAACCAAATCTACAACAAACTCTTCACCATCAATTTTTT

>B488_05460|590246..590149|Liberibacter_crescens_BT-1_EZ-Tn5_Transposon_Disruption

GTCCTGGAGAAGTATCTTTAGCCCATAACGGCGTTTTATTTATGGATGAATTCCCGGAATTTTCTCCACAAGCTCTCAATGCACTTCGTCAACCACTT

>B488_00040|5189..5288|Liberibacter_crescens_BT-1_EZ-Tn5_Transposon_Disruption

GTATGAATATACACAAAATGATCTTGGAGAAATTATTGGAAAAAGTCGTAGTCATATTTCTAATATATTAAGGCTTTTAAAATTACCTGATTCTGTTAAA

>B488_09440|1015023..1015122|Liberibacter_crescens_BT-1_EZ-Tn5_Transposon_Disruption

AATCTAATCGCATGATCTCCTCTTGAAGATTTACAGAAAGTTCATCACGATTCACCCCGACTAATTGAGAAAAATATTTTTGTAGCACTTCTTGCTGATT

>B488_05460|590414..590315|Liberibacter_crescens_BT-1_EZ-Tn5_Transposon_Disruption

CTTCAATACTTCCTCCCTTGTCAACGGCCGAACTCCTTGAAGTTGCAGCAATTCATTCAATGGCAGGACAATTGAGCCATGAACAAAATCTCTCCCAATA

>Intergenic_Region|1235516..1235615|Liberibacter_crescens_BT-1_EZ-Tn5_Transposon_Disruption

CAATAGAAATCGCTATTTTTTTATTATAGAATTTCTCTTACCAAGAGATCTATGATCACTTTTTTATCAAGTAAATAATTACTCAAGATTAGAATTATTC

>B488_00700|83974..83875|Liberibacter_crescens_BT-1_EZ-Tn5_Transposon_Disruption

ACTTACGACGCACAACGACAGCGTCAGGCGACATAGATAAAGCTTTCTCATAGAAAAGGGCTGCAGGCTTATAATCCTGCTCCAGATCAGCTATATAACC

>B488_11650|1270195..1270096|Liberibacter_crescens_BT-1_EZ-Tn5_Transposon_Disruption

GTATAATCTTCAGGTGGAAGAAGAACTTGATATGACATTATTTTCTAAACTGGATCTTGTAACTGAGCCTCCTGCTGTTTGGGTGTCACGGTATGCTCCG

>B488_07940|870940..870841|Liberibacter_crescens_BT-1_EZ-Tn5_Transposon_Disruption

ACTTTTTCCTGCCCCGTTTGGTCCGGCAATGGCTGAAAGCTCTCCGGCAAGAAAGGAGCCATTGACATGATGGACTGCAGGCTGCCGTAAATAGGCAAGC

>B488_04010|464482..464394|Liberibacter_crescens_BT-1_EZ-Tn5_Transposon_Disruption

CTGTATCCTTTCACCAGGATTAGGAAACATTTGATTTGTAGAAATATTAAATTTTTCAAGATAATGAAATAAACAAACTTGATTTCTTGAAAACCGCATG

>B488_04010|464485..464584|Liberibacter_crescens_BT-1_EZ-Tn5_Transposon_Disruption

GTGGATACAGAAATTCATTATCAAGGAAATGCTTATTCTTGGAAGAAAGGTGAAGCTCCACCACTTATGTTTGAAAAACTTTATATTGGCTGGACTGCGT

>Intergenic_Region|1403414..1403315|Liberibacter_crescens_BT-1_EZ-Tn5_Transposon_Disruption

TAATTTGGCTGTGTTATCTAAATTGAGATAATTAATTATTCTAGTTACTAGGCAAGAGATGCAAAGATATCTACCAAAGATAGTAGCTTTCTATTACAAA

>B488_00700|83974..83875|Liberibacter_crescens_BT-1_EZ-Tn5_Transposon_Disruption

ACTTACGACGCACAACGACAGCGTCAGGCGACATAGATAAAGCTTTCTCATAGAAAAGGGCTGCAGGCTTATAATCCTGCTCCAGATCAGCTATATAACC

>B488_01970|235156..235057|Liberibacter_crescens_BT-1_EZ-Tn5_Transposon_Disruption

ACTGAATCGGTAACGTCTAGTTCAAAGACTTTATGCTTCTCTCCTAGAACAGATTTCAAATTCTCTAAGTCATTGCTACTCCGAGCTGAAAGGACAAGTA

>B488_01690|203599..203500|Liberibacter_crescens_BT-1_EZ-Tn5_Transposon_Disruption

AATAATAACATACCACCAGTCATAAAAATGCAGCGCGGAAAAAAATTCCATGCACGTTTATATAATGGTATTGGAGAACCAACAACCATTCATTGGCATG

>B488_03860|451147..451246|Liberibacter_crescens_BT-1_EZ-Tn5_Transposon_Disruption

GCCTTAAGTACAGTATATATGCATTTTAAGGTTTCATAGTAGCCTCTATAAACAGGGATAATAATATCTACTATTTGTTTTTTTGTTGAACGTATTTTTT

>B488_09580|1031828..1031729|Liberibacter_crescens_BT-1_EZ-Tn5_Transposon_Disruption

GAGCATTTGTTCCTTCATATCGAGGATCATTCGGTGATCCAGGGACAGCAAACACAATTATTCCAAATTCTCCTGCTAGCCTTGCAGTGATTAAAAAGCC

>B488_03240|373688..373589|Liberibacter_crescens_BT-1_EZ-Tn5_Transposon_Disruption

GTCCTATAACGAGAATTCATCATTCCCTACTCTCTCTGTTCTTAACTTAACAAGTTCTCTGTATATAAAAGGAAAATGGAAGCCTATAATTCAAAATATC

>B488_08120|882809..882908|Liberibacter_crescens_BT-1_EZ-Tn5_Transposon_Disruption

CCCTGAGATCGCTTTGATCTGGGACAGTTTTAGTTTTGATATCAAGCGTCACGAAGAACACCATGCTGAAATCGCCAGAATTCATGCCCATCAATTATAT

>B488_12020|1312379..1312280|Liberibacter_crescens_BT-1_EZ-Tn5_Transposon_Disruption

GTATATATTTGAACCAAAAAAAACGTGTTGAAGATACATTGTTAGGATCTGATCTTTGGTATATTTATGCTCTAGCCATAAAGCTAAAAGTACTTCCTGA

>B488_05460|590246..590149|Liberibacter_crescens_BT-1_EZ-Tn5_Transposon_Disruption

GTCCTGGAGAAGTATCTTTAGCCCATAACGGCGTTTTATTTATGGATGAATTCCCGGAATTTTCTCCACAAGCTCTCAATGCACTTCGTCAACCACTT

>B488_08910|968564..968663|Liberibacter_crescens_BT-1_EZ-Tn5_Transposon_Disruption

ATACAATGCTGCACATAAGTGTCAAGTAGATATTCCGTTCGAACGAGTCAAGTACCAGAGTTGTTTTTTATGAGCTCTTGTACCCCCAATGTTGTGCAAG

>B488_04500|503068..503167|Liberibacter_crescens_BT-1_EZ-Tn5_Transposon_Disruption

ATCTGGGACAATGTCAGGAGGAGCATTAACCCAGTTTATGTTGTATTCAATTATTGCTGCAGGATCTATAAGTCTCTTATCAGATATTTGGGGAGAAGTG

>Intergenic_Region|1403414..1403315|Liberibacter_crescens_BT-1_EZ-Tn5_Transposon_Disruption

TAATTTGGCTGTGTTATCTAAATTGAGATAATTAATTATTCTAGTTACTAGGCAAGAGATGCAAAGATATCTACCAAAGATAGTAGCTTTCTATTACAAA

>B488_10610|1156014..1156112|Liberibacter_crescens_BT-1_EZ-Tn5_Transposon_Disruption

AATTTGTACTTGATTAATATTTTCCACACCTTCAGCAATGCAATCCAATCCCATATCCTTACATAACAAGCTAAGGAATTTTACTATTTTATAGCTATTA

>B488_13370|1453106..1453205|Liberibacter_crescens_BT-1_EZ-Tn5_Transposon_Disruption

AAATACCACCGCAACCGCCCATGACAACAATAATGACACCGCTAAAACAAAAAAGAGACTAAACGTAAATTCTCCAGCCGCAGAGTCATTAAAACTGATA

>B488_06210|684934..684835|Liberibacter_crescens_BT-1_EZ-Tn5_Transposon_Disruption

ACCAAACACCCCCTTATTGCAATTCTGAGCGTATATCTTTAACCTTCATAAGAAATAATAAGATTGATTTAAAGTAGGTGTTGATTAAATATAAATACCA

>B488_09530|1026016..1025917|Liberibacter_crescens_BT-1_EZ-Tn5_Transposon_Disruption

ATAAGATACTCGGCATGTCCCCCCTTATTTCTTAACTATAATTTTAATTTTATATTCAAGGGACATATAATTATGAGTAGTATTTTAACCAATGAACATG

>B488_03870|451579..451678|Liberibacter_crescens_BT-1_EZ-Tn5_Transposon_Disruption

GTTTAGTAGGTTATCGATATTTTTTGTAAAAGCCTTGTATTTTTTAAAACGAGAATAAAGCCTTGAGAATAACTTTTTTATTTTTCTAGCCAAAAAATAA

>B488_03820|443897..443798|Liberibacter_crescens_BT-1_EZ-Tn5_Transposon_Disruption

TTTATATACTTGCACCTATAATACGTAACCGAAAAGGAGAATATAAAAAAGAACTCTCAGAATTATCTAAAAAGGGATTCCAACGTGTTAAAATTGACGA

>B488_06100|674398..674497|Liberibacter_crescens_BT-1_EZ-Tn5_Transposon_Disruption

ATTCTGATGCCTGTGCAGACATCAAAATTCCAAACCCCAAGAAAAAAGCTAAAAGTCTAAGAACAACAACACAAATGATAGATAGTAAATAATCTATTAA

>B488_10960|1194416..1194515|Liberibacter_crescens_BT-1_EZ-Tn5_Transposon_Disruption

GCTCTTGTTATCCTTTTTATAACACCTTCAGAAGTCATGGGGCCTAGTTTTCAAATGTCGTTTGCAGCGACTATAGCTCTTGTAGCAAGCTATACAAAGT

>B488_08120|882809..882908|Liberibacter_crescens_BT-1_EZ-Tn5_Transposon_Disruption

CCCTGAGATCGCTTTGATCTGGGACAGTTTTAGTTTTGATATCAAGCGTCACGAAGAACACCATGCTGAAATCGCCAGAATTCATGCCCATCAATTATAT

>B488_06540|723819..723720|Liberibacter_crescens_BT-1_EZ-Tn5_Transposon_Disruption

CCGTTATGGAGCCCCTTTTTATACTTGCATAAAGTGCATACTTTTATTCCTTTCTTGTCAGCAACCAAATCTACAACAAACTCTTCACCATCAATTTTTT

>B488_08060|878817..878718|Liberibacter_crescens_BT-1_EZ-Tn5_Transposon_Disruption

GTTTAAGTAACTGTGTGGCGATATGCAATCCAGTCCCCCGAGTGGCGCCAACAACGGCAAAAACATCACTGGACATGCTTGAAATTTCTCCTGCTCGCGT

>B488_04510|504899..504800|Liberibacter_crescens_BT-1_EZ-Tn5_Transposon_Disruption

GTATAGAAGAATAAAATTTAAGCGCATTTCCACCAGTTGTTGTCTCTGGTGAACCAAACATAACACCTATTTTCATTCGCATTTGATTAATAAAAATCAA

>B488_13500|1471274..1471175|Liberibacter_crescens_BT-1_EZ-Tn5_Transposon_Disruption

AGTCCATTGTTACCAGTAGAGATCCCTTTGAACAAGGGATGTGGTTGCAAGAGATCTAATGTATTCCAGCCAACTTGCGGTATTTTAAGGTTATATTTAT

>B488_01350|167238..167139|Liberibacter_crescens_BT-1_EZ-Tn5_Transposon_Disruption

GTCTATAGTTGTTTATCATTGGATCAAACTCTTTATCTTAAATCATATAAAGATTCAGGAAAAGATGTTCGCAATAAAATGAAGTTTGCATATGTTTCTT

>B488_07690|849017..849116|Liberibacter_crescens_BT-1_EZ-Tn5_Transposon_Disruption

TTCCTGATACCCAAAACCCATGCCAACAGAGTGAAAAGAATAAAACTCCCTGCGAGAAGAAAAATTCTTTGCACTCCTGATAACAGCATCATCGCAGATA

>Intergenic_Region|1390323..1390422|Liberibacter_crescens_BT-1_EZ-Tn5_Transposon_Disruption

GTATAAAATCTACTGAATTGGAATAAACCTCTCCCTTATAGGGTAGTTTTTCTAATCTAAAAATTAATATTCCTTTTCCATAAAATACCTATAATAAATA

>B488_12760|1390967..1391066|Liberibacter_crescens_BT-1_EZ-Tn5_Transposon_Disruption

CTTATAGACCAGGCAATTGGGAAAATGGCAGATGTGAAAAAAAGCTAAAAAATAGAATAATTATATTCTTATAATACCATTAGAAGCTGGCGATTTCCTA

>B488_12550|1370056..1369957|Liberibacter_crescens_BT-1_EZ-Tn5_Transposon_Disruption

GCTAAGATTGCACCAGCCATTGCGGCTATTTCTCTGCCTCCAAGTCGACGCATTAATTCAAAGGGATCAGAAAGATGATTACGGTGGAATGCTACGGCTT

>B488_02420|276943..277042|Liberibacter_crescens_BT-1_EZ-Tn5_Transposon_Disruption

TATCTCATGATTATCCATCCTCTTTCCAAAGAAGAACCGTTGCAGTAAGGGATATGGCAACAATTATTGGCACTGATGTTACTATCAGAGGAGATATGAT

>B488_10290|1126216..1126315|Liberibacter_crescens_BT-1_EZ-Tn5_Transposon_Disruption

ACCCAATACCGCTCCTGACATTGATCCAATGCCCCCTAAAACGGCTGCTGTAAAGGCTTTAATCCCAGGAAGATATCCTGAATAAAAAGAAATCACACCA

>Intergenic_Region|329143..329044|Liberibacter_crescens_BT-1_EZ-Tn5_Transposon_Disruption

CTAGTACTCCTCGGTATTCGGAGTTTGGTTAGGAGCAGTAAGGCGGTGAGCCCCCATAGCCTATCCAGTGCTCTACCCCCGAGGGTATTCAGTTAACGCT

>B488_04010|464485..464584|Liberibacter_crescens_BT-1_EZ-Tn5_Transposon_Disruption

GTGGATACAGAAATTCATTATCAAGGAAATGCTTATTCTTGGAAGAAAGGTGAAGCTCCACCACTTATGTTTGAAAAACTTTATATTGGCTGGACTGCGT

>Intergenic_Region|330202..330103|Liberibacter_crescens_BT-1_EZ-Tn5_Transposon_Disruption

ATCCTACACATACCTACACAAATGCCAGTGTAAAGTTATAGTAAAGGTGCACGGGGTCTTTCCGTCTGACCGCAGGAACCCCGCATCTTCACGGGGAATT

>Intergenic_Region|330194..330293|Liberibacter_crescens_BT-1_EZ-Tn5_Transposon_Disruption

GTGTAGGATAGGTGGTAGGCTTTGAAGCAAGGGCGCCAGCTTTTGTGGAGCCATCCTTGAAATACCACCCTTATCCACATGGATGTCTAACCGCGGTCCG

>B488_10300|1127456..1127555|Liberibacter_crescens_BT-1_EZ-Tn5_Transposon_Disruption

GTTTTAGAGAGTACAATGGCCGAAAAAATGCAATCCCGAATAAAATATGTAATTGGTCCAGATGGAAGTCCACTAACCATAGCAAACCTACCTCCACCAA

>B488_06490|719487..719388|Liberibacter_crescens_BT-1_EZ-Tn5_Transposon_Disruption

GTGTATATGTGTTTTGATTAAATATACAATAGGAAGCGTAGAAAGATTCTGTACAGAAATACTAGTTGCATTAATAATTTGTTCTATATTTTCAGTGGAA

>B488_10290|1126216..1126315|Liberibacter_crescens_BT-1_EZ-Tn5_Transposon_Disruption

ACCCAATACCGCTCCTGACATTGATCCAATGCCCCCTAAAACGGCTGCTGTAAAGGCTTTAATCCCAGGAAGATATCCTGAATAAAAAGAAATCACACCA

>B488_01360|167817..167718|Liberibacter_crescens_BT-1_EZ-Tn5_Transposon_Disruption

GCCTTGGATTCATAAGAATTGTCTTGTTACCAGAGATACGAACTAAGTAATTTTAAGTTGTATAGCGTTTTAAAATTTTATATTATTGTTACAATTTAAG

>B488_03450|396634..396535|Liberibacter_crescens_BT-1_EZ-Tn5_Transposon_Disruption

ACGTGGAGTACCTCGCGCTCGCATAGCAATTTCATGTGCTCCTTCATCTGTCATAGACAACCCCATAATCGAAGAACCACGCTTAACTATTAATTCCAAT

>B488_12250|1339482..1339581|Liberibacter_crescens_BT-1_EZ-Tn5_Transposon_Disruption

GGATACAGGCACTGAAGATTAATGCCAAACCTTCTAGAAATGATAACCAAATTTCGAAGAAATTCATTCTCTTTCTCTGTAAAGATCAGGATATACTGAT

>Intergenic_Region|326377..326278|Liberibacter_crescens_BT-1_EZ-Tn5_Transposon_Disruption

GTCCCAGTGTGGCTGATCATCCTCTCAGACCAGCTATGGATCGTCGCCTTGGTAGGCCTTTACCCCACCAACTAGCTAATCCAACGCGGGCTCATCTCTC

>B488_00110|14987..15086|Liberibacter_crescens_BT-1_EZ-Tn5_Transposon_Disruption

CACCAGAACAATGCCGTCTTATTATGATTGATCCAAAGATGTTAGAATTGTCTGTCTATGATGGAATACCACATCTTCTCTCACCAGTTGTTACTGATCC

>B488_02780|316826..316727|Liberibacter_crescens_BT-1_EZ-Tn5_Transposon_Disruption

GCTCAGCAACACCCATTGAACGCGCAAAAATTATAGCATTCGAAGGTACAGGACTATTAGAAGAATGATTATCATCTCTTATCAAATGACGTAATAAGCG

>B488_05420|586761..586662|Liberibacter_crescens_BT-1_EZ-Tn5_Transposon_Disruption

GTTCTTTGCTAGCCTGAATGTGAGCTTCGCAGGAACACTGTATATATTTATAACACTTGACATGACAGAAACAGGTAATCTTTTATTGCTTCAAGGATTC

>B488_06070|670778..670679|Liberibacter_crescens_BT-1_EZ-Tn5_Transposon_Disruption

CTCTTATACAACCTAGAATCATACCATCTCGTACAACTGGTTATTTAATTGGTAAACAAGCGGCTGCTGATCTTTTAAACAGTCGAAAAAAAATATTTCG

>B488_12120|1325208..1325307|Liberibacter_crescens_BT-1_EZ-Tn5_Transposon_Disruption

TCCTAAATCCGCGCTAACTACAGCCTGATCTATAATCATCTGTAAACCCAAGGGTGAAAGAATTGCCATAACTTCAAGACAAAGAGCCATAGAGAACATC

>B488_11850|1294625..1294526|Liberibacter_crescens_BT-1_EZ-Tn5_Transposon_Disruption

GTAAGATTACAGTTTTAGATGTTGTTAAAGTCACTGAAGAATCGTTTTATATGGCATCATGTTTTGAAGAAGGAGTCAAAGATTGTCCGCTTACTGGTAA

>B488_03760|434038..433939|Liberibacter_crescens_BT-1_EZ-Tn5_Transposon_Disruption

GTCTTGTATCATTGCAGATATATGCAAATCTCCAGAGAATGTATCAACACTATTAACATAAGTCATCATTAAGCAGTATAATTTGTTATACTGCTTGATT

>B488_01670|201079..200980|Liberibacter_crescens_BT-1_EZ-Tn5_Transposon_Disruption

GTATAAAATACATCACTTAGTATCATGATTTTTGCTTTTTTTCTAAAATATTTTCCATAAAAAGACTAAAAAGATAATAGCTATCTTGAGGACCAGGAGA

>B488_13030|1415221..1415320|Liberibacter_crescens_BT-1_EZ-Tn5_Transposon_Disruption

CCTATAAAAGGGGAACCATCTCCTTTCTATAATACAAGGGGTGTTATGAGCCATCGGCTTGGATCCTTGGTCAAGGATACCTACCAATGGTTTGTAAGTC

>B488_11600|1260273..1260174|Liberibacter_crescens_BT-1_EZ-Tn5_Transposon_Disruption

AGGCTGTAGGTTTGGCGCAGGCAATAGATTTACAAGTAGTGCGTTCTTTTGTGGTTGCTGTTTCAAGACCTAGCCCTGCAACTCTTATAAGAAAAGGAAA

>Intergenic_Region|329143..329044|Liberibacter_crescens_BT-1_EZ-Tn5_Transposon_Disruption

CTAGTACTCCTCGGTATTCGGAGTTTGGTTAGGAGCAGTAAGGCGGTGAGCCCCCATAGCCTATCCAGTGCTCTACCCCCGAGGGTATTCAGTTAACGCT

>B488_03240|372154..372253|Liberibacter_crescens_BT-1_EZ-Tn5_Transposon_Disruption

TGTCGTGTGTAATGAATATATACGATATGCCAAGGCTTTCTTGTAGTTCCATGAGGAGATTGCATACCTGTACTTTAATTGATGAATCAAGAGCAGAGAC

>B488_07710|850001..850100|Liberibacter_crescens_BT-1_EZ-Tn5_Transposon_Disruption

GTTCTTTCCATGGAAACAGAGGACCAGGATCATTCTTTCGGGTATAGGCAATATCGGAATGGGCCAAAACATGGGTGGGTGTAATATACGGATAACGCTG

>B488_12250|1338241..1338340|Liberibacter_crescens_BT-1_EZ-Tn5_Transposon_Disruption

AATTTGTGTATCATCATGTTTCATATTCTCAACACTAAAGAAATTTTTATAACGTTTGAACCCTATGTAATTCTCAGGTGACTGGTTTCTGCTAATAGCA

>B488_02290|263544..263445|Liberibacter_crescens_BT-1_EZ-Tn5_Transposon_Disruption

CTTCTGCCCTACAAATACACGTAAAGACAAAACACTATTTTCAGAAGTGTCTATACCTTCTGTTTTTCCAAGACGGATTGAAAAGCTTTTATCGCGTGAA

>B488_06570|726657..726756|Liberibacter_crescens_BT-1_EZ-Tn5_Transposon_Disruption

TACTTCTTCTGTTGAGTCTCTGGCAACATGGACTTGAACTTTTTGTTCTTCATCTATTTTTTGTTTTGTAAAAAGGGTTTTATGCAGTCGATTCTCATTA

>B488_12020|1312975..1313074|Liberibacter_crescens_BT-1_EZ-Tn5_Transposon_Disruption

GTAATGATGAACCTGTACGTATTGGAAATTGGACACCTCAAAACTATGATCATAAATATCGTGGACCTGTAACCTTGACAACAGCACTCTTGAATTCTCT

>B488_05830|639619..639520|Liberibacter_crescens_BT-1_EZ-Tn5_Transposon_Disruption

CATCAAGTACTTGCCGCAGATCAGGGAATTTTAATGTCAGGAACCCCCTTAACAATGGCAGAAACAGCTTCTATTTTTGGAGAAACACTAACTTTTCAGG

>B488_13100|1422249..1422150|Liberibacter_crescens_BT-1_EZ-Tn5_Transposon_Disruption

GGCGTAATCAGAAGGACTTTTTCATGCAAGCGATGCTGGAACATCTCTATCAGGGCGGGCGTATCAGCCGTGAACAAAGCCAGCAGCTGTTCGGGGCCAT

>B488_13290|1443089..1442990|Liberibacter_crescens_BT-1_EZ-Tn5_Transposon_Disruption

GACGAATCCAGAGCAACTTTTTGCTGTAGGGTATAGCGCTTGTTTTTTGGGAGCAATGAAATTTGTTTCTAAACGTGACAAGCTTCCACTTCCTACTGAT

>B488_07050|784784..784685|Liberibacter_crescens_BT-1_EZ-Tn5_Transposon_Disruption

GTCAAAGGCAAAGCGCAGATCCTGACCGTGCAAGTCCGGGTTCATGCTCTGCTCGGTGAATACATCATTCTCATCAACGATGAATACATCATTATTGAAC

>B488_03240|373470..373371|Liberibacter_crescens_BT-1_EZ-Tn5_Transposon_Disruption

GTAAAATCCTGCTAGATGGACAAGATATATTATCACTCCCTTTAAAAAAAATGCACAAAATTCGTGGCAATAAAATTGGAATGATTTTTCAAGAACCAAT

>B488_13280|1442190..1442289|Liberibacter_crescens_BT-1_EZ-Tn5_Transposon_Disruption

CCATCATCTAAACCAAAGGTTGGAGTTCTTGGTATCGAATTATCTTCAGATGGTAGTCAGTATGACTTAATAAAAATTCCTGCAGGCGGAAGTACCCGAC

>B488_01600|193590..193689|Liberibacter_crescens_BT-1_EZ-Tn5_Transposon_Disruption

ATGTGATAGATAATAAAGGTAGAATTTTAGCTGCATCAAATAAAATACATTTGATACCTTTTATTGAATATTTGCCTTATAAGGATTTTTTTAAGGTTTT

>B488_06810|757103..757005|Liberibacter_crescens_BT-1_EZ-Tn5_Transposon_Disruption

CTATACAAGTAGCTGGAGCATCACATGCGTGCCTCGAATTGCTTTATGCTGATGATAACAAACTATTTCTTCCTGATGAAAATATTGAACTATTATCTCA

>B488_04780|531350..531251|Liberibacter_crescens_BT-1_EZ-Tn5_Transposon_Disruption

AGTCACAACTGCCGAATTTGGAACTATAACATATACGTCTAATGAGCCAAGTAAAGTAAATATCACCAGCCCGGCTATTGGTGGTTTTGCAGGTTTTAAT

>B488_06280|691828..691729|Liberibacter_crescens_BT-1_EZ-Tn5_Transposon_Disruption

GACCTGCAGTGTCCTTGTAGACATGTGCATCAAAGCCCTTCCAGTGATTTGATTGACTCCGCCGTATGATCCTGAAGACCCTGAAAGATAATCAATCCAT

>B488_10900|1185312..1185213|Liberibacter_crescens_BT-1_EZ-Tn5_Transposon_Disruption

CCGCAAGCCTATCCCTGAAGAGGATTTTTTATCAAATTAATAGATAATCTATTTCAACACGGCATAGGGTATTGTATTTTTTAATGTGCCCTTCTAAGCT

>Intergenic_Region|721990..721891|Liberibacter_crescens_BT-1_EZ-Tn5_Transposon_Disruption

GTTCTAAACTGGATTATTTAAACTTGTTTAATAAAGAATTTTATACTTTAAATAGCAATATCTTTCAAGATATTGCCTCCCGATTCTAACTGTTTTTTAG

>B488_00130|17124..17025|Liberibacter_crescens_BT-1_EZ-Tn5_Transposon_Disruption

GAATAGATTTCATCTCTTCAAGAAATTTAATCTTATAAGAAAATTTTGGATTTTTTATAGGATCAGGCTCATATCCTCCTGCAGGCACATAAAAATTATG

>B488_13160|1429406..1429505|Liberibacter_crescens_BT-1_EZ-Tn5_Transposon_Disruption

CTGCTATACCGAACGATCTCGTGTAAAAGTTTTTAAAACTATGTGGATTATAGGCAAAAAAAGAGCATATATTACCCGGGCCAATGCATCAACACTTCGA

>B488_11610|1263346..1263444|Liberibacter_crescens_BT-1_EZ-Tn5_Transposon_Disruption

GCCTCACTGTTTCTAATATAATACGATTCTGCACTCACATTCACAGGAACCGTAATCCCGTAGGAGTACGCAATCTGTTGACTGAACGCCTGATAATTAA

>B488_02370|270941..270860|Liberibacter_crescens_BT-1_EZ-Tn5_Transposon_Disruption

CTCCTGGAGTTTGATGTCACGTGTTGCTCAAGTACTTGGAGAAAGGTTTAAGATAATTATATCGAATTGACTAAATTATCGGCATAGGAAGAATATACAG

>B488_05900|649130..649229|Liberibacter_crescens_BT-1_EZ-Tn5_Transposon_Disruption

TTTCATACCTTGGGCACCTTCAGTTAAAACAAGTTCAGCTCCGAGCAAGGCCAACATTTTACGACGCTCAAGAGACATAGTTTCGGGCATCGTAAGAATA

>B488_05190|562433..562334|Liberibacter_crescens_BT-1_EZ-Tn5_Transposon_Disruption

CATTAAGGTTGCCAGTAATATTAACAGTTCCCTTTGACATATCCAGCTTCGTTTCTATTGTTTTGCCATCGGGTGAGCGTTTCTCAATCACAAGAAAGTT

>B488_09240|997325..997424|Liberibacter_crescens_BT-1_EZ-Tn5_Transposon_Disruption

CGAATGTTCTGTGTATCCAGATTATCAAAAACAACGCGAATATATTGGTATTATTCATAAATCTGGAACTCATTTACTGAGTTTAATTAACCGCATGCTT

>B488_09240|997325..997424|Liberibacter_crescens_BT-1_EZ-Tn5_Transposon_Disruption

CGAATGTTCTGTGTATCCAGATTATCAAAAACAACGCGAATATATTGGTATTATTCATAAATCTGGAACTCATTTACTGAGTTTAATTAACCGCATGCTT

>B488_04480|501236..501335|Liberibacter_crescens_BT-1_EZ-Tn5_Transposon_Disruption

TTTTTATACATATGGAAGTACTTCGCCAATTTGGCTTGACATGGATTTATCCAAAACAAACAGTTCTGGTTCGATTTGGTCATACTAAAAAAGGTCTGAT

>B488_06170|680209..680110|Liberibacter_crescens_BT-1_EZ-Tn5_Transposon_Disruption

GGCTTGAGCTTCAGATATTGCATGAAGAATTAACATCTTCAAAGGATGATAGCTCTCATTTTAAATCATTATCTTAATTTTTAGCACGCTCAATGTAAGA

>B488_13030|1414984..1414885|Liberibacter_crescens_BT-1_EZ-Tn5_Transposon_Disruption

CTGTTCCTCCTGGTGAAGATATAGATACAACTAATGCTTTGACGTTCTTATCTTCAGCAACTCCCCCCAGAACTCTCAATAATTCAGGGTTATCTTCTAT

>Intergenic_Region|1122631..1122730|Liberibacter_crescens_BT-1_EZ-Tn5_Transposon_Disruption

ATTTTAACCCCTTCAGTAGCTTACATCACATGTTAAAAACATCAGAAGACATCTAATTGTTTTTAACAACGCTTTATATAAAGATATGTAGTATTATCAA

>B488_03220|370465..370564|Liberibacter_crescens_BT-1_EZ-Tn5_Transposon_Disruption

GTGTAGGTGTTGTTGTTACTGATTTAAAAGCAGGTTCTATTGCAAGTCGATTGGGTTTTTCTCCTCGGGACATTATAATTACTGTTAACAATACGCCAGT

>B488_13470|1468385..1468286|Liberibacter_crescens_BT-1_EZ-Tn5_Transposon_Disruption

TCCCAGTTCAGGTAAAATTGAAATCTTTAAACAAAGATGATTTTCGTCGTATTTTAATAGAAACAGAGTCAAACCTTATTATACAATATAAATCTTTGAT

>B488_12240|1337170..1337269|Liberibacter_crescens_BT-1_EZ-Tn5_Transposon_Disruption

GAGATAACTGTTGCAACAAAACTGGGCAGCCCGGAACCATCAGAAAATTCTCGTCTACGCCTTGCTATTCAAAATGCTAAAGTTCATTCAATGCCTAAGG

>B488_10820|1179956..1180055|Liberibacter_crescens_BT-1_EZ-Tn5_Transposon_Disruption

CCAAGCAGACGGGCAACATATAAATTCAGATGTACTTACATCAATAAGTATAGTTTTCGGGTTGATTATAGTTATGGTAACCGGCTACACTATAGTTGAT

>B488_06100|673496..673595|Liberibacter_crescens_BT-1_EZ-Tn5_Transposon_Disruption

ACCGTGATGTCATGGAGTTGCAACTTTATAAAGCGTACGAGATAATTTTTCTATTGCTGAGATATTTTCAGGTCCTGGTGTGAGTTCACTATATTTTAAT

>B488_03860|449874..449775|Liberibacter_crescens_BT-1_EZ-Tn5_Transposon_Disruption

TTTCGTTCCTGATCTTGATGTCTCTCAACGATTAAAGAGATATTTCAGAGACTTAGATTTTACGGTTAAAGAACATCCCTTCATAAATTTATATGATGAA

>B488_10540|1149368..1149467|Liberibacter_crescens_BT-1_EZ-Tn5_Transposon_Disruption

TTTCACAGCTTATCAAAGCTATTGAAACAGATGTAAATATCATTAGCGGTGCTATTGACGAAATAGATGGTGAAGCTTTCGGTTCCTTAGTCATTTCATA

>B488_13250|1439468..1439567|Liberibacter_crescens_BT-1_EZ-Tn5_Transposon_Disruption

AGAAGAACCAGAGAGACGAGAAAAACTTCATGATCTCATTTCTTTCACAGATAGGCTTTTTAACAATATTTTCGGATTCTCAAGTGGTTCTCAAATTCAG

>B488_10390|1135789..1135888|Liberibacter_crescens_BT-1_EZ-Tn5_Transposon_Disruption

CATATTGACACTCCTTAAAATATTTTTTGCAGAATTCATAATGACCTATAGGCTGAGAAGTTGGGCCTGAAATCAGCATTTTCGATTTTTCTTGAGCATT

>B488_06280|691828..691729|Liberibacter_crescens_BT-1_EZ-Tn5_Transposon_Disruption

GACCTGCAGTGTCCTTGTAGACATGTGCATCAAAGCCCTTCCAGTGATTTGATTGACTCCGCCGTATGATCCTGAAGACCCTGAAAGATAATCAATCCAT

>B488_03240|372154..372253|Liberibacter_crescens_BT-1_EZ-Tn5_Transposon_Disruption

TGTCGTGTGTAATGAATATATACGATATGCCAAGGCTTTCTTGTAGTTCCATGAGGAGATTGCATACCTGTACTTTAATTGATGAATCAAGAGCAGAGAC

>B488_04700|526581..526482|Liberibacter_crescens_BT-1_EZ-Tn5_Transposon_Disruption

GTTTCATACCGAGTCAACAAAGGAACTGGTCACGGTTACCTGTATCATTTCTCACAAGGATGGGCATCGTGAAAGAAACAGCCTTTCTGCTCCTCCTGAT

>B488_11850|1294625..1294526|Liberibacter_crescens_BT-1_EZ-Tn5_Transposon_Disruption

GTAAGATTACAGTTTTAGATGTTGTTAAAGTCACTGAAGAATCGTTTTATATGGCATCATGTTTTGAAGAAGGAGTCAAAGATTGTCCGCTTACTGGTAA

>B488_03970|460128..460029|Liberibacter_crescens_BT-1_EZ-Tn5_Transposon_Disruption

CGTCCAAATGAACATATTATTCAAGGGAGTTTGAATTATTCATGGGATACCAACGATATCCATTTCATTCAAATGCAAAATCATCCTTTCTTTAAAGAAC

>B488_01570|190152..190251|Liberibacter_crescens_BT-1_EZ-Tn5_Transposon_Disruption

GGTTAGGGCTGTTCGTTCTGATATAGCTATTTCAAGTGATTTTATTGTTGGTTTCCCTGGAGAAACAGATAGTGACTTTGAAGAGACTATGCATCTTGTT

>B488_12020|1312975..1313074|Liberibacter_crescens_BT-1_EZ-Tn5_Transposon_Disruption

GTAATGATGAACCTGTACGTATTGGAAATTGGACACCTCAAAACTATGATCATAAATATCGTGGACCTGTAACCTTGACAACAGCACTCTTGAATTCTCT

>B488_07630|846357..846258|Liberibacter_crescens_BT-1_EZ-Tn5_Transposon_Disruption

TCCTGGTACAAAGGTATTCGGTAAGCCATTTTCCTCTGTTTTTATAGGAATATCACGTGTTAATGCTGTTTCAGATAGTGCTCCAAGTGTTTTCAGGGGA

>B488_13630|1484458..1484557|Liberibacter_crescens_BT-1_EZ-Tn5_Transposon_Disruption

TGCTAAAAGTCTAGCAGAATTAATTGATACAATGGAAGCAGAAGAAAAGAATTTAGAAAATCTTCATTTTTTAAATCATGAAAAATATGGTGACTGGTGG

>B488_07950|872239..872338|Liberibacter_crescens_BT-1_EZ-Tn5_Transposon_Disruption

CTTTTCGGGATTGCTGTGTCTGTGATCGGCCTGTTGCTTTCATATCATTACAGCCTGCCTTCCGGTCCGGCAATCATCCTCACTGCAAGTGTATGGTACG

>B488_01950|233506..233407|Liberibacter_crescens_BT-1_EZ-Tn5_Transposon_Disruption

ACGCTATAGGCACCGACTGCAATAATCATACAAAGGATAAACCAAATCACAGGCGAAAAAAATACAACATTAAAAAGCCCGAATATCGATAGGGAGAGTA

>B488_05420|586761..586662|Liberibacter_crescens_BT-1_EZ-Tn5_Transposon_Disruption

GTTCTTTGCTAGCCTGAATGTGAGCTTCGCAGGAACACTGTATATATTTATAACACTTGACATGACAGAAACAGGTAATCTTTTATTGCTTCAAGGATTC

>B488_06790|753070..752971|Liberibacter_crescens_BT-1_EZ-Tn5_Transposon_Disruption

TAACAGAAGTGTTGAGCAACCTAAATGGCGAAAGAAACTTTCGACTGTATTTAGACGCGTTCGCAATTTTTTATTGAGACGAGGCAACTAAAAATGAAGA

>Intergenic_Region|329143..329044|Liberibacter_crescens_BT-1_EZ-Tn5_Transposon_Disruption

CTAGTACTCCTCGGTATTCGGAGTTTGGTTAGGAGCAGTAAGGCGGTGAGCCCCCATAGCCTATCCAGTGCTCTACCCCCGAGGGTATTCAGTTAACGCT

>B488_06570|726657..726756|Liberibacter_crescens_BT-1_EZ-Tn5_Transposon_Disruption

TACTTCTTCTGTTGAGTCTCTGGCAACATGGACTTGAACTTTTTGTTCTTCATCTATTTTTTGTTTTGTAAAAAGGGTTTTATGCAGTCGATTCTCATTA

>B488_04940|543017..542918|Liberibacter_crescens_BT-1_EZ-Tn5_Transposon_Disruption

CTTTAATACTTCTTCTATTTTTTCTCTTTGTTTTTTATCAACTCTAAACTTCTCAACAGAAAGTGCTTTATAAACACCTTCTTTATTGTTTGCGTCATTG

>B488_10530|1147859..1147760|Liberibacter_crescens_BT-1_EZ-Tn5_Transposon_Disruption

TGTTATGAATGCTTGGGGGAAGAGTCAAAATCTTAAGAATGTTAAGTTAATTCCAGATGGTTCTGGTGAGTTTACACGCAAGATGGGGATGCTTGTTTAC

>B488_06810|757103..757004|Liberibacter_crescens_BT-1_EZ-Tn5_Transposon_Disruption

CTATACAAGTAGCTGGAGCATCACATGCGTGCCTCGAATTGCTTTATGCTGATGATAACAAACTATTTCTTCCTGTTGAAAATATTGAACTATTATCTCG

>B488_05830|639619..639520|Liberibacter_crescens_BT-1_EZ-Tn5_Transposon_Disruption

CATCAAGTACTTGCCGCAGATCAGGGAATTTTAATGTCAGGAACCCCCTTAACAATGGCAGAAACAGCTTCTATTTTTGGAGAAACACTAACTTTTCAGG

>B488_01760|213135..213036|Liberibacter_crescens_BT-1_EZ-Tn5_Transposon_Disruption

GTATTTCCATACCTTTACTTATATATTCCGGTCACCGTATATACTACACAAATTTAGCTTTCCTTATACTTACTGGCTATAAATCGCATGAAGAGATTGA

>B488_06780|751266..751365|Liberibacter_crescens_BT-1_EZ-Tn5_Transposon_Disruption

GACCAAGCTGTGCAGCTCAGTATACCTGGAGAGTAATAGTCAAGCAAATATTTATGATCCTTGACAAATTGTCTTATGCCCATCTGTACTTTCATTGCTT

>Intergenic_Region|329143..329046|Liberibacter_crescens_BT-1_EZ-Tn5_Transposon_Disruption

CTAATACTACTCGACTATTCTGGAGTTTGGTTAGGAGCAGTAAGGCGGTGAGCCCCCATAGCCTATCCAGTGCTCTACCCCCGAGGGTATTCAGTTAACG

>B488_04480|501236..501335|Liberibacter_crescens_BT-1_EZ-Tn5_Transposon_Disruption

TTTTTATACATATGGAAGTACTTCGCCAATTTGGCTTGACATGGATTTATCCAAAACAAACAGTTCTGGTTCGATTTGGTCATACTAAAAAAGGTCTGAT

>Intergenic_Region|1122631..1122730|Liberibacter_crescens_BT-1_EZ-Tn5_Transposon_Disruption

ATTTTAACCCCTTCAGTAGCTTACATCACATGTTAAAAACATCAGAAGACATCTAATTGTTTTTAACAACGCTTTATATAAAGATATGTAGTATTATCAA

>B488_12770|1391294..1391350|Liberibacter_crescens_BT-1_EZ-Tn5_Transposon_Disruption

GCTCAATATCGCTATCTTGCTTCCTCTCACCATTAACATCTAACCATATTGATCCTTCAACTCAGCAAAAGTTCCAGTTACCGGTATCTAAAAAAAAAGA

>Intergenic_Region|860214..860313|Liberibacter_crescens_BT-1_EZ-Tn5_Transposon_Disruption

GGTCTGGTACCTCCCCATTATAGGAGAAAATTCTTTCATTATCAAGAGTTTTATCCTATATGTTCTTTTTTTGTTTACTTTCCTGTACAACAGCCTCACA

>B488_11610|1263346..1263445|Liberibacter_crescens_BT-1_EZ-Tn5_Transposon_Disruption

GCCTCACTGTTTCTAATATAATACGATTTGCACTCACATTAACAGGAACCGTAATCTTAGTAGGAGTCGCAATCTGTTGATTGAATGTTTGATAATTAAA

>Intergenic_Region|1267101..1267155|Liberibacter_crescens_BT-1_EZ-Tn5_Transposon_Disruption

GAACAGTACTGGGTGATTAGCTCAGTTGGTAGAGCAGCTGACTCTTTTTTTCATCCTTACAATCTCCTATTTCATTCCCATTCAATGCATAAATATATAT

>B488_03940|457420..457321|Liberibacter_crescens_BT-1_EZ-Tn5_Transposon_Disruption

GTTCTCTATACACCACGTAATGTCTTAAAGCGTGTCCTAGAACTTTATAAAAAAAATTCTCTTAATCCCATCATTGCTCCAGAAATTGAGTTTTATCTTA

>B488_08800|958995..958896|Liberibacter_crescens_BT-1_EZ-Tn5_Transposon_Disruption

CCTTATAATTTAGCACTCATGGCTTCTGATGTTGTTGATCTCCTTGATTATTTAGGAGTTCGGACTGCTCATATTATGGGGTATTCAATGGGTGCGCGTA

>B488_13280|1442190..1442289|Liberibacter_crescens_BT-1_EZ-Tn5_Transposon_Disruption

CCATCATCTAAACCAAAGGTTGGAGTTCTTGGTATCGAATTATCTTCAGATGGTAGTCAGTATGACTTAATAAAAATTCCTGCAGGCGGAAGTACCCGAC

>Intergenic_Region|534977..535076|Liberibacter_crescens_BT-1_EZ-Tn5_Transposon_Disruption

GTATAAGAATGAGCAAAACATTCGTCAGTCCGAGATAAGCAACAGAAAAACCAAGTCCAAAGACAACAATGCAAGGTAAAATAAAGTATTTAACCAGTTT

>B488_03220|370465..370564|Liberibacter_crescens_BT-1_EZ-Tn5_Transposon_Disruption

GTGTAGGTGTTGTTGTTACTGATTTAAAAGCAGGTTCTAATACAAGGGGTTTGATGAGCCCAACTCGGGACATTATAATTACTGTTAACAATACGCCAGT

>B488_07980|873962..873863|Liberibacter_crescens_BT-1_EZ-Tn5_Transposon_Disruption

GTATACCCATGCGCATGCAGATCATGTGCATGGGATTGATGACTTGCGCGGATATTATCTCAAGACAAAAAAACCTGTCCCTATCTATGCCGATAAAGAG

>B488_11600|1260273..1260174|Liberibacter_crescens_BT-1_EZ-Tn5_Transposon_Disruption

AGGCTGTAGGTTTGGCGCAGGCAATAGATTTACAAGTAGTGCGTTCTTTTGTGGTTGCTGTTTCAAGACCTAGCCCTGCAACTCTTATAAGAAAAGGAAA

>B488_05080|551974..551875|Liberibacter_crescens_BT-1_EZ-Tn5_Transposon_Disruption

GCTTTCTGTGCTGCTTTTTTCTCCGCTTCCTCCATCTTCAGACCTTTCATGGCATAATGTGTGAGGACGTAATAGCGATGGTCAGAAAGCAAAGAGGGAA

>B488_01820|223683..223782|Liberibacter_crescens_BT-1_EZ-Tn5_Transposon_Disruption

ATTTAAAGGTCTTTGTGAGTTTTCTTTGTAGAAATCACTAAAGGCCATCGTGGCTTCCTTTTCTTGATTATAGCGCTATAAAAGAAAAAATTCTTATAAA

>B488_09000|686460..686361|Liberibacter_crescens_BT-1_EZ-Tn5_Transposon_Disruption

GTACAGAACTTTATGGTTCTATAAGAGCCCGCTTAGGATATTCACTTGATAGGGCCCTCGTATATGCTACGGCTGGGTGGTCTTTTAGCAAGGCCAAGTT

>B488_07990|874340..874241|Liberibacter_crescens_BT-1_EZ-Tn5_Transposon_Disruption

GACGTAACGAGCCTTCCTATGTCGTGCATACGGCGAAAGTCCTTGCAGAGATCAGGAAGATTGATTATGATCAACTATCGGTTATTACAACCGAAAATGC

>B488_09000|686460..686361|Liberibacter_crescens_BT-1_EZ-Tn5_Transposon_Disruption

GTACAGAACTTTATGGTTCTATAAGAGCCCGCTTAGGATATTCACTTGATAGGGCCCTCGTATATGCTACGGCTGGGTGGTCTTTTAGCAAGGCCAAGTT

>B488_07740|853422..853521|Liberibacter_crescens_BT-1_EZ-Tn5_Transposon_Disruption

CATCAGGAGTCACCTGGTTGATATTGCCAATCGCTGAACTGCTGATCATGGAACCGATATTGCCTGACATCTGCATGGATGCCATGTGACGCGGATCAGG

>B488_11660|1272106..1272007|Liberibacter_crescens_BT-1_EZ-Tn5_Transposon_Disruption

GCTCTAACATCTTGGGATCTACCATAATCATACGGCATTCCTTGGGTTGAAGACGATAGAGCAAAGATAAAATCATCGTATTGATGGCAACAGACTTCCC

>B488_10750|1173342..1173441|Liberibacter_crescens_BT-1_EZ-Tn5_Transposon_Disruption

CCATTGATTACTCAAAAATCCTAACATTTTGTATTGTAAAGAAATTACCGTTGTAACAGTAAATACTTTTGGATCAGCTACGCTTGGTATAGGACCTGGA

>B488_13150|1428977..1428886|Liberibacter_crescens_BT-1_EZ-Tn5_Transposon_Disruption

GTATTAAAGATATAGCTTTAAGAAAGGCATTAATGCGCCTTGGGTATGGTGTTCTTAGAAGCAAAACTGTTATTTATAGGGGTCGGTTTTCCTTAATTGT

>B488_10900|1185312..1185215|Liberibacter_crescens_BT-1_EZ-Tn5_Transposon_Disruption

CCGCAAGCCTATCCCTGAAGAGGATTTTTTATCAAATTAATAGATAATCTATTTCAACACGGCATAAGGTATTGTATTTTTTCCTGTGCCCATCAAAGAA

>B488_04920|540542..540641|Liberibacter_crescens_BT-1_EZ-Tn5_Transposon_Disruption

GTTTAACGACTCGAGAGGCGATATTTTCTACGCGTATAGAAGGAACCCGGGCAGAAATAGACGAAGTCCTTGAGTTCGAAGCAGAAGAAAAAGACAAAAG

>B488_02780|316826..316727|Liberibacter_crescens_BT-1_EZ-Tn5_Transposon_Disruption

GCTCAGCAACACCCATTGAACGCGCAAAAATTATAGCATTCGAAGGTACAGGACTATTAGAAGAATGATTATCATCTCTTATCAAATGACGTAATAAGCG

>B488_07740|853422..853521|Liberibacter_crescens_BT-1_EZ-Tn5_Transposon_Disruption

CATCAGGAGTCACCTGGTTGATATTGCCAATCGCTGAACTGCTGATCATGGAACCGATATTGCCTGACATCTGCATGGATGCCATGTGACGCGGATCAGG

>B488_03230|371414..371315|Liberibacter_crescens_BT-1_EZ-Tn5_Transposon_Disruption

TTACGGACGTACAAAATATATTGTTGAGCAAATAATACATGATCATAATGATTATTGCGGGCTAAAATCAGTCATTTTAAGATATTTTAATGCTGCAGGA

>B488_11700|1277141..1277042|Liberibacter_crescens_BT-1_EZ-Tn5_Transposon_Disruption

GTCCTTCCTGTTTCTAGACGACATTTTACTAAGGATGCTATAATATTTGATTGTCTTTTATAGTTATAACGGTTTATAACTTGATAATGTGTAATAGCTT

>B488_13300|1443932..1444031|Liberibacter_crescens_BT-1_EZ-Tn5_Transposon_Disruption

GTCCTAGAGCATGTACTCCAGAATCTGTACGACCTGCTCCATATACCGTCACAGTTTCTTTTGTTAATGCAAAAAGTGCATCTTCAATAACACCTTGAAC

>Intergenic_Region|433180..433268|Liberibacter_crescens_BT-1_EZ-Tn5_Transposon_Disruption

GCCCCATTTTCAGGCTCCACTCCTCTTAGCGGCGCTACGGACACAGGTTTGGGGACTTCTGTTCCGCAGCAAAAATCCGAGGGGACTCCAGATCAGGAAC

>B488_00130|17271..17370|Liberibacter_crescens_BT-1_EZ-Tn5_Transposon_Disruption

GAGTATGGTGCATGGATTGATTTAATAAGATTACATATCCCAGAACATCAAAAAATTTATACTTGGTGGAGCTATCGAGCAATAAATTGGGAAGATGCTG

>B488_03860|449874..449775|Liberibacter_crescens_BT-1_EZ-Tn5_Transposon_Disruption

TTTCGTTCCTGATCTTGATGTCTCTCAACGATTAACGAGATATTTCAGAGACTTAGATTTTACGGTTAAAGAACATCCCTTCATGAATTTATATGATGAA

>B488_13120|1423885..1423786|Liberibacter_crescens_BT-1_EZ-Tn5_Transposon_Disruption

GGACAGCACTGCCCCGATTACTGCTTCTATCTGGCGGAAACGCTGTTGATCCTTGATCATCAGCAACAAACCTGCGAACTGCAATGCAGCTTGTTCAGCC

>Intergenic_Region|329143..329044|Liberibacter_crescens_BT-1_EZ-Tn5_Transposon_Disruption

CTAGTACTCCTCGGTATTCGGAGTTTGGTTAGGAGCAGTAAGGCGGTGAGCCCCCATAGCCTATCCAGTGCTCTACCCCCGAGGGTATTCAGTTAACGCT

>B488_03860|449866..449965|Liberibacter_crescens_BT-1_EZ-Tn5_Transposon_Disruption

GGAACGAAACGAACTCTTGCCCCTTTCAGCACTCTATGATGTTTTTCTCTCCATTCCCAAACAGGAGGTTTACCAAAATGAGATCCAAGGAGATCAATGC

>B488_06930|770833..770734|Liberibacter_crescens_BT-1_EZ-Tn5_Transposon_Disruption

CAATAAGGCTGGCAAGGAATTTTCTGATACTCAACTTGAATCTGTTGTATCTCATCTTGGACAAAAAGAAGTAAAGGATTTGATAGCTTCTGTTGGTAGA

>B488_01760|213135..213036|Liberibacter_crescens_BT-1_EZ-Tn5_Transposon_Disruption

GTATTTCCATACCTTTACTTATATATTCCGGTCACCGTATATACTACACAAATTTAGCTTTCCTTATACTTACTGGCTATAAATCGCATGAAGAGATTGA

>B488_12770|1391294..1391350|Liberibacter_crescens_BT-1_EZ-Tn5_Transposon_Disruption

GCTCAATATCGCTATCTTGCTTCCTCTCACCATTAACATCTAACCATATTGATCCTTCAACTCAGCAAAAGTTCAATTTTCGGGATTAATAAGAAAAAAG

>B488_01950|233506..233407|Liberibacter_crescens_BT-1_EZ-Tn5_Transposon_Disruption

ACGCTATAGGCACCGACTGCAATAATCATACAAAGGATAAACCAAATCACAGGCGAAAAAAATACAACATTAAAAAGCCCGAATATCGATAGGGAGAGTA

>Intergenic_Region|433172..433271|Liberibacter_crescens_BT-1_EZ-Tn5_Transposon_Disruption

GCCTTATGTTCAGCCTCAAACTCCTCCTAGCGGCGCTACGGACACAGGTTTGGGGACTTCTGTTCCGCAGCAAAAATCTGAGGGGACTCCTGTTCAGCAG

>B488_13520|1472522..1472621|Liberibacter_crescens_BT-1_EZ-Tn5_Transposon_Disruption

AACTTTTACTTTGTGGAGCTGATAAGGTATCCATAAATTCAGCTGCAGTAAATAATCCAGATTTTATCTTAGAAGCTACAGAAAAATTTGGTAGTCAATG

>B488_13030|1415221..1415320|Liberibacter_crescens_BT-1_EZ-Tn5_Transposon_Disruption

CCTATAAAAGGGGAACCATCTCCTTTCTATAATACAAGGGGTGTTATGAGCCATCGGCTTGGATCCTTGGTCAAGGATACCTACCAATGGTTTGTAAGTC

>Intergenic_Region|433172..433271|Liberibacter_crescens_BT-1_EZ-Tn5_Transposon_Disruption

GCCTTATGTTCAGCCTCAAACTCCTCCTAGCGGCGCTACGGACACAGGTTTGGGGACTTCTGTTCCGCAGCAAAAATCTGAGGGGACTCCTGTTCAGCAG

>Intergenic_Region|541905..541806|Liberibacter_crescens_BT-1_EZ-Tn5_Transposon_Disruption

GCATAAGACTGTGCGGAATAGTGTTTGGCTTAGGAAGTCTTGCTACTCGGCTAATAAAATCTGATCTTAAATTTGCCATGAAATGACTACCATAAATAGA

>B488_10270|1123788..1123887|Liberibacter_crescens_BT-1_EZ-Tn5_Transposon_Disruption

ATCACGCACCTTTAAAAGTAACTGTTTTTCTCTCATAGCAAACTACCAACATTTTGGTGATCATTTTTTAAATCATCATCATTATCATCATCATCATCTC

>B488_12570|1371479..1371380|Liberibacter_crescens_BT-1_EZ-Tn5_Transposon_Disruption

ATCTTGGGGTTCTCAGAAGTTATAGAAACAGAGGCAATGGGACCAATTAATCATCCAATTTATAAAGAATATATTGGTGATATTCATCGTTCTGGACAGC

>B488_01020|121673..121772|Liberibacter_crescens_BT-1_EZ-Tn5_Transposon_Disruption

GAACAGATGTTATTCATTGAAATAAAAGGAATTTTTTTATTTTCTTCTGGTTCGGGAGCATTAAGGAAAGAAAAACTGCCAAAAATAAATCCATGCCCAT

>B488_13640|1489786..1489885|Liberibacter_crescens_BT-1_EZ-Tn5_Transposon_Disruption

GTGCTGCAGATAGATTGATCATTTGTGGTTACGGTAGCACAAAAAACAATGAAGGAACATGGAGTGATATTGTTACAAAAGCTTTTACTGGGGATGAAAG

>B488_08280|903516..903586|Liberibacter_crescens_BT-1_EZ-Tn5_Transposon_Disruption

GCCTATGACTGTCATATTCGTACGATCAATGGTACGCGCTGCTTCTGGAATAACACCTTGTCGACCGACCA

>B488_10780|1175755..1175655|Liberibacter_crescens_BT-1_EZ-Tn5_Transposon_Disruption

GTTTTGAGGCTGGCTTTTGTATTGTAATCTTGGCGATTATTCTTGATCGTATGCTTAGTATTGTGGGCGTATTAGATCTGTATGACTGATTATGCAGTCA

>B488_02720|309996..309897|Liberibacter_crescens_BT-1_EZ-Tn5_Transposon_Disruption

ATTACGTACTCCTCTGTTAACAAACACTGTTACTAATGGCTTCTTTCCCCAAAATTGCTTTATATTAGACCGCAAAGCAGAAGATATCGCCCGTCGCAAC

>Intergenic_Region|329319..329399|Liberibacter_crescens_BT-1_EZ-Tn5_Transposon_Disruption

AAATAAGGGTCTTTGCGCCGAAATGTAACGGGGCTAAAGCCATGCACCGAAGCTGAGAATTTGTTTTTAATACAAGGGGTGTTATGAGCCAAAATGGCCG

>Intergenic_Region|329327..329228|Liberibacter_crescens_BT-1_EZ-Tn5_Transposon_Disruption

CCCTTATTTAGACCAGTGAGCTGTTACGCTTTCTTTAAATGATGGCTGCTTCTAAGCCAACATCCTGGTTGTTTTGGGACCCTCACATCCTTTCCCACTT

>Intergenic_Region|1163324..1163225|Liberibacter_crescens_BT-1_EZ-Tn5_Transposon_Disruption

ATTATAAGCTGTTTAGAGATTTAGACTCTTGACTTCAAATGTATGATAATACCATTTTATAATTAGGTCAGGAGTACAATTAAGGAGAACAAGACAATTC

>B488_08880|966333..966432|Liberibacter_crescens_BT-1_EZ-Tn5_Transposon_Disruption

GTATATAGAAGTGACCTTTTTCACCAACATTATCTCCCCAGGAATTTCGGATTTTAAACAATTGCGTCTCATCATCATAGCCTGTGACTACAACTGCATG

>B488_02910|337846..337748|Liberibacter_crescens_BT-1_EZ-Tn5_Transposon_Disruption

TGTAAAGAGTCCCCTGATGAGCCTCCTCCTAACAAGCCAATCTTTCTTGGCCTTTCAGAAGAACCTTCTATACCAAAAAGTGCAATTTCCATAAACTCAG

>B488_12570|1371461..1371558|Liberibacter_crescens_BT-1_EZ-Tn5_Transposon_Disruption

CTTCTGAGAACCCCAAGATAGCATTAAGTGGCGTTCGAAGTTCGTGCGACATTGAAGCAAGGAATCGTGACTTCTGCCAAATTCTGCCTCCTCAGCACGT

>Intergenic_Region|329319..329418|Liberibacter_crescens_BT-1_EZ-Tn5_Transposon_Disruption

AAATAAGGGTCTTTGCGCCGAAAATGCAACGGGGCTAAAGCCATGCACCGAAGCTGAGAATTTGTTTTAAAGACAAGTGGTAGCGGAGCGTTCCGTAAGC

>Intergenic_Region|329327..329228|Liberibacter_crescens_BT-1_EZ-Tn5_Transposon_Disruption

CCCTTATTTATACCAGTGAGCTGTTACGCTTTCTTTAAATGATGGCTGCTTCTAAGCCAACATCCTGGTTGTTTTGGGACCCTCACATCCTTTCCCACTT

>B488_07060|786840..786741|Liberibacter_crescens_BT-1_EZ-Tn5_Transposon_Disruption

GTCATAAGCAACAGCGAGTGCCGGGATCTTTTCGTGAACGATTGCCAACGCTTCTTGTTCTGCCGGGGTCAGGTTGTTTGGGCGTTTCAATGTCATCCAG

>B488_03090|355668..355569|Liberibacter_crescens_BT-1_EZ-Tn5_Transposon_Disruption

CTTCCTCTCCTTGAAGTTCTTAGTCACCGAATTTTATGGTCATTACCCATACTCTTTATCGTTATCGTATTCACACAATCCATTAATGAACTAAAAGCTT

>B488_01970|235581..235680|Liberibacter_crescens_BT-1_EZ-Tn5_Transposon_Disruption

GCTAAAGCCATTGCGCAAGGACTTTTATCTAAAGCGTTTGAAGTTCATTTTCCTAAACACTTCACCATGCTTGTTAAACTTTTGCGTTTTTTCCCTTACC

>B488_07060|786840..786741|Liberibacter_crescens_BT-1_EZ-Tn5_Transposon_Disruption

GTCATAAGCAACAGCGAGTGCCGGGATCTTTTCGTGAACGATTGCCAACGCTTCTTGTTCTGCCGGGGTCAGGTTGTTTGGGCGTTTCAATGTCATCCAG

>B488_11610|1263009..1262911|Liberibacter_crescens_BT-1_EZ-Tn5_Transposon_Disruption

TATCAGGTCCTTTTTCTCATCCGGATGTTTTGGCTAATTTTACAAGCAATGAGATAATTCTTTGCAGATCATAGATTTTCTGAACTGAAAGCAAATCTTA

>B488_01790|220291..220390|Liberibacter_crescens_BT-1_EZ-Tn5_Transposon_Disruption

GTATGGCGTTTGGCGGAATGGGTCAAGGAGATATTGGTTCTTTACAGAGATCAGGTGCATTATTCCGTCCATTTTTTTATACTCTTCCAGAGAATGCAAA

>B488_13290|1443081..1443180|Liberibacter_crescens_BT-1_EZ-Tn5_Transposon_Disruption

GGATTCGTCCCTGAACCATCTTCTCCTCCTAATTCCTTAGGAGTAGAAAGATACACATCTAAATGACCATCATCGCTTATACCTCGACCATCACGACCAC

>B488_10040|1099665..1099764|Liberibacter_crescens_BT-1_EZ-Tn5_Transposon_Disruption

CCATCAGCAGGGTCAACATACTGGCAAGTTTTATATTCTGGTTACAAGCATTATTTATTTTTAAAGAAAACCATAAATTATACTTCAAAAACTTTTATAT

>B488_13380|1455363..1455264|Liberibacter_crescens_BT-1_EZ-Tn5_Transposon_Disruption

ATTGAAGCCTTAGAGATGGAATTAAAGCTGAGATCAGCAAAAGAAAGTCTTATCAAAGCTAAAGCAAACGCTAATAATGCTTTTAATATGGCTCTTAGAA

>B488_07730|851884..851983|Liberibacter_crescens_BT-1_EZ-Tn5_Transposon_Disruption

GACAAGAGTATTCTCATGAACTCACTTAAAGGGGCCGTATCATAGGTTCCCTGTGTTGTTGTATAAACAATCCGATTTTTGGAAGAAGCGAGGCTGATCA

>B488_12700|1385969..1385870|Liberibacter_crescens_BT-1_EZ-Tn5_Transposon_Disruption

TTGATATAGAGATCAAGTTTATTCAATTTTTCTGCATCAACTCGATTTCTTATTCCATCGAACAAGACAATAATTTTATTTTTTTTCTGAAATAGAGTAA

>B488_06150|678806..678707|Liberibacter_crescens_BT-1_EZ-Tn5_Transposon_Disruption

GGAATACATGTAGCAAATGATGATACTTGGTCTGATATTTTTTCTCGCGTCCTCGTTGAAAAAATTGAACCAAATCTTGGTATAAAATGTTGTAGCATTC

>B488_09900|1080991..1081090|Liberibacter_crescens_BT-1_EZ-Tn5_Transposon_Disruption

CTTCTTGGTTATTGAATGATGATGATGATGCCTTAATCTCTTATATTCAGGAGAGAATGCATTTTACTAGTTATTCTAATAACACTAGTCGCTCACCGAG

>Intergenic_Region|618251..618293|Liberibacter_crescens_BT-1_EZ-Tn5_Transposon_Disruption

ATGTAGTAGGAGTGATCATTGCCCGAAGCCGAACTCGAAAAACGATCCCGATCTCCCTCTCGTCCTCCGCCTAAAGCTTCCACTGACGTTAGAGTCCCTC

>B488_04720|528657..528558|Liberibacter_crescens_BT-1_EZ-Tn5_Transposon_Disruption

GTTCTTAGGTATCTCAAGAATTATTCTCTTTACTTCCTCGCTGATAAACTGAAGCTTGACTGGCTTATGAGATATTTTCTCAACAATCCCTCTATCACCA

>B488_01000|116614..116713|Liberibacter_crescens_BT-1_EZ-Tn5_Transposon_Disruption

ATATACTTCTAAAGAAGTTACCGCTTATACTATTAACATTTTAAAAGATGGTATCTCTTTAGCTGTTGATATACTTGGAGATATGTTAAGTAATTCTTTA

>B488_11470|1243413..1243512|Liberibacter_crescens_BT-1_EZ-Tn5_Transposon_Disruption

GGTTATACAGGTCGTGCTATTGCTCAGCAAGCAATGCAAAATGGCATAAAAACATCAGTAACCTCACGATCAATTTTAAAGATAGAAGAGCTCCGATATA

>B488_01870|227412..227511|Liberibacter_crescens_BT-1_EZ-Tn5_Transposon_Disruption

TTTTAAGATTGCTCCAACCAGTTCCTGGTGTGGTCGTGTTATCAATGCATTAGGTGAAGCTATTGATGGTAGACCTGCTTTGGAAAAAGGAAGGTCAATT

>Intergenic_Region|779004..778905|Liberibacter_crescens_BT-1_EZ-Tn5_Transposon_Disruption

AAATTATATCCTCAAAAAATACTACAATAAATTAATCTTCATTGGTAATACTATGAAATCATATAGGTTGAAACCGAAACGAGATACAACGCAACGCGTT

>B488_07020|778996..779095|Liberibacter_crescens_BT-1_EZ-Tn5_Transposon_Disruption

ATATAATTTAATGTATATATATGCTTAAATTTTTATCTAATACCAAGAAACTTATGTGTTTGTACACTAAGACGCCATTTCGGATGAGATAAACAATAAT

>B488_05790|635229..635130|Liberibacter_crescens_BT-1_EZ-Tn5_Transposon_Disruption

CTGTAACACCCTGGGGCGTTAAGGTTACACGAGTAGAAATCAAGGATATCAGTCCACCCAATGATCTGATTGCAGCCATGGCGCGTCAAATGAAAGCAGA

>B488_03370|388553..388454|Liberibacter_crescens_BT-1_EZ-Tn5_Transposon_Disruption

GTTTTGTTCACGTTTATTAAGCTCACGTGCTGAAGTAATCTGATTATCAAGATTAGATATTAATTCCTCAAGGCTCGTCGCACGTTCTGCTAATTTTTGA

>B488_08140|884213..884312|Liberibacter_crescens_BT-1_EZ-Tn5_Transposon_Disruption

GCCTATGGGATTTCAACCGTAGCTTATGGTGCAGTTGGGTTATTGATACAGGGACTTTTCGTTTCTTTTTTGCAGGAGATACCGGTTATACCCTGCGGCT

>B488_01010|118419..118518|Liberibacter_crescens_BT-1_EZ-Tn5_Transposon_Disruption

CTGCTATTCTGTACCATTTAGCGGCTATGGCAGGATCAGCAGAAACAGTATTTCTTCCTTCGAGATAATGTGTAGCAATTTCAAAAAAAGCGACAGGTTC

>B488_09530|1024928..1024829|Liberibacter_crescens_BT-1_EZ-Tn5_Transposon_Disruption

CACCAAAGCTGATAGCGATGGTATGTATACCAAAAGTGGTGGTATCAAAAATATGGTAAAGTTTGTTAATAACCAGTTGCAAGCCATGACTGCTGCTGCT

>Intergenic_Region|329143..329044|Liberibacter_crescens_BT-1_EZ-Tn5_Transposon_Disruption

CTAGTACTCCTCGGTATTCGGAGTTTGGTTAGGAGCAGTAAGGCGGTGAGCCCCCATAGCCTATCCAGTGCTCTACCCCCGAGGGTATTCAGTTAACGCT

>B488_05690|619538..619637|Liberibacter_crescens_BT-1_EZ-Tn5_Transposon_Disruption

CGTTATGATCAGAAAAATTCTCCAGAAATTTCTTTTGGTGGGCATGCAGAGAGTATAAAAACTGCAGCCATAAAACAGTTCTGGCCATTCTGGGTTACTC

>Intergenic_Region|779714..779813|Liberibacter_crescens_BT-1_EZ-Tn5_Transposon_Disruption

GGATATGATATTATTTCTTTACCTCACCACTCCTCATCATAATCAATAGCATAGTATTTAAGTCCTTTTTAAATTATTTCGTCGCTCTTTTCTAGCAACT

>B488_01440|178831..178732|Liberibacter_crescens_BT-1_EZ-Tn5_Transposon_Disruption

CTACAGAATTTGTAAATACCAAGAAAAAATCAGTGACTTCCATACGCATGGTTTCTGGATTAAAAACTGAACCAACAGGATGCTGCATCCAACCGTTAGC

>B488_06520|721660..721561|Liberibacter_crescens_BT-1_EZ-Tn5_Transposon_Disruption

CATATACAGGATAGAAATATCTCGTAGCTGATCTAAAAATAGAATCATTCTTTTCTCTTCTTTTTTATGACTCCTATCAATCTCATTAACTCATAAAGAT

>Intergenic_Region|606000..605902|Liberibacter_crescens_BT-1_EZ-Tn5_Transposon_Disruption

GAACAAGACCTCTTGTTACCGTATCTTATTTTAACGAATTAGACGACACAGTGCACAAGCAAAATCCTTTAGCCACCGATTTAAATATTATTTAGAATAA

>B488_05150|556754..556655|Liberibacter_crescens_BT-1_EZ-Tn5_Transposon_Disruption

CCGCCTGACACTGCAATTCATCCCATGGATAATGATAAATAGCCTGTAAAATAAAACGGTTCAGCCAGCGGCGGTCATCGGATGCCCCCGATAATTCAAT

>B488_09970|1091572..1091671|Liberibacter_crescens_BT-1_EZ-Tn5_Transposon_Disruption

CCTTATCGTAGAAATAAAACCCTGTCACAGCAAGATTTGTTTTTGGACAATCAGGTTTTTCTTCTATTATAATGGCTTTACCAGAAGCATCCAATTCAAC

>B488_09490|1021066..1021162|Liberibacter_crescens_BT-1_EZ-Tn5_Transposon_Disruption

GTCCTATATTAAAGAATTCACTCTTCATTGGCTGCACCTCGCAGCATGGAAAAAAACTCATTCCCAAATTCACTATCCGCCTCTCACTATCAAGAACCGT

>B488_05150|556754..556655|Liberibacter_crescens_BT-1_EZ-Tn5_Transposon_Disruption

CCGCCTGACACTGCAATTCATCCCATGGATAATGATAAATAGCCTGTAAAATAAAACGGTTCAGCCAGCGGCGGTCATCGGATGCCCCCGATAATTCAAT

>B488_02290|264609..264708|Liberibacter_crescens_BT-1_EZ-Tn5_Transposon_Disruption

GAATGATATAGATCGTCGCTATAAAATTTCTACGCCTACATTAGTTATTGATAATATGACATTGGCAGGTCAGTAATTCGTAGTCTCTTGTCTCGAGGAA

>B488_09900|1080991..1081090|Liberibacter_crescens_BT-1_EZ-Tn5_Transposon_Disruption

CTTCTTGGTTATTGAATGATGATGATGATGCCTTAATCTCTTATATTCAGGAGAGAATGCATTTTACTAGTTATTCTAATAACACTAGTCGCTCACCGAG

>B488_03860|451041..450942|Liberibacter_crescens_BT-1_EZ-Tn5_Transposon_Disruption

GTATATAACAAAGCACAACATGGGTTTTCCCGGGGCTTGTAATTTAGGAATGAGCATACACCCTAATCGTGATGTCGTTCTTCTTAATTCTGATACTGAA

>B488_09450|1016837..1016936|Liberibacter_crescens_BT-1_EZ-Tn5_Transposon_Disruption

ACTAAATCATTATTGCCTCTTGTAATTGTTGATATTCCAACAATTTGAGAAATCTGTTTTAATATTGAATCGCGCTTATCCAAAAGATCACTATTATCTT

>B488_07890|866285..866384|Liberibacter_crescens_BT-1_EZ-Tn5_Transposon_Disruption

GTTTTACGATCGTTGACTCGACATGAACAATGCCTTGTTCTTCTTTTCTTTGTATGGTCTTCAATGTTCCTTATCATGTTCTTTTTTTCCTTGTTGGTTC

>B488_03240|372562..372463|Liberibacter_crescens_BT-1_EZ-Tn5_Transposon_Disruption

ATACTAACCCTTGATCGCTCATCATTACGTACAATACGTCGTTCCATACAAATGGTATTCCAAAATCCACTTGCATCTCTTAATCCGTCTATGTCAATAG

>Intergenic_Region|329218..329318|Liberibacter_crescens_BT-1_EZ-Tn5_Transposon_Disruption

AGTCATGGCTAAGTGGGAAAGGATGTGAGGGCCCAAAACAACCAGGATGTTGGCTTCGAGGCAGCCATCATTCCAAGAGAGCGTAACAGCTCACCGGTCT

>Intergenic_Region|329226..329127|Liberibacter_crescens_BT-1_EZ-Tn5_Transposon_Disruption

GCCATGACTTGGGGACCTTAGCTGGAGATCAGGGTTGTTTCCCTTTCCACGACGGACGTTAGCACCCGCCGTGTGTCTGCCAACTAGTACTCCTCGGTAT

>B488_05410|584235..584136|Liberibacter_crescens_BT-1_EZ-Tn5_Transposon_Disruption

ATCAGATCTTTCTATAAAACATTATTGTATAGAAAAAGATGACCAATATGATAACGAATATTCGGTATGTACTCAACCATTTAACATATTAACTCTGCAA

>B488_09490|1021066..1021165|Liberibacter_crescens_BT-1_EZ-Tn5_Transposon_Disruption

GTCCTATATTAAAGAATTCACTCTTCATTGGCTGCACCTGCAGCATGGAAAAAAACTCATTTTCAAATTCACTATCTGTTTCTTCACTATCAAGAACTGT

>B488_07960|872661..872760|Liberibacter_crescens_BT-1_EZ-Tn5_Transposon_Disruption

ATCATGAATGGCGCAAAACTGGAAGGCTGGCTTGATCGCCTGATTCAGTCCTCCGGCTATAAAGGACCGGTTGTCGAGGCAACAAAAGGAATTTCATTGC

>Intergenic_Region|329915..329816|Liberibacter_crescens_BT-1_EZ-Tn5_Transposon_Disruption

CTATACATCGTCTTAATGACTTAGCAGAGCCCTGTGTTTTTGGTAAACAGTCGCTACCCCCTGGTCTGTGACACCTTCTCATGGTTGCCCAAAAAAAGGT

>Intergenic_Region|329907..330006|Liberibacter_crescens_BT-1_EZ-Tn5_Transposon_Disruption

GATGTATAGGGCCTGACGCCTGCCCGGTGCTGGAAGGTTAAGAGGAGGAGTGCAAGCTCTGAATTGAAGCCCCAGTAAACGGCGGCCGTAACTATAACGG

>B488_07890|866285..866385|Liberibacter_crescens_BT-1_EZ-Tn5_Transposon_Disruption

GTTTTACGATCGTTGACTCGACATGAGAATGCCTTGTTCTTCTTTTCTTTGTATGGTCTTCAATGTTCCTTATCATGTTCTTTTTTTCCTTGTTGGTTCA

>B488_03730|429314..429413|Liberibacter_crescens_BT-1_EZ-Tn5_Transposon_Disruption

TGATAATCCAGTCGCTTTAAAGAAACTGAATGATTACGTTTCCTTATATCATATTAATAAAGGTTATAAGAAAGGGTATCGTCATGATGCTTTTGATGGC

>B488_06830|759844..759746|Liberibacter_crescens_BT-1_EZ-Tn5_Transposon_Disruption

CTTGTGAATAAGATCACCCTTGACACGTACGGGAATCCCAACTTGTTTTTTAAATTTTTGACGAACCAAGAGTAAAGCAATCTGATTGGCGAGTAATTCA

>B488_10250|1122551..1122650|Liberibacter_crescens_BT-1_EZ-Tn5_Transposon_Disruption

GGTTAGCCATAAAGTGATCAGACGGAATGATTATCTCCACTTCTCGCTTTAAACCTTCTGCAACGCTCTCAATAACTTGCATTTTAACCCCTTCAGTAGC

>B488_11610|1262506..1262605|Liberibacter_crescens_BT-1_EZ-Tn5_Transposon_Disruption

GTATAGAAAACTTGTTAAAATCTAAAATTTTTAAATCAATTTTAGGATTTTTTATGCTGAATCTATTACTAACGGCTCCTACACCGGAAGCTTTAACTGT

>B488_11600|1259384..1259483|Liberibacter_crescens_BT-1_EZ-Tn5_Transposon_Disruption

ATCTACTTTATTCCAAACCTCAAGAATACGACCACAATCTTCCATAACATCAATATCAAGATCTGATAGTATAGTATATACATCACGTGCTTGAGCTGCA

>Intergenic_Region|1155805..1155904|Liberibacter_crescens_BT-1_EZ-Tn5_Transposon_Disruption

CACTAAAATAACTGATTTTTAATATAATATTAATTGATCAGGAAGATTCCTTAGAGAAAGGATGTTCTAGAATCCTATTAATAATGATCAATATTTTTGC

>B488_13510|1471702..1471603|Liberibacter_crescens_BT-1_EZ-Tn5_Transposon_Disruption

GTATTGGAGTTTACACTTTTCCCACAGAATGCCCCATTTAAATCAATTAAATGGAGCCATTCAAAGCCTGAGGTTTCAAAAATTTTTGCTTGTATTACTG

>B488_06390|700220..700121|Liberibacter_crescens_BT-1_EZ-Tn5_Transposon_Disruption

GCATTAATAATCTCGCCATTGTATTTATCTGCCAATTGTAAAGCAAATCCTGACTTGCCGCTTGCTGTTGGTCCCGTTATTAATATAATATTAATATTTT

>B488_10250|1122551..1122650|Liberibacter_crescens_BT-1_EZ-Tn5_Transposon_Disruption

GGTTAGCCATAAAGTGATCAGACGGAATGATTATCTCCACTTCTCGCTTTAAACCTTCTGCAACGCTCTCAATAACTTGCATTTTAACCCCTTCAGTAGC

>B488_03730|429314..429413|Liberibacter_crescens_BT-1_EZ-Tn5_Transposon_Disruption

TGATAATCCAGTCGCTTTAAAGAAACTGAATGATTACGTTTCCTTATATCATATTAATAAAGGTTATAAGAAAGGGTATCGTCATGATGCTTTTGATGGC

>B488_06450|713820..713721|Liberibacter_crescens_BT-1_EZ-Tn5_Transposon_Disruption

ACCTTGGAGTGTAACTGCAAAAAAGAAAACAGGTTTATCTGTGCTGTGATCAGCTTCATTCATAATGACCTTGATAAAGGCTTCATCAGATGCGAATACA

>B488_13130|1425447..1425546|Liberibacter_crescens_BT-1_EZ-Tn5_Transposon_Disruption

CATCTGGGTCAAGATATTCCCATGTGCGGTGTCCCGGTACATACTGCTGATGATTATCTACAAAAACTTATTACATTGGGTTATCGTGTTGCTGTATGCG

>B488_12300|1345976..1345877|Liberibacter_crescens_BT-1_EZ-Tn5_Transposon_Disruption

GAGTGCACCTGCGTTTTGGTAGAATAGCTCGAGGAGGATTACGGTGGTCAGATCGAGCAGAAGATTATCGTACTGAAGCGTTAGGTCTGGTAAAGGCACA

>B488_13420|1459132..1459231|Liberibacter_crescens_BT-1_EZ-Tn5_Transposon_Disruption

GAGCATGGTACAGGCAATGACCCTGAAAATACAGCTTTTATTAACGCTGCATTTGTACTTTATTCGCTCGGATGTGGAAAGGTGTCAGTGCCTTATCTTG

>B488_12270|1340209..1340308|Liberibacter_crescens_BT-1_EZ-Tn5_Transposon_Disruption

TTCTTCATCTCATGATCTCTTGGGAATAAAAGTTAACACTGCACCATTATTGCAATAGCGTAAACCTGTAGGCTGGGGCCCATCCTCAAATACATGACCT

>Intergenic_Region|394391..394292|Liberibacter_crescens_BT-1_EZ-Tn5_Transposon_Disruption

GTGCAGCTCGGTGGCGGATATACATCGGAGGGATCTCTTTGACGGCTAATACTTTTATTATGAATCGATGCTTTCGTGTTTTGTCAAAAGGCCATGGCCA

>B488_12300|1344918..1345017|Liberibacter_crescens_BT-1_EZ-Tn5_Transposon_Disruption

GTCCACCATTTAATGCATAAACAATACGACCTTGTTGCGTAACTCCAAGATTAGCTCCTTCACCAATAACTTTTGCTTGCACTTGATCTGCACTAACACG

>B488_05690|619538..619637|Liberibacter_crescens_BT-1_EZ-Tn5_Transposon_Disruption

CGTTATGATCATAAAAATTCTCCAGAAATTTCTTTTGGTGGGCATGCAGAGAGTATAAAAACTGCAGCCATAAAACAGTTCTGGCCATTCTGGGTTACTC

>Intergenic_Region|329143..329044|Liberibacter_crescens_BT-1_EZ-Tn5_Transposon_Disruption

CTAGTACTCCTCGGTATTCGGAGTTTGGTTAGGAGCAGTAAGGCGGTGAGCCCCCATAGCCTATCCAGTGCTCTACCCCCGAGGGTATTCAGTTAACGCT

>Intergenic_Region|331004..331103|Liberibacter_crescens_BT-1_EZ-Tn5_Transposon_Disruption

TTATAGACCTGGTGGCTTTTGCGGAGTGACTGCACCCGTTCCCATTCCGAACACGGCCGTGAAACGCTCCAGCGCCTATGGTACTTCGTCTTAAGACGCG

>Intergenic_Region|331004..331103|Liberibacter_crescens_BT-1_EZ-Tn5_Transposon_Disruption

TTATAGACCTGGTGGCTTTTGCGGAGTGACTGCACCCGTTCCCATTCCGAACACGGCCGTGAAACGCTCCAGCGCCTATGGTACTTCGTCTTAAGACGCG

>B488_13490|1470306..1470406|Liberibacter_crescens_BT-1_EZ-Tn5_Transposon_Disruption

GTACTAGGACAAGCAATTTCTAAGCTTTAGGTAATAAAAAAGAAATAACTCTATTTGCATCTATTGATCTTGTCATGGATGAGACAATGATAACATCTGC

>Intergenic_Region|328845..328944|Liberibacter_crescens_BT-1_EZ-Tn5_Transposon_Disruption

AGTCTGAATAGGGCGCTATAGTTTGTTGCATTAGACCCGAAACCGAGTGATCTAGCCATGAGCAGGTTGAAGGTTGGATAACACCAATTGGAGGACCGAA

>Intergenic_Region|328853..328754|Liberibacter_crescens_BT-1_EZ-Tn5_Transposon_Disruption

ATTCAGACTCGCTTTCGCTGCGCCTACACCTAACGGCTTAAGCTCGCTTGTCACACTAAGTCGTTGACCCATTATACAAAAGGTACGCCGTCAGCATTGC

>B488_11420|1239640..1239739|Liberibacter_crescens_BT-1_EZ-Tn5_Transposon_Disruption

GCTCAAGCAGATGCAGGACAATTACAAGAAGCAATTACAACCATTGACCGAGCTCAGACTCCTGATAGACCTAACTGGGAATTAATGTCTGCAAAAGGTT

>B488_01930|232835..232934|Liberibacter_crescens_BT-1_EZ-Tn5_Transposon_Disruption

ATGTCGTGCAGTGGGAGCTTCAGCATGAAGCGTAGTTTTCTTATTTACTTAAGTGCCTTTGCGTTTTTACGCACATAACGCTTTTGTCGCTGAGATCGTT

>B488_11600|1259384..1259483|Liberibacter_crescens_BT-1_EZ-Tn5_Transposon_Disruption

ATCTACTTTATTCCAAACCTCAAGAATACGACCACAATCTTCCATAACATCAATATCAAGATCTGATAGTATAGTATATACATCACGTGCTTGAGCTGCA

>B488_08240|897222..897123|Liberibacter_crescens_BT-1_EZ-Tn5_Transposon_Disruption

GGTGTTAGTCTTCTGGTTTCTATTCCTGGAGCATTAGGCTATATTTATGCAGGTTGGGGAGTATCCGGTTTACCTGTTATGTCTCTTGGCTTTATTAATT

>Intergenic_Region|329143..329044|Liberibacter_crescens_BT-1_EZ-Tn5_Transposon_Disruption

CTAGTACTCCTCGGTATTCGGAGTTTGGTTAGGAGCAGTAAGGCGGTGAGCCCCCATAGCCTATCCAGTGCTCTACCCCCGAGGGTATTCAGTTAACGCT

>Intergenic_Region|779722..779623|Liberibacter_crescens_BT-1_EZ-Tn5_Transposon_Disruption

ATCATATCCTGATCTTATGCGCTATAATTTTAAAACATAATCAATATCAATGGATATAAGAGATGAAATCTTATTCTGTAAAAGAAATTTTTTTGACCCT

>B488_05790|635229..635130|Liberibacter_crescens_BT-1_EZ-Tn5_Transposon_Disruption

CTGTAACACCCTGGGGCGTTAAGGTTACACGAGTAGAAATCAAGGATATCAGTCCACCCAATGATCTGATTGCAGCCATGGCGCGTCAAATGAAAGCAGA

>B488_11320|1230330..1230429|Liberibacter_crescens_BT-1_EZ-Tn5_Transposon_Disruption

AATGAGAAAATACATAATTAATACCAGAAATATTGGTTATTATATCCTTCTAAGGTCTACACTCTAATAAATATTCTTCTAAAAAATTCTCTATTAAACA

>B488_11610|1262506..1262605|Liberibacter_crescens_BT-1_EZ-Tn5_Transposon_Disruption

GTATAGAAAACTTGTTAAAATCTAAAATTTTTAAATCAATTTTAGGATTTTTTATGCTGAATCTATTACTAACAACTCCTACACCAGAAGCTTTAACTGT

>Intergenic_Region|250081..250180|Liberibacter_crescens_BT-1_EZ-Tn5_Transposon_Disruption

GACTGGACTTGAACCAGCGACCTTTTGACCCCCAGTCAAGTGTACTACTAGACTGTGCTATGCCCCGATTTTAATTATACATAAAGATATAGAAGCAACC

>B488_04480|501020..500921|Liberibacter_crescens_BT-1_EZ-Tn5_Transposon_Disruption

ATACGTGTTCCAGCCAAAATACTCTGATACCCATCACGTCGAAGACAAGTAACATGCAATAAAACATCTTTTGAACCATTATCTGGTACAAAAAAACCAT

>B488_02200|254856..254757|Liberibacter_crescens_BT-1_EZ-Tn5_Transposon_Disruption

ACAAATACCTACATGCTTTCAGGGGATAAACATCCTCAAGAAATCATTGCTTCTGTAAAAAAAGGAATTTATGCTGTTACATTTGGTGGAGGGCAAGTAG

>B488_02780|317434..317335|Liberibacter_crescens_BT-1_EZ-Tn5_Transposon_Disruption

CAACAATACAGACCTATCAAGAGTTAATCGAATAGCTCTCCAGCCAAGAGCCGGATTACTTTCTTTTCTTAAGTGGAAATAAGGAACAACCTTATCATCT

>B488_12250|1338146..1338245|Liberibacter_crescens_BT-1_EZ-Tn5_Transposon_Disruption

GCACCAGCTGCTTCGGAAGTGACATGCTCCGAACCAACCGTCCACGCACCATAGAGCGCCCAATGATTAGAAGGAATGAATTTTGGCGCTGTGTAAATTT

>B488_12570|1371880..1371978|Liberibacter_crescens_BT-1_EZ-Tn5_Transposon_Disruption

GTCCAAGAACCTGGCTCAATAAGAGACAAAAACATCCAACAAAACCCAATAGCCACTTCGACCAATGAGAAGTCGTATACGCCATATCCCAACTTTTTCT

>Intergenic_Region|250081..250180|Liberibacter_crescens_BT-1_EZ-Tn5_Transposon_Disruption

GACTGGACTTGAACCAGCGACCTTTTGACCCCCAGTCAAGTGTACTACTAGACTGTGCTATGCCCCGATTTTAATTATACATAAAGATATAGAAGCAACC

>B488_05550|594716..594633|Liberibacter_crescens_BT-1_EZ-Tn5_Transposon_Disruption

CCATTATACAGATCAAGTCGACCGGGGAGACGATTGTGGCCAGAGACATTAAAGATCAAAACCTGCAAATCCGTTTTAATAATCGGGGCGCCATGATATC

>B488_05410|584235..584136|Liberibacter_crescens_BT-1_EZ-Tn5_Transposon_Disruption

ATCAGATCTTTCTATAAAACATTATTGTATAGAAAAAGATGACCAATATGATAACGAATATTCGGTATGTACTCAACCATTTAACATATTAACTCTGCAA

>B488_01920|232843..232744|Liberibacter_crescens_BT-1_EZ-Tn5_Transposon_Disruption

GCACGACATCAACCCGCCCATATTTGAAAGCAGCGATACAATAGCTCAGATAAAAACGCCACATATAGATAAATTTTTCATCAAAACCTATAGACTTGAT

>B488_08260|900354..900453|Liberibacter_crescens_BT-1_EZ-Tn5_Transposon_Disruption

CATGACGATATCCTCTGATGTTATTCACTGTCTGTATAACATGAAACACATAAGCAGGAGTAACAGATATGAATAATCTCATTCCGCATCATTTTAAAAT

>B488_11970|1306335..1306236|Liberibacter_crescens_BT-1_EZ-Tn5_Transposon_Disruption

GCACTAACATGTGGTGAATATATTAATCCAAATAGCTCCAGATCACTCATGATATCACGTACAGAGGCTGGTGAGAGCGATACAGATAATAGTCGTGATA

>B488_11840|1293848..1293947|Liberibacter_crescens_BT-1_EZ-Tn5_Transposon_Disruption

ACGTAATCCACCTCCAATTATTATTTTTTCAGATATTCAACAAAATAGCTTTTTATTCGAATTACGTGTTAATGTAGCCAACATACTTTCTGGAACAAAA

>B488_00090|11633..11732|Liberibacter_crescens_BT-1_EZ-Tn5_Transposon_Disruption

GTTGAATATGTACAAGATTTTTCTGATTCTAAGAAGCCAGCTCAATACCCAATGCATGAAACTGCAAATAGTGGTTTAGCGCAATCATTGTTTGAGCGGA

>B488_06860|763069..763168|Liberibacter_crescens_BT-1_EZ-Tn5_Transposon_Disruption

CCACAAGATATAAGAAATTTTAAAGCATCTTCAACGCTCATATCAAGTATAGTTATTTTATTTCGTGGGATAAATAATAACATACCAGCAGTTGGTAATG

>B488_04900|539518..539419|Liberibacter_crescens_BT-1_EZ-Tn5_Transposon_Disruption

CCTTACAAGAGAGGCCAAAACAGAAGGAAGAGGAACAACTCCCTTAAGAATTTCCCTGTTGCTGGAATACATCCCAAAGACAAGATTATCCGTAACTTTT

>Intergenic_Region|418388..418293|Liberibacter_crescens_BT-1_EZ-Tn5_Transposon_Disruption

GGTTACAGACTTACCCATTATAGATCCTTGCCTATATTATTCAACCTTGAAACATCTGAAGAAGAAGCTGCTATACACAGAAACCCTTATAAACTTGTTC

>B488_11420|1239707..1239608|Liberibacter_crescens_BT-1_EZ-Tn5_Transposon_Disruption

CTATCAGGAGTCTGAGCTCGGTCAATGGTTGTAATTGCTTCTTCTAATTGTCCTGCATCTGCTTGAGCTTTCCCATAAGCAGCAAGAACACTTCTATCTT

>B488_08800|958706..958805|Liberibacter_crescens_BT-1_EZ-Tn5_Transposon_Disruption

TTTATGTGCTGAGCAAATTTTCTAAACATAATCCCTAAAGGATGACGAACATCTTGAAGAGAAGGCAAAAGAAAAGAATCTATGACAGGTTGCCAATCTA

>B488_06330|694750..694849|Liberibacter_crescens_BT-1_EZ-Tn5_Transposon_Disruption

CTTCAACATACATGCAAAGAAAAAAAATAGCACGTAAAGAATACTTTTCTTCTAAGGAATATGGTGTGAAGCCAAGTGAGCGTGTATATTATGGACGAAA

>B488_06570|726480..726381|Liberibacter_crescens_BT-1_EZ-Tn5_Transposon_Disruption

CGACAGTATAGCCTATTTTCGTTTGGTCTATCAGGCCAATGACAATCTGGCTTTTGAACGTATCGTCAATACTCCAAAACGCGGTATTGGCAATACTACT

>B488_00030|4665..4764|Liberibacter_crescens_BT-1_EZ-Tn5_Transposon_Disruption

AGTCAAGCTTATATAAAATTAGCTTCTGAAATGATTGAACGAGAAAGAGAACGTAAAGCTGCTTAACTTGAAAGAAAAAAAATGAATGAGAATCATTCTA

>B488_01680|202224..202165|Liberibacter_crescens_BT-1_EZ-Tn5_Transposon_Disruption

ATTATAGACCCACTAGATTCTGAAATCAATTTAAGCGCTTTTTTTAATTTGATCCTTCAACTCAGCAAAAGTTCCACGTTCATCTTTTTAAAAATGATTT

>B488_11420|1239707..1239608|Liberibacter_crescens_BT-1_EZ-Tn5_Transposon_Disruption

CTATCAGGAGTCTGAGCTCGGTCAATGGTTGTAATTGCTTCTTCTAATTGTCCTGCATCTGCTTGAGCTTTCCCATAAGCAGCAAGAACACTTCTATCTT

>B488_11700|1276996..1276897|Liberibacter_crescens_BT-1_EZ-Tn5_Transposon_Disruption

GATTAGACCTGCAACGCCCTAAGGGTGCGTCAATACTTCCAGATATTGTAGAAAGGTTACCCCAAACAATAGCATAATATGAGCGCTCTAGTCCCATGGT

>B488_09640|1041209..1041308|Liberibacter_crescens_BT-1_EZ-Tn5_Transposon_Disruption

GCGCTACACTCTCAGATAAATCTTCATTAAGTTTCAAGGCACGTGCCAGCGATCTAGCAATTTGAGATACTTCAACAGTGTGAACAAGACGAGTACGATA

>Intergenic_Region|963517..963418|Liberibacter_crescens_BT-1_EZ-Tn5_Transposon_Disruption

GATAAGAAGAGGTCATTTATTTCTCCACTAGTGCAACTTTTTTATTAAACCACTTCATGAATAAAGATATCCTAACCCAAAGAGATGAATAAACAGATAT

>B488_10750|1173192..1173291|Liberibacter_crescens_BT-1_EZ-Tn5_Transposon_Disruption

CCTTTTGAGCATAAACTAATGTTTATAGACTATATTTTTAAAACTTTATCAATATTTAATCGATAGACATTAAGAAGAACAATCTGAGCAAATAATTTCC

>B488_03700|424080..423981|Liberibacter_crescens_BT-1_EZ-Tn5_Transposon_Disruption

CTATCAAAGATACTCCTTGAGTCTCAAGAAAAAGATCTTAAAAAGATGCAAACCCTTTTTACAAAGAATCTAACAAGCGAGACAAATTTACATCAACAAG

>B488_12580|1373328..1373229|Liberibacter_crescens_BT-1_EZ-Tn5_Transposon_Disruption

GTCTATAAGGGTTTAGGTGAGCTATACTTTTGTTTTTCAGGAAGTACATATTTTAGTGGCAATTCAGTAGTAAATTTTATTTTTTCTATAGCAAAAAGTG

>B488_07200|803095..803006|Liberibacter_crescens_BT-1_EZ-Tn5_Transposon_Disruption

TCTATATTGTTTTCAAGACTTATTGTTTCCTGCATTAGCGTAGGAGGAAGTGATCCCCCGGAACGTCCGAGCAGCAGACCACCGATACGACTCCCCCCAC

>B488_13420|1458947..1459046|Liberibacter_crescens_BT-1_EZ-Tn5_Transposon_Disruption

CGATATGCCTAGCTAATTATTACTCTGGAACGGCGCCGCTTATAAGTCTCCACGGCCAATATATCTGCAACAAGCGCAAGCGTCTTGAGGCGTTGGCCGA

>Intergenic_Region|330912..331011|Liberibacter_crescens_BT-1_EZ-Tn5_Transposon_Disruption

GTGTGGAAGTTGGGTAACCAGTGAAGCTTACCAGTACTAATAGCTCGATTGGCTTGATCGTTCTCATTGACTATTATCATCTTTTTTGCCCTTTATAGAC

>B488_07370|821143..821242|Liberibacter_crescens_BT-1_EZ-Tn5_Transposon_Disruption

CATTCGTACAGATTGCATTTCTTGAATCAATTTCAACACCTGCACGTTCAAGACCCATATTTCCGGTATTAGGTTTGCGACCCGTTGCAAGTAATACCTG

>B488_09270|1000645..1000744|Liberibacter_crescens_BT-1_EZ-Tn5_Transposon_Disruption

CTGTAAAGCGAACTTGATCAAGATACATTGCCTTCACTGTTCCAGCTGTACCAACAGGCATAAAAGCAGGTGTTTTAATAATGCCTCGTGGCATAGAAAT

>B488_09580|1031697..1031796|Liberibacter_crescens_BT-1_EZ-Tn5_Transposon_Disruption

GCTTAATAGTCATTGAAGCAGCAAAAAGATCAGGCTCTTTAATCACTGCAAGGCTAGCAGGAGAATTTGGAAGAATTGTGTTTGCTGTCCCTGGATCACC

>B488_04660|523926..524020|Liberibacter_crescens_BT-1_EZ-Tn5_Transposon_Disruption

GATAAGCAAGGCGTGTCTTGTCTGCTTCTTTTGTATATGTTTCAGCCATAGCTATTTTTGACCACCCGAACATGGCCATAAGTTCCTGTGTGCTGATCCT

>B488_03700|424072..424171|Liberibacter_crescens_BT-1_EZ-Tn5_Transposon_Disruption

CTTTGATAGAGACTCTTGAGAGTGCATATTCCTGTAGTCAGAAAATATATTGCGCATTTGTACTATTTTTAAATTTGTTAATTCAAATAAAGAAAGCATG

>B488_00010|1718..1817|Liberibacter_crescens_BT-1_EZ-Tn5_Transposon_Disruption

CACTTGCTAATCTTGAAGTTATTGAAAAAGAAGCTTTTGAGCTTCTTATAGAAGATGGAAAAATTTCTGCAGTGATCACAAAAGATGGTCATAAAATATA

>B488_13100|1421692..1421593|Liberibacter_crescens_BT-1_EZ-Tn5_Transposon_Disruption

CCGCTAATCAATCCTGCCCGACCGCCGTTGGCGCTGATTGGCGTCTACAGCCCGAAACTCGTGCTGCCAATTGCCGAGACGATGTGCGTGTTGGGCTATC

>B488_03300|382546..382447|Liberibacter_crescens_BT-1_EZ-Tn5_Transposon_Disruption

CCTTTATCCTTATTGCTTGTATCGATAGATTTTTCTTTAGCTAACTCTTCAAACTTCTTTCCAGAAAGAAGACTTTTAATAATTGCCTTTGCTTCGTCCT

>B488_06810|756291..756390|Liberibacter_crescens_BT-1_EZ-Tn5_Transposon_Disruption

CCCCTGTCATAGCCAATTGTAATGTCCGGGGAATTGGTGTGGCTGAAAGAGTTAATATATGAACATCATTTTTTAGCTCTTTAAGTCGTTCTTTATGTTT

>Intergenic_Region|1323124..1323025|Liberibacter_crescens_BT-1_EZ-Tn5_Transposon_Disruption

CTATTATATAAAACTATCGAATAAGTTTCTTTAATGAGACATATTCTCCTTGACTGACGTTTTCAAATCCTCCCTGTCGTCGGTCAAGAGTTAAGCCCGA

>B488_05000|545260..545359|Liberibacter_crescens_BT-1_EZ-Tn5_Transposon_Disruption

CAATTTATCTCTCCTGGATCTTTCAGGGTCATAGCTCTAGGTGTTTGATATTAAGATATACCTTATCCCTGGTTTGTATTTTGAAAATAAATATTTCGTT

>Intergenic_Region|329145..329046|Liberibacter_crescens_BT-1_EZ-Tn5_Transposon_Disruption

AACTAGTACTCCTCGGTATTCGGAGTTTGGTTAGGAGCAGTAAGGCGGTGAGCCCCCATAGCCTATCCAGTGCTCTACCCCCGAGGGTATTCAGTTAACG

>B488_11840|1292923..1293022|Liberibacter_crescens_BT-1_EZ-Tn5_Transposon_Disruption

TAGCTACTCTTTCAGGATACATAGGTCTAGCACATTTTGTATCAAAACAAATTGTTATCACCAGCTCAGTCTTAATAATAATGTATATTGGTGTTCTTGC

>Intergenic_Region|433661..433562|Liberibacter_crescens_BT-1_EZ-Tn5_Transposon_Disruption

GGTTAATACATGTCATGCAGCATGCAGACCATCATGAGCATGCAGACCATCATGAGCATGCAGATCATCATGAGTATGCGGTACACTCTCATCTTCTTTG

>B488_03950|458043..458142|Liberibacter_crescens_BT-1_EZ-Tn5_Transposon_Disruption

GTTATAGGCTGTAACAGATAATGGGTTATCATTTATTCTTTCCAATACGAGATAATCACCTTGAGAAGCTTCACCAGCATTGTAAATGCGGACGCTGTTA

>Intergenic_Region|330920..330821|Liberibacter_crescens_BT-1_EZ-Tn5_Transposon_Disruption

CTTCCACACCCAGCCTATCAACGTGGTAGTCTTCCACGGCTCTGATAGGGAATACTCGTTTTCAGGTTGGTTTCCCGCTTAGATGCTTTCAGCGGTTATC

>Intergenic_Region|330912..331011|Liberibacter_crescens_BT-1_EZ-Tn5_Transposon_Disruption

GTGTGGAAGTTGGGTAACCAGTGAAGCTTACCAGTACTAATAGCTCGATTGGCTTGATCGTTCTCATTGACTATTATCATCTTTTTTGCCCTTTATAGAC

>Intergenic_Region|329145..329046|Liberibacter_crescens_BT-1_EZ-Tn5_Transposon_Disruption

AACTAGTACTCCTCGGTATTCGGAGTTTGGTTAGGAGCAGTAAGGCGGTGAGCCCCCATAGCCTATCCAGTGCTCTACCCCCGAGGGTATTCAGTTAACG

>B488_07370|821151..821052|Liberibacter_crescens_BT-1_EZ-Tn5_Transposon_Disruption

GTACGAATGAGTATTTTCAGACAAATATACAGTCAATATTTGCAATTGGTGATGTTATTAATCGTGTACAGCTTACACCTGTTGCTATTCATGAAGCAAT

>Intergenic_Region|1323124..1323025|Liberibacter_crescens_BT-1_EZ-Tn5_Transposon_Disruption

CTATTATATAAAACTATCGAATAAGTTTCTTTAATGAGACATATTCTCCTTGACTGACGTTTTCAAATCCTCCCTGTCGTCGGTCAAGAGTTAAGCCCGA

>B488_09580|1031697..1031796|Liberibacter_crescens_BT-1_EZ-Tn5_Transposon_Disruption

GCTTAATAGTCATTGAAGCAGCAAAAAGATCAGGCTCTTTAATCACTGCAAGGCTAGCAGGAGAATTTGGAAGAATTGTGTTTGCTGTCCCTGGATCACC

>B488_07730|851295..851196|Liberibacter_crescens_BT-1_EZ-Tn5_Transposon_Disruption

TGCCAAGGCAGTCCAGGAACTCAGTGCTGAGGTCAAAACATTGCGAAAGCATCTTTCGGAACAGGAAGAGAAGAACAGTCTCCAAAAGGAATGAAACGAT

>Intergenic_Region|329143..329045|Liberibacter_crescens_BT-1_EZ-Tn5_Transposon_Disruption

CTAGTACTTCTCGGTATTGTTGAGTTTGGTTAGGAGCAGTAAGGCGGTGAGCCCCCATAGCCGATCCTGTGCTCTACCCCCGAGGGTATTCAGTTAACGC

>B488_10800|1178533..1178434|Liberibacter_crescens_BT-1_EZ-Tn5_Transposon_Disruption

GTATATGATCAACAGGTGTATAAGACGAGAATATATTTCAATTTGACGTCGTCCTGACACTTTTACTGAGCCCGGTTTACAAACACGACTCTTATCATTT

>B488_13680|1493725..1493626|Liberibacter_crescens_BT-1_EZ-Tn5_Transposon_Disruption

CAATAGTGTGCTCTGAAGTACGATTATTAATTGAAATCATGAGTGATCACCTTTTTTTAAAAAATATAAATTTTTATAAGAAAAAACTCTTTATTTAGAA

>B488_03980|461667..461763|Liberibacter_crescens_BT-1_EZ-Tn5_Transposon_Disruption

ACCATACACGTTGTGTACGTAAAGGTGCGCCTCATGCTCCCATCCTCACAGATCTGCATCCCTGACTTCTGCTACACCGGAACAGGCTCCGTATAAACGC

>B488_09230|995587..995488|Liberibacter_crescens_BT-1_EZ-Tn5_Transposon_Disruption

AATAGGAGGAGATGATACAAATACAACAGCATCAGATCTTGTTGATTATTTGTCTAAGCGGAGCTATAAATTAACAGTTGTTGGTGTGCCAAAGACAATA

>B488_03980|461667..461763|Liberibacter_crescens_BT-1_EZ-Tn5_Transposon_Disruption

ACCATACACGTTGTGTACGTAAAGGTGCGCCTAAAGCTTTTCTTCTCACAGATATGCCATTCCTGACTTATGCTACACCGGAACAGGCTTGTATAAACGC

>B488_03950|458141..458240|Liberibacter_crescens_BT-1_EZ-Tn5_Transposon_Disruption

TATATAAGGAGCTCGAGAAGGTTTCTTGTTGTTGATGTGCTGAACCGGAGAATATTGCTTTTATCTTGTATTTTTGGATGATATTTTGCATATTTTGTTT

>B488_01140|135249..135151|Liberibacter_crescens_BT-1_EZ-Tn5_Transposon_Disruption

CTATAAGAATTTTTTGTAATAAAGTGTTAAACACAATCTAATCTAAACTCTCATTCGGCATTAATACATAAAATACATCATTATTATCATTGTTCTTAAC

>Intergenic_Region|326541..326442|Liberibacter_crescens_BT-1_EZ-Tn5_Transposon_Disruption

GTTAGCCGGGGCTTCTTCTCCGGATACCGTCATTATCTTCTCCGGTGAAAGAGCTTTACAACCCTAGGGCCTTCTTCACTCACGCGGCATGGCTGGATCA

>B488_13090|1420266..1420365|Liberibacter_crescens_BT-1_EZ-Tn5_Transposon_Disruption

CTTCTACCGGCGCATCCGCAAACACGCCAACATAGAGCAGCGCAGCGCCGGCCATCACGGTGCGGGCGGTGTCGATATCCACACACCGCGGGGAGCCGGG

>B488_10610|1156014..1156112|Liberibacter_crescens_BT-1_EZ-Tn5_Transposon_Disruption

AATTTGTACTTGATTAATATTTTCCACACCTTCAGCAATGCAATCCAATCCCATATCCTTACATAACAAGCTAAGGAATTTTACTATTTTATAGCTATTA

>B488_07710|850051..850149|Liberibacter_crescens_BT-1_EZ-Tn5_Transposon_Disruption

ATATCGGAATGGGCCAGAACATGGGTGGGTGTAATATACGGATAACGCTGAATAATACTTCTTGCCAGCTGGATCACGGCCTTCTCGCTGTTCCAGGGGA

>B488_13070|1418418..1418517|Liberibacter_crescens_BT-1_EZ-Tn5_Transposon_Disruption

GAGGAAAACGCACGCAGATTGGCGTTCTGGATGGTGGGGCCGTCGGCCAGCGGGTCGGAGAAAGGGATGCCCAGCTCCAGTGCATCGGCGCCGGCGTCAA

>B488_13130|1426839..1426739|Liberibacter_crescens_BT-1_EZ-Tn5_Transposon_Disruption

GTTCAAGAGTAGTAAACCGCATGACTTAGCCATTGTTTGACGGTGGATGAATCTGGCTTTAGCTTCTTTCGTAGCTGTTAAAGTTTCAGAAAGATTTGAT

>B488_06780|752534..752633|Liberibacter_crescens_BT-1_EZ-Tn5_Transposon_Disruption

TTCCAGAATGGTGCTCAGCCAGTCGCTGGGTAAATACATGTCTGAAATGAAGGCTACTTGTTCAGCCATGCCCTTCATTTCATCGAAAACCTTGAGGCCT

>B488_05160|557260..557177|Liberibacter_crescens_BT-1_EZ-Tn5_Transposon_Disruption

GCATAATCCTGCGTATTGCCCGTGTCCAGCTTCAGCGAGCCTGCCCCCTGACTGGCAACATTGCCCAAGGTCTGCCGCGTTTGCTTTCATATCTGGATGT

>B488_04360|490190..490286|Liberibacter_crescens_BT-1_EZ-Tn5_Transposon_Disruption

CATAGCTTGTTTTTTTGGATAAAGTAATGGGAAGGTATCCATAAATAAAGGAAAGTTGATTTTCAGCTCAGTGAAGCACTCGTCTGATACTATTGTCATT

>B488_03940|456765..456860|Liberibacter_crescens_BT-1_EZ-Tn5_Transposon_Disruption

CTATTATCATAACCCCAAGCGGTATTTACTTGGACAAGCCATATTCCGGTGTCAAACGGCGATCAGAATCAACATCATGGAGCAAGCACGACTAAAGCAC

>B488_06780|752377..752278|Liberibacter_crescens_BT-1_EZ-Tn5_Transposon_Disruption

GATATATCCCAAGCCAGCAACCAATCTATAAGCACACGATTTGCAGATTGGGCCAAAGTTGACAATCGGCGAATCCCAACGACTGCAACGCATACGGAAT

>B488_11840|1292923..1293022|Liberibacter_crescens_BT-1_EZ-Tn5_Transposon_Disruption

TAGCTACTCTTTCAGGATACATAGGTCTAGCACATTTTGTATCAAAACAAATTGTTATCACCAGCTCAGTCTTAATAATAATGTATATTGGTGTTCTTGC

>Intergenic_Region|326541..326442|Liberibacter_crescens_BT-1_EZ-Tn5_Transposon_Disruption

GTTAGCCGGGGCTTCTTCTCCGGATACCGTCATTATCTTCTCCGGTGAAAGAGCTTTACAACCCTAGGGCCTTCTTCACTCACGCGGCATGGCTGGATCA

>B488_06790|753082..753173|Liberibacter_crescens_BT-1_EZ-Tn5_Transposon_Disruption

GTCTAATAGTTCTTGAATTTCACTGAGAACAGTCATATTAAATGCAAACGGCCGTCCACTCCGACAGCTACACCGAAAAGAGCGTCTCGAACAAACTGAG

>B488_06990|777118..777019|Liberibacter_crescens_BT-1_EZ-Tn5_Transposon_Disruption

CAATAGCCATTCGTATAGCTCAAGAAAATGCTGTTTTAAACCATGTGAGTTTAAAAACAAGTTTTGATGTATCTACAAATTTTGAAGCAAGAACTTTTCA

>Intergenic_Region|329143..329044|Liberibacter_crescens_BT-1_EZ-Tn5_Transposon_Disruption

CTAGTACTCCTCGGTATTCGGAGTTTGGTTAGGAGCAGTAAGGCGGTGAGCCCCCATAGCCTATCCAGTGCTCTACCCCCGAGGGTATTCAGTTAACGCT

>Intergenic_Region|329143..329044|Liberibacter_crescens_BT-1_EZ-Tn5_Transposon_Disruption

CTAGTACTCCTCGGTATTCGGAGTTTGGTTAGGAGCAGTAAGGCGGTGAGCCCCCATAGCCTATCCAGTGCTCTACCCCCGAGGGTATTCAGTTAACGCT

>B488_13280|1441676..1441775|Liberibacter_crescens_BT-1_EZ-Tn5_Transposon_Disruption

CTATTAAATGATGAAACCTTCTAGTCGTATTCTCGGATTTGGTCATTCTGTCCCTGAAAAATGTATTAACAATTCCGATATCGAATTACAATTAGGACTA

>B488_07030|781661..781760|Liberibacter_crescens_BT-1_EZ-Tn5_Transposon_Disruption

CCTATGGCTGGGCAGAAACTTTCGCCAGGGGTGCTATCAAGTCTGACAAGGCCAATACACTCAAAGTTAAAGCTAACAAGACCTTGATCACTGCGCTGAT

>B488_07030|781668..781570|Liberibacter_crescens_BT-1_EZ-Tn5_Transposon_Disruption

GCCATAGGTCTGCCGTTTGTCCGAGCAGAGGCTTGAGGGCTTCCTGCCAGAATTCTTTATGCTCGTCAGAGAGCTTAACCCAAGCGGCTTCAGATTCAAG

>Intergenic_Region|329143..329044|Liberibacter_crescens_BT-1_EZ-Tn5_Transposon_Disruption

CTAGTACTCCTCGGTATTCGGAGTTTGGTTAGGAGCAGTAAGGCGGTGAGCCCCCATAGCCTATCCAGTGCTCTACCCCCGAGGGTATTCAGTTAACGCT

>B488_05170|558618..558519|Liberibacter_crescens_BT-1_EZ-Tn5_Transposon_Disruption

CCTTTAAACCTCTGCTCATAGTTTATAATGTTGGCTGCTTCCTCAGGAGATTGCGCATTCGCAAGCCCTTTTATCGTATCGGGGTAGGATTTCATAAAAT

>B488_10970|1196236..1196137|Liberibacter_crescens_BT-1_EZ-Tn5_Transposon_Disruption

CTTTTTATTCATGGACGCATAAGACAAACAGGTGTATCCCCATCTTTTGATGAAATGAAAGATGCGTTGAGACTTGTGTCAAAAGCAAGTATTCATCGCT

>B488_01270|155260..155161|Liberibacter_crescens_BT-1_EZ-Tn5_Transposon_Disruption

ATCTTAGACTGATCAAACTTTCCAACACGGAATTTAGGTCCTTGAAGATCTACTATGATACCAATCGGGTATCCGTGCCGTGATTCTACTGATCGAATTT

>B488_04680|525213..525313|Liberibacter_crescens_BT-1_EZ-Tn5_Transposon_Disruption

GACGAAGCATTTCCTTCAAGATTGGGAACAATGCGCATTTCCTTTCGCANGGCATCANGGAAGATCAGCTTCCAGTCCTCGGCAGCAAGCCTTGTCCCAT

>B488_07940|871461..871362|Liberibacter_crescens_BT-1_EZ-Tn5_Transposon_Disruption

TCACAGGGTTTACCCCAGGCGATAGATTCACAGGCCATGAGCAGGCAATTGGTGAAATGCTTCCTGATCTGTTCAAAATCATGCAGGACACATATGACGG

>B488_12800|1395050..1395149|Liberibacter_crescens_BT-1_EZ-Tn5_Transposon_Disruption

CCATACGAGAAATACTCTCTGGTATGCTTAAACCAATCTGTTGAGAATCAACAATACGACGGAATTCATTTCTGAGAGGCTGTTTCGATTGCGTGGCAAT

>B488_09000|686692..686593|Liberibacter_crescens_BT-1_EZ-Tn5_Transposon_Disruption

GTTCTAGGTGGTGTTAGCGCAGCAAGATATAAAGACCCAGTCACAAGTACGTCTAATGAGCCAGAGAAATTAAATATCACCAGCCCGGCTATTGGTGGTT

>B488_09000|686692..686593|Liberibacter_crescens_BT-1_EZ-Tn5_Transposon_Disruption

GTTCTAGGTGGTGTTAGCGCAGCAAGATATAAAGACCCAGTCACAAGTACGTCTAATGAGCCAGAGAAATTAAATATCACCAGCCCGGCTATTGGTGGTT

>B488_09000|686684..686783|Liberibacter_crescens_BT-1_EZ-Tn5_Transposon_Disruption

ACCTAGAACACCGCCGTATGCGCCTTCCCAAGAGAAATTACCAGAATTCGAACCCGTGGGCTGTGACGCAGCATGAGGAATATTATCTACATTATTCACG

>B488_13570|1477848..1477749|Liberibacter_crescens_BT-1_EZ-Tn5_Transposon_Disruption

GGTAAATAGATCCATCTCCATTGGACAATTCTTTATACACAGGAAGATGAGTAGGCTGTAAAAGGGAGTTGAACCAGTTACACAAGGTCTGTTTCCATTC

>B488_13420|1459557..1459642|Liberibacter_crescens_BT-1_EZ-Tn5_Transposon_Disruption

CTATAACACCAGTCAAGTCTACGAAATTGTCAAAGATTATAATCTCGCAGAAAAGCAGCTTCGGCTAGCAATTTCATATGATCCTTCAACTCAGCAAAAG

>B488_03770|435282..435183|Liberibacter_crescens_BT-1_EZ-Tn5_Transposon_Disruption

GATATAGCAGCTCGTGGCCTTGATATTCATGATGTAGGACATGTTTTTAATTTTGATGTTCCAATTCATGCTGAAGATTATATTCATCGGATTGGGCGTA

>B488_03770|435282..435183|Liberibacter_crescens_BT-1_EZ-Tn5_Transposon_Disruption

GATATAGCAGCTCGTGGCCTTGATATTCATGATGTAGGACATGTTTTTAATTTTGATGTTCCAATTCATGCTGAAGATTATATTCATCGGATTGGGCGTA

>B488_04500|502604..502505|Liberibacter_crescens_BT-1_EZ-Tn5_Transposon_Disruption

AATTATAGCTATCAAAAAAAGATGGAGATAATTTCATTAATCTAGAGAAAACATCTATACGCAAATCAGATGCAACTCGTTCCCCTAACAATGATACACA

>B488_01970|235499..235598|Liberibacter_crescens_BT-1_EZ-Tn5_Transposon_Disruption

TATCAGTCCAGGCTTTGTACGAACGCGATTAACTGATCAGAACGATTTTACAATGCCAATGATTATCAAACCAGAAGATGCTGCTAAAGCCATTGCGCAA

>B488_01970|235507..235408|Liberibacter_crescens_BT-1_EZ-Tn5_Transposon_Disruption

GGACTGATAAGCTTAATATCAATTTTATCAAATAATTCCGCATAAAGACTCTCTGCTAAATTGATAATAGCTGCCTTAGTTGCACTATAAGGCTGTCCAC

>Intergenic_Region|1451408..1451507|Liberibacter_crescens_BT-1_EZ-Tn5_Transposon_Disruption

CTTTAATAAAGATAGACACAAAGATCATTCTAAACCTTATCCATTAAGCTCCTTCGAATGAGGATATAATAATATTAAAGATATTTATTTTGTTAATTCA

>B488_06720|744479..744578|Liberibacter_crescens_BT-1_EZ-Tn5_Transposon_Disruption

CGTTTAAAAAGCCTCAAATCATCTTATTTTGTAGACTTTAAATTTCAGCCAACAGGCATCCGTGTCGGCGGGAATGTTTTTCCTATTGTCAAGATGATCT

>B488_11310|1230194..1230094|Liberibacter_crescens_BT-1_EZ-Tn5_Transposon_Disruption

CGTTTCTTTGGAGCCTCAAATCCTTTTTGATGTCCAATGTGTAATTTGCTTTGATCTGTTACTTCTAAAATTTTTGGGAGGAATGCATCTGAAAGTTTCG

>B488_08390|915419..915320|Liberibacter_crescens_BT-1_EZ-Tn5_Transposon_Disruption

AGAATGTGCTGATTGGGGTCTGGAAAGTTTTCTTGAGATTAAAGCCATGATTGGTTATTTTAATACTTCCTATGAAGAAGAAAACTGATTGAATGTTTAA

>B488_03770|435938..435858|Liberibacter_crescens_BT-1_EZ-Tn5_Transposon_Disruption

TCGCTGCACAAGTCGCGGACAGTTTTGAAAAATATGGAAAAAATCATAAGCTAAATGTCGCATTACTAATNNGAGGTGTGTNNTNNGNNNNNCAAAACCG

>Intergenic_Region|179913..179814|Liberibacter_crescens_BT-1_EZ-Tn5_Transposon_Disruption

ATATAATCCGTAAGCTACCATAATCTAATAACATATGCAGAATCATTATTCTTCTCTACTTTTACTACTTTTCAAATATTAAGTGCTTTAATTCGTACTC

>B488_03320|384549..384648|Liberibacter_crescens_BT-1_EZ-Tn5_Transposon_Disruption

GTATTAGAGTGCCATACTGAATAAATACAGTACTTTTAAAAAAGAGAGTAATTTTTCGTGAATTCTAACAAACACAAACTTGTTTTAGTGGTTGCTTGTG

>B488_09600|1034631..1034532|Liberibacter_crescens_BT-1_EZ-Tn5_Transposon_Disruption

TATATTAACAGAACAAGGAGAGAGTCTTTATAAGACAGCACATGATATACTCTTGAAGTTAGAGAGAGTTAAAATAAAATTAACTGAAGCAACTAAAAAA

>B488_13250|1439117..1439037|Liberibacter_crescens_BT-1_EZ-Tn5_Transposon_Disruption

TCCTTTACCTCCGCGTTTGCGCCATTGGCTAATGGCGTCTTCAAAAGCATTGACGTTATTATGAGGTATAGGAATAGTTTT

>B488_02930|339618..339519|Liberibacter_crescens_BT-1_EZ-Tn5_Transposon_Disruption

GTATTTGCTATTACCAGTTTTACTTTTTATTCCTGGATCGGTGTATCCAAAACTTTTTTATATAGTTTTTACACAATCACACAGTTTTCTTTCGGAATGA

>Intergenic_Region|326427..326378|Liberibacter_crescens_BT-1_EZ-Tn5_Transposon_Disruption

GTCCATATTCCCCACTGCTGCCTCCCGTAGGAGTCTGGGCCGTGTCTCA

>Intergenic_Region|326419..326518|Liberibacter_crescens_BT-1_EZ-Tn5_Transposon_Disruption

ATATTGGACAATGGGGGCAACCCTGATCCAGCCATGCCGCGTGAGTGAAGAAGGCCCTAGGGTTGTAAAGCTCTTTCACCGGAGAAGATAATGACGGTAT

>B488_13160|1429898..1429985|Liberibacter_crescens_BT-1_EZ-Tn5_Transposon_Disruption

AAGCAATACATCATCTTCAAGAATAAGAGCGGCTTCATGTCCATCAGAAACAAATTTCTCTAAGGCTGCAACATGACTATAATACAAGGGGCGTTATGAG

>B488_09670|1043549..1043648|Liberibacter_crescens_BT-1_EZ-Tn5_Transposon_Disruption

CCACTATAGACACATAACGCTATCACAGAGAAAATAAGAAAAAATGACTTAAACCTCAATATACCATTAATGCATTGCATTTTTATTCTCTAACAATTAT

>B488_07750|853748..853649|Liberibacter_crescens_BT-1_EZ-Tn5_Transposon_Disruption

ATGCACAGCACAGGATGTTGTTGACCCTTGGCGGGCTGCGGTATCGCATTCCACGGTTGCGTGGCCCTTCTGCTGCTGAAAATATTTACGTGATCACCAG

>B488_05060|549525..549426|Liberibacter_crescens_BT-1_EZ-Tn5_Transposon_Disruption

CTATAGCTCATCCCGTCAAACGTCTTTTTGTTCTCTACGCTATCACCGAGAGGCAACGCGTCCACAACATCACGATCATAACCCATCTCAATCAGATCAG

>B488_05910|650204..650303|Liberibacter_crescens_BT-1_EZ-Tn5_Transposon_Disruption

TTCAAAGTCGCAGTTGTATACTGTACGGGGTAGATATTCATCCAGCTGCAAAGATTGGTAGCGGTATCATGCTTGACCATGCAACAGGGCTTGTAATCGG

>B488_01430|176917..177016|Liberibacter_crescens_BT-1_EZ-Tn5_Transposon_Disruption

ATTCTAGATACTCTTTATTTAAGAGGGTTGGTTCCGATGGTCGTGGCAATTTCTAGCTCCTTTATTTTAGGTGTAGGGTTGTTTTATTTTTCTCCATTAT

>B488_04830|535614..535515|Liberibacter_crescens_BT-1_EZ-Tn5_Transposon_Disruption

GTATTAGTCTTAGCCTCAAAATATCCCCCAGAAATAGAATTAAGGATCCTGAAAAAATGTGAAGAATTACATATAAAAAATCTAGAAAATCTTAAGAAAA

>Intergenic_Region|1236478..1236379|Liberibacter_crescens_BT-1_EZ-Tn5_Transposon_Disruption

GTATATACTAACCACTGTACCATATATTCAGGATTTTAATTTTCAAAATCAAAAACATATGAACAACATTCGTTATCTAGTGTATATTGAGATTAAAGTC

>B488_04360|490344..490245|Liberibacter_crescens_BT-1_EZ-Tn5_Transposon_Disruption

TACTTATACATTGCAAAGGAGGAGCAGATCGCACAGGATTAGCATCTGCGCTCTATTTAATGACAATAGTATCAGACAGTGCTTCACAAGCTGAAAATCA

>B488_07030|781661..781760|Liberibacter_crescens_BT-1_EZ-Tn5_Transposon_Disruption

CCTATGGCTGGGCAGAAACTTTCGCCAGGGGTGCTATCAAGTCTGACAAGGCCAATACACTCAAAGTTAAAGCTAACAAGACCTTGATCACTGCGCTGAT

>B488_01850|225715..225616|Liberibacter_crescens_BT-1_EZ-Tn5_Transposon_Disruption

ATACTATGCTATCTTGGCCATGGGAGAATCATTTCCCGCTATAGGTATCGTTGGTGCCGTTCTTGGAATTGTGAAAGCTATGGCAAACATCAACCAATCC

>B488_01970|235156..235057|Liberibacter_crescens_BT-1_EZ-Tn5_Transposon_Disruption

ACTGAATCGGTAACGTCTAGTACAAAGACTTTATGCTTCTCTCCTAGAACAGATTTCAAATTCTCTAAGTCATTGCTACTCCGAGCTGAAAGGACAAGTA

>B488_09340|1008826..1008925|Liberibacter_crescens_BT-1_EZ-Tn5_Transposon_Disruption

ACTTAGAACGGCTAGAATTATGGCGTATGGAAGCCTTTGGGAAATAATCAATCTTATCAATGAAAACACGTAATAATACATCGGTTCCAAATTGAGAATT

>B488_11970|1306523..1306423|Liberibacter_crescens_BT-1_EZ-Tn5_Transposon_Disruption

AGCCGCACCACGTGAAATACCTGAAAGTACTTTGCTTGCTTTACTCATAACTTCTTCCAGAGATTTATTAGATTCGCTATTGCCGATCTGATTCTCTACC

>B488_01270|155260..155161|Liberibacter_crescens_BT-1_EZ-Tn5_Transposon_Disruption

ATCTTAGACTGATCAAACTTTCCAACACGGAATTTAGGTCCTTGAAGATCTACTATGATACCAATCGGGTATCCGTGCCGTGATTCTACTGATCGAATTT

>B488_04960|544262..544362|Liberibacter_crescens_BT-1_EZ-Tn5_Transposon_Disruption

CTCCAACCTGAGTAAAGTAAAGCTGGCTCCCATAACAACAAAGATAATCGTTACAATCTGAATCGTGGTAGGTATGTTGGTTAGCTTTCCTTCAATGCCA

>B488_03820|441529..441430|Liberibacter_crescens_BT-1_EZ-Tn5_Transposon_Disruption

TTCTCACACAGGCTTCTTTCTTAAGACGTTACTCAAAAAAAATGAATAAAAGCTTCAAAAAGAATTAAATTTTCTTTTTACTCTGGTAAACATTGGATAG

>B488_05470|591534..591633|Liberibacter_crescens_BT-1_EZ-Tn5_Transposon_Disruption

TCGTTAAGGAGTCTATCTTTGTTCCATTCCAGTTTCAGAGAGTTTGCTTCTTTTTTGGCAGTTGAAGGTAATAACTGCATTAATCCTTGAGCACCAGCAG

>B488_13280|1441676..1441775|Liberibacter_crescens_BT-1_EZ-Tn5_Transposon_Disruption

CTATTAAATGATGAAACCTTCTAGTCGTATTCTCGGATTTGGTCATTCTGTCCCTGAAAAATGTATTAACAATTCCGATATCGAATTACAATTAGGACTA

>B488_12810|1396082..1396181|Liberibacter_crescens_BT-1_EZ-Tn5_Transposon_Disruption

CGTGTATAGTACCCATAGAACCATCATGACCTGTATTCATTGCCTGTAAAAGATCAAAAACTTCTGGGCCTCTAACTTCTCCCAAAATAATCCTCTCAGG

>B488_11620|1265670..1265769|Liberibacter_crescens_BT-1_EZ-Tn5_Transposon_Disruption

CCCCAATAACCATTACCAGAAACACCATCAACAGTTGCAAACTGCACACCTAATCCAAAATATCGATAAGGACTTTCTGATACCTCAATGATGACCGGCA

>B488_12020|1312587..1312686|Liberibacter_crescens_BT-1_EZ-Tn5_Transposon_Disruption

ATATTACAATCCTCAGAAAAGGCAAAAAGAAAAAGGTCAGGAGCAGAACATTATGTTGTTGATATGGTTATTGATCAAATACAAAGTTTAGTACCCCAAA

>B488_06540|723224..723323|Liberibacter_crescens_BT-1_EZ-Tn5_Transposon_Disruption

ATTCAATGTTATGCGGTAATATCATCAGATGATATCCCTGATAGATTTTTAAAATCATATACATCTGAAGGTTTTGTCGATAAAATTCTGTACAGCGCTG

>B488_00930|109796..109896|Liberibacter_crescens_BT-1_EZ-Tn5_Transposon_Disruption

GAGTATGGCGTTGAAATTCTTCATATATTTTTGTATTTCTCGACATACTGTTGCAAAGCGCTCCCCTCCTAATATTTCTGAACAATCTTTTAGGGTTTCT

>B488_01820|222962..223061|Liberibacter_crescens_BT-1_EZ-Tn5_Transposon_Disruption

AACTAGGTGAAGGTATCGTAATGAGATCTTCAAAAAGAAAAACTTTAGGGCGAACTTTATCAAATGCTTCTTTACTAAGAGTTTCTAATGATTTCAGTAA

>B488_10470|1140786..1140885|Liberibacter_crescens_BT-1_EZ-Tn5_Transposon_Disruption

AATCATCCTCTATAACCTTCCAAAAAGAAAGAAACAGTGCCCTCAGAAAAATTGCAAATGCTATTCCCAATAGTAAAGGTATATTCTGGATGATTACCAA

>B488_02050|240497..240456|Liberibacter_crescens_BT-1_EZ-Tn5_Transposon_Disruption

TCTTTAAACAAAGCAGCTTGAGAATCTCTCCATAAAGAATAATACAAGGGGTGTTATGAGCCATCAATCCCTTACCGCCCTGAATGTTATTCATTCGTCT

>Intergenic_Region|461333..461432|Liberibacter_crescens_BT-1_EZ-Tn5_Transposon_Disruption

GATTATTATAGCGGTGACTTTAGAATAATTTTAAAAAGGATTCTTATCTCAATTTACAGTGGATTCCCTATATAAAAAATAATAAGTTTAAACATTAATA

>B488_03950|458927..458883|Liberibacter_crescens_BT-1_EZ-Tn5_Transposon_Disruption

CTCTTACCAGACCTGCCAGAAAAATGATACGTTTATTGTCATGGCTAATATTCAGGCATGGAAACTTTCGGATGGTGAAGATAACAAAGTGCAATGGATA

>B488_11150|1215469..1215369|Liberibacter_crescens_BT-1_EZ-Tn5_Transposon_Disruption

CTATAGCAACACGCTGCTTTTCTCCTCCGGATAATTTTAATCCACGTTCACCTAATATAAGGGGTGTTACGACCCATAGACTTTCATGAATGCGTCTAAT

>B488_01210|145055..145154|Liberibacter_crescens_BT-1_EZ-Tn5_Transposon_Disruption

GTATTGGATTGCGAAATATAATCGGATGGGCTTGAATCATTGGATTGGAGTTGAAAAACCATATTCTTATATATGAAGTATATAGCTGTTATATTTTTTA

>B488_06090|673182..673254|Liberibacter_crescens_BT-1_EZ-Tn5_Transposon_Disruption

AACCAATACTGCTCCTGTTGATGCGCCAAAAGAAAGACCGAAAAGGAATGGATCTGCCAAATCATTGCCAGTAATACAAGGGGTGTTATGACCCATCATT

>B488_01680|202224..202125|Liberibacter_crescens_BT-1_EZ-Tn5_Transposon_Disruption

ATTATAGACCCACTAGATTCTGAAATCAATTTAAGCGCTTTTTTTAATTTGAGGCTTAACCCTTCCATCGTAAAAGTTTTCCACGCTGCACGACAAGATA

>B488_01760|213646..213747|Liberibacter_crescens_BT-1_EZ-Tn5_Transposon_Disruption

AATCAGTGATGGCTTCAGAAATTCGGATAATGCCAAGCCTCTAAAGCCATCAAATTTACGGTCGCGCGCTACGCCGGAAGAGCAGCAAAATCTATCGGAA

>B488_13420|1459663..1459762|Liberibacter_crescens_BT-1_EZ-Tn5_Transposon_Disruption

GATCTGGGAGACCTGCTTGCCAAAATAGAAGGCCGTGAAGCAGAAGCCCTGGAGGAGTATACTCGCGCCATTGATCTTTGCCCCCCTTACTATGAGGCTT

>B488_04360|490570..490668|Liberibacter_crescens_BT-1_EZ-Tn5_Transposon_Disruption

GGGCAGAGCGATAGAGCTCTCCTGTAATAATTTCATGAAAGTTCGTTATTATAATAATTAACTGCTACATGACTTCCTACATAAAAAGCAGGAAGAAGCA

>Intergenic_Region|330760..330661|Liberibacter_crescens_BT-1_EZ-Tn5_Transposon_Disruption

GTCCATCCCGGTCCTCTCGTACTAGGGAAAGATCCTGTCAATATTCCTGCACCCACGGCAGATAGGGACCGAACTGTCTCACGACGTTCTGAACCCAGCT

>B488_01290|159360..159459|Liberibacter_crescens_BT-1_EZ-Tn5_Transposon_Disruption

GTATTACGACGTGCGTGCCAAATACGAAATTGTCTCCCAATGGGTTTTTGCCATAAAGATGCTAATGAAAATAGCCCAATATGAGTGATTTTTTTAGGCA

>B488_05540|596843..596943|Liberibacter_crescens_BT-1_EZ-Tn5_Transposon_Disruption

GAATGATAGGTAACAGCTCTCTATCATCGTCCTCTAGATCTTTGTAGTATAGGAGACTTGTCTCCAGACATTATAGCCCAATATTGGCCCAGGAAAGAGG

>Intergenic_Region|439824..439721|Liberibacter_crescens_BT-1_EZ-Tn5_Transposon_Disruption

GTATAATGTTCTTCATTCATACCTTATAAAGGTTCTCTTTTGTGATTGATGTGATGCCCCAAAAAACAGCTATGCAAAAAAATAAAAATAATAATCATCA

>Intergenic_Region|329736..329835|Liberibacter_crescens_BT-1_EZ-Tn5_Transposon_Disruption

GGTAGAGCATACTAAGGCGCTTGAGAGAACTGCGTTGAAGGAACTCGGCAAATTGCACGCGTAACTTCGGAAGAAGCGTGACCTTTTTTTGGGCAACCAT

>B488_05820|637790..637889|Liberibacter_crescens_BT-1_EZ-Tn5_Transposon_Disruption

GGATAAGAGCAATCAGAAGTTTTGTTCATCATCGCAAAACGGGCTTTTAAAAGACATTCTTGATATTCTTGCCAGGCATTGGAATCATAGACAATGCCGC

>B488_00200|28026..27927|Liberibacter_crescens_BT-1_EZ-Tn5_Transposon_Disruption

AGTTTAAACGATCTATTGTTTGTGTTAATTCTATTTCTAATTCATTTTGAATACTAGAAAAAACTGAATATCCCAATCCATGTCTAATATTAAAAGATAT

>Intergenic_Region|418388..418289|Liberibacter_crescens_BT-1_EZ-Tn5_Transposon_Disruption

GGTTACCGACTTACCCATTATAAATCCTTGCCTATATTATTCAGCCTTGAAACATCTGAAGAAGAAGTTGCTATACCCCCAAACCCTTATAAACTTATTG

>B488_03940|457418..457319|Liberibacter_crescens_BT-1_EZ-Tn5_Transposon_Disruption

TCTCTATACACCACGTAATGTCTTAAAGCGTGTCCTAGAACTTTATAAAAAAAATTCTCTTAATCCCATCATTGCTCCAGAAATTGAGTTTTATCTTATT

>B488_05170|560064..559965|Liberibacter_crescens_BT-1_EZ-Tn5_Transposon_Disruption

CCTTTAAATCGATGTCCCTTAGACACAAACCCAATAGGTGGAGCAAGTATTGTGCTTCCTGTTCCCCCCGTCTGCGTCTGCGGTTGTGCTCCATGGGGTT

>B488_13420|1459132..1459231|Liberibacter_crescens_BT-1_EZ-Tn5_Transposon_Disruption

GAGCATGGTACAGGCAATGACCCTGAAAATACAGCTTTTATTAACGCTGCATTTGTACTTTATTCGCTCGGATGTGGAAAGGTGTCAGTGCCTTATCTTG

>Intergenic_Region|330194..330293|Liberibacter_crescens_BT-1_EZ-Tn5_Transposon_Disruption

GTGTAGGATAGGTGGTAGGCTTTGAAGCAAGGGCGCCAGCTTTTGTGGAGCCATCCTTGAAATACCACCCTTATCCACATGGATGTCTAACCGCGGTCCG

>Intergenic_Region|650939..650840|Liberibacter_crescens_BT-1_EZ-Tn5_Transposon_Disruption

CATAACCGCTGACTTGGCGGATCAATTCATAACTTTAAATTTGAGCGTAACTATATCATCCATCAATTAGACTTCTCTCGACAAAACATCTTTCTTCTGA

>B488_07050|784724..784625|Liberibacter_crescens_BT-1_EZ-Tn5_Transposon_Disruption

TACATCATTCTCATCAACGATGAATACATCATTATTGAACTCACCATCGTCCTGGTGTATCACCTGCTTGCCGGTCATCTTGCCAGAGAAGGCTATAAGT

>B488_03950|458927..458883|Liberibacter_crescens_BT-1_EZ-Tn5_Transposon_Disruption

CTCTTACCAGACCTGCCAAGAAAAATGATACGTTTATTGTCATGGCTAATATTCAGGCATGGAAACTTTCGGATGGTGAAGATAACAAAGTGCAATGGAT

>B488_03720|425366..425298|Liberibacter_crescens_BT-1_EZ-Tn5_Transposon_Disruption

TTCTTATCGATGCCACAATCCGAAGAACTTTTGCCTAATAATAAAATTACTGTAAGAAATCTGACTTTACATTACACTCATACAAGGGGTGTTATGAGCC

>B488_00200|26546..26645|Liberibacter_crescens_BT-1_EZ-Tn5_Transposon_Disruption

ACTTACTTCTTATGTAGAGCCAGTGATATGTAAAGAACAAAATGATAATGTTCATTCCGCTTTTTCCAGGATGTTTGTTACACCAAAGATTAATCAGGAA

>B488_12440|1360226..1360127|Liberibacter_crescens_BT-1_EZ-Tn5_Transposon_Disruption

GCCTGGGGATGTTACGTTTTCTCGTAGGGTTGAGATTTCATCAGTAGATTGACGTATAAGCTCGCTTGATCCTATGATTGTATCACGTGCTAACCTAACC

>B488_11490|1246874..1246775|Liberibacter_crescens_BT-1_EZ-Tn5_Transposon_Disruption

ATGTTGGTCTAGCAATTAAAGATCAGAATTTGCCAGCTGTAGGGTATGCTTTGTTAACAATGTTGTTAGTAATTATTTTTTATGATCAGTTGTTATTTCG

>B488_05550|596102..596022|Liberibacter_crescens_BT-1_EZ-Tn5_Transposon_Disruption

GTGGTGCAGACATTGCGCAATATCACACGGATGTTTTTAAACAGGTTTACGGCCCTGCTTCAAAAGCAGAAGAAGCCTGGAATATCCTTCTTCTACAAGG

>B488_11620|1264787..1264688|Liberibacter_crescens_BT-1_EZ-Tn5_Transposon_Disruption

AGTATATGCAGGTATTGGGCATGCATTTTAAGGTGTGAACAAATGGGATTTAATTTATGAAAGCACATTTTTTAACATCTGTAATCAAGATTTTTCTTTC

>Intergenic_Region|329143..329044|Liberibacter_crescens_BT-1_EZ-Tn5_Transposon_Disruption

CTAGTACTCCTCGGTATTCGGAGTTTGGTTAGGAGCAGTAAGGCGGTGAGCCCCCATAGCCTATCCAGTGCTCTACCCCCGAGGGTATTCAGTTAACGCT

>B488_02780|315883..315982|Liberibacter_crescens_BT-1_EZ-Tn5_Transposon_Disruption

GAATAGGCTTGTTAAGCAGATTGCTGAAGATATGGCATTCCACGTATGTTCGATATATGTATTGTCTCCGGATGGTCTTCTTGAGTTATATGCTACAGAA

>B488_11420|1240033..1239934|Liberibacter_crescens_BT-1_EZ-Tn5_Transposon_Disruption

AGTTTACATTGTGCTCAGCATCTTCAGGAGACAATTCCTGACGAGCAATTTTATAGGCTTCATCAATACGGCCTTGTAATCCCAAAACAAAAGCTAGATT

>B488_11620|1264769..1264865|Liberibacter_crescens_BT-1_EZ-Tn5_Transposon_Disruption

CCCATATACACTAGCATATACTGCATAACGAGATGAATTCTTGTACCTATTTAATGGAATAGCCACATCAAACCGAATAGGACCTAAAAAAGTATTATAT

>Intergenic_Region|330828..330927|Liberibacter_crescens_BT-1_EZ-Tn5_Transposon_Disruption

GCTGAAAGCATCTAAGCGGGAAACCAACCTGAAAACGAGTATTCCCTATCAGAGCCGTGGAAGACTACCACGTTGATAGGCTGGGTGTGGAAGTTGGGTA

>B488_04360|490301..490400|Liberibacter_crescens_BT-1_EZ-Tn5_Transposon_Disruption

TGCTAATCCTGTGCGATCTGCTCCTCCTTTGCAATGTATAAGTAGGGGTTTTGGAGAATTTTTCATCATTTCTATAAGTGTATTTATTTCTTTTGTTGTT

>Intergenic_Region|329018..328919|Liberibacter_crescens_BT-1_EZ-Tn5_Transposon_Disruption

CCAGCTATCTCCGAGTTTGATTGGCCTTTCACCCCTAGCCACAAGTCATCCCAATCTATTGCAACAGATACGGGTTCGGTCCTCCAATTGGTGTTATCCA

>B488_09910|1081779..1081878|Liberibacter_crescens_BT-1_EZ-Tn5_Transposon_Disruption

TATGTGTAGAGTTTTGTGCACGTCTGGGATTTGGACAAGCAACCTTAGTAGTTGCTGTTCCGGAAATATGGTTAGATGTAGATACTATGGCTGATCTTGT

>B488_11070|1206671..1206572|Liberibacter_crescens_BT-1_EZ-Tn5_Transposon_Disruption

GGAGGAGGCTCTCTAGAAAAGCCGGTAGGTTTAGCTCATGTAGTCGCTATTTCACAGTCAGGAAATTTTATTCATAGTAAAATTAATGATCTATCATTGG

>B488_11450|1241602..1241701|Liberibacter_crescens_BT-1_EZ-Tn5_Transposon_Disruption

TTCTTATGGAGTAGACCCATGCAAAAAACACTATTAAATTTAAATGCATAAATGTCATTTCATATAGATAAGGCTATTAAAGATATTACACCAAATATTA

>Intergenic_Region|326785..326686|Liberibacter_crescens_BT-1_EZ-Tn5_Transposon_Disruption

CGTCAGTATCAGACCAGTGAGCCGCCTTCGCCACTGGTGTTCCTCCGAATATCTACGAATTTCACCTCTACACTCGGAATTCCACTCACCTCTTCTGAAC

>Intergenic_Region|1157898..1157997|Liberibacter_crescens_BT-1_EZ-Tn5_Transposon_Disruption

CTTCTAAAAAATTCAGTAAAGAAAGAATATAAAAGAACATATCTCTTTATATTATAAATAAATCCTTATAAAAGAGTTCTCTAAGAGAGATAGAGCAATC

>Intergenic_Region|329622..329523|Liberibacter_crescens_BT-1_EZ-Tn5_Transposon_Disruption

CGCCAGGCCCACGAATATTAACGTGGTTCCCATCGACTACGCGTGTCCGCCTCGCCTTAGGGGCCGGCTAACCCTGCTCAGATTAACTTTAAGCAGGAAC

>B488_04730|529209..529110|Liberibacter_crescens_BT-1_EZ-Tn5_Transposon_Disruption

CTTCCAGTAAAACCAACGGCTCTACAGAAGACGGTTTTTTCTGCGCCAAAATAGCATCAGCCTCCTGTCTTATTCCAGCTGCAACAGAAGTTAAAAGAGA

>B488_13080|1418893..1418794|Liberibacter_crescens_BT-1_EZ-Tn5_Transposon_Disruption

CGCCTACCTTGATAGCATAGGCCGGGCGGAGTACGTATCGGTTACCGACGAAGAAGCCCTGGATGCCTTCCGTCGCCTTTCCCGTCATGAGGGAATTATT

>B488_03860|451167..451068|Liberibacter_crescens_BT-1_EZ-Tn5_Transposon_Disruption

CATATATACTGTACTTAAGGCACCACAAAAAACAGCTTATAACTTAATTGTTATAGATGATCACATCCCTGAAAAAAGATTACGAAATGAGATTTATCAA

>Intergenic_Region|330828..330927|Liberibacter_crescens_BT-1_EZ-Tn5_Transposon_Disruption

GCTGAAAGCATCTAAGCGGGAAACCAACCTGAAAACGAGTATTCCCTATCAGAGCCGTGGAAGACTACCACGTTGATAGGCTGGGTGTGGAAGTTGGGTA

>B488_01920|232751..232850|Liberibacter_crescens_BT-1_EZ-Tn5_Transposon_Disruption

CTATAGGTTTTGATGAAAAATTTATCTATATGTGGCGTTTTTATCTGAGCTATTGTATCGCTGCTTTCAAATATGGGCGGGTTGATGTCGTGCAGTGGGA

>B488_04830|535276..535375|Liberibacter_crescens_BT-1_EZ-Tn5_Transposon_Disruption

GTATTTATCAATATACCTCTCAAGTTTTGCATGTTTAAAGCATATCATACTTACATCTTTTTCGATATTATCTACCTTTTCAGAAACTTTTAATATAAAA

>B488_03970|460414..460315|Liberibacter_crescens_BT-1_EZ-Tn5_Transposon_Disruption

CATTAACGGTAACATTACTGACTCTGGAAAAGGAAATATAATAAATTCTTTCTATAAAGTTTATAATCAACTGAATTTTCCTTATTATATAGGATTAGGA

>Intergenic_Region|431339..431240|Liberibacter_crescens_BT-1_EZ-Tn5_Transposon_Disruption

ATATTGAAGAGCATTATCTGCATTGATAGCAGCATCTGGTTTCTCTAAAATATAGCTTAAAGCAATCTCTTCCTCTAATTTATCGTCCAATTCACTCATG

>B488_04460|499579..499481|Liberibacter_crescens_BT-1_EZ-Tn5_Transposon_Disruption

GCTTTATACCTCTAAAACAATACCCCTAAAATAGATTTGCCATTTTAAAGCATAGGTAAATATCTCATTGCCTAACATTCTGTACTTCTTTATCAATAAA

>Intergenic_Region|433171..433263|Liberibacter_crescens_BT-1_EZ-Tn5_Transposon_Disruption

CCTAAGGAGCCTTATGTTCAGCCTCAAACTCCTCCTAGCGGCGCTACGGACACAGGTTTGGGGACTTCTGTTCCGCAGCAAAAATCTGAGGGGACTCCTG

>B488_08300|907211..907112|Liberibacter_crescens_BT-1_EZ-Tn5_Transposon_Disruption

GTATATAAACTGTCAAGATCATGCGTTATCATGTAGACCGTTAATCCTAATGTATCACGTAATTTCAAAATAAGTTCATCAAACTCGGCTGCACTAATAG

>B488_08060|878950..878852|Liberibacter_crescens_BT-1_EZ-Tn5_Transposon_Disruption

TGACAAATCCTGCTTCAATGCGGTGCCGAGACTTTCACGAATGGTGACATCTCCGGCAATGACTTCTGCCTTTGTTCCCAGAAGGCTTTTAGCCTTTGTG

>Intergenic_Region|330621..330680|Liberibacter_crescens_BT-1_EZ-Tn5_Transposon_Disruption

TCCCAAGGGTTTGGCTGTTCGCCAATTAAAGCGGTACGTGAGCTGGGTTCAGAACGTCGT

>B488_05900|648960..649055|Liberibacter_crescens_BT-1_EZ-Tn5_Transposon_Disruption

ACCCTGTACCACTCCCGATATCCGAATATCAACTTCACCTTGAGTGTCATTCCAAATTTCTTCAGCTGTTGTTTGGCGATGAATTTCCGGATTGGCAAGG

>Intergenic_Region|330557..330458|Liberibacter_crescens_BT-1_EZ-Tn5_Transposon_Disruption

ACTCTTGGGGGTCATCAGCCTGTTATCCCCGGCGTACCTTTTATCCGTTGAGCGATGGCCCTTCCACACGGGACCACCGGATCTCTATGACCGACTTTCG

>B488_03660|419549..419450|Liberibacter_crescens_BT-1_EZ-Tn5_Transposon_Disruption

GACAACTACAGCATGACCTCCATTCAGTCGGTCAGAAACCGAAGGCATCGGGATAATTGTAGAAGGATTTCCTCCTTTTCCCTGGAACCAGCTGTCATAA

>B488_12250|1339237..1339138|Liberibacter_crescens_BT-1_EZ-Tn5_Transposon_Disruption

CTCTAATGTAGGACATCGATTATTCAGAAATGCTAACGACGGTTTTTTTAGCGGTATTTTAATCGGTTTGTTAATCGGGGTAGTCTGGACTCCCTGTGCA

>B488_11900|1300463..1300562|Liberibacter_crescens_BT-1_EZ-Tn5_Transposon_Disruption

CAATACAACGCCAGATTCTAAGTTGTTCATATTGCAAAAAGCAATTCATAATGTAATTTCTGAAGCAATCGAAGCTGGAGGATCATCTTTACGTGACTAT

>B488_03150|363177..363276|Liberibacter_crescens_BT-1_EZ-Tn5_Transposon_Disruption

TTGTATCAGAGAGCATAATGAGAGAAACCGGAGTTCCTTTTTTAGATTTTTGATTTAAATGATAATGAATATCTTTAATTGTGCTATCTTCAGTAATAGT

>Intergenic_Region|1410918..1411017|Liberibacter_crescens_BT-1_EZ-Tn5_Transposon_Disruption

AAGGAATACACTTTATTAGTATTTCTAAACAGGTCTAAATTTTAAAAATAAATTTTAAAAATAAATTTTTATAACGCGGGGGACACCTTTTTGTGTGTCC

>B488_05930|146246..146297|Liberibacter_crescens_BT-1_EZ-Tn5_Transposon_Disruption

GTTTAGAGAGATCTTGAATGGCTCTTCCAGTTCTTCAAAGAAAAGGCGGAGGTAAAAGAATTTTCGAGCAATACGATCATTATAGAGTTGTGCGTTTATT

>B488_07740|852571..852670|Liberibacter_crescens_BT-1_EZ-Tn5_Transposon_Disruption

TTTGATATCTGTTTTGGCACGTACATCAGAAAGTGCGGAAAAACCTGTTAAAAGTTTGCTCCCCAGGGATAACCCCTCCCTGAATTTTTTCTGCTGATCC

>B488_10000|1094117..1094216|Liberibacter_crescens_BT-1_EZ-Tn5_Transposon_Disruption

GGAATACCGTTATACTCAAAAGAGCTATTTAACTCTTTCAAACCAGGTAAAATTTTGTCTTTATCAGAAATTTTTATAGACATACCTTCCAAAAGTGGCC

>B488_05410|583705..583804|Liberibacter_crescens_BT-1_EZ-Tn5_Transposon_Disruption

TCATGGAACATATGATGCATGTCTGATGATGTATGCAGAAGATCATCCTGTTGTTCATCCTGGAGATGATACAATGGATTATCAATATCGTGTCTTTGAA

>B488_13110|1422604..1422703|Liberibacter_crescens_BT-1_EZ-Tn5_Transposon_Disruption

GTCGTGAGCAATGCCGGCATGCAGCCTGCCTGGGCCGGCACACCGGGACCTGGCGAGAGCATCAGGATCGGGTTTTCCATTTCGGACAGCACCCGCGTGA

>B488_03290|379019..379118|Liberibacter_crescens_BT-1_EZ-Tn5_Transposon_Disruption

GGGTAGGAAGGACTCTTTTACAAAGGTATCACGAAAATGATTAAGATGAATGTTTTAGGTATTTTTTTTATTTAAAGAACCATGGCAATGTTTATATTTT

>B488_01860|226407..226308|Liberibacter_crescens_BT-1_EZ-Tn5_Transposon_Disruption

CTCTAACGCTATCTGCGCAGAAAGCGAAACATTAAGAGCTGCTTGCATTATTTTCTCCCAACGCTATATAAAAATTATAATGTAATATATTAAAACTTGC

>B488_11490|1246874..1246776|Liberibacter_crescens_BT-1_EZ-Tn5_Transposon_Disruption

ATGTTGCGTCTAGCAATTAAAGATCAGAGTTTGCCAGCTGTAGGGTATGCTTTGTTAACAATGTTGTTAGTAATTATTTTTTATGATCAGTTGTTATTTC

>B488_11450|1241602..1241701|Liberibacter_crescens_BT-1_EZ-Tn5_Transposon_Disruption

TTCTTATGGAGTAGACCCATGCAAAAAACACTATTAAATTTAAATGCATAAATGTCATTTCATATAGATAAGGCTATTAAAGATATTACACCAAATATTA

>Intergenic_Region|605835..605934|Liberibacter_crescens_BT-1_EZ-Tn5_Transposon_Disruption

GTTTAATGTATAGTGAAAGTTTAAAAGTTTCTTTGGAAACGAGATCGTATCAAAACCAATCATAATTTTATTCTAAATAATATTTAAATCGGTGGCAAAG

>B488_03770|435664..435763|Liberibacter_crescens_BT-1_EZ-Tn5_Transposon_Disruption

TGATATAATTCTAACAATATTTTCAATATCAGGAATAAAGCCCATATCAAGCATTCGATCTGCTTCATCTATAACGAGTATTTTAATACCACTCATAAGC

>B488_01950|234129..234228|Liberibacter_crescens_BT-1_EZ-Tn5_Transposon_Disruption

GACCTGATTTTACCTCCATCTATCCTTTCAGATGATATTCACGCACATAAAAATAGCCAATTTTCGGCAACGCATTACGCCTTATTAGCTTTTATTTCTA

>B488_00200|28026..27927|Liberibacter_crescens_BT-1_EZ-Tn5_Transposon_Disruption

AGTTTAAACGATCTATTGTTTGTGTTAATTCTATTTCTAATTCATTTTGAATACTAGAAAAAACTGAATATCCCAATCCATGTCTAATATTAAAAGATAT

>B488_06090|673182..673281|Liberibacter_crescens_BT-1_EZ-Tn5_Transposon_Disruption

AACCAATACTGCTCCTGTTGATGCGCCAAAAGAAAGACCGAAAAGGAATGGATCTGCCAAATCATTGCCAGTAGTAGTTTGCAGCAGTGCACCAACCATT

>B488_06750|748715..748814|Liberibacter_crescens_BT-1_EZ-Tn5_Transposon_Disruption

TGTCTATACTGACTTACTTGGATCACTTTGATTTATTTACTTATCGCGTAGGTTCTGTTGAGTTTTCACAATACGTGGTCGGCGAAAGCGAATCTTCATG

>B488_05090|552619..552520|Liberibacter_crescens_BT-1_EZ-Tn5_Transposon_Disruption

CCTGTGCCACGATCAACATTACTGGTAAGCCATGTTGGAAGTCCACCACTCCTGCGAACAGTTGCACTTCCATTCGCAGAAGGAGTTGACGCGACAATAG

>Intergenic_Region|1043886..1043985|Liberibacter_crescens_BT-1_EZ-Tn5_Transposon_Disruption

AAATAGAACAGTACGATCCACATCTTTAACAACAAGAAATATAGTGCATCATACACTATTATGAATTTACTTTTAAAAAATATTCAAAAGCTTTTTTAAA

>B488_13080|1419471..1419570|Liberibacter_crescens_BT-1_EZ-Tn5_Transposon_Disruption

GGGCGGAAGCCACGCCGTGCTGACCGGCACCGGTTTCGGCGATGATTTCAGTTTTGCCCATCCGTTTGGCCAGCAGCACCTGCCCCAGCACTTGATTAGT

>B488_11490|1246874..1246775|Liberibacter_crescens_BT-1_EZ-Tn5_Transposon_Disruption

ATGTTAGTCTAGCAATTAAAGATCAGAATTTGCCAGCTGTAGGGTATGCTTTGCTAACAATGTTGTTAGTAATTATTTTTTATGATCAGTTGTTATTTCG

>B488_04910|539874..539970|Liberibacter_crescens_BT-1_EZ-Tn5_Transposon_Disruption

GGCTACTAAATAGAGGAAGGAACGCACCAACTGGCATCGGATCGTTGTTTTTAACCAGCATATTATTGATGTTGTAAGAAACTACGTGAACAAGGGCAGC

>Intergenic_Region|329143..329044|Liberibacter_crescens_BT-1_EZ-Tn5_Transposon_Disruption

CTAGTACTCCTCGGTATTCGGAGTTTGGTTAGGAGCAGTAAGGCGGTGAGCCCCCATAGCCTATCCAGTGCTCTACCCCCGAGGGTATTCAGTTAACGCT

>Intergenic_Region|329143..329044|Liberibacter_crescens_BT-1_EZ-Tn5_Transposon_Disruption

CTAGTACTCCTCGGTATTCGGAGTTTGGTTAGGAGCAGTAAGGCGGTGAGCCCCCATAGCCTATCCAGTGCTCTACCCCCGAGGGTATTCAGTTAACGCT

>B488_01190|141521..141422|Liberibacter_crescens_BT-1_EZ-Tn5_Transposon_Disruption

GCCTTTAGATCTAAATACTGCATTATCTTTAATCTCATTTATTATTTTGAAAAACTCTATACTACGATCTGTGCCTTGAATTTTTTTTGCTAATTTTTCA

>B488_07580|842787..842886|Liberibacter_crescens_BT-1_EZ-Tn5_Transposon_Disruption

CTACAAAGCCTTTTCATCCAAATCTTATGATGAAGCAAAAATCTCTGTTCCTGCCAACAGGAAACATGATGACATGGAAGGAAATCTTCCCCAAAAACCA

>Intergenic_Region|648515..648458|Liberibacter_crescens_BT-1_EZ-Tn5_Transposon_Disruption

GTCAACAATTCTGTACGCTACTTTTACAATTTGGTGATACTTTCGGGTAATGGATAATTAATAGGGTGTTAAGAGGGTTGTTATGAGCCATCATAAAGAA

>B488_02200|255227..255128|Liberibacter_crescens_BT-1_EZ-Tn5_Transposon_Disruption

CTATAGATGCTCCAGCAGGTACTATGGATGTAGTACTTGCTTCAGGCTGGCCTGGAGTAATGCTGCACGAAGCAGTTGGACATGGACTGGAAGGTGATTT

>Intergenic_Region|329143..329044|Liberibacter_crescens_BT-1_EZ-Tn5_Transposon_Disruption

CTATTACTCCACCGTATTCAGGAGTTTGGTTAGGAGTAGAAGGCGGTGAGCCCCCATAGCCTGTCCAGTGCTCTACCCCCGAGGGTATTCAGTTAACGCT

>B488_02370|269941..269884|Liberibacter_crescens_BT-1_EZ-Tn5_Transposon_Disruption

CTGTTGCTTTGATGTAATACAAGGGAGAGTTATGAGCCATCGTGCAACTATGGCATGTCATGCATCAATCAAATCTGGTAGAAAAATGCAAATTGAAGAA

>B488_01570|189906..189807|Liberibacter_crescens_BT-1_EZ-Tn5_Transposon_Disruption

CCCTTGCCCACGCCAAGCATTAACATTCTGCCCTAATAAAGTAATTTCTCGAACTCCTGACTGCACAAAAATAGAAGCTTCTTCGAAAACCTGTGCAACT

>B488_13430|1461076..1460977|Liberibacter_crescens_BT-1_EZ-Tn5_Transposon_Disruption

GGACATAGCGAATAATGAGAATGTGATAAATCCAGCTGTTAATGTCATCAATTTCAAGGCACCTATACGTTGATAGGCCGGTTTTACGCATAATGCTCCG

>Intergenic_Region|329124..329047|Liberibacter_crescens_BT-1_EZ-Tn5_Transposon_Disruption

CTATTACGTCTACGAATATAAAGGAGTTTGGTTACGAGCTGTAAGGCGGTGAGCCCCCATAGCCTATCCAGTGCTCTACCCCCGAGGGTATTCAGTTAAC

>Intergenic_Region|329139..329046|Liberibacter_crescens_BT-1_EZ-Tn5_Transposon_Disruption

ATATTACGTCCACGGTATAAAGGAGTTTGGTTAGGAGCAGTAAGGCGGTGAGCCCCCATAGCCTATCCAGTGCTCTACCCCCGAGGGTATTCAGTTAACG

>Intergenic_Region|326665..326764|Liberibacter_crescens_BT-1_EZ-Tn5_Transposon_Disruption

CTCTGATACTGGTTATCTTGAGTTCAGAAGAGGTGAGTGGAATTCCGAGTGTAGAGGTGAAATTCGTAGATATTCGGAGGAACACCAGTGGCGAAGGCGG

>Intergenic_Region|326673..326574|Liberibacter_crescens_BT-1_EZ-Tn5_Transposon_Disruption

GTATCAGAGGCAGTTCCAAGGTTGAGCCTTGGGATTTCACCACTGACTTAATCATCCGCCTACGCGCCCTTTACGCCCAGTTATTCCGAACAACGCTCGC

>B488_11490|1246859..1246789|Liberibacter_crescens_BT-1_EZ-Tn5_Transposon_Disruption

ACGGTATGTCGAAAAGTTAAGGACCAAAATTTGCCGGCTGTAGGGTATGCTTTGTTAACAATGTTGTTAGTAATTATTTTTTATGATTGATTGGACAAGT

>B488_09670|1042959..1043055|Liberibacter_crescens_BT-1_EZ-Tn5_Transposon_Disruption

GATCAACAGCAGCTGTGATATATGCAAGACCCCGAGATATTTGACCTTCCTGAAACAAAATATTTCCAAATAATGCGATCGCTTTAATATGACCTCTTTT

>Intergenic_Region|739201..739165|Liberibacter_crescens_BT-1_EZ-Tn5_Transposon_Disruption

GTGATACCATGTTGCGCATATTCTGTTTATTGATCCTTCAACTCAGCAAAAGTTCCACGTTAGGTTCATTCAGTCTTTTATGTTTTCTCGATATTCATTA

>B488_08580|940773..940674|Liberibacter_crescens_BT-1_EZ-Tn5_Transposon_Disruption

TCTATAGGCTTTTCAGAAGAAATGAGGCGTATCATAGAACTTCTTTCGCAGGTGGCTCCAGATGCTGTCGGACAACAACGCTCAGCTACTGCCATGTCTG

>Intergenic_Region|329143..329044|Liberibacter_crescens_BT-1_EZ-Tn5_Transposon_Disruption

CTATTACGTCCTCCGTATTCGGAGTTTGGTTAGGAGCTGTAGGCGGTGAGCCCCCATAGCCTATCCAGTGCTCTACCCCCGAGGGTATTCAGTTAACGCT

>B488_11490|1246874..1246775|Liberibacter_crescens_BT-1_EZ-Tn5_Transposon_Disruption

ATGTTGGTCTAGCAATTAAAGATCTGAATTTGCCAGCTGTAGGGTATGCTTTGTTAACAATGTTGTTAGTAATTATTTTTTATGATCAGTTGTTATTTCG

>Intergenic_Region|329143..329044|Liberibacter_crescens_BT-1_EZ-Tn5_Transposon_Disruption

CTAGTACTCCTCGGTATTCGGAGTTTGGTTAGGAGCAGTAAGGCGGTGAGCCCCCATAGCCTATCCAGTGCTCTACCCCCGAGGGTATTCAGTTAACGCT

>B488_12500|1365991..1365891|Liberibacter_crescens_BT-1_EZ-Tn5_Transposon_Disruption

TATAAATGGTGCTGCTTGTCGTAGTCGTAATGGTATTTGGACTCCATTATTCTAAGATTTTATAACTGAAACCTATTTATAATTAATAAAAAGTTATTTT

>Intergenic_Region|329124..329048|Liberibacter_crescens_BT-1_EZ-Tn5_Transposon_Disruption

CTATTACGTCTACGAATATAAAGGAGTTTAGGTTAGGAGCTGTAAGGCGGTGAGCCCCCATAGCGTATCCAGTGCTCTACCCCCGAGGGTATTCAGTTAA

>B488_06660|738834..738933|Liberibacter_crescens_BT-1_EZ-Tn5_Transposon_Disruption

GAACCAGCCAGGCCAGGAACAGAGGTAATGGCTCCACCAGTCGCTAAGGGAAGCAAAAAACCATTGCCCAAGGTGAGAAGCGAAACAGCACTAATACAGG

>B488_12400|1356353..1356254|Liberibacter_crescens_BT-1_EZ-Tn5_Transposon_Disruption

GATTATCGCCTGCCTTGGTCCATCCATTACCTCGGCATATTATGAAGTAGGTCCAGAATTTGTAAAACAGTTTTTAGCATATGATATTTCGTATCAAGAT

>B488_08600|943372..943273|Liberibacter_crescens_BT-1_EZ-Tn5_Transposon_Disruption

CGATAAGGCTTTAAAATATGCAGAGAAAGGCAATGTTTCTGCACAGACTTTGGTAGGATTGATTCTAGGTGAAGGTTTGGGTGTAAAACGTAATATAAAA

>B488_05210|563233..563332|Liberibacter_crescens_BT-1_EZ-Tn5_Transposon_Disruption

GTATGAAGCTGCAGCGGAACAGTTTCTGCGCTGGGGCAACGATCACAGGAACGGCAAACTTGTTCCCGTTGCTGGATTAATGAAGCGCAGGAAAGAGGAG

>B488_03970|459654..459753|Liberibacter_crescens_BT-1_EZ-Tn5_Transposon_Disruption

CTGTAACAGATAATGTGTTATCATTTATTCTTTCCAGGACGAGATAATCACCTTTATAGGCCAGACCAGCGTTATAAATGCGGGCATTGTTATATATATA

>Intergenic_Region|329143..329043|Liberibacter_crescens_BT-1_EZ-Tn5_Transposon_Disruption

CTAGTACTCCTCGGTCTTCGGAGTTCGGTCCGGAGCAGTCAGGCGGTCAGCCCCCATCGCCTATCCAGTGCTCTACCCCCGAGGGTACCAGTTCACGCTC

>Intergenic_Region|329143..329044|Liberibacter_crescens_BT-1_EZ-Tn5_Transposon_Disruption

CTAGTACTCCTCGGTATTCGGAGTTTGGTTAGGAGCAGTAAGGCGGTGAGCCCCCATAGCCTATCCAGTGCTCTACCCCCGAGGGTATTCAGTTAACGCT

>B488_03730|426938..427027|Liberibacter_crescens_BT-1_EZ-Tn5_Transposon_Disruption

TGCCTTGGGTCTTGATCATGCAGGGAATATGGTGAACACTAACCTATGGTACCGAAACGATACGCAACGATCCATTTAACAAGGGGCTGTCTAACCCAGT

>B488_12500|1365983..1366082|Liberibacter_crescens_BT-1_EZ-Tn5_Transposon_Disruption

CCATTTATATCTACCTGCGCATCAGAAAGAAATATTTTATGTATATATTGCCGACTATTCTCAGAACCAACTTGATATGGAGAAAAAGCTATGACCTGCC

>B488_12770|1391254..1391350|Liberibacter_crescens_BT-1_EZ-Tn5_Transposon_Disruption

GATAAGCGTGCAATAATTCTCTGGAGTTTTTACAATCATGCGCTCAATATCGCTATCTTCGCTTCCTCTCACCATTAACATCTAACCATATTGATCCTTC

>B488_12170|1330384..1330285|Liberibacter_crescens_BT-1_EZ-Tn5_Transposon_Disruption

TGCCTATACTGGTCGTTCGCTGCAACTAGAACTTAATATGATTAAAAAATATCTGGATACAGCTCATAGGCCAATTATTGCCATTATTGGTGGTTCTAAA

>B488_05540|600839..600740|Liberibacter_crescens_BT-1_EZ-Tn5_Transposon_Disruption

CAGGAAGAGATTGCTAAGACAAACCGGGATAAAGAAGAATAAGAGAGCAACTGTCATTGCGGGAAGTAATATTATATATAGGCAGACAAAACACATTAGT

>B488_08300|908306..908207|Liberibacter_crescens_BT-1_EZ-Tn5_Transposon_Disruption

GCAGAACTTTCACGTGCATTGGCGATTGCTACTGTTATATTATCAACAGAAGAACTGATTTTATTTTTATCTATTGTTGTTATTAAAGAATCAAAATGTT

>B488_11490|1246874..1246775|Liberibacter_crescens_BT-1_EZ-Tn5_Transposon_Disruption

ATGTTGGTCTAGCAATTAAAGATCAGAATTTGCCAGCTGTAGGGTATGCTTTGTTAACAATGTTGTTAGTAATTATTTTTTATGATCAGTTGTTATTTCG

>B488_11490|1246874..1246775|Liberibacter_crescens_BT-1_EZ-Tn5_Transposon_Disruption

ATGTTGGTCTAGCAATTAAAGATCATAGTTTGCCAGCTGTAGGGTATGCTTTGTTAACAATGTTGTTAGTAATTATTTTTTATGATCAGTTGTTATTTCG

>B488_05400|582728..582800|Liberibacter_crescens_BT-1_EZ-Tn5_Transposon_Disruption

ATATAACCTAATATTGGTCCAGCTATGGCGCTGTAAATGGCGGGAAGATCCGCGATTGTTGGTTGAGCAGATTAGTTGAAATGAGAAAACCAAGAGTACA

>B488_01350|166778..166877|Liberibacter_crescens_BT-1_EZ-Tn5_Transposon_Disruption

AATCCATATGAGGCTGCGTAATGCATGGCCATTTGATTTATAGTTGAAACATGAAGATCAGAAGCTTGTTTGATCAAGATAATTTTCTTTATCACTTCTA

>B488_07740|853367..853466|Liberibacter_crescens_BT-1_EZ-Tn5_Transposon_Disruption

CCTGTAAAAGGATCCCTGATCATTCTCTTTCCCGTCTGGACATAATCCAGGCGTGCATCAGGAGTCACCTGGTTGATATTGCCAATCGCTGAACTGCTGA

>B488_10280|1125990..1126089|Liberibacter_crescens_BT-1_EZ-Tn5_Transposon_Disruption

GATAAAGCACCTGTTATCAAAGTCTCTTTTAAAAAAGTGAATAACCCACAAAAAAACAATCTCTTAGATCTATCCATAACTAAACTTTCTCAACTTCAGG

>Intergenic_Region|329143..329057|Liberibacter_crescens_BT-1_EZ-Tn5_Transposon_Disruption

CTAGTACTCCTCGGTATTCGGAGTTTGGTTCGGAGCAGTCAGGCGGCAGCCTTATAGCCTATCCAGTGCTCTACCCCGAGGGTACCAGCCACGCCCTACC

>B488_12300|1345941..1345842|Liberibacter_crescens_BT-1_EZ-Tn5_Transposon_Disruption

GGATTACGGTGGTCAGATCGAGCAGAAGATTATCGTACTGAAGCGTTAGGTCTGGTAAAGGCACAAAGAGTTAAGAATGCAGTGATTGTTCCTGTGGGAG

>Intergenic_Region|111863..111962|Liberibacter_crescens_BT-1_EZ-Tn5_Transposon_Disruption

TCTCTCTGGAGAAGCCTTAACAAAATTCTTGGAAAATTTTATTTATTAATTTTTTTTGAAGTTCCTTATTTGCATTTGAGAAAATATTGCTTTAATATTT

>Intergenic_Region|329143..329044|Liberibacter_crescens_BT-1_EZ-Tn5_Transposon_Disruption

CTAGTACTCCTCGGTATTCGGAGTTTGGTTAGGAGCAGTAAGGCGGTGAGCCCCCATAGCCTATCCAGTGCTCTACCCCCGAGGGTATTCAGTTAACGCT

>B488_04690|525793..525892|Liberibacter_crescens_BT-1_EZ-Tn5_Transposon_Disruption

CTGTCAGCCCATCAGGAGAAGCACCACTGTCAGGAATATCGGGATGAACAACAAAGCCTGTTTCCTGAACATCCTCGTTGACGATGAACTGATAGTTTTC

>Intergenic_Region|329143..329044|Liberibacter_crescens_BT-1_EZ-Tn5_Transposon_Disruption

CTAGTACTCCTCGGTATTCGGAGTTTGGTTAGGAGCAGTAAGGCGGTGAGCCCCCATAGCCTATCCAGTGCTCTACCCCCGAGGGTATTCAGTTAACGCT

>Intergenic_Region|329143..329044|Liberibacter_crescens_BT-1_EZ-Tn5_Transposon_Disruption

CTAGTACTCCTCGGTATTCGGAGTTTGGTTAGGAGCAGTAAGGCGGTGAGCCCCCATAGCCTATCCAGTGCTCTACCCCCGAGGGTATTCAGTTAACGCT

>Intergenic_Region|329143..329044|Liberibacter_crescens_BT-1_EZ-Tn5_Transposon_Disruption

CTAGTACTCCTCGGTATTCGGAGTTTGGTTAGGAGCAGTAAGGCGGTGAGCCCCCATAGCCTATCCAGTGCTCTACCCCCGAGGGTATTCAGTTAACGCT

>B488_03720|426216..426117|Liberibacter_crescens_BT-1_EZ-Tn5_Transposon_Disruption

CATCTATATGATGCACATTTATGACAGCGTCATTGGCACACAAAGTGAAATAAATCTTGTTGCTATAAGCACTATAGCTCTCTTTTTTTACATACTTTTC

>Intergenic_Region|329073..329172|Liberibacter_crescens_BT-1_EZ-Tn5_Transposon_Disruption

GCACTGGATAGGCTATGGGGGCTCACCGCCTTACTGCTCCTAACCTAACTCCGAATACCGAATACTACTAATTGGCGGACACACTGCGGGTGCTAACGTC

>B488_11810|1289608..1289530|Liberibacter_crescens_BT-1_EZ-Tn5_Transposon_Disruption

GCCTCATGCTGATGAACCTCCTGTTGCTTTGTGTGCAGGAGGCTTTTCTGTATCATTGGTCGAGGGAGTTACAGGATCTGGAAAGACTGAAGTTTATTTG

>Intergenic_Region|328930..328843|Liberibacter_crescens_BT-1_EZ-Tn5_Transposon_Disruption

CTAGTACTCCTGGTGTTATCCAACCTTCAACCTAGCTCATGGCTAGATCACTCGGTTCTCGGGTCTAATACAACAAACTATAGCGCCCTATTCAGACTCG

>B488_10540|1148505..1148435|Liberibacter_crescens_BT-1_EZ-Tn5_Transposon_Disruption

CAGATGATCCTTCAACTAGTCAAAAGTTCATTAAATGTTAAAGAAGGAGAGATATTATTCATATAATTTCCACAAAATCTACATCACCCCCTTAATTTAT

>Intergenic_Region|329143..329044|Liberibacter_crescens_BT-1_EZ-Tn5_Transposon_Disruption

CTAGTACTCCTCGGTATTCGGAGTTTGGTTAGGAGCAGTAAGGCGGTGAGCCCCCATAGCCTATCCAGTGCTCTACCCCCGAGGGTATTCAGTTAACGCT

>B488_07940|870857..870758|Liberibacter_crescens_BT-1_EZ-Tn5_Transposon_Disruption

GCCGTAAATAGGCAAGCGTCAGGTCATGCAGGGTAATGGCTTTCGTCATCGTCAGGCCTTTGCCCAATAGGCGACAGACCATAGCATGGCGATCAAAACC

>Intergenic_Region|329143..329050|Liberibacter_crescens_BT-1_EZ-Tn5_Transposon_Disruption

CTAGTACTCCTCGGTATTCGGAGTTTGGTTAGGAGCAGGAAGGCGGTGAGCCCACTAACCCTATCCACTGCTCTACCCCCGAGGGTATTCAGTTGGGCCT

>B488_12480|1363355..1363453|Liberibacter_crescens_BT-1_EZ-Tn5_Transposon_Disruption

CTATCGTACATGGCTCTTAAACCACATATTTCAAAATATGCTGATTATAGAAAAAATAAAAAAACAAAGCTTCTAAAACCATTTATCCATGTTCTTCAGG

>B488_11490|1246874..1246775|Liberibacter_crescens_BT-1_EZ-Tn5_Transposon_Disruption

ATGTTGGTCTAGCAATTAAAGATCAGAATTTGCCAGCTGTAGGGTATGCTTTGTTAACAATGTTGTTAGTAATTATTTTTTATGATCAGTTGTTATTTCG

>Intergenic_Region|254336..254435|Liberibacter_crescens_BT-1_EZ-Tn5_Transposon_Disruption

GTGGTATTCGATCAATAACATCATGATTAAAAGTGCGTGTCTATTGCAATATCTTCAGAACACAGGATATTTGTAAATGCCTACCAATTCGGTATGTTTG

>B488_07100|790992..791057|Liberibacter_crescens_BT-1_EZ-Tn5_Transposon_Disruption

CTGATAAAAAAAAGAACAGCCGCTGACCATAAGTAATGATTTTACGATCTTCAACACAATGAATAATTTTTTTTACTTCTGGATGATCCTTCAACTCAGC

>B488_13420|1459557..1459642|Liberibacter_crescens_BT-1_EZ-Tn5_Transposon_Disruption

CTATAACACCAGTCAAGTCTACGAAATTGTCAAAGATTATAATCTTGCAGAAAAGCAGCTTCGGCTAGCAATTTCATATGATCCTTCAACTCAGCAAAAG

>B488_08150|885553..885652|Liberibacter_crescens_BT-1_EZ-Tn5_Transposon_Disruption

CTCCTTCCCTGCTCAAGGGAAAATACGGGGATCACCTTACAGGTTAAAGACGATCATGAGGATCTCAATACTCAGCCGGTAACAATTAATCTTCTTAAAG

>Intergenic_Region|329143..329044|Liberibacter_crescens_BT-1_EZ-Tn5_Transposon_Disruption

CTAGTACTCCTCGGTATTCGGAGTTTGGTTAGGAGCAGTAAGGCGGTGAGCCCCCATAGCCTATCCAGTGCTCTACCCCCGAGGGTATTCAGTTAACGCT

>B488_08310|909196..909295|Liberibacter_crescens_BT-1_EZ-Tn5_Transposon_Disruption

GACTTGTAAAGTCACAACATATTTTCCATGTTGAAAGCCCTTTAGAAGGAAACAGCAACAATGCTTTCCTAAATTCCCTTAATAAGAGCTTTACAAAAAT

>B488_08310|909196..909295|Liberibacter_crescens_BT-1_EZ-Tn5_Transposon_Disruption

GACTTGTAAAGTCACAACATATTTTCCATGTTGAAAGCCCTTTAGAAGGAAACAGCAACAATGCTTTTGTAAATTCCCTTAATAAGAGCTTTACAAAAAT

>B488_12300|1345941..1345842|Liberibacter_crescens_BT-1_EZ-Tn5_Transposon_Disruption

GGATTACGGTGGTCAGATCGAGCAGAAGATTATCGTACTGAAGCGTTAGGTCTGGTAAAGGCACAAAGAGTTAAGAATGCAGTGATTGTTCCTGTGGGAG

>Intergenic_Region|329143..329044|Liberibacter_crescens_BT-1_EZ-Tn5_Transposon_Disruption

CTAGTACTCCTCGGTATTCGGAGTTTTGGTTAGGAGCAGTAAGGCGGTGAGCCCCCATAGCCTATCCAGCGCTCACCCCCGAGGGTATTCAGTTCACGCT

>B488_08300|908298..908397|Liberibacter_crescens_BT-1_EZ-Tn5_Transposon_Disruption

AAGTTCTGCTTCTATAAAAAGCATAACAGAGCAGCTTTCTCAAAAACAGAACGAAATGACTCAAACAATCCATAATTTCAATGAAATGTCTCAGCATTTT

>B488_10530|1147646..1147547|Liberibacter_crescens_BT-1_EZ-Tn5_Transposon_Disruption

TCCTTATGGAGTGTCGTCCCCACAAAATATTTTAAAATATCTTCTTGAAAATAAACAATGACTTTTATCAAATTATCTCCTTTTTTCAAAAAGATGAATT

>B488_05990|660649..660550|Liberibacter_crescens_BT-1_EZ-Tn5_Transposon_Disruption

CTTTAAAATATCTGCATACCAAATACTATTTGGATAATTCTTATGAAGGACTGCAGCTGCAACTTGTACTTCATTCATAATACCCATTGCATAATAAGCT

>Intergenic_Region|1127302..1127203|Liberibacter_crescens_BT-1_EZ-Tn5_Transposon_Disruption

GGATAAGAGTATATTGATATACTTCACTGAGATATCTAAGGCATAAATCTGGTTGTTAGTTTTTTGAACAAGTTAAGATGAGTTTTAAATTATAAATAGA

>B488_12840|1400316..1400217|Liberibacter_crescens_BT-1_EZ-Tn5_Transposon_Disruption

ATTCAGGATTCTCAAAAAGCAGTTGATTTGGCTAATGCTTTTATTAAAGGAGGAGAAGCAACTACTCAAACAGCATCAGCGGGTCAATCTTTATCTTTAC

>B488_07830|860839..860938|Liberibacter_crescens_BT-1_EZ-Tn5_Transposon_Disruption

TTCCAGGAGCGTGGATCAAATTCAATCCAGCGATCCCGCAATCGTGTCATCCGCGCCCTGTCCTGATGCTGGACCAGTAACCGTAACAAACCACGGAACA

>B488_03770|435282..435183|Liberibacter_crescens_BT-1_EZ-Tn5_Transposon_Disruption

GATATAGCAGCTCGTGGCCTTGATATTCATGATGTAGGACATGTTTTTAATTTTGATGTTCCAATTCATGCTGAAGATTATATTCATCGGATTGGGCGTA

>B488_11610|1260871..1260772|Liberibacter_crescens_BT-1_EZ-Tn5_Transposon_Disruption

GTATCACAGCTTGTTCAGCTTGCTAATGCTGTTAATCAATTAGTAGGAGGACCGTCGATACTGGCTTTTGATGAACTGCGCTCTAGGTTAGGGATTGATA

>B488_06100|673737..673638|Liberibacter_crescens_BT-1_EZ-Tn5_Transposon_Disruption

AAATAGTATCAGCAGCAAATCCGGATGTAATCATTCTATTGGATTATCAAAAAGGAAATGACTCTATGATGTTGCAGCATTTCCTGGAAAAACACCCTCT

>B488_02000|237298..237397|Liberibacter_crescens_BT-1_EZ-Tn5_Transposon_Disruption

ATAGCAGTTCGAGATAAGCTTGTTTCCGCTTATCTCGAAATTACAAAAATGCAGATATAATTGAAGGAAAATTAACCTATGAGAGCTCTTTCTATTGCAG

>Intergenic_Region|433287..433386|Liberibacter_crescens_BT-1_EZ-Tn5_Transposon_Disruption

CCTTCTGTTCAGCCTCAAACTCCTATTAGCGATGGTGCGGGCCTAGGTTCTCATACTAGCGGAGTTGATAGCGAGCATAAAAAACCTACCCTTGACCCTG

>Intergenic_Region|433294..433196|Liberibacter_crescens_BT-1_EZ-Tn5_Transposon_Disruption

GACAGAAGGCTGCTCAGATTGTTGCTGCTGAACAGGAGTCCCCTCAGATTTTTGCTGCGGAACAGAAGTCCCCAAACCTGTGTCCGTAGCGCCGCTAGGA

>Intergenic_Region|329073..329172|Liberibacter_crescens_BT-1_EZ-Tn5_Transposon_Disruption

GCACTGGATAGGCTATGGGGGCTCACCGCCTTACTGCTCCTAACCAAACTCCGAATACCGAGGAGTACTAGTTGGCAGACACACGGCGGGTGCTAACGTC

>B488_07290|813155..813056|Liberibacter_crescens_BT-1_EZ-Tn5_Transposon_Disruption

CTTTATGATCATCAGTATTTTGCGCTACATGAGTGTAGATAACATTAAAAGTTGTTGGTATCAATTTTCCTACAACTGTAAAAGGTGCTTTCTGTGAGGA

>B488_03090|354932..355031|Liberibacter_crescens_BT-1_EZ-Tn5_Transposon_Disruption

GTCCATATAATAATAAAGGTTATCATATCTATCCTAGTAACTTTTTCCTTGAAGACAAGAACTGCAATTAAAAAAGAAATAGTTGGTGAAACATACTGTA

>B488_01000|116614..116713|Liberibacter_crescens_BT-1_EZ-Tn5_Transposon_Disruption

ATATACTTCTAAAGAAGTTACCGCTTATACTATTAACATTTTAAAAGATGATATCTCTTTAGCTGTTGATATACTTGGAGATATGTTAAGTAATTCTTTA

>B488_03770|435282..435183|Liberibacter_crescens_BT-1_EZ-Tn5_Transposon_Disruption

GATATAGCAGCTCGTGGCCTTGATATTCATGATGTAGGACATGTTTTTAATTTTGATGTTCCAATTCATGCTGAAGATTATATTCAGCGGATTGGGCGTA

>Intergenic_Region|917503..917602|Liberibacter_crescens_BT-1_EZ-Tn5_Transposon_Disruption

GTATAATGTCATTCATAGATATACAATTTTCATTAATCATCTTTTTGAGATAAAATCAAAGTCTTTTAAAATATTACTTCCAACGTACCAAGGGCGGCAA

>B488_09000|686479..686540|Liberibacter_crescens_BT-1_EZ-Tn5_Transposon_Disruption

GTTATTGTAGGACTACCATCTATTGTGAAACTAAGTTTTTTCTTTTTAAAAGCATAATACAAGGGGTGTTATGAGCCATAGAAAAAGCAGGAAGAAGCAT

>B488_00530|67434..67533|Liberibacter_crescens_BT-1_EZ-Tn5_Transposon_Disruption

TTCAACAGGAGAGCAAAAAGTGCTTCTAATGAGTATTTTTTTAGCTCATGCACGCTTAATATCTTCTACAACTGGTTTTAGACCTATTTTACTATTAGAT

>B488_01290|159297..159198|Liberibacter_crescens_BT-1_EZ-Tn5_Transposon_Disruption

CCTCAGCCTCTCAACGCAATCATACAAAATGGACAAAATATCTCATATCTCGTATGAATGCAGTAATAACTACAAGTAAAAAATCTGCTGCCTTTATCAA

>B488_09000|686479..686540|Liberibacter_crescens_BT-1_EZ-Tn5_Transposon_Disruption

GTTATTGTAGGACTACCATCTATTGTGAAACTAAGTTTTTTCTTTTTAAAAGCATAATACAAGGGGTGTTATGAGCCAAAATGGTATTACCAACATATTC

>Intergenic_Region|95037..95136|Liberibacter_crescens_BT-1_EZ-Tn5_Transposon_Disruption

CTTATATTCAGTTTATCGGTTACTTGCTTGTTGTATTGTTGATAGTATCATGTTTTGGTAAGTAACTTTTATTGAAAAAGTGAGACATATGTTTGTAGAA

>B488_03720|426040..425941|Liberibacter_crescens_BT-1_EZ-Tn5_Transposon_Disruption

TACATACAGTATTGAAAAAATATGACCCAACTGAACAAACTTTTGCTTCAACAGCAAATGCTCTTGATAAATTTAAGCAATTTGTTACCTCTCCTATAAT

>B488_00130|17149..17248|Liberibacter_crescens_BT-1_EZ-Tn5_Transposon_Disruption

CATCAATCCTAGTAGGGGATCTTAATGTTGCTCCGCTTGAAAATGATGTTTGGTCACATAAAAAAATGTTGGGAGTTGTTAGTCATACACCTGTAGAAAC

>Intergenic_Region|1147352..1147451|Liberibacter_crescens_BT-1_EZ-Tn5_Transposon_Disruption

AACCAATGCTGAATATTTTTAATAAAAGATGTTCTGTTCTCTTCATTATAGGATTCTATAATTAGAGAAGAGAATTTTTGAAATAAGCGATGTAATAGCC

>B488_08930|970438..970537|Liberibacter_crescens_BT-1_EZ-Tn5_Transposon_Disruption

CCTCAAAGGCAGCAGTATAAACAAGCTTCTTCTTCGGTTTTTTATGGCCTTTGCCAGCGTTTTGTTGAGGTTCTTCTCGTGAAATTGCCGCTGAAGAAAC

>B488_11470|1243653..1243752|Liberibacter_crescens_BT-1_EZ-Tn5_Transposon_Disruption

CTTTTGTCATTTCTACCTAATTTAAGATGGATTGGCTATATTTCCACAACAAGTGTTTACGGTAATCATGACGGAAGATGGGTTACCGAAAAAACTCCTG

>B488_06540|723597..723498|Liberibacter_crescens_BT-1_EZ-Tn5_Transposon_Disruption

GAGTTATCCGGCTTAATAACATAGCCAGTGCTACTGTTTAAATCATCGATGCTTGTATTCTCCTGGCTTATCCTCTTTGACAATATGCTGTTTAATCCAG

>B488_05570|605267..605168|Liberibacter_crescens_BT-1_EZ-Tn5_Transposon_Disruption

CATCTATACTGCTTTCAAGTTGCAAAACGATAACGCCGGAAGAACAACGAGCCTATGATCGATCAAAATGCAGTTCATATGGCTTTAAAGAAAGAACGGA

>B488_08600|942575..942674|Liberibacter_crescens_BT-1_EZ-Tn5_Transposon_Disruption

ATTCAGGATATGCTTAAGGAATGCGCAGCAATAAAAGAGTCTGCTTTTTTTACTCCAAGATTTTGTAAAATATCAGGAACACCAACATAAAAACCTTCTA

>B488_03770|435282..435183|Liberibacter_crescens_BT-1_EZ-Tn5_Transposon_Disruption

GATATAGCAGCTCGTGGCCTTGATATTCATGATGTATGACATGTTTTTAATTTTGATGTTCCAATTCGTGCTGAAGATTATATTCATCGGATTGGGCGTA

>B488_03770|435282..435183|Liberibacter_crescens_BT-1_EZ-Tn5_Transposon_Disruption

GATATAGCAGCTCGTGGCCTTGATATTCATGATGTAGGACATGTTTTTAATTTTGATGTTCCAATTCATGCTGAAGATTATATTCATCGGATTGGGCGTA

>Intergenic_Region|1048610..1048709|Liberibacter_crescens_BT-1_EZ-Tn5_Transposon_Disruption

ACTCAATACTATAAGAAGAAAGGACTGTGATCCTTTAAACATATTTTGCTAAGCTATAAGCTAACAGTGCATAGATTATCACTATCTAGACCTTCTTTCG

>B488_09670|1042956..1043055|Liberibacter_crescens_BT-1_EZ-Tn5_Transposon_Disruption

GAACAACAGCAGCTGTCATATATGCAAGACCCCGAGATATTTGACCTTCCTGAAACAAAATATTTCCAAATAATGCGATCGCTTTAATATGACCTCTTTT

>Intergenic_Region|1311499..1311598|Liberibacter_crescens_BT-1_EZ-Tn5_Transposon_Disruption

GCTTTAAATAAAGATTTTTTACCGTATAAAAGCCATTTTCTAAACCTTTACAAGATTTAAATCGTTACTCAGAAATTTTTTCTAATCCTTAAATCCTTTT

>B488_06810|757645..757744|Liberibacter_crescens_BT-1_EZ-Tn5_Transposon_Disruption

ATTGAGTATAGAGTTGAATTGCTGATTATCCAGATAAAGATCTTCTGGAGAAATTGGTTTATATGGAATGCTGTCTTTTTTAGTATTAGTAGTAAAATTA

>B488_05620|610369..610468|Liberibacter_crescens_BT-1_EZ-Tn5_Transposon_Disruption

CTCTGGATCACCGTTTCGTCGCTACGCTTGTAGTGGCAATCATGGGAATGTATCTTGGTCGTCGGTTTCGTATTCCAGCTGGTGCAATGTTGATGCCAAT

>B488_05690|619651..619552|Liberibacter_crescens_BT-1_EZ-Tn5_Transposon_Disruption

GTTATGTACATCAGGAGTAACCCAGAATGGCCAGAACTGTTTTATGGCTGCAGTTTTTATACTCTCTGCATGCCCACCAAAAGAAATTTCTGGAGAATTT

>B488_10700|1166865..1166766|Liberibacter_crescens_BT-1_EZ-Tn5_Transposon_Disruption

ATTTATATTCGAAAAGTGAACCAAGAAGGTCCTCTTTTGCAGTTTGTATTTCCTAAAAAATTAGAAGAATTAGCTGGAGAGAAAACCGAAAATAGCCTTC

>Intergenic_Region|386589..386688|Liberibacter_crescens_BT-1_EZ-Tn5_Transposon_Disruption

GCTTTAGTGTTCGAGTAGGATCAAGTATGATTGCTCACTTTCTTGTTATGTTTTTGAATGCAGAAAAAGTCATATTTTCTGTTAAAGGTATTCAAATTAA

>B488_03230|371195..371294|Liberibacter_crescens_BT-1_EZ-Tn5_Transposon_Disruption

ATTATAATCATAGCCAAAAACTTTAAATACATCTTTGGGATTTAAAGTCGCTTTTATTGCTAGTGGAATAGCATGAGTTTCAGGATTGTGCCATTCACCA

>B488_05410|584600..584699|Liberibacter_crescens_BT-1_EZ-Tn5_Transposon_Disruption

GGATATTATTGCCGTCACCTGCTTTCAGCGTATCATTACCCTCGCCGCCAATGATCAGGTCATCGCCATTGCCTCCGGCCAATTTATCGTTGCCCTGTGT

>B488_06640|737337..737238|Liberibacter_crescens_BT-1_EZ-Tn5_Transposon_Disruption

TACCAGTATAGCAAAATGAACACGTAAGAGTACATCCAACTTGACTGGAAACACAAAGAGTGCCACGATCTTTTTCTGGAATATAAACTGTTTCTACTTC

>Intergenic_Region|330202..330103|Liberibacter_crescens_BT-1_EZ-Tn5_Transposon_Disruption

ATCCTACACATACCTACACAAATGCCAGTGTAAAGTTATAGTAAAGGTGCACGGGGTCTTTCCGTCTGACCGCAGGAACCCCGCATCTTCACGGGGAATT

>Intergenic_Region|330194..330293|Liberibacter_crescens_BT-1_EZ-Tn5_Transposon_Disruption

GTGTAGGATAGGTGGTAGGCTTTGAAGCAAGGGCGCCAGCTTTTGTGGAGCCATCCTTGAAATACCACCCTTATCCACATGGATGTCTAACCGCGGTCCG

>Intergenic_Region|330202..330103|Liberibacter_crescens_BT-1_EZ-Tn5_Transposon_Disruption

ATCCTACACATACCTACACAAATGCCAGTGTAAAGTTATAGTAAAGGTGCACGGGGTCTTTCCGTCTGACCGCAGGAACCCCGCATCTTCACGGGGAATT

>B488_11250|1225535..1225436|Liberibacter_crescens_BT-1_EZ-Tn5_Transposon_Disruption

TCCTATTACTGTTCAAGAAAAAAATTATGTTGTTGATGTCCATGCTATCCTTTCTGCTGTTACTGAAAGAACCAAGATTATTTTCATTTCTAATCCAGGT

>Intergenic_Region|1048668..1048767|Liberibacter_crescens_BT-1_EZ-Tn5_Transposon_Disruption

AGCTAACAGTGCATAGATTATCACTATCTAGACCTTCTTTCGCTTTTTTTCTACGAATCTTCTAAACTTAAGGAAAAATGAGAAAAAATCCTCTATGACA

>B488_00020|3867..3768|Liberibacter_crescens_BT-1_EZ-Tn5_Transposon_Disruption

ACTACACTCAATAGCACTTTTATGTTCTATAAGAGAAAAAGTCCAATGATTACACGCTTTATTAACTTCATTTTTATAACTACAACCTTTATAAAAAAAA

>B488_02370|270445..270544|Liberibacter_crescens_BT-1_EZ-Tn5_Transposon_Disruption

ATATGAAGGAGAATATTTTATATCCAACTCTTTATTGAGAAGAGGTTTCCCAAATTTATTGTGGAAAGAAGAAAGCATACTTGTACTAAGAGTAGAGGAA

>Intergenic_Region|408726..408627|Liberibacter_crescens_BT-1_EZ-Tn5_Transposon_Disruption

GATGAATAGTATTTACATTAAGGAATAAATTCTTATTTATGCTTTATCTAAGATTAAAATATTTATTTTAATCATTAAGAATGTTTTTTAAAAACTTTTT

>Intergenic_Region|329076..329172|Liberibacter_crescens_BT-1_EZ-Tn5_Transposon_Disruption

GTTCTGGATAGGCTATGGGGGCTCACCGCCTTACTGCTCCTAACCTAACTCCGAATACCGAGGATTACTAATTGGCAGAAACACCGCCGGTGCTAACGTC

>B488_11990|1308687..1308588|Liberibacter_crescens_BT-1_EZ-Tn5_Transposon_Disruption

GGTGGACGTTGTGCTAATTCAGTTGCTTCTTTGAGCCAACTAGATCCAAAATTCAATTGAATTGAAGATTTTATCCATTCTGGAATGTCTGCTTGAATGT

>B488_01270|156405..156306|Liberibacter_crescens_BT-1_EZ-Tn5_Transposon_Disruption

AAGCAATACGCAACATATTTGTTGCTCCAGGAGTACCAAGAGGAAAGCCAGCAGAAATTATGATACGATCTCCAGGCTTTCCAAATCCTGTACTTACAAC

>B488_03390|390702..390642|Liberibacter_crescens_BT-1_EZ-Tn5_Transposon_Disruption

TCCTGAGGCTTAGAAAAATCTTGTAAAGATTTTATATTTGAATCTCGTAATACAAGGGGTGTTATGAGCCATAGCCTGCTTTCCTGTCGTATTTTTAAAC

>B488_03770|435986..435887|Liberibacter_crescens_BT-1_EZ-Tn5_Transposon_Disruption

CACGTGCACGGATGCCACTTGCTCTTATATTAGAACCAACTCGAGAACTCGCTGCACAAGTCGCGGACAGTTTTGAAAAATATGGAAAAAATCATAAGCT

>B488_07730|851908..852007|Liberibacter_crescens_BT-1_EZ-Tn5_Transposon_Disruption

CTTAAAGGGGCCGTATCATAGGTTCCCTGTGTTGTTGTATAAACAATCCGATTTTTGGAAGAAGCGAGGCTGATCAATGTTTGCAAGGCTTTTGGTGCCA

>B488_11230|1223601..1223502|Liberibacter_crescens_BT-1_EZ-Tn5_Transposon_Disruption

GTCTATAGTTAAATTTTTTACTTCTCCTTTGATAAAGCATAAACTTAGGATGTCTTCTTCTAAGTTTTTATTTGGATTTTGGAATAATAAAACATCACTT

>B488_03230|371195..371294|Liberibacter_crescens_BT-1_EZ-Tn5_Transposon_Disruption

ATTATAATCATAGCCAAAAACTTTAAATACATCTTTGGGATTTAAAGTCGCTTTTATTGCTAGTGGAATAGCATGAGTTTCAGGATTGTGCTATTCACCA

>B488_10340|1130685..1130586|Liberibacter_crescens_BT-1_EZ-Tn5_Transposon_Disruption

GTATAATATAATCTCAGGATAAAACTTAAGGGGAGATCTGTTGTGATCAATACAATTAATTTTAATCTTTCTGCGAGTGATCTTTCTTCGTTACTTTGTA

>B488_06170|680523..680424|Liberibacter_crescens_BT-1_EZ-Tn5_Transposon_Disruption

GTATTCAGATATGCTTATACAGAAGCAGAAAAAAACGCAATTATGTCAAGAATCTCTTTCAAAAAAATTAAATAAAATTCCTTCAACAACACAAGAATCT

>B488_05470|592758..592659|Liberibacter_crescens_BT-1_EZ-Tn5_Transposon_Disruption

ATGCCAAACATCTGATACGTCTTATCTGGAGAACACGAATATTCGATACATCTATTGAGGATAATATTCTCCAAAATTTTTCATCTCTGCTCACTGCTGC

>B488_03970|459654..459753|Liberibacter_crescens_BT-1_EZ-Tn5_Transposon_Disruption

CTGTAACAGATAATGTGTTATCATTTATTCTTTCCAGGACGAGATAATCACCTTTATAGGCCAGACCAGCGTTATAAATGCGGGCATTGTTATATATATA

>Intergenic_Region|329143..329044|Liberibacter_crescens_BT-1_EZ-Tn5_Transposon_Disruption

CTAGTACTCCTCGGTATTCGGAGTTCGGTTAGGAGCAGTAAGGCGGTGAGCCCCCATAGCCTATCCAGTGCTCTACCCCCGAGGGTATTCAGTTAACGCT

>B488_01170|137240..137141|Liberibacter_crescens_BT-1_EZ-Tn5_Transposon_Disruption

TTTAAAAAGAGCAGGTATATTTGTAGCAAATCATGAACTTAATGCTTTTCTTGCTAGAAATCCTGAGCTTAACACTACCCCTGAAGAAGTTCGAGCTCAC

>Intergenic_Region|329143..329044|Liberibacter_crescens_BT-1_EZ-Tn5_Transposon_Disruption

CTAGTACTCCTCGGTATTCGGAGTTTGGTTAGGAGCAGTAAGGCGGTGAGCCCCCATAGCCTATCCAGTGCTCTACCCCCGAGGGTATTCAGTTAACGCT

>B488_01690|202445..202544|Liberibacter_crescens_BT-1_EZ-Tn5_Transposon_Disruption

TTTCTGCTGTTCAATAATATCGCAATGAAATACCCAATCTCCTAACTTATCAGCTACAACTGCGAGACGAACCTTTTCATCCGGAAGTATTAGATATGTA

>B488_13120|1424207..1424108|Liberibacter_crescens_BT-1_EZ-Tn5_Transposon_Disruption

GCGTATCACCGCCCTTGGCCAGACTGTCACGCTACAGGCGCTGAGCGCCAACGGCGCTGCGCTGTTGCTGCTATTGGATGCCGCCTTACCCGAAGAGGTC

>Intergenic_Region|329143..329044|Liberibacter_crescens_BT-1_EZ-Tn5_Transposon_Disruption

CTAGTACTCCTCGGTATTCGGAGTTTGGTTAGGAGCAGTAAGGCGGTGAGCCCCCATAGCCTATCCAGTGCTCTACCCCCGAGGGTATTCAGTTAACGCT

>B488_11810|1287974..1288073|Liberibacter_crescens_BT-1_EZ-Tn5_Transposon_Disruption

ACTCAGGAGTCATTAATAAACTTTTTTGATCATCAACAACTTCATATTCCATGTTTAAATCATTTTTTTTAAAGAAAACTTTGCGGATCTATATCAAACT

>B488_13680|1494070..1493971|Liberibacter_crescens_BT-1_EZ-Tn5_Transposon_Disruption

GAGCATAATAGCCTAACATAATTGTTTGCAATGCAAGAAACCCCCAAGAACAACCAGCATCAAAAGAATGACTATAAATTTTTTCGTTTGAGTGATTTTT

>Intergenic_Region|525440..525360|Liberibacter_crescens_BT-1_EZ-Tn5_Transposon_Disruption

GCTATGCACTGGGTCAAAACAGCTCCTGCCGAAACAAGGATAGAGTTCAGGAAACCCTTGCGCAGTCTGCCGCAGAACAATAATACGTGGGGTGTGATCA

>B488_02050|240590..240491|Liberibacter_crescens_BT-1_EZ-Tn5_Transposon_Disruption

GTACGACTCCGGCCAGTATTATTATTAAAACTTGCCTTATCATCAATCTGTATATTCACAGTTAGAATATCACCAACATTAATAGCGCGTGCATCTTTAA

>B488_05150|556767..556866|Liberibacter_crescens_BT-1_EZ-Tn5_Transposon_Disruption

TTCCTGATGATGATCAGCAGCATCGCTGGCTTCCTCTTCTGGGAGGTGTCCGCTATCGCATACCCCGTTTACGCGGAAGAAATGCAGCGGAGAATATTTA

>B488_08580|940765..940863|Liberibacter_crescens_BT-1_EZ-Tn5_Transposon_Disruption

GCCTATAGAAGACATTCTCAGTATTTAAAGTGTTCTTTTCAAATGAAACATCAGAAAGCTGTGACAAGGTATTTTCTAGCTCTGTATAAAAACGACCCTG

>B488_05210|563241..563145|Liberibacter_crescens_BT-1_EZ-Tn5_Transposon_Disruption

GCTTCATACTGACCTTCATTGACAAGATGCAAAAGGGTAGAGCCATAAAAAGCAGAACAGCCAAGATTCGTAGACAAAGCTCACCAGAGCATCGAACTCG

>B488_10610|1156014..1156113|Liberibacter_crescens_BT-1_EZ-Tn5_Transposon_Disruption

AATTTGTACTTGATTAATATTTTCCACACCTTCAGCAATGCAATCCAATCCCATATCCTTACATAACAAGCTAAGGAATTTTACTATTTTATAGCTATTG

>Intergenic_Region|1256260..1256161|Liberibacter_crescens_BT-1_EZ-Tn5_Transposon_Disruption

GTATATGACTTGATCGTTTTAATAATCTTCTGCCTGCTACTTCCTGTTTATAAAGTTTCTGATCAAAAAAGGAAGGAGTTTAAAACTAACATAAAGCTTT

>B488_10610|1156906..1157005|Liberibacter_crescens_BT-1_EZ-Tn5_Transposon_Disruption

TCTTGATTCATTTTTCTGGAAATAATAATTCCGAATTCATCACCCCCCAGCCTATAAATAGCTGAACATTTTCCTACAGTCTCTTCAAGACGTTTAGCTG

>B488_13490|1470165..1470066|Liberibacter_crescens_BT-1_EZ-Tn5_Transposon_Disruption

TACCTCTACCATCTAGATTAACGTAAACAGAGATTGTGCTCTCAGTCGTTTTTCGGACAATCTCTGCTATACGGCAGCTTGTTTTTTTTTCCATAAATAA

>Intergenic_Region|329143..329044|Liberibacter_crescens_BT-1_EZ-Tn5_Transposon_Disruption

CTAGTACTCCTCGGTATTCGGAGTTTGGTTAGGAGCAGTAAGGCGGTGAGCCCCCATAGCCTATCCAGTGCTCTACCCCCGAGGGTATTCAGTTAACGCT

>B488_11280|1228494..1228395|Liberibacter_crescens_BT-1_EZ-Tn5_Transposon_Disruption

GTGCACTTTAAGTATTATATTTATTTTATTGATGCCACTATTTTCTTGGGCAGTTGAAGAACAATCTCATATTGTTGAATACGATGTAAGATTTTCTAGT

>Intergenic_Region|489570..489669|Liberibacter_crescens_BT-1_EZ-Tn5_Transposon_Disruption

ATCTTTAAGTATTGTTATCTTTTTTATAACATGACGATAATATGTAGTGATTGGTTATTGTTATTATTTTTTATGGAGTAAGAAGCAATTTTCTAATATC

>B488_11150|1214295..1214196|Liberibacter_crescens_BT-1_EZ-Tn5_Transposon_Disruption

CGCTAAAACTCGTACTGCTTTTGTCCGACCTTGGAAAACAAGATATCACGCAACTGTGCTAGTAAAACATTTAGAAGTCGCATCAAATTCGTAGGCTGCT

>B488_05540|600839..600740|Liberibacter_crescens_BT-1_EZ-Tn5_Transposon_Disruption

CAGGAAGAGATTGCTAAGACAAACCGGGATAAAGAAGAATAAGAGAGCAACTGTCATTGCGGGAAGTAATATTATATATAGGCAGACAAAACACATTAGT

>B488_05440|588306..588248|Liberibacter_crescens_BT-1_EZ-Tn5_Transposon_Disruption

CTCTTCCTGAATACCAATCGATGGGGCACAATTGTTTATTATACGCTCTCTGACTCCCCCCCCATTCCCCCGACCAGCCCCCCCCCCAGCCGCTTCCACG

>Intergenic_Region|329143..329044|Liberibacter_crescens_BT-1_EZ-Tn5_Transposon_Disruption

CTAGTACTCCTCGGTATTCGGAGTTTGGTTAGGAGCAGTAAGGCGGTGAGCCGCCATAGCCTATCCAGTGCTCTACCCCCGAGGGTATTCAGTTAACGCT

>B488_00200|29544..29643|Liberibacter_crescens_BT-1_EZ-Tn5_Transposon_Disruption

CAATATACACATGCTGCTGCGTGGGTAGTTATGGCTCTTATTGAGCTTGGTCGGTATGATGAGGCTTGGAAATGTTTTGCAATGTTAATGCCAATAAATC

>Intergenic_Region|329143..329045|Liberibacter_crescens_BT-1_EZ-Tn5_Transposon_Disruption

CTAGTACTCCTCGGTATTCGGAGTTTGGTTAGGAGCAGTAAGGCGGTGAGCCGCCATAGCCTATCCAGTAGCTCTACCCCCGAGGGTATTCAGTTAACGC

>B488_05410|585359..585260|Liberibacter_crescens_BT-1_EZ-Tn5_Transposon_Disruption

CCTGAGTACCGACATTACTTTGAGTGGATTCAGGATATGCGTGCATTTGCCTGGGATGGACGCAAGGGGTGGTTTGAAAAAGGCGATAGCGTCATGTGGA

>B488_01000|116615..116713|Liberibacter_crescens_BT-1_EZ-Tn5_Transposon_Disruption

CTATACTTCTGACAAGTTACCGATAATACTATTAACATTTTAAAAGATGATATCTCTTTACCTGTTGATATACCTGGAGATATGTTAAGCAATTCTTTAT

>Intergenic_Region|329143..329044|Liberibacter_crescens_BT-1_EZ-Tn5_Transposon_Disruption

CTAGTACTCCTCGGTATTCGGAGTTTGGTTAGGAGCAGTAAGGCGGTGAGCCCCCATAGCCTATCCAGTGCTCTACCCCCGAGGGTATTCAGTTAACGCT

>B488_00200|29544..29643|Liberibacter_crescens_BT-1_EZ-Tn5_Transposon_Disruption

CAATATACACATGCTGCTGCGTGGGTAGTTATGGCTCTTATTGAGCTTGGTCGGTATGATGAGGCTTGGAAATGTTTTGCAATGTTAATGCCAATAAATC

>Intergenic_Region|329143..329044|Liberibacter_crescens_BT-1_EZ-Tn5_Transposon_Disruption

CTAGTACTCCTCGGTATTCGGAGTTTGGTTAGGAGCAGTAAGGCGGTGAGCCCCCATAGCCTATCCAGTGCTCTACCCCCGAGGGTATTCAGTTAACGCT

>Intergenic_Region|330752..330851|Liberibacter_crescens_BT-1_EZ-Tn5_Transposon_Disruption

GGGATGGACGTATCTCTGGTGGACCTGTTGTCCTGCCAAGGGCATAGCAGGGTAGCTACATACGGAATGGATAACCGCTGAAAGCATCTAAGCGGGAAAC

>B488_09520|1024046..1024145|Liberibacter_crescens_BT-1_EZ-Tn5_Transposon_Disruption

GTGCCAAGCCTTCCTCTAAGACAGGAACCAAGGCTTTAAAAACCTTAGTTTCTAAAAAATCACGTAATGATTGTATAATTTCTTTAAGTCCAACAGAAAT

>B488_12080|1320303..1320402|Liberibacter_crescens_BT-1_EZ-Tn5_Transposon_Disruption

TGTAAGTACTACAGAAATTCGATATGGAGACAATGATCGGCTCGCTGCACGCATTGCAACTATGATCAGCGCTGATCTACTTATAATTTTATCAGATGTC

>Intergenic_Region|329143..329044|Liberibacter_crescens_BT-1_EZ-Tn5_Transposon_Disruption

CTAGTACTCCTCGGTATTCGGAGTTTGGTTAGGAGCAGTAAGGCGGTGAGCCCCCATAGCCTATCCAGTGCTCTACCCCCGAGGGTATTCAGTTAACGCT

>B488_00530|67115..67017|Liberibacter_crescens_BT-1_EZ-Tn5_Transposon_Disruption

AGCCATGATGGATCAAATCGACCTTCAGTTAATAAACGATTCCGATCACGCATTAATTCTTTCAAAATTAATGATATAGCTTCCATGTCTTGGATCAAAA

>B488_03280|377804..377705|Liberibacter_crescens_BT-1_EZ-Tn5_Transposon_Disruption

ACTATGGACTGAAAAAGAACCCTTAGGATCAATTGTAATCGGATTAAGACAAAAAAATAACCAACAAGTTCGACAAGTTGCTGCCCAAATGCATTTGTAT

>B488_13420|1459557..1459642|Liberibacter_crescens_BT-1_EZ-Tn5_Transposon_Disruption

CTATAACACCAGTCAAGTCTACGAAATTAGTCAAAGATTATAATCTTAGCAGAAAAGCAGCTTCGGCTAGCAATTTCATATGATCCTTCAACTCAGCAAA

>Intergenic_Region|329143..329044|Liberibacter_crescens_BT-1_EZ-Tn5_Transposon_Disruption

CTAGTACTCCTCGGTATTCGGAGTTTGGTTAGGAGCAGTAAGGCGGTGAGCCCCCATAGCCTATCCAGTGCTCTACCCCCGAGGGTATTCAGTTAACGCT

>B488_05550|595605..595506|Liberibacter_crescens_BT-1_EZ-Tn5_Transposon_Disruption

GTATATGGGTTTTATCAATTTGATTCTGATATCCGCTCCATCATAAAGAAAGCGGTTCAGGAGAAACCGAGCCGTATCAAGATGGATTACGAAAGAATAA

>Intergenic_Region|329143..329044|Liberibacter_crescens_BT-1_EZ-Tn5_Transposon_Disruption

CTAGTACTCCTCGGTATTCGGAGTTTGGTTAGGAGCAGTAAGGCGGTGAGCCCCCATAGCCTATCCAGTGCTCTACCCCCGAGGGTATTCAGTTAACGCT

>Intergenic_Region|329143..329044|Liberibacter_crescens_BT-1_EZ-Tn5_Transposon_Disruption

CTAGTACTCCTCGGTATTCGGAGTTTGGTTAGGAGCAGTAAGGCGGTGAGCCCCCATAGCCTATCCAGTGCTCTACCCCCGAGGGTATTCAGTTAACGCT

>Intergenic_Region|1163598..1163697|Liberibacter_crescens_BT-1_EZ-Tn5_Transposon_Disruption

GTACCATACATAAAAACATGCTGGATTTATCTGTATGTCCTTGATATAGGCTAAAGGTTGACTTTTAATTATCAAATATCTGTAACCATGAGCAATAATG

>B488_12300|1345941..1345842|Liberibacter_crescens_BT-1_EZ-Tn5_Transposon_Disruption

GGATTACGGTGGTCAGATCGAGCAGAAGATTATCGTACTGAAGCGTTAGGTCTGGTAAAGGCACAAAGAGTTAAGAATGCAGTGATTGTTCCTGTGGGAG

>Intergenic_Region|329123..329046|Liberibacter_crescens_BT-1_EZ-Tn5_Transposon_Disruption

CTAGTCCTCCTTACCTATTTATGAGTTTGGTTAGGAGCAGTAAGGCGGTGAGCCCCCATAGCCTATCCTTTGCTCTACCCCCGAGGGTATTCAGTTAACG

>Intergenic_Region|329143..329044|Liberibacter_crescens_BT-1_EZ-Tn5_Transposon_Disruption

CTAGTACTCCTCGGTATTCGGAGTTTGGTTAGGAGCAGTAAGGCGGTGAGCCCCCATAGCCTATCCAGTGCTCTACCCCCGAGGGTATTCAGTTAACGCT

>B488_13630|1484247..1484148|Liberibacter_crescens_BT-1_EZ-Tn5_Transposon_Disruption

GATTTGATCAATGGAAGAATAGTTGATGTTGTTCCTATAATTTCTAAAAACTCTGAGCGTAAAGCTTGTTTTACCTGTTCTGTTGGGACATAAATTGTAA

>Intergenic_Region|235912..235813|Liberibacter_crescens_BT-1_EZ-Tn5_Transposon_Disruption

CTTCGAATCCGAAGGTCGCAGGTTCGAATCCTGCCGAGTGCGCCATGTAACTCATACCCCATTGATTTTACTGAGTTTTTTCTTGGATTTGGTTGCACCT

>Intergenic_Region|329143..329043|Liberibacter_crescens_BT-1_EZ-Tn5_Transposon_Disruption

CTAGTACTCCTCCTTCTTAGGAGTTTGGTTGGAGCAGTAAGGCGGTGAGCCCCCATAGCCTATCCCGTGCTCTACCCCCGAGGGTATTCAGTTAACGCTC

>B488_09470|1019120..1019219|Liberibacter_crescens_BT-1_EZ-Tn5_Transposon_Disruption

GGAAATACTTCCCCTTTTATCTCCGGATCACGTCCATCAGAAAATACACGAATTGGCTCTACATCATTAGAGTCAGTAATTTTTTTAACTCGACGGCGAA

>Intergenic_Region|330202..330103|Liberibacter_crescens_BT-1_EZ-Tn5_Transposon_Disruption

ATCCTACACATACCTACACAAATGCCAGTGTAAAGTTATAGTAAAGGTGCACGGGGTCTTTCCGTCTGACCGCAGGAACCCCGCATCTTCACGGGGAATT

>Intergenic_Region|330202..330103|Liberibacter_crescens_BT-1_EZ-Tn5_Transposon_Disruption

ATCCTACACATACCTACACAAATGCCAGTGTAAAGTTATAGTAAAGGTGCACGGGGTCTTTCCGTCTGACCGCAGGAACCCCGCATCTTCACGGGGAATT

>B488_05140|554929..555030|Liberibacter_crescens_BT-1_EZ-Tn5_Transposon_Disruption

CACCTGCACTCTTTCCTGCGCCTGTAAGGTCTGGTTACTGTAGAGGATTTTCAGGATGGCACACCGGGATCGGCTCGGGTTCTCAAAAACTTCTGGCCAA

>Intergenic_Region|418388..418289|Liberibacter_crescens_BT-1_EZ-Tn5_Transposon_Disruption

GGTTACAGACTTACCCATTATAGATCCTTGCCTATATTATTCAACCTTGAAACATCTGAAGAAGAAGTTGCTATACACAGAAACCCTTATAAACTTATTG

>Intergenic_Region|330752..330852|Liberibacter_crescens_BT-1_EZ-Tn5_Transposon_Disruption

GGGATGGACGTATCTCTGGTGGACCTGTTGTCCTGCCTGGGCATAACAAGGTAGCTACATACGGAATGGATAACCGCTGAAAGCATCTAAACGGGAAACC

>B488_11490|1246874..1246770|Liberibacter_crescens_BT-1_EZ-Tn5_Transposon_Disruption

ATGTTGGTCTAGCAATTAAAGATCAGAATTTGCCAGCTGTAGGGTACCTCAGTCAAGAACGTCTTAGTAATATTCACATGATCAGTCTTATATCGACCTA

>B488_08280|904591..904689|Liberibacter_crescens_BT-1_EZ-Tn5_Transposon_Disruption

AGCCCATTCAATACAGCAGCGGCAACAGTAATCCAAGGTTCCGTTATGAGCCATATGGAAAACAGGTATATTCATTTTTTCACTTAAGATGCGTTCAACC

>B488_12790|1394156..1394057|Liberibacter_crescens_BT-1_EZ-Tn5_Transposon_Disruption

GTTTGGCCTGATGCTCTTGATTTGATGTTGATATGTGTTGAATCTGGTCTCGCACTAGATCATAGTTTACGCCGTGTTGCAGAAGAGATCGCATCACAAT

>B488_12300|1345941..1345842|Liberibacter_crescens_BT-1_EZ-Tn5_Transposon_Disruption

GGATTACGGTGGTCAGATCGAGCAGAAGACTATCGTACTGAAGCGTTAGGTCTGGTAAAGGCACAAAGAGTTAAGAATGCAGTGATTGTTCCTGTGGGAG

>B488_12080|1320303..1320402|Liberibacter_crescens_BT-1_EZ-Tn5_Transposon_Disruption

TGTAAGTACTACAGAAATTCGATATGGAGACAATGATCGGCTCGCTGCACGCATTGCAACTATGATCAGCGCTGATCTACTTATAATTTTATCAGATGTC

>Intergenic_Region|329143..329044|Liberibacter_crescens_BT-1_EZ-Tn5_Transposon_Disruption

CTAGTACTCCTCGGTATTCGGAGTTTGGTTAGGAGCAGTAAGGCGGTGAGCCCCCATAGCCTATCCAGTGCTCTACCCCCGAGGGTATTCAGTTAACGCT

>B488_01000|116614..116713|Liberibacter_crescens_BT-1_EZ-Tn5_Transposon_Disruption

ATATACTTCTAAAGAAGTTACCGCTTATACTATTAACATTTTAAAAGATGATATCTCTTTAGCTGTTGATATACTTGGAGATATGTTAAGTAATTCTTTA

>B488_04420|496323..496224|Liberibacter_crescens_BT-1_EZ-Tn5_Transposon_Disruption

GGACCAATCATTAATAATTCTCCTACAATATCTGATTCAACAACGATTGCATTCATCACTCCATTGACCTGAGCAATCATGGAATCACGTTTCACTAGAA

>B488_12260|1340131..1340032|Liberibacter_crescens_BT-1_EZ-Tn5_Transposon_Disruption

ATGCAGTAGAATCAGGTATCGAAACCAATCAGATAATCATGCCAGTCACCAAAACCATTGTATTGGGAGGGGGATGCTTTTGGGGTATGGAAGCTGTGTT

>Intergenic_Region|330816..330915|Liberibacter_crescens_BT-1_EZ-Tn5_Transposon_Disruption

GAATGGATAACCGCTGAAAGCATCTAAGCGGGAAACCAACCTGAAAACGAGTATTCCCTATCAGAGCCGTGGAAGACTACCACGTTGATAGGCTGGGTGT

>B488_00350|49159..49060|Liberibacter_crescens_BT-1_EZ-Tn5_Transposon_Disruption

CTCTAATTCTTAGCACAACATTATATGAAAACACGTCATGGGGTAAGAGATAAATTAAAATTCTTTCCCTGTTCTTTGATCAACAGATTTCATTGACAAT

>B488_08350|911597..911498|Liberibacter_crescens_BT-1_EZ-Tn5_Transposon_Disruption

CTGTTCACCGTTGTCGAGGACGAGAAGGGGAACAGAGCCTCTGGAATTAATAGTGTAATAGTCTGTGTTGTCTTCAAGTTTATGCGTGCTAAGATCGACT

>B488_04940|543400..543499|Liberibacter_crescens_BT-1_EZ-Tn5_Transposon_Disruption

GAGCAGATGTTGTTGTTTTTGATAGTGTTTATGGCTTGAGTTATAATGGTAATAATTCTGAAATTCTTCTTGCTGAATTTAAAAAACCAGGACGTACTAA

>B488_12300|1345941..1345842|Liberibacter_crescens_BT-1_EZ-Tn5_Transposon_Disruption

GGATTACGGTGGTCAGATCGAGCAGAAGATTATCGTACTGAAGCGTTAGGTCTGGTAAAGGCACAAAGAGTTAAGAATGCAGTGATTGTTCCTGCGGGAG

>B488_13640|1488735..1488635|Liberibacter_crescens_BT-1_EZ-Tn5_Transposon_Disruption

CTCCATACGGTTAGAGTGATGTATAATATCTTTGCTTTCTGTGGCTAATACAAGGGGTGTTATGAGCCATCGAAAGACTTTATCAACAGCACTCAGTATA

>Intergenic_Region|329143..329045|Liberibacter_crescens_BT-1_EZ-Tn5_Transposon_Disruption

CTAGTACTCCTCGGTATTCGGAGTTTGGTTAGGAGCAGTAAGGCGGTGAGCCCCCATAGCCTATCCAGTAGCTCTACCCCCGAGGGTATTCAGTTAACGC

>B488_07310|815778..815867|Liberibacter_crescens_BT-1_EZ-Tn5_Transposon_Disruption

TACCTGATCCAGTAAACGTTTACATTCCAATAAATCGTAGATTGCTTCCTGCAACAGTCTACGATTCTCATCACTTAAATAATGATGATCCTTCAACTCA

>B488_12080|1320303..1320402|Liberibacter_crescens_BT-1_EZ-Tn5_Transposon_Disruption

TGTAAGTACTACAGAAATTCGATATGGAGACAATGATCGGCTCGCTGCACGCTTTGCAACTATGATCAACGCTGATCTACTTATAATTTTATCATATGTC

>Intergenic_Region|329143..329045|Liberibacter_crescens_BT-1_EZ-Tn5_Transposon_Disruption

CTACTACTCCTCGTATATTCGGAGTTTGGTTAGGAGCAGTAAGGCGGTGAGCCCCCATAGCCTATCCAGTGCTCTACCCCCGAGGGTATTCAGTTAACGC

>B488_00350|49159..49060|Liberibacter_crescens_BT-1_EZ-Tn5_Transposon_Disruption

CTCTAATTCTTAGCACAACATTATATGAAAACACGTCATGGGGTAAGAGATAAATTAAAATTCTTTCCCTGTTCTTTGATCAACAGATTTCATTGACAAT

>B488_01760|213646..213744|Liberibacter_crescens_BT-1_EZ-Tn5_Transposon_Disruption

AATCAGTGATGGCTTCAGAAATTCGGATAATGCCAAAGCCTCTAAAGCCATCAAATTTACGGTCGCGCGTAGTATGTTGGAAGAGCAGCAAAATCTATCG

>B488_02390|273621..273551|Liberibacter_crescens_BT-1_EZ-Tn5_Transposon_Disruption

ACTAATACAGGTTGAATTTTATGACCAATCATGAATATCCTTCTCCAATTATCTTAACTCAAGGTGATCCTTCAACTCAGCAAAAGTTAAGTAACTCTCA

>B488_08210|893334..893433|Liberibacter_crescens_BT-1_EZ-Tn5_Transposon_Disruption

GATAAGGAACTGGCTAAAGGCTCTGGTGTTTCTCAGGTTACAGATTTTTTTAACATCAATGCCTACGGACAAGAAAAAAATGGCAAAGAGATACAATCCG

>Intergenic_Region|330752..330851|Liberibacter_crescens_BT-1_EZ-Tn5_Transposon_Disruption

GGGATGGACGTATCTCTGGTGGACCTGTTGTCCTGCCAAGGGCATATCAGGGTACCTACATACGGAATGGATAACCGCTGAAAGCATCTAAACGGGAAAC

>B488_04760|975656..975558|Liberibacter_crescens_BT-1_EZ-Tn5_Transposon_Disruption

ACAGCGATAAGTGCGCCAAACAGAATACGAAAATCTCTATCCGCTGATTTTCGTAATTCAGATACCTCAGTATGTATCTTAGTCATTCAGACGGTTTTCG

>B488_11480|1244882..1244784|Liberibacter_crescens_BT-1_EZ-Tn5_Transposon_Disruption

TATCGAACCTATTATTTCTGTTCCGTATAATGGTCATGCAGCTCTTCCAGAGCTAGCAAATAATTTACAACTTGAAGCGGATGAGCTTTTCCATAATGGT

>Intergenic_Region|329143..329044|Liberibacter_crescens_BT-1_EZ-Tn5_Transposon_Disruption

CTAGTACTCCTCGGTATTCGGAGTTTGGTTAGGAGCAGTAAGGCGGTGAGCCCCCATAGCCTATCCAGTGCTCTACCCCCGAGGGTATTCAGTTAACGCT

>B488_01290|159178..159274|Liberibacter_crescens_BT-1_EZ-Tn5_Transposon_Disruption

TTATGACAGTGGTTGGTCGTTTGATAAAGGCAGCAGATTTTTTACTTGTAGTTATTACTGCATTCATACGAGATATGAAATATTTTGTCCGTTTTGTGTG

>Intergenic_Region|329073..329171|Liberibacter_crescens_BT-1_EZ-Tn5_Transposon_Disruption

GCACTGGATAGGTATATGGGGGCTCACCGCCTTACTGCTCCTAACCAAACTCCGAATACCGAGGAGTACTAGTTGGCAGACACACGGCGGGAGCTAACGT

>B488_03730|428423..428522|Liberibacter_crescens_BT-1_EZ-Tn5_Transposon_Disruption

TCTTTTATATGTTGCATCTCATTCGGATTTGATAACAGATGTTTTGAAGAGAGGTCTTTCTGATAAACAAGATATTCTTGGTTATGCTGTTTGGCATTAT

>B488_05050|547658..547558|Liberibacter_crescens_BT-1_EZ-Tn5_Transposon_Disruption

CTTCTCAATCAGACGTTTCGCCGATTGTTCAAGTGACTTCTGAACCTCGCGGACACAGACAGACAACAGGCCTAGATGCTGAAGATGATGCAAAAGCAAA

>Intergenic_Region|329143..329044|Liberibacter_crescens_BT-1_EZ-Tn5_Transposon_Disruption

CTAGTACTCCTCGGTATTCGGAGTTTGGTTAGGAGCAGTAAGGCGGTGAGCCCCCATAGCCTATCCAGTGCTCTACCCCCGAGGGTATTCAGTTAACGCT

>B488_05440|588135..588036|Liberibacter_crescens_BT-1_EZ-Tn5_Transposon_Disruption

CTCCTACACTACACGGATCTAAAGGACGATGATAGAGAACGGTTACCTACCATTTCATCCTGGGAAATATGCCGCCCTATCATAAATGTTTTAAGAAACC

>B488_07810|858926..859025|Liberibacter_crescens_BT-1_EZ-Tn5_Transposon_Disruption

CATCCTGACCATCTCCCAGAGATACAGAGACATGATCATCCTCTTCTGCTGTTTCCTGTTCTTCGCCTTGCTGCTGAAGACTTTCCTGAAGCCTTGTTTT

>Intergenic_Region|583497..583398|Liberibacter_crescens_BT-1_EZ-Tn5_Transposon_Disruption

AGATTAGCTTTTTATTTTAAAACGCATCTCGAAAGAACACTTTATCCGAACAAATTTATTCCGCATTAACATTATTTAATGCAGAATAAATTGTAATTGT

>B488_06570|726665..726566|Liberibacter_crescens_BT-1_EZ-Tn5_Transposon_Disruption

GAAGAAGTACAAGCAATTATAAATGAAATTGAATGTCTGCGCTATAAGGAGCATCCCTTGAGTCGTATGGCAATCCTCGTGCGTGCTTCTTTTCAAATGC

>B488_07910|868170..868269|Liberibacter_crescens_BT-1_EZ-Tn5_Transposon_Disruption

CTTCCTGCATGTGCCGGGAAACCATGACTCTCCCCCATTGATGAATAACCCGACGGGCGGCCTCTATGGCCAGCGGCCCGGCGGATACAGTCGATGACAC

>Intergenic_Region|527694..527595|Liberibacter_crescens_BT-1_EZ-Tn5_Transposon_Disruption

GTTCAGGTGATCACCCCGAGGCACGAAGACGGGCAACCAGATCTGCCTTGGGAAACGAAACAGGGAAAGAGACACGGAAATGACAGAGGTTACAAATCCC

>Intergenic_Region|418388..418290|Liberibacter_crescens_BT-1_EZ-Tn5_Transposon_Disruption

GGTTACAGACTTACCCATTATAGATCCTTGCCTATATTATTCAACCTTGAAACATCTGAAGAAGAAGTTGCTATACACAGAAACCCTTATAAACTTATTA

>B488_05460|590573..590672|Liberibacter_crescens_BT-1_EZ-Tn5_Transposon_Disruption

GGCTGAGGTCGGGACAATATCTGGGTTTTTCGGAAATGATTGGTCAGCTCGATCAAAGTGTCAGGTGCCAGAATATCAAGATGTTCATTTGCCCAGGCAG

>Intergenic_Region|329143..329044|Liberibacter_crescens_BT-1_EZ-Tn5_Transposon_Disruption

CTAGTACTCCTCGGTATTCGGAGTTTGGTTAGGAGCAGTAAGGCGGTGAGCCCCCATAGCCTATCCAGTGCTCTACCCCCGAGGGTATTCAGTTAACGCT

>Intergenic_Region|813767..813668|Liberibacter_crescens_BT-1_EZ-Tn5_Transposon_Disruption

GTTGTAGAGAGTTTTCTGGCTTCTTACAATTCTTTTTGATATATTATGGGATCATGTTTTTAACATAAAAATAAATTCACCATTAAATCTTCTTTGTATC

>B488_00030|4380..4478|Liberibacter_crescens_BT-1_EZ-Tn5_Transposon_Disruption

GTACTTGTGTCCATTACAATGTGAGTTTTTTGCTTTAGAAGGTCTTAGTCAGCTTTTAGAAACAGTTAATGAAGTTCGAGATACAGTCAAAACAAACCTT

>B488_10820|1180098..1179999|Liberibacter_crescens_BT-1_EZ-Tn5_Transposon_Disruption

CTTGATAACAGATATGAATGGCCACCATAGTCGCTATGATAGGATCAACTATAGTGTAGCCGGTTACCATAACTATAATCAACCCGAAAACTATACTTAT

>B488_05550|594367..594268|Liberibacter_crescens_BT-1_EZ-Tn5_Transposon_Disruption

CTCTGAGCCAGCGGTCACTGATGATGCTACCATTGCCAGCAGTCCTGATACAGAAGAAATTCCCCCGGCGCCAGCCCCTCAGCACCTGCATTATCCCTAT

>Intergenic_Region|329143..329044|Liberibacter_crescens_BT-1_EZ-Tn5_Transposon_Disruption

CTAGTACTCCTCGGTATTCGGAGTTTGGTTAGGAGCAGTAAGGCGGTGAGCCCCCATAGCCTATCCAGTGCTCTACCCCCGAGGGTATTCAGTTAACGCT

>B488_10240|1120300..1120202|Liberibacter_crescens_BT-1_EZ-Tn5_Transposon_Disruption

CTTAGATATCACAAAAGATAAGCCCCAGATAGTTTCTAGTATTCCTATACCAATAACAAAAGAGCGTTATGCTTCGGGCTGCATTATCCCAAGATGAGAA

>B488_10610|1155954..1156053|Liberibacter_crescens_BT-1_EZ-Tn5_Transposon_Disruption

ACCCAACATAGGCTTCGCAAAATAATGCCCCTGAAATAAGTTATACCCTATTTCTGATAGAATTTGTACTTGATTAATATTTTCCACACCTTCAGCAATG

>Intergenic_Region|329143..329045|Liberibacter_crescens_BT-1_EZ-Tn5_Transposon_Disruption

CTAGTACTCCTCGGTATTCGGAGTTTGGTTAGGAGCAGTAAGGCGGTGAGCCCCCATAGCCTATCCAGTAGCTCTACCCCCGAGGGTATTCAGTTAACGC

>B488_13420|1458601..1458502|Liberibacter_crescens_BT-1_EZ-Tn5_Transposon_Disruption

CCTTTGCACCGTTGAAAAATAATGATTGCCTATAGTCCAACAACAGGAATTTAAAAGTTTCTGATCCACCGGAGAGTCGGTTTAGAACTCTAATCAAATT

>Intergenic_Region|648293..648393|Liberibacter_crescens_BT-1_EZ-Tn5_Transposon_Disruption

GTGTTAAGAAATAAAAGGAATATATCTAGCTTAAGAAAGAAATAGATTTTGAGAGAAAGTGTATTGGTGGATATTATTTTTTCTTTTTATCTCCCTTGTT

>Intergenic_Region|329143..329044|Liberibacter_crescens_BT-1_EZ-Tn5_Transposon_Disruption

CTAGTACTCCTCGGTATTCGGAGTTTGGTTAGGAGCAGTAAGGCGGTGAGCCCCCATAGCCTATCCAGTGCTCTACCCCCGAGGGTATTCAGTTAACGCT

>B488_06540|723399..723498|Liberibacter_crescens_BT-1_EZ-Tn5_Transposon_Disruption

CTGAAGAGCTATCTGCTTTTTATAATGTAAAGCGCAACAATCATCTCACGACTGATTGGAGACGAAAGAAAAGTGTGATGCAAGAGAAGTTAGTCGAAGC

>B488_12830|1398519..1398603|Liberibacter_crescens_BT-1_EZ-Tn5_Transposon_Disruption

ACTGACAAAAAATGACGACCCGGATGAAGTTGAAGACCCGGATGTCCTTCGCGTATCTCAAGAAGTATTCTGACGATAAGTTTGCATAGTATTATTAAGC

>B488_11490|1246874..1246775|Liberibacter_crescens_BT-1_EZ-Tn5_Transposon_Disruption

ATGTTGGTCTAGCAATTAAAGATCAGAATTTGCCAGCTGTAGGGTAAGCTTCGTTAACAACGTTGTTAGTAATTCTTCCCTATGATCAGTCGTTATCTCG

>B488_03660|419260..419161|Liberibacter_crescens_BT-1_EZ-Tn5_Transposon_Disruption

TCGTAAGTCCATTCATCTTCAGGACAAACACCTTTCTTGTGTAACGTCTTAATACCACTCAGCATTGATGAACCAGCATCACTGGCTACATTGTTCTCAA

>B488_02290|263341..263440|Liberibacter_crescens_BT-1_EZ-Tn5_Transposon_Disruption

ATGCTGCCTGATGTTCTTTCAGCTTCTCTTATAGATCGTGCTTCTAAAATTGTTCATCTTGCCTTGAAATCTGGTGCTGATGCTGCTGATGTAAAGGTTC

>Intergenic_Region|329143..329045|Liberibacter_crescens_BT-1_EZ-Tn5_Transposon_Disruption

CTAGTACTCCTCGGTATTCGGAGTTTGGTTAGGAGCAGTAAGGCGGTGAGCCCCCATAGCCTATCCAGTAGCTCTACCCCCGAGGGTATTCAGTTAACGC

>Intergenic_Region|329143..329045|Liberibacter_crescens_BT-1_EZ-Tn5_Transposon_Disruption

CTAGTACTCCTCGGTATTCGGAGTTTGGTTAGGAGCAGTAAGGCGGTGAGCCCCCATAGCCTATCCAGTAGCTCTACCCCCGAGGGTATTCAGTTAACGC

>B488_13270|1440904..1441003|Liberibacter_crescens_BT-1_EZ-Tn5_Transposon_Disruption

TTCAATGTTAATACTATACCCTTTCCCTCTGCAGGACACGAACAAGATTCTCTTGATGCGCTTGAGAAGTTTTGTTGTAATGGCACAGCTGCAGCTTTTT

>Intergenic_Region|329143..329044|Liberibacter_crescens_BT-1_EZ-Tn5_Transposon_Disruption

CTAGTACTCCTCGGTATTCGGAGTTTGGTTAGGAGCAGTAAGGCGGTGAGCCCCCATAGCCTATCCAGTGCTCTACCCCCGAGGGTATTCAGTTAACGCT

>B488_08070|879670..879766|Liberibacter_crescens_BT-1_EZ-Tn5_Transposon_Disruption

GAAATATTCCGTATGAATCTCCGACAAGATCTTAATGAACTTATTGATAATAAAGTTGATTCGCTTGTTAATAAAATGGACACCAAGTTTGATCCTTCAA

>B488_13150|1429042..1428943|Liberibacter_crescens_BT-1_EZ-Tn5_Transposon_Disruption

GTGTAATGTTAATTTTAATTCTTTAGAACCAGAAGTTCTTAAAAATGTTGATAAAAAAACGCAATGTATTAAAGATATAGCTTTAAGAAAGGCATTAATG

>Intergenic_Region|329143..329044|Liberibacter_crescens_BT-1_EZ-Tn5_Transposon_Disruption

CTAGTACTCCTCGGTATTCGGAGTTTGGTTAGGAGCAGTAAGGCGGTGAGCCCCCATAGCCTATCCAGTGCTCTACCCCCGAGGGTATTCAGTTAACGCT

>B488_08140|884085..884184|Liberibacter_crescens_BT-1_EZ-Tn5_Transposon_Disruption

TCTGGGAGTAGGAAAGATTTTACGTCAATACGGTGCTGGTTCTGTGAGTGAATGCGACTGGTGGGATGAGGTGTCAATGATGGGATTGAAACTACATTGT

>Intergenic_Region|329143..329045|Liberibacter_crescens_BT-1_EZ-Tn5_Transposon_Disruption

CTAGTACTCCTCGGTATTCGGAGTTTGGTTAGGAGCAGTAAGGCGGTGAGCCCCCATAGCCTATCCAGTAGCTCTACCCCCGAGGGTATTCAGTTAACGC

>B488_05550|596056..595956|Liberibacter_crescens_BT-1_EZ-Tn5_Transposon_Disruption

TTACGGCCCTGCTTCAAAGCAGAAGAAGCCTGGACAGCCTATCATCTCAGCAAAGTACTCGGATCCGAAAGCTGGGCGGGAAAAGATGGGTTCCGGGGTG

>Intergenic_Region|531715..531814|Liberibacter_crescens_BT-1_EZ-Tn5_Transposon_Disruption

GTTGAGAAACGGTTAGTTGATAAAATCTACAACGTTTCATATCTTATATTTGGCTTACTGATTGCAAGAATCGTTGGGTTGTCTGGATTAATGACCAAAG

>Intergenic_Region|418388..418289|Liberibacter_crescens_BT-1_EZ-Tn5_Transposon_Disruption

GGTTACAGACTTACCCATTATAGATCCTTGCCTATATTATTCAACCTTGAAACATCTGAAGAAGAAGTTGCTATACACAGAAACCCTTATAAACTTGTTG

>B488_06110|674852..674753|Liberibacter_crescens_BT-1_EZ-Tn5_Transposon_Disruption

CATTGGCACAGCGTCCAGAACTATTATTGCTAGACGAACCAACAAACCATCTGGATCCCGCTAGACGCAAGGAACTACTGACATTGGTCCAACAACGCGG

>Intergenic_Region|329143..329044|Liberibacter_crescens_BT-1_EZ-Tn5_Transposon_Disruption

CTAGTACTCCTCGGTATTCGGAGTTTGGTTAGGAGCAGTAAGGCGGTGAGCCCCCATAGCCTATCCAGTGCTCTACCCCCGAGGGTATTCAGTTAACGCT

>B488_11490|1246874..1246775|Liberibacter_crescens_BT-1_EZ-Tn5_Transposon_Disruption

ATGTTGGTCTATAAATTAAAGATCTCATTTTGCCTGCTGTAGGGTATGCTTTGTTAACAATGTTGTTAGTAATTATTTTTTATGATCAGTTGTTATTTCG

>B488_07810|859042..859141|Liberibacter_crescens_BT-1_EZ-Tn5_Transposon_Disruption

TCACAATCCTGTGCTGATGGAGTGTTTTCATCTGCATTCCCTGTAAACCAGGAAGAAGGACAATCGTCATTTTTAATAACACGATCAGCATCATCATGAG

>B488_11990|1309167..1309069|Liberibacter_crescens_BT-1_EZ-Tn5_Transposon_Disruption

GTTCCACTTCCAGAGCAGGGAGCATCAATGAGTACGGTGTCGAAATGTTCTTCGTAAAGGCAGAAGCGTTGATAATCTGCTATGGACCTGAACATTGTGT

>Intergenic_Region|329143..329044|Liberibacter_crescens_BT-1_EZ-Tn5_Transposon_Disruption

CTAGTACTCCTCGGTATTCGGAGTTTGGTTAGGAGCAGTAAGGCGGTGAGCCCCCATAGCCTATCCAGTGCTCTACCCCCGAGGGTATTCAGTTAACGCT

>B488_06660|738834..738933|Liberibacter_crescens_BT-1_EZ-Tn5_Transposon_Disruption

GAACCAGCCAGGCCAGGAACAGAGGTAATGGCTCCACCAGTCGCTAAGGGAAGCAAAAAACCATTGCCCAAGGTGAGAAGCGAAACAGCACTAATACAGG

>Intergenic_Region|329143..329044|Liberibacter_crescens_BT-1_EZ-Tn5_Transposon_Disruption

CTAGTACTCCTCGGTATTCGGAGTTTGGTTAGGAGCAGTAAGGCGGTGAGCCCCCATAGCCTATCCAGTGCTCTACCCCCGAGGGTATTCAGTTAACGCT

>B488_05120|553914..553815|Liberibacter_crescens_BT-1_EZ-Tn5_Transposon_Disruption

TCCCTGATCTGAATGATCTCAAGACAATCGTTTGGCACTGATACCATACCGTCGACAAGAGCAAGAGAAGTCTGCTTTTCCATTCCTGACAGACGCAAGT

>Intergenic_Region|329143..329044|Liberibacter_crescens_BT-1_EZ-Tn5_Transposon_Disruption

CTAGTACTCCTCGGTATTCGGAGTTTGGTTAGGAGCAGTAAGGCGGTGAGCCCCCATAGCCTATCCAGTGCTCTACCCCCGAGGGTATTCAGTTAACGCT

>Intergenic_Region|329143..329044|Liberibacter_crescens_BT-1_EZ-Tn5_Transposon_Disruption

CTAGTACTCCTCGGTATTCGGAGTTTGGTTAGGAGCAGTAAGGCGGTGAGCCCCCATAGCCTATCCAGTGCTCTACCCCCGAGGGTATTCAGTTAACGCT

>B488_08370|914648..914747|Liberibacter_crescens_BT-1_EZ-Tn5_Transposon_Disruption

GATAAAGCATCATCAATCAGCATAGAACTCATAATTTACCTCCAGAAATAAAAGATAAAATACCCCACATCACTTATAAATCAATTTTTCTCCAACGAAC

>B488_12810|1395859..1395958|Liberibacter_crescens_BT-1_EZ-Tn5_Transposon_Disruption

ATTTCATACCGTATTAAATCTTGAGTAATAATAACGTCACCTTCCATACCAATAATACAAGGGGTATTAAGAGCCATTCGAGAACCATCTCGTAAACGTT

>B488_07810|859042..859141|Liberibacter_crescens_BT-1_EZ-Tn5_Transposon_Disruption

TCACAATCCTGTGCTGATGGAGTGTTTTCATCTGCATTCCCTGTAAACCAGGAAGAAGGACAATCGTCATTTTTAATAACACGATCAGCATCATCATGAG

>B488_13370|1451615..1451516|Liberibacter_crescens_BT-1_EZ-Tn5_Transposon_Disruption

GGCTTACGCTATGATAGGTGGAATAATATCAGGAACATTGTTAACATTAACATTTTTCCCTGCTTTGTATGTGGTGTTGTTTCGTATTAAACCGTTTGAT

>B488_13570|1477768..1477669|Liberibacter_crescens_BT-1_EZ-Tn5_Transposon_Disruption

GACCAGGTCTGTTTCCATTCAAATTTTTTTGATTCAACTAAAGGAAGTTCAGAACGAATAATTTGTTCACGTGAATATGAAAATAGATAACTTGAATCAA

>Intergenic_Region|329143..329044|Liberibacter_crescens_BT-1_EZ-Tn5_Transposon_Disruption

CTAGTACTCCTCGGTATTCGGAGTTTGGTTAGGAGCAGTAAGGCGGTGAGCCCCCATAGCCTATCCAGTGCTCTACCCCCGAGGGTATTCAGTTAACGCT

>B488_04020|466706..466805|Liberibacter_crescens_BT-1_EZ-Tn5_Transposon_Disruption

TACATATTATCTGCAGGAGCAGAATATTATTGATAAATTTGATAGTATCATTAATCTTATCAGTGATCATCATGCTAAACAATTAATAAAAGATCAGATA

>B488_06660|738836..738744|Liberibacter_crescens_BT-1_EZ-Tn5_Transposon_Disruption

GGTTACATTCTGCTTCTGTTTTTATGGGAGCTTTTATTATCGCTTCTTCAAACATGACAACTGCCTATATTGGTCAGTTTTCTCACCATCAACCTGGCAG

>Intergenic_Region|329143..329044|Liberibacter_crescens_BT-1_EZ-Tn5_Transposon_Disruption

CTAGTACTCCTCGGTATTCGGAGCTTGGTTAGGAGCAGTAAGGCGGTGAGCCCCCATAGCCTATCCAGTGCTCTACCCCCGAGGGTATTCAGTTAACGCT

>B488_05550|594057..594156|Liberibacter_crescens_BT-1_EZ-Tn5_Transposon_Disruption

GCTCTGTTATCTTTATGAGATCGGTGACCAGATCTTTTTGGTACTTCTTGTTTGAGCTTTCATAGCGCTCACAATAATACGGATCAAGCTTCTTCATATA

>B488_07810|859042..859141|Liberibacter_crescens_BT-1_EZ-Tn5_Transposon_Disruption

TCACAATCCTGTGCTGATGGAGTGTTTTCATCTGCATTCCCTGTAAACCAGGAAGAAGGACAATCGTCATTTTTAATAACACGATCAGCATCATCATGAG

>Intergenic_Region|329143..329044|Liberibacter_crescens_BT-1_EZ-Tn5_Transposon_Disruption

CTAGTACTCCTCGGTATTCGGAGTTTGGTTAGGAGCAGTAAGGCGGTGAGCCCCCATAGCCTATCCAGTGCTCTACCCCCGAGGGTATTCAGTTAACGCT

>B488_10520|1147176..1147077|Liberibacter_crescens_BT-1_EZ-Tn5_Transposon_Disruption

GCGTGTAAGAGTCCAAAAACAAGAAATGATACGCATTCTTGATGAAATTCAAGATATGCATATGAATACAATTGTACTTCAAGTTAAGCCATCTGCAGAG

>B488_06140|678243..678144|Liberibacter_crescens_BT-1_EZ-Tn5_Transposon_Disruption

CTATTGGCTTAACCTCAACTATTGTTAAGCTTATCAATCGACACGACCCTCATGACCCTATAGCACGTCAGTTTATTCCACAAGAAGCAGAATTAAACAT

>Intergenic_Region|329143..329044|Liberibacter_crescens_BT-1_EZ-Tn5_Transposon_Disruption

CTAGTACTCCTCGGTATTCGGAGTTTGGTTAGGAGCAGTAAGGCGGTGAGCCCCCATAGCCTATCCAGTGCTCTACCCCCGAGGGTATTCAGTTAACGCT

>B488_07740|853367..853466|Liberibacter_crescens_BT-1_EZ-Tn5_Transposon_Disruption

CCTGTAAAAGGATCCCTGATCATTCTCTTTCCCGTCTGGACATAATCCAGGCGTGCATCAGGAGTCACCTGGTTGATATTGCCAATCGCTGAACTGCTGA

>Intergenic_Region|329143..329044|Liberibacter_crescens_BT-1_EZ-Tn5_Transposon_Disruption

CTAGTACTCCTCGGTATTCGGAGTTTGGTTAGGAGCAGTAAGGCGGTGAGCCCCCATAGCCTATCCAGTGCTCTACCCCCGAGGGTATTCAGTTAACGCT

>Intergenic_Region|525440..525360|Liberibacter_crescens_BT-1_EZ-Tn5_Transposon_Disruption

GCTATGCACTGGGTCAAAACAGCTCCTGCCGAAACAAGGATAGAGTTCAGGAAACCCTTGCGCAGTCTGCCGCAGAACAATAATACAAGGGGTGTTATGA

>Intergenic_Region|329143..329044|Liberibacter_crescens_BT-1_EZ-Tn5_Transposon_Disruption

CTAGTACTCCTCGGTATTCGGAGTTTGGTTAGGAGCAGTAAGGCGGTGAGCCCCCACAGCCTATCCAGTGCTCTACCCCCGAGGGTATTCAGTTAACGCT

>B488_12000|1309741..1309642|Liberibacter_crescens_BT-1_EZ-Tn5_Transposon_Disruption

GTCCACAACTTACTATGAAAACTATAAAAAATACCCTCATAAAAGATTTCTGCATATAAATGTCCTTATTTTATTACTCGTCCATTTTTGACATCATAAA

>Intergenic_Region|329143..329044|Liberibacter_crescens_BT-1_EZ-Tn5_Transposon_Disruption

CTAGTACTCCTCGGTATTCGGAGTTTGGTTAGGAGCAGTAAGGCGGTGAGCCCCCATAGCCTATCCAGTCCTCTACCCCCGAGGGTATTCAGTTAACGCT

>B488_11420|1239533..1239632|Liberibacter_crescens_BT-1_EZ-Tn5_Transposon_Disruption

CCAATATACTAAGGATGATAGGCAGAGATGCTCAAGCTTTAGCTGTTATGCAACAAGTAGCGATACTTCATCCACAAGATAGAAGTGTTCTTGCTGCTTA

>Intergenic_Region|525440..525360|Liberibacter_crescens_BT-1_EZ-Tn5_Transposon_Disruption

GCTATGCACTGGGTCAAAACAGCTCCTGCCGAAACAAGGATAGAGTTCAGGAAACCCTTGCGCAGTCTGCCGCAGAACAATAATACAAGGGGTGTTATGA

>B488_11810|1288935..1288836|Liberibacter_crescens_BT-1_EZ-Tn5_Transposon_Disruption

CCTTTAACTTTATGCCGGGTATGTGGTTATCGATTCCAATGTCCTGATTGTTCCTCTTGGCTTGTAGAACATCGTTCTTCGAATGACTTGCGCTGTCATC

>B488_12820|1398381..1398282|Liberibacter_crescens_BT-1_EZ-Tn5_Transposon_Disruption

GTCCATTACCACGGATATCTATTCATGCATTTTGTGATACTGAAACATTATATAATGTTATAGAAAAATCTAAAGTAGATCGTCGTATGGCTCGGGTTAA

>Intergenic_Region|329143..329044|Liberibacter_crescens_BT-1_EZ-Tn5_Transposon_Disruption

CTAGTACTCCTCGGTATTCGGAGTTTGGTTAGGAGCAGTAAGGCGGTGAGCCCCCATAGCCTATCCAGTGCTCTACCCCCGAGGGTATTCAGTTAACGCT

>B488_08370|914053..914152|Liberibacter_crescens_BT-1_EZ-Tn5_Transposon_Disruption

CCTTTACGCATTAAATAAAAAGGAGATTCAGGAAGCCAAAACCGTAAAGCAATCCCTATAAGAGACGGGAAAGTTATAAAGAAAAAAATATACCTCCAGG

>Intergenic_Region|329143..329044|Liberibacter_crescens_BT-1_EZ-Tn5_Transposon_Disruption

CTAGTACTCCTCGGTATTCGGAGTTTGGTTAGGAGCAGTAAGGCGGTGAGCCCCCATAGCCTATCCAGTGCTCTACCCCCGAGGGTATTCAGTTAACGCT

>B488_11900|1300196..1300096|Liberibacter_crescens_BT-1_EZ-Tn5_Transposon_Disruption

GTTGTATATAACACGGTACAGCAATGCTTCCTTTTCAGATACAGTATAACGTGATCATGCTTGGAGTTTTTTGCACGTGTCAGATCAGTTTTTTCTAAGC

>Intergenic_Region|329143..329044|Liberibacter_crescens_BT-1_EZ-Tn5_Transposon_Disruption

CTAGTACTCCTCGGTATTCGGAGTTTGGTTAGGAGCAGTAAGGCGGTGAGCCCCCATAGCCTATCCAGTGCTCTACCCCCGAGGGTATTCAGTTAACGCT

>Intergenic_Region|1390323..1390427|Liberibacter_crescens_BT-1_EZ-Tn5_Transposon_Disruption

GTATAAAATCTACTGAATTGGAATAAACCTCTCCCTCCTAGAGAGCCCCCTAATCCAAAACCATACTCCCCTTCATAAAATACCCTAATAAATAATAAAC

>Intergenic_Region|329143..329044|Liberibacter_crescens_BT-1_EZ-Tn5_Transposon_Disruption

CTAGTACTCCTCGGTATTCGGAGTTTGGTTAGGAGCAGTAAGGCGGTGAGCCCCCATAGCCTATCCAGTGCTCTACCCCCGAGGGTATTCAGTTAACGCT

>B488_13090|1420404..1420301|Liberibacter_crescens_BT-1_EZ-Tn5_Transposon_Disruption

CTGGAAGCGGGCGCGATTTACGGTGGGCTGATTTTTGTCCCGGCTCTCGCGGTGTGTGGAATCGACCCGCCCGCCCGTGAGGCCGGCGCTCGCTGCTTAC

>Intergenic_Region|1480543..1480444|Liberibacter_crescens_BT-1_EZ-Tn5_Transposon_Disruption

ATACGATACTTAGCTTAACGAGCTAAGGTAACATTGTTTTTTCAATTCAGCCGTATTTTCTTCTTAAAGAATGAGATTTATTGTACAGAAATTCATAAAA

>B488_12300|1345941..1345842|Liberibacter_crescens_BT-1_EZ-Tn5_Transposon_Disruption

GGATTACGGTGGTCAGATCGAGCAGAAGATTATCGTACTGAAGCGTTAGGTCTGGTAAAGGCACAAAGAGTTAAGAATGCAGTGATTGTTCCTGTGGGAG

>Intergenic_Region|329143..329044|Liberibacter_crescens_BT-1_EZ-Tn5_Transposon_Disruption

CTAGTACTCCTCGGTATTCGGAGTTTGGTTAGGAGCAGTAAGGCGGTGAGCCCCCATAGCCTATCCAGTGCTCTACCCCCGAGGGTATTCAGTTAACGCT

>B488_11570|1257468..1257567|Liberibacter_crescens_BT-1_EZ-Tn5_Transposon_Disruption

AGATTAGGCTCAACAATACTTGATATATTTGCGACACCATCAAGCATACATAATGCTCCTACAAGTGAATTTAAGTTATTGCGCTCGTCTCCAATTTGAA

>Intergenic_Region|329143..329044|Liberibacter_crescens_BT-1_EZ-Tn5_Transposon_Disruption

CTAGTACTCCTCGGTATTCGGAGTTTGGTTAGGAGCAGTAAGGCGGTGAGCCCCCATAGCCTATCCAGTGCTCTACCCCCGAGGGTATTCAGTTAACGCT

>B488_09530|1025354..1025255|Liberibacter_crescens_BT-1_EZ-Tn5_Transposon_Disruption

GAGTAATCTTGAGCAGAAAATGGCCAAGATTGATGTACAATATGTCGATACTGATGATGGGAAGTTACATACAAAAACTTTTGATCTCGCAACTGCAAGT

>Intergenic_Region|329127..329053|Liberibacter_crescens_BT-1_EZ-Tn5_Transposon_Disruption

CTAGTCCACCTCATCCTTCGGAGTTTGGTTAGGAGCAGTAAGGCCGTGAGCCCCCCTATCCTATCCAGTGCTCTGACCCCTACGGTATTCAATTTATTGT

>Intergenic_Region|329143..329044|Liberibacter_crescens_BT-1_EZ-Tn5_Transposon_Disruption

CTATTACTCCTCGGTATTCGGAGTTTGGTTAGGATCATTAAGGCGGTGAGCCCCCGTAGCCTATCCAGTGCTCTACCCCCGAGGGTATTCAGTTAACGCT

>B488_05530|599929..600028|Liberibacter_crescens_BT-1_EZ-Tn5_Transposon_Disruption

CTCTTGACTACTCCTTTGATGTTCTGTCTTCTTTCCCCCCTTCAGCCATTCTTTAATACCATGTTCAATGTTATTTGTAGCCTCTTTCCTGGAGCAATAT

>B488_12250|1339237..1339138|Liberibacter_crescens_BT-1_EZ-Tn5_Transposon_Disruption

CTCTAATGTAGGACATCGATTATTCAGAAATGCTAACGACGGTTTTTTTAGCGGTATTTTAATCGGTTTGTTAATCGGGGTAGTCTGGACTCCCTGTGCA

>Intergenic_Region|418388..418293|Liberibacter_crescens_BT-1_EZ-Tn5_Transposon_Disruption

GGTTACAGACTTACCCATTATAGATCCTTGCCTATATTATTCAACCTTGAAACATCTGAAGAAGAGGTTCGCTATACACAGAAACCCTTATAAACTTCGT

>B488_05420|586264..586363|Liberibacter_crescens_BT-1_EZ-Tn5_Transposon_Disruption

GTGTAGTATAGGAGACAATGTCTCCAGAGGTTATAGCCCAATATTGCTCCAGGAAGAAAGACGCTGTAAATAACAGTGAACATGCTACTCAAGAGCGGCT

>Intergenic_Region|430816..430915|Liberibacter_crescens_BT-1_EZ-Tn5_Transposon_Disruption

CTCCTGATCAGAACCAGGATTCTGTGGTGACTCCTGTTCATGTCCAAACTTCTCCTAGCGAAGTTGTTAATGCCAAAACTCTTTCTCCTGACGAAGGTGT

>Intergenic_Region|329143..329044|Liberibacter_crescens_BT-1_EZ-Tn5_Transposon_Disruption

CTAGTACTCCTCGGTATTCGGAGTTTGGTTAGGAGCAGTAAGGCGGTGAGCCCCCATAGCCTATCCAGTGCTCTACCCCCGAGGGTATTCAGTTAACGCT

>B488_08070|879678..879579|Liberibacter_crescens_BT-1_EZ-Tn5_Transposon_Disruption

GAATATTTCTAATGATTTTGTAATATTATTATCAGCCATATTACGATCGCCTTTACACCTTACACGACATTATATGTGAACATACACTTATTTATATGAA

>B488_01840|224438..224339|Liberibacter_crescens_BT-1_EZ-Tn5_Transposon_Disruption

CAATTATACAGTTCGCATAAAAAATAAAATTAGCCTTGATGAAAAAACATAAAGACAGCTTTTGCTATAAAATCTCTCTAATAGTTCTCTGAAAAGTTTT

>Intergenic_Region|418388..418289|Liberibacter_crescens_BT-1_EZ-Tn5_Transposon_Disruption

GGTTACAGACTTACCCATTATAGATCCTTGCCTATATTATTCAACCTTGAAACATCTGAAGAAGAAGTTGCTATACACAGAAACGCTTATAAACTTATTG

>B488_12300|1345941..1345842|Liberibacter_crescens_BT-1_EZ-Tn5_Transposon_Disruption

GGATTACGGTGGTCAGATCGAGCAGAAGATTATCGTACTGAAGCGTTAGGTCTGGTAAAGGCACAAAGAGTTAAGAATGCAGTGATTGTTCCTGTGGGAG

>Intergenic_Region|329143..329044|Liberibacter_crescens_BT-1_EZ-Tn5_Transposon_Disruption

CTAGTACTCCTCGGTATTCGGAGTTTGGTTAGGAGCAGTAAGGCGGTGAGCCCCCATAGCCTATCCAGTGCTCTACCCCCGAGGGTATTCAGTTAACGCT

>B488_01000|116614..116713|Liberibacter_crescens_BT-1_EZ-Tn5_Transposon_Disruption

ATATACTTCTAAAGAAGTTACCGCTTATACTATTAACATTTTAAAAGATGATATCTCTTTAGCTGTTGATATACTTGGAGATATGTTAAGTAATTCTTTA

>Intergenic_Region|329143..329044|Liberibacter_crescens_BT-1_EZ-Tn5_Transposon_Disruption

CTAGTACTCCTCGGTATTCGGAGTTTGGTTAGGAGCAGTAAGGCGGTGAGCCCCCATAGCCTATCCAGTGCTCTACCCCCGAGGGTATTCAGTTAACGCT

>B488_11900|1300188..1300287|Liberibacter_crescens_BT-1_EZ-Tn5_Transposon_Disruption

ATATACAACGATCCACGTCGCTTTGGATTCATGGATCTAGTAGTTAGCTCCTTAGTGAATGAATATCCTTCCTTTGTAAATCTAGGACCAGAACCACTCG

>B488_07300|814234..814333|Liberibacter_crescens_BT-1_EZ-Tn5_Transposon_Disruption

CATCAACTCTATGGAAACCCAGGAACAACCAAGCGATGAAAATTCACACGTAAACTATCTTGTACATGTAGAGTTAATGGGAGAATTAAATGTTAATCAA

>Intergenic_Region|329143..329044|Liberibacter_crescens_BT-1_EZ-Tn5_Transposon_Disruption

CTAGTACTCCTCGGTATTCGGAGTTTGGTTAGGAGCAGTAAGGCGGTGAGCCCCCATAGCCTATCCAGTGCTCTACCCCCGAGGGTATTCAGTTAACGCT

>B488_10830|1180545..1180446|Liberibacter_crescens_BT-1_EZ-Tn5_Transposon_Disruption

ACATAATTCCTTGTAAATTGATGGTATTATCACAAGCAAGGCAGTTTCAGGGCTGTCACTAAAAGTTGTTTTTTCTCCAAGTATGGATAAACTAGGTGTA

>B488_10830|1180545..1180446|Liberibacter_crescens_BT-1_EZ-Tn5_Transposon_Disruption

ACATAATTCCTTGTAAGTTGATGGTATTTTCTCAAGCAATGCAGTTTCAGGGCTGTCACTAAAAGTTGTTTTTTCTCCAAGTATGGATAAACTAGGTGTA

>B488_08140|884085..884184|Liberibacter_crescens_BT-1_EZ-Tn5_Transposon_Disruption

TCTGGGAGTAGGAAAGATTTTACGTCAATACGGTGCTGGTTCTGTGAGTGAATGCGACTGGTGGGATGAGGTGTCAATGATGGGATTGAAACTACATTGT

>B488_01350|167438..167339|Liberibacter_crescens_BT-1_EZ-Tn5_Transposon_Disruption

ACTTGCCCCTACGAGAGTTTATTGCTTCTGAAATGAATGAAATAGGAATCTCCTGTAGCGCAGCAAATATTATTATTACATCTGGATCTCAGCAAGCCCT

>Intergenic_Region|329143..329044|Liberibacter_crescens_BT-1_EZ-Tn5_Transposon_Disruption

CTAGTACTCCTCGGTATTCGGAGTTTGGTTAGGAGCAGTAAGGCGGTGAGCCCCCATAGCCTATCCAGTGCTCTACCCCCGAGGGTATTCAGTTAACGCT

>B488_01000|117248..117149|Liberibacter_crescens_BT-1_EZ-Tn5_Transposon_Disruption

GAGTTGATAAGATCGAATAACATAAACTACGCCGTTCACGAATTTCTTGATATAAACGCGAAGACATACCTCCACCACCAAGAATATTATCCAAAATTAA

>Intergenic_Region|329143..329044|Liberibacter_crescens_BT-1_EZ-Tn5_Transposon_Disruption

CTAGTACTCCTCGGTATTCGGAGTTTGGTTAGGAGCAGTAAGGCGGTGAGCCCCCATAGCCTATCCAGTGCTCTACCCCCGAGGGTATTCAGTTAACGCT

>B488_12000|1309741..1309642|Liberibacter_crescens_BT-1_EZ-Tn5_Transposon_Disruption

GTCCACAACTTACTATGAAAACTATAAAAAATACCCTCATAAAAGATTTCTGCATATAAATGTCCTTATTTTATTACTCGTCCATTTTTGACATCATAAA

>B488_13300|1443578..1443479|Liberibacter_crescens_BT-1_EZ-Tn5_Transposon_Disruption

ATAACAAGTATTGGAAATATTGTTGAGATTAAAGCTGTTGCACGAAGTTTCCTTCATCATCAAATACGTTCTTTTGTTGGTAGTTTGAAATTGGTAGGAG

>B488_07040|782568..782667|Liberibacter_crescens_BT-1_EZ-Tn5_Transposon_Disruption

CTCGACCGCGAAACCGCCCGGATTGATGCGCTGATTGGCAAAACGGAGCAAAGTATCACTCTGCTCAAAGAACGCCGCGCCGCCTTTATTACTGCCGCTG

>Intergenic_Region|329143..329044|Liberibacter_crescens_BT-1_EZ-Tn5_Transposon_Disruption

CTAGTACTCCTCGGTATTCGGAGTTTGGTTAGGAGCAGTAAGGCGGTGAGCCCCCATAGCCTATCCAGTGCTCTACCCCCGAGGGTATTCAGTTAACGCT

>B488_12950|1406276..1406174|Liberibacter_crescens_BT-1_EZ-Tn5_Transposon_Disruption

AACAGGCACTGCTGGAAGCCCCTGATTTAGAACACGTGCACGATCCTAATCGTCTTATGCAGACACTCCGCACATAATTTTACGGCATAAGAAGTACTAC

>Intergenic_Region|329493..329592|Liberibacter_crescens_BT-1_EZ-Tn5_Transposon_Disruption

GTGAGAGACCCTCTCGCCGAAAGACCAAGGGTTCCTGCTTAAAGTTAATCTGAGCAGGGTTAGCCGGCCCCTAAGGCGAGGCGGACACGCGTAGTCGATG

>Intergenic_Region|329143..329044|Liberibacter_crescens_BT-1_EZ-Tn5_Transposon_Disruption

CTAGTACTCCTCGGTATTCGGAGTTTGGTTAGGAGCAGTAAGGCGGTGAGCCCCCATAGCCTATCCAGTGCTCTACCCCCGAGGGTATTCAGTTAACGCT

>B488_06040|667728..667629|Liberibacter_crescens_BT-1_EZ-Tn5_Transposon_Disruption

ATTTAATCCAATAAACAAATCTGGGTTTTCACGTTTTATTTCATAAATTATTTCATAATTCAAAGGAGGAACTTTACGATTTTCACTCGGTGACAATCCT

>B488_06040|667728..667629|Liberibacter_crescens_BT-1_EZ-Tn5_Transposon_Disruption

ATTTAATCCAATAAACAAATCTGGGTTTTCACGTTTTATTTCATAAATTATTTCATAATTCAAAGGAGGAACTTTACGATTTTCACTCGGTGACAATCCT

>Intergenic_Region|329143..329044|Liberibacter_crescens_BT-1_EZ-Tn5_Transposon_Disruption

CTATTACTCCTCGGTATTCGGAGTTTGGTTAGGAGCAGTAAGGCGGTGAGCCCCCATAACCTATCCTGTGCTCTACCCCCGAGGGTATTCAGTTAACGCT

>Intergenic_Region|418388..418289|Liberibacter_crescens_BT-1_EZ-Tn5_Transposon_Disruption

GGTTACAGACTTACCCATTATAGATCCTTGCCTATACTATTCAACCTTGAAACATCTGAAGAAGAAGTTGCTATACACAGAAACCCTTATAAACTTATTG

>B488_10700|1167970..1167871|Liberibacter_crescens_BT-1_EZ-Tn5_Transposon_Disruption

CAGCAGCTGTTCGCGATGCATTGAATGGTATTGAGTTTATTCGTCGTTCTGAAGATATTTTTGGACAAAAAATATTAGTTCTTTCTGGACAAGAAGAAGC

>B488_10390|1135789..1135888|Liberibacter_crescens_BT-1_EZ-Tn5_Transposon_Disruption

CATATTGACACTCCTTAAAATATTTTTTGCAGAATTCATAATGACCTATAGGCTGAGAAGTTGGGCCTGAAATCAGCATTTTCGATTTTTCTTGAGCATT

>Intergenic_Region|330816..330915|Liberibacter_crescens_BT-1_EZ-Tn5_Transposon_Disruption

GAATGGATAACCGCTGAAAGCATCTAAGCGGGAAACCAACCTGAAAACGAGTATTCCCTATCAGAGCCGTGGAAGACTACCACGTTGATAGGCTGGGTGT

>Intergenic_Region|1480830..1480766|Liberibacter_crescens_BT-1_EZ-Tn5_Transposon_Disruption

GTATAAAATACCATTTTCATATGAACCCTGAAAACAGTCGTTTGTTAAAGCTTTATATAAAGTAATACAAGGGGTGTTATGAGCCATAATTCTTTTTATT

>Intergenic_Region|329143..329044|Liberibacter_crescens_BT-1_EZ-Tn5_Transposon_Disruption

CTAGTACTCCTCGGTATTCGGAGTTTGGTTAGGAGCAGTAAGGCGGTGAGCCCCCATAGCCTATCCAGTGCTCTACCCCCGAGGGTATTCAGTTAACGCT

>Intergenic_Region|329218..329317|Liberibacter_crescens_BT-1_EZ-Tn5_Transposon_Disruption

AGTCATGGCTAAGTGGGAAAGGATGTGAGGGTCCCAAAACAACCAGGATGTTGGCTTAGAAGCAGCCATCATTTAAAGAAAGCGTAACAGCTCACTGGTC

>B488_03030|350061..349962|Liberibacter_crescens_BT-1_EZ-Tn5_Transposon_Disruption

ATTTATATCTATCTCAAAAGTTCTCAGATCACGATTCGATTATTAATGCTTATCAATTTTGCTTTCCTAGTTAAAGCCCTCTCCAAATCTTTCTCATCAT

>Intergenic_Region|329143..329044|Liberibacter_crescens_BT-1_EZ-Tn5_Transposon_Disruption

CTAGTACTCCTCGGTATTCGGAGTTTGGTTAGGGGCAGTAAGGCGGTGAGCCCCCATAGCCTATCCAGTGCTCTACCCCCGAGGGTATTCAGTTAACGCT

>B488_06540|723407..723308|Liberibacter_crescens_BT-1_EZ-Tn5_Transposon_Disruption

GCTCTTCAGTGCAATAAACGCCTGTTTCTGTTCCTGGGACAACGGCAAGCACATTTTCAAAATTAATGTGATTTTTTATTTCTTCAGCGCTGTACAGAAT

>Intergenic_Region|505852..505923|Liberibacter_crescens_BT-1_EZ-Tn5_Transposon_Disruption

CAGATGATCCTTCAACTAGCAAAAGTTCGAATTATAATATGTCTGGCGTAATAAATGGTCAGGTAAATATGTTTTGGTATAAAAATTAAGTTATTAAACT

>Intergenic_Region|418388..418289|Liberibacter_crescens_BT-1_EZ-Tn5_Transposon_Disruption

GGTTACAGACTTACCCATTATAGATCCTTGCCTATATTATTCAACCTTGAAACATCTGAAGAAGAAGTTGCTATACACAGAAACCCTTATAAACTTGTTG

>Intergenic_Region|329143..329044|Liberibacter_crescens_BT-1_EZ-Tn5_Transposon_Disruption

CTAGTACTCCTCGGTATTCGGAGTTTGGTTAGGAGCAGTAAGGCGGTGAGCCCCCATAGCCTATCCAGTGCTCTACCCCCGAGGGTATTCAGTTAACGCT

>Intergenic_Region|418388..418333|Liberibacter_crescens_BT-1_EZ-Tn5_Transposon_Disruption

GGTTACAGACTTACCCATTATAGATCCTTGCCTATATTATTCATCCTTGAACCATCAGATTGCCTATTGCGATCCGTTTTCTCCCCTACAACCTGGCTGA

>Intergenic_Region|242782..242683|Liberibacter_crescens_BT-1_EZ-Tn5_Transposon_Disruption

ATATACATGGTTGTTATTTCTGAGCAGTTTTCTAATTATCTTTTAACTCTCGGTTATAAAAACTATATGATGTAGATCATAAAATATAATGTACATAATG

>B488_13560|1476387..1476486|Liberibacter_crescens_BT-1_EZ-Tn5_Transposon_Disruption

TCCCGTAACTGCGCTTGAGAAAAGAGCAAATTTAATGGAGAATAGTAAAATGTATACAATAGCGCTCGTAGATGACGATCGCAATATCCTTACCTCAGTA

>Intergenic_Region|329143..329044|Liberibacter_crescens_BT-1_EZ-Tn5_Transposon_Disruption

CTAGTACTCCTCGGTATTCGGAGTTTGGTTAGGAGCAGTAAGGCGGTGAGCCCCCATAGCCTATCCAGTGCTCTACCCCCGAGGGTATTCAGTTAACGCT

>B488_03680|420984..421081|Liberibacter_crescens_BT-1_EZ-Tn5_Transposon_Disruption

ACATAAAGCTGTATTGATGCTGCAAAAGAAGCTGATCTCTGGTTCCATCTGCTTATACAAGCATACTTCATGCTGATACGTCACCTTTCCTTCCTCGCTA

>B488_01000|116614..116713|Liberibacter_crescens_BT-1_EZ-Tn5_Transposon_Disruption

ATATACTTCTAAAGAAGTTATCACTTATACTATTAACATTTTAAAAGATGATATCTCTTTAGCTGTTGATATACTTGGAGATATGTTAAGTAATTCTTTA

>Intergenic_Region|329101..329046|Liberibacter_crescens_BT-1_EZ-Tn5_Transposon_Disruption

CGTTACCTACTTACCCATTTATAAATTCGGGAGGAGTTATTCAGCGGTGAGCCCCCATAGCCTATCCTTTGCTCTACCCCCGAGGGTATTCAGTTAACGG

>Intergenic_Region|329143..329044|Liberibacter_crescens_BT-1_EZ-Tn5_Transposon_Disruption

CTAGTACTCCTCGGTATTCGGAGTTTGGTTAGGAGCAGTAAGGCGGTGAGCCCCCATAGCCTATCCAGTGCTCTACCCCCGAGGGTATTCAGTTAACGCT

>B488_10240|1120292..1120391|Liberibacter_crescens_BT-1_EZ-Tn5_Transposon_Disruption

ATATCTAAGAACAAGAGAGAATGTGACTGATCATCAGGGATTAAAACATATCCATCAGAAATTGTCATAACGCCAACATGATTCCCTAACATAATATCTG

>B488_04780|531386..531485|Liberibacter_crescens_BT-1_EZ-Tn5_Transposon_Disruption

ACCTAGAATACCACCATAAGCACCTTCCCACGAGAAATTACCAGAATTCGAACCCGTGGGCTGTGACGCAGCATGAGGAATATTATCTACATTATTCACG

>B488_12830|1398506..1398605|Liberibacter_crescens_BT-1_EZ-Tn5_Transposon_Disruption

ACTGAGATGAAGTTGATGACCCGGATGAAGTTGATGACCCGGATGTACTTCCCGTACCAGAAGAAGTATTCTGACGATAAGTTTGCATAGTATTATTACG

>B488_07810|859042..859141|Liberibacter_crescens_BT-1_EZ-Tn5_Transposon_Disruption

TCACAATCCTGTGCTGATGGAGTGTTTTCATCTGCATTCCCTGTAAACCAGGAAGAAGGACAATCGTCATTTTTAATAACACGATCAGCATCATCATGAG

>Intergenic_Region|327264..327363|Liberibacter_crescens_BT-1_EZ-Tn5_Transposon_Disruption

CTACAATGGTGGTGACAATGGGCAGCGAAGTCGTGAGGCGGAGCTAATCTCCAAAAGCCATCTCAGTTCGGATTGCACTCTGCAACTCGAGTGCATGAAG

>Intergenic_Region|327272..327173|Liberibacter_crescens_BT-1_EZ-Tn5_Transposon_Disruption

CCATTGTAGCACGTGTGTAGCCCAGCCCATAAGGGCCATGAGGACTTGACGTCATCCCCACCTTCCTCCGGCTTATCGCCGGCAGTCCCCATAGAGTGCC

>B488_05550|594367..594268|Liberibacter_crescens_BT-1_EZ-Tn5_Transposon_Disruption

CTCTGAGCCAGCGGTCACTGATGATGCTACCATTGCCAGCAGTCCTGATACAGAAGAAATTCCCCCGGCGCCAGCCCCTCAGCACCTGCATTATCCCTAT

>Intergenic_Region|329143..329045|Liberibacter_crescens_BT-1_EZ-Tn5_Transposon_Disruption

CTAGTACTCCTCAGTATTATTGAGTTTGGTTAGGAGCAGTAAGGCGGTGAGCCCCCATAGCCTATCCAGTGCTCTACCCCCGAGGGTATTCAGTTAACGC

>B488_05550|594065..593966|Liberibacter_crescens_BT-1_EZ-Tn5_Transposon_Disruption

TAACAGAGCATGGATCTGTTGGCGATACAGAGGTGCATTCGCTTTTATACGGTTGGGACAAGTACTCAGCAGTAGAAATAGACGGTTATATCGATACTGT

>Intergenic_Region|329143..329044|Liberibacter_crescens_BT-1_EZ-Tn5_Transposon_Disruption

CTAGTACTCCTCGGTATTCGGAGTTTGGTTAGGAGCAGTAAGGCGGTGAGCCCCCATAGCCTATCCAGTGCTCTACCCCCGAGGGTATTCAGTTAACGCT

>B488_03000|348356..348455|Liberibacter_crescens_BT-1_EZ-Tn5_Transposon_Disruption

TTACAAAAGAGCAGGTGCCTAATCAATTTTTGGTTGATCCTGTTTTTGCTGTTACTGAAGAAGGGGGGACAACTGATGTTATTAATGGGCCTTTCGGGTC

>B488_13120|1424205..1424114|Liberibacter_crescens_BT-1_EZ-Tn5_Transposon_Disruption

GGGTATCACCACCCTTGGCCAATCTGTCAAGCTACATGCGCTGAGCGCCAGCATTGCTGCGCTGTTGCTGCTATTGGATGCCGCCTTACCCGAAATGGTC

>B488_05680|617471..617372|Liberibacter_crescens_BT-1_EZ-Tn5_Transposon_Disruption

TTCCAGGACACACACAAGCCGTTTTGGAAGCAGTAAAATCCTTGGTGTGATTCTTAATAGAAAAGAAGCTGATATATCTAAAAAACATAAGCCAACATCC

>Intergenic_Region|329122..329044|Liberibacter_crescens_BT-1_EZ-Tn5_Transposon_Disruption

ATACTACGCCGCGGTATTAAATAGTTTGGTTGGAGCAGTAAGGCGGTGAGCCCCCATAGCCTATCCCGTGCTCTACCCCCGAGGGTATTCATCTAACGCT

>Intergenic_Region|418388..418289|Liberibacter_crescens_BT-1_EZ-Tn5_Transposon_Disruption

GGTTACAGACTTACCCATTATAGATCCTTGCCTATATTATTCAACCTTGAAACATCTGAAGAAGAAGTTGCTATACACAGAAACCCTTATAAACTTATTG

>B488_08600|942901..942938|Liberibacter_crescens_BT-1_EZ-Tn5_Transposon_Disruption

ATCAAAACCAGCCTTTGCAGCTCTCATCATCCATTGGC

>B488_09920|1082577..1082478|Liberibacter_crescens_BT-1_EZ-Tn5_Transposon_Disruption

TCGCTATACACCTCAAGTTATAGGAGATTACATAGCAGGTTCTAATCATATATTGCCTACAGCAGGTTCTGCTCGGTTTTCATCAGGGTTAAATGTCTTG

>Intergenic_Region|329143..329044|Liberibacter_crescens_BT-1_EZ-Tn5_Transposon_Disruption

CTAGTACTCCTCGGTATTCGGAGTTTGGTTAGGAGCAGTAAGGCGGTGGGCCCCCATAGCCTATCCAGTGCTCTACCCCCGAGGGTATTCAGTTAACGCT

>B488_12950|1406276..1406177|Liberibacter_crescens_BT-1_EZ-Tn5_Transposon_Disruption

AACAGGCACTGCTGGAAGCCCCTGATTTTAGAACACGTGCACGAATACTAATTGCTATTATGCAGATAACTCTTGCACATAATTCTTATGGTTATAAGAA

>B488_07810|859042..859141|Liberibacter_crescens_BT-1_EZ-Tn5_Transposon_Disruption

TCACAATCCTGTGCTGATGGAGTGTTTTCATCTGCATTCCCTGTAAACCAGGAAGAAGGACAATCGTCATTTTTAATAACACGATCAGCATCATCATGAG

>B488_12820|1398373..1398472|Liberibacter_crescens_BT-1_EZ-Tn5_Transposon_Disruption

GTAATGGACGCATATTTTCAATATTTTCGACTTGAGCATTTTCTCTCAAATTAGAAGAAGTTTTATTTCGAATTTCATATTCAATAGAACTCATACCTAC

>Intergenic_Region|330194..330293|Liberibacter_crescens_BT-1_EZ-Tn5_Transposon_Disruption

GTGTAGGATAGGTGGTAGGCTTTGAAGCAAGGGCGCCAGCTTTTGTGGAGCCATCCTTGAAATACCACCCTTATCCACATGGATGTCTAACCGCGGTCCG

>Intergenic_Region|53059..53158|Liberibacter_crescens_BT-1_EZ-Tn5_Transposon_Disruption

GCAATAAATAAAGAAAAACTTGATATTTTTTATTTGCTGACACTCAATAACAAGGTGTTATAAATTGTCATTTATATCTTGTTGTGATTTTTAATATATA

>Intergenic_Region|329143..329044|Liberibacter_crescens_BT-1_EZ-Tn5_Transposon_Disruption

CTACTACTCCTCGTTCTTCAATAGTTTGGTTGGAGCAGTAAGGCGGAGAGCCCCCATAGCCTATCCAGTGCTCTACCCCCGAGGGTATTCAGTTAACGCT

>Intergenic_Region|330194..330293|Liberibacter_crescens_BT-1_EZ-Tn5_Transposon_Disruption

GTGTAGGATAGGTGGTAGGCTTTGAAGCAAGGGCGCCAGCTTTTGTGGAGCCATCCTTGAAATACCACCCTTATCCACATGGATGTCTAACCGCGGTCCG

>B488_03090|355169..355069|Liberibacter_crescens_BT-1_EZ-Tn5_Transposon_Disruption

GAACAGGGCAAAGCCATTTTTTATATAAAGGTGAATATGACACAATGCTTTTGATAGGCTTTGGTTTTATCACGGGTACTTTGCTGAGTCTCTTGCTAAT

>B488_10550|1149823..1149922|Liberibacter_crescens_BT-1_EZ-Tn5_Transposon_Disruption

ACCATTAACTATTTCAGCTATACCTTTTATAGCGCGATTAATTGAAACTTCCATTCGTGAAGTTAATATTGAGCTTGTTGAAACCGCACGTTCAATGGGA

>B488_00540|68479..68578|Liberibacter_crescens_BT-1_EZ-Tn5_Transposon_Disruption

GGAATAGTTTGTGCATCCGGAAACATATTTCGGCAAGCTGTATCAGAGTTGGCACCAAAATCACCCTGAAATGCGATTTTATTTTGTATTTTTTTCATAC

>B488_07050|783239..783140|Liberibacter_crescens_BT-1_EZ-Tn5_Transposon_Disruption

GTCATATGGTAGGCGGTCATTGCGGTATTGATCCATGGCGGCTTCGCAGGACTGGGTGAAGTCAGTCTTCAATTCGACCGTAGCCACAGGCAGGCCATTG

>Intergenic_Region|329114..329047|Liberibacter_crescens_BT-1_EZ-Tn5_Transposon_Disruption

CTTTTCCTCCTTACGTCATTTATAAGTTCGTGTAGGAGCAATTCGGCGGTGAGCCCCCATAGCCTATCCTTTGCTCTACCCCCGAGGGTATTCAGTTAAC

>B488_12750|1390351..1390252|Liberibacter_crescens_BT-1_EZ-Tn5_Transposon_Disruption

GGTTTATTCCAATTCAGTAGATTTTATACTCAAGAGAAGAAGAACTCATTTAATTTATATTTTTAAAACATTTTGAACAATTCCCTAATATCTCAATAGT

>Intergenic_Region|329143..329044|Liberibacter_crescens_BT-1_EZ-Tn5_Transposon_Disruption

CTAGTACTCCTCGGTATTCGGAGTTTGGTTAGGAGCAGTAAGGCGGTGAGCCCCCATAGCCTATCCAGTGCTCTACCCCCGAGGGTATTCAGTTAACGCT

>Intergenic_Region|329143..329044|Liberibacter_crescens_BT-1_EZ-Tn5_Transposon_Disruption

CTAGTACTCCTCGGTATTCGGAGTTTGGTTAGGAGCAGTAAGGCGGTGAGCCCCCATAGCCTATCCAGTGCTCTACCCCCGAGGGTATTCAGTTAACGCT

>B488_09380|1011037..1011136|Liberibacter_crescens_BT-1_EZ-Tn5_Transposon_Disruption

TATATAAGCAGGACCCAATCCAGAAACATTAGGATAAACGACATCCCATAAAAATGGGAGTATAATTATAGAAAACGCAATCGAAAAATATAAACGAACT

>B488_12250|1339237..1339138|Liberibacter_crescens_BT-1_EZ-Tn5_Transposon_Disruption

CTCTAATGTAGGACATCGATTATTCAGAAATGCTAGCGACGGTTTTTTTAGCGGTATTTTAATCGGTTTGTTAATCGGGGTAGTCTGGACTCCCTGTGCA

>Intergenic_Region|329143..329042|Liberibacter_crescens_BT-1_EZ-Tn5_Transposon_Disruption

CTAGTACTCCTCGGTATTCGGAGTTTGGTTAGGAGCAGTAAGGCGGTGAGCTCCATAGCCTATCCAGTGCTCTACCTCGAGGGTATTCAGTTAACGCTCT

>B488_03730|427876..427777|Liberibacter_crescens_BT-1_EZ-Tn5_Transposon_Disruption

ATATAATACTGAGCAGCATAATCAGAAATAGCTTGTTTATCAGAAAGACCTTTTTTCACAACCTCTGCCATCAAATCCAGATGAGATGCAAGAAACGCAA

>B488_07020|779123..779222|Liberibacter_crescens_BT-1_EZ-Tn5_Transposon_Disruption

TATCTGGACCATCCATTGGCTGCAATAAAAATCTTTTAAAGTCAAGAGAAAGGTATCGTTCTGGTGGAGATTCTTTTTGTGGAAAAACAAGTTTTAATTC

>Intergenic_Region|329143..329044|Liberibacter_crescens_BT-1_EZ-Tn5_Transposon_Disruption

CTAGTACTCCTCGGTATTCGGAGTTTGGTTAGGAGCAGTAAGGCGGTGAGCCCCCATAGCCTATCCAGTGCTCTACCCCCGAGGGTATTCAGTTAACGCT

>B488_12160|1328935..1329034|Liberibacter_crescens_BT-1_EZ-Tn5_Transposon_Disruption

CTCGCATACTGGATAATTCATTAAAAAGAACTCGCAAAACATATTCTGTTACTTCATTACAACGATCTATGCTATGATTTCCGTCCATAAGAACCTCAGG

>B488_13240|1438016..1438115|Liberibacter_crescens_BT-1_EZ-Tn5_Transposon_Disruption

GATGAACTTGTTTCTATGATACGAGGGGTAAAAGCTCTTGGTCTTGAAACATGTATGACTCTTGGTATGTTATCATTAGAACAAGCGCAAATTTTAGCTG

>B488_01000|116614..116713|Liberibacter_crescens_BT-1_EZ-Tn5_Transposon_Disruption

ATATACTTCTAAAGAAGTTACCGCTTATACTATTAACATTTTAAAAGATGATATCTCTTTAGCTGTTGATATACTTGGAGATATGTTAAGTAATTCTTTA

>Intergenic_Region|329143..329044|Liberibacter_crescens_BT-1_EZ-Tn5_Transposon_Disruption

CTAGTACTCCTCGGTATTCGGAGTTTGGTTAGGAGCAGTAAGGCGGTGAGCCCCCATAGCCTATCCAGTGCTCTACCCCCGAGGGTATTCAGTTAACGCT

>Intergenic_Region|329143..329044|Liberibacter_crescens_BT-1_EZ-Tn5_Transposon_Disruption

CTAGTACTCCTCGGTATTCGGAGTTTGGTTAGGAGCAGTAAGGCGGTGAGCCCCCATAGCCTATCCAGTGCTCTACCCCCGAGGGTATTCAGTTAACGCT

>B488_06980|776047..776146|Liberibacter_crescens_BT-1_EZ-Tn5_Transposon_Disruption

TTCTTACTCCGTATCAAGGCGGCAATACCCAATCCTGTTCCTGGGCCAAGAATGACCCGGGATGCAAAATTATTCTTTTTTATATCAAAAACATTACAAT

>B488_02290|263341..263440|Liberibacter_crescens_BT-1_EZ-Tn5_Transposon_Disruption

ATGCTGCCTGATGTTCTTTCAGCTTCTCTTATAGATCGTGCTTCTAAAATTGTTCATCTTGCCTTGAAATCTGGTGCTGATGCTGCTGATGTAAAGGTTC

>B488_01760|212975..212876|Liberibacter_crescens_BT-1_EZ-Tn5_Transposon_Disruption

CTCTAGTACAATCTCAATTCTATGTTCTGATGGTACATTTATTCCCGTATCAGTTCGTTTACATTCAATATTGTGGGAAGGACAAAAGTTATTGAGCCTA

>B488_05440|588564..588465|Liberibacter_crescens_BT-1_EZ-Tn5_Transposon_Disruption

CTCTACGTAGGCATAATATTTCGATTCGTAGCAATACAGACTATTGCCTTTTTTATCCGGATTTGTCTTTGTAATCTCTTCCTGAATGCCAATCGATGGG

>B488_05620|610370..610469|Liberibacter_crescens_BT-1_EZ-Tn5_Transposon_Disruption

GTCTGGGTTACCGCGTTCGGCTCTCCCTTGTAGCGGCAACCATGGGAATGTATCTTGGTCGTCGGTTTCGTATTCCAGCTGGTGCCTGTTGATGCCAATG

>B488_09430|1014937..1014838|Liberibacter_crescens_BT-1_EZ-Tn5_Transposon_Disruption

GGATAGGGCCATGTATCATTCCTGTTATGAGGAAAATATGCAAAGCTCGATGGTTGAAGCCAGGAAACGTGAGCGTTTGATACTTGATCGGTCTATATCT

>B488_06000|662276..662375|Liberibacter_crescens_BT-1_EZ-Tn5_Transposon_Disruption

TTTATCTACCGATCATCAAAGGATGGTAACACATGTTACAACAATGACAGAAAAAGAACGTTGCGAGGAGGTTGCCAGAATGTTGGCTGGCACTTATATA

>Intergenic_Region|329143..329044|Liberibacter_crescens_BT-1_EZ-Tn5_Transposon_Disruption

CTAGTACTCCTCGGTATTCGGAGTTTGGTTAGGAGCAGTAAGGCGGTGAGCCCCCATAGCCTATCCAGTGCTCTACCCCCGAGGGTATTCAGTTAACGCT

>B488_06660|738844..738943|Liberibacter_crescens_BT-1_EZ-Tn5_Transposon_Disruption

GGCCAGGAACAGAGGTAATGGCTCCACCAGTCGCTAAGGGAAGCAAAAAACCATTGCCCAAGGTGAGAAGCGAAACAGCACTAATACAGGAAAAGAAGGA

>B488_02440|279940..279841|Liberibacter_crescens_BT-1_EZ-Tn5_Transposon_Disruption

AACATCCACAAAAGCTTTTTTAGCAGCAGAAGCTTCTTTCATTACGATTAAAACAGAGACTTCTGTATCCTGATCATCAGAAATTTCATCTTTACGTGTT

>B488_08300|906575..906674|Liberibacter_crescens_BT-1_EZ-Tn5_Transposon_Disruption

ATGTTCCCCTGAACAAAAAAGAATGTGTATTATCTGTCTCTAATATGACTGTTGTTCTGAATAAAAAAACAATATTTAAAAATCTTGATCTTCAGGTATA

>Intergenic_Region|330816..330915|Liberibacter_crescens_BT-1_EZ-Tn5_Transposon_Disruption

GAATGGATAACCGCTGAAAGCATCTAAGCGGGAAACCAACCTGAAAACGAGTATTCCCTATCAGAGCCGTGGAAGACTACCACGTTGATAGGCTGGGTGT

>Intergenic_Region|329143..329042|Liberibacter_crescens_BT-1_EZ-Tn5_Transposon_Disruption

CTAGTACTCCTCGGTATTCGGAGTTTGGTCGGAGCAGTAAGGCGGTGAGTCCCATAACCTATCCAGTGCTCTACCCCCGAGGGTATTCAGTTAACGCTCT

>B488_10290|1126061..1126160|Liberibacter_crescens_BT-1_EZ-Tn5_Transposon_Disruption

ATCCATAACTAAACTTTCTCAACTTCAGGGCGCCCTAAAATACCTGTTGGCTTAAACAGCAACACTAAAATAAGTATAGAAAAAGTTGCAATATCTTTAT

>B488_10290|1126061..1126160|Liberibacter_crescens_BT-1_EZ-Tn5_Transposon_Disruption

ATCCATAACTAAACTTTCTCAACTTCAGGGCGCCCTAAAATACCTGTTGGCTTAAACAGCAACACTAAAATAAGTATAGAAAAAGTTGCAATATCTTTAT

>B488_06980|776408..776309|Liberibacter_crescens_BT-1_EZ-Tn5_Transposon_Disruption

ACACTAAACTTTGACACTTTAGAGCAAGCTATTCAACAGACAGTTCTTGAGAAAGTATCCTGTCAACCTAGTTCTTCTATCCTTGCTGTTGCTGGACCTG

>Intergenic_Region|329468..329567|Liberibacter_crescens_BT-1_EZ-Tn5_Transposon_Disruption

GTTGACATGAGTAACGATAAAGAGGGTGAGAGACCCTCTCGCCGAAAGACCAAGGGTTCCTGCTTAAAGTTAATCTGAGCAGGGTTAGCCGGCCCCTAAG

>Intergenic_Region|329476..329377|Liberibacter_crescens_BT-1_EZ-Tn5_Transposon_Disruption

CATGTCAACTTTCTCACTTCCGATACCTCCAAAGGTCCTCACAGATCCTTCTTCTTCGGCTTACGGAACGCTCCGCTACCAATTGTCTTTAAAACAAATT

>B488_07040|782568..782667|Liberibacter_crescens_BT-1_EZ-Tn5_Transposon_Disruption

CTCGACCGCGAAACCGCCCGGATTGATGCGCTGATTGGCAAAACGGAGCAAAGTATCACTCTGCTCAAAGGACGCCACGCCGCCTTTATTACTGCCGCTG

>Intergenic_Region|329143..329044|Liberibacter_crescens_BT-1_EZ-Tn5_Transposon_Disruption

CTAGTACTCCTCGGTATTCGGAGTTTGGTTAGGAGCAGTAAGGCGGTGAGCCCCCATAGCCTATCCAGCGCTCTACCCCCGAGGGTATTCAGTTAGCGCT

>B488_01350|167618..167717|Liberibacter_crescens_BT-1_EZ-Tn5_Transposon_Disruption

CGGTTCAAGCGTGTAGAAAACTTTTCTTCCCAATTCAACATAGAAAAATTTCCTTATTAGTGTTTTCTGCGCTGCTTTATAATTTTATAATAATCTATAT

>B488_01350|167621..167531|Liberibacter_crescens_BT-1_EZ-Tn5_Transposon_Disruption

CTAGACCGTATGTAGTCAATTGAAATTCGGGATCTTTTGAAAATTATAACGCCCCGATATCATTTCATTTGCTGGAGGAATTCTTGATGTAAACAAAAAC

>Intergenic_Region|329143..329044|Liberibacter_crescens_BT-1_EZ-Tn5_Transposon_Disruption

CTAGTACTCCTCGGTATTCGGAGTTTGGTTAGGAGCAGTAAGGCGGTGAGCCCCCATAGCCTATCCAGTGCTCTACCCCCGAGGGTATTCAGTTAACGCT

>B488_04410|495228..495327|Liberibacter_crescens_BT-1_EZ-Tn5_Transposon_Disruption

GTTATGTTCGTATTGCATTAGTTGAAAACGAACATCGTATTCGTCAAGCCGCTCGTAATTTAAAGATTTTTTTTTCTGAATCTAACAGTAATTTTCAAAT

>Intergenic_Region|329143..329045|Liberibacter_crescens_BT-1_EZ-Tn5_Transposon_Disruption

CTAGTACTGTATCGGTATTCGGAGTTTGGTTAGGAGCAGTGAGGCGGTGAGCCCCCATAGCCTATCCAGTGCTCTAACCCCGAGGGTATTCAGTTAACGC

>B488_00650|80024..79925|Liberibacter_crescens_BT-1_EZ-Tn5_Transposon_Disruption

AATTAGTACAAAAACGCTCTTCTCCATTATAATCAATATAGGTACCAGTATTTTCATTAAATGAATTGTATCTCCTGTAACAATACATACGCCACTCATC

>B488_03940|456982..456883|Liberibacter_crescens_BT-1_EZ-Tn5_Transposon_Disruption

GCCATGCATGTACATCAATCTGTGTTAGATAGTAATGGGTACAATATCTTTTCAAATCAAGATGGTTCCGAAACAGATATGTTTAAATATTTTATCGGAG

>B488_13260|1439807..1439906|Liberibacter_crescens_BT-1_EZ-Tn5_Transposon_Disruption

GATGTCGGAAAAACAGTTTTCTCAGCTGCTCTCGCTAATGCATTGGATTCTTACTATTGGAAACCAATCCAGGCAGGCCTTGATAGCGAAACTGACAGCG

>B488_08300|908306..908207|Liberibacter_crescens_BT-1_EZ-Tn5_Transposon_Disruption

GCAGAACTTTCACGTGCATTGGCGATTGCTACTGTTATATTATCAACAGAACAACTGATTTTATTTTTATCTATTGTTGTTATTAAAGAATCAAAATGTT

>Intergenic_Region|329143..329044|Liberibacter_crescens_BT-1_EZ-Tn5_Transposon_Disruption

CTAGTACTCCTCGGTATTCGGAGTTTGGTTAGGAGCAGTAAGGCGGTGAGCCCCCATAGCCTATCCAGTGCTCTACCCCCGAGGGTATTCAGTTAACGCT

>B488_13370|1451615..1451516|Liberibacter_crescens_BT-1_EZ-Tn5_Transposon_Disruption

GGCTTACGCTATGATAGGTGGAATAATATCAGGAACATTGTTAACATTAACATTTTTCCCTGCTTTGTATGTGGTGTTGTTTCGTATTAAACCGTTTGAT

>B488_11490|1246874..1246775|Liberibacter_crescens_BT-1_EZ-Tn5_Transposon_Disruption

ATGTTGGTCTAGCAATTAAAGATCAGAATTTGCCAGCTGTAGGGTATGCTCTGTTAACAATGTTGTTAGTAATTATTTTTTATGATCAGTTGTTATTTCG

>Intergenic_Region|418388..418289|Liberibacter_crescens_BT-1_EZ-Tn5_Transposon_Disruption

GGTTACAGACTTACCCATTATAGATCCTTGCCTATATTATTCAACCTTGAAACATCTGAAGAAGAAGTTGCTATACACAGAAACCCTTATAAACTTATTG

>Intergenic_Region|329143..329044|Liberibacter_crescens_BT-1_EZ-Tn5_Transposon_Disruption

CTAGTACTCCTCGGTATTCGGAGTTTGGTTAGGAGCAGTAAGGCGGTGAGCCCCCATAGCCTATCCAGTGCTCTACCCCCGAGGGTATTCAGTTAACGCT

>B488_06640|737248..737149|Liberibacter_crescens_BT-1_EZ-Tn5_Transposon_Disruption

GTTTCTACTTCTACTGGACGACCAGCACCACCTGCAGGAAACTGCAGTAACCATTTCCTTGTTCCGTCTTTAGAAATATTCTCGCTCATAATTTTTGGAT

>B488_05140|555998..555899|Liberibacter_crescens_BT-1_EZ-Tn5_Transposon_Disruption

GGATGTGGAATACCAGTTGGTGAAGATAGACCAGCTGTTGGTGAGAACGTTTAATACGAAATTTGTATACGGTAAAATATGACCGCTAAAGGATATAAGA

>Intergenic_Region|329143..329043|Liberibacter_crescens_BT-1_EZ-Tn5_Transposon_Disruption

CTAGTACTCCTCGGTATTCGGAGTTTGGTTAGGAGCATAAGGCGGTGAGCCCCCATAGCCTATCCAGTGCTCTACCCCCGAGGGTATTCAGTTAACGCTC

>Intergenic_Region|329143..329044|Liberibacter_crescens_BT-1_EZ-Tn5_Transposon_Disruption

CTAGTACTCCTCGGTATTCGGAGTTTGGTTAGGAGCAGTAAGGCGGTGAGCCCCCATAGCCTATCCAGTGCTCTACCCCCGAGGGTATTCAGTTAACGCT

>B488_11650|1270239..1270140|Liberibacter_crescens_BT-1_EZ-Tn5_Transposon_Disruption

GCCCTATTGTTGAAAGGAAGCGCTATAATTTTTATTGATGGCCGGTATAATCTTCAGGTGGAAGAAGAACTTGATATAACATTATTTTCTAAACTGGATC

>B488_04700|526581..526482|Liberibacter_crescens_BT-1_EZ-Tn5_Transposon_Disruption

GTTTCATACCGAGTCAACAAAGGAACTGGTCACGGTTACCTGTATCATTTCTCACAAGGATGGGCATCGTGAAAGAAACAGCCTTTCTGCTCCTCCTGAT

>Intergenic_Region|329143..329044|Liberibacter_crescens_BT-1_EZ-Tn5_Transposon_Disruption

CTAGTACTCCCCGGTATTCGGAGTTTGGTTAGGAGCAGTAAGGCGGTGAGCCCCCATAGCCTATCCAGTGCTCTACCCCCGAGGGTATTCAGTTAACGCT

>Intergenic_Region|329143..329044|Liberibacter_crescens_BT-1_EZ-Tn5_Transposon_Disruption

CTAGTACTCCTCGGTATTCGGAGTTTGGTTAGGAGCAGTAAGGCGGTGAGCCCCCATAGCCTATCCAGTGCTCTACCCCCGAGGGTATTCAGTTAACGCT

>B488_03370|388560..388659|Liberibacter_crescens_BT-1_EZ-Tn5_Transposon_Disruption

CTTTTAAAGAACAATAGGGTAGTTTCTGATAAGGAGAAACATCAAGTTTTTGAGCGTCCTTTTGCGGATTTAAAAGGAAAATTAGAAATGCCAGTTGATT

>B488_00630|77375..77276|Liberibacter_crescens_BT-1_EZ-Tn5_Transposon_Disruption

GGCCATGAAATAGCATTAAAGCAGCAATCCGGTTTTGGTGCTATATTCAGTTTCGAACTGTTTGGTGATGAAACAACTGTTAAGGCATTTCTTAAAAGCT

>B488_11650|1270239..1270139|Liberibacter_crescens_BT-1_EZ-Tn5_Transposon_Disruption

GCCCGATTGTTGAAAGGAAGCGCTATAATTTTTATTGATGGCCGGTATAATCTTCCCTGGAAGACCAACTTGATATGACATTATTTTCTAAACGGGATCT

>B488_05720|624388..624289|Liberibacter_crescens_BT-1_EZ-Tn5_Transposon_Disruption

AATGGCTACTTCAACAGAAGGAAGTGCAGGACCTTTGGGAGCAAGTTCAATTACCAAGAATAATGCCTTTAACTCTGTTACGTTTGGAGGAGGGAAAGAT

>Intergenic_Region|329143..329044|Liberibacter_crescens_BT-1_EZ-Tn5_Transposon_Disruption

CTAGTACTCCTCGGTATTCGGAGTTTGGTTAGGAGCAGTAAGGCGGTGAGCCCCCATAGCCTATCCAGTGCTCTACCCCCGAGGGTATTCAGTTAACGCT

>Intergenic_Region|329142..329044|Liberibacter_crescens_BT-1_EZ-Tn5_Transposon_Disruption

GTAGTACTCCTCGGTATTCGGAGTTTGGTTAGGAGCAGTAAGGCGGTGAGCCCCCATAACCTATCCTGTGCTCTACCCCCGAGGGTATTCAGTTAACGCT

>B488_03710|424538..424439|Liberibacter_crescens_BT-1_EZ-Tn5_Transposon_Disruption

CAGTAGTATTACCTATAGAAATACGAGTTTCTGCTCCTGGAGAAATCCTAAATGATGAAGACATTGTTGAAATTAAAGCGCCTGTTTCAGGAATAGCAAA

>Intergenic_Region|418388..418289|Liberibacter_crescens_BT-1_EZ-Tn5_Transposon_Disruption

GGTTACAGACTTACCCATTATAGATCCTTGCCTATATTATTCAACCTTGAAACATCTGAAGAAGAAGTTGCTATACACAGAAACCCTTATAAACTTATTG

>B488_11490|1246874..1246775|Liberibacter_crescens_BT-1_EZ-Tn5_Transposon_Disruption

ATGTTGGTCTAGCAATTAAAGATCAGAATTTGCCAGCTGTAGGGTATGCTTTGTTAACAATGTTGTTAGTAATTATTTTTTATGATCAGTTGTTATTTCG

>Intergenic_Region|418388..418289|Liberibacter_crescens_BT-1_EZ-Tn5_Transposon_Disruption

GGTTACAGACTTACCCATTATAGATCCTTGCCTATATTATTCAACCTTGAAACATCTGAAGAAGAAGTTGCTATACACAGAAACCCTTATAAACTTATTG

>B488_05670|616816..616717|Liberibacter_crescens_BT-1_EZ-Tn5_Transposon_Disruption

ATCACGACCTTGATCAACATAACTAAGATGCACGGTTTCACCAATTCGAATAACGCCTGAATCTGGCTGCTCATAGCCAGTAATAATCCGAAATAAAGTG

>Intergenic_Region|329143..329050|Liberibacter_crescens_BT-1_EZ-Tn5_Transposon_Disruption

CTAGTACTCCTCGGTATTCGGAGTTTGGTTAGGAGCAGTAAGGCGGTGAGCCCCCATAGCCTATCCAGTGCTCTACCCCCAAGGGTATTCGTTTAAGCCT

>B488_05690|618251..618350|Liberibacter_crescens_BT-1_EZ-Tn5_Transposon_Disruption

ATGTAGTAGGAGTGATCATTGCCCGAAGCCGAATCTCGAAAAATGAATACTGTATTTTCTTCTAGTCTTCGCTATAATAGCTTTTCATTGATGTTTAGAG

>Intergenic_Region|329143..329044|Liberibacter_crescens_BT-1_EZ-Tn5_Transposon_Disruption

CTAGTACTCCTCGGTATTCGGAGTTTGGTTAGGAGCAGTAAGGCGGTGAGCCCCCATAGCCTATCCAGTGCTCTACCCCCGAGGGTATTCAGTTAACGCT

>Intergenic_Region|329143..329044|Liberibacter_crescens_BT-1_EZ-Tn5_Transposon_Disruption

CTAGTACTCCTCGGTATTCGGAGTTTGGTTAGGAGCAGTAAGGCGGTGAGCCCCCATAGCCTATCCAGTGCTCTACCCCCGAGGGTATTCAGTTAACGCT

>B488_04960|544298..544199|Liberibacter_crescens_BT-1_EZ-Tn5_Transposon_Disruption

GTTATGGGAGCCAGCTTTACTTTACTCAGGTTTGGAGCACCTTTTCTAGGGCATTGATTCAGCGTCCGCCTGTCACTTTTGGAGCACAAATTCATTTGCC

>B488_05530|599547..599646|Liberibacter_crescens_BT-1_EZ-Tn5_Transposon_Disruption

GTATATGTTTATATGTGACATGACAGAAACAATTAATTTTTCATTTCTTGAAACATTCATTTTAAGCTTAATATTGTTCCTTTTCTACATGGGCATAATA

>B488_01760|213664..213744|Liberibacter_crescens_BT-1_EZ-Tn5_Transposon_Disruption

CAACATACGACTCCTTCTTAAATTCTGATAATGCCAAAGCCTCTAAAGCCATCCCATTTACGGTCGCGCGTGTATGTTGGAAGAGCATCAAAATCTATCG

>Intergenic_Region|329143..329044|Liberibacter_crescens_BT-1_EZ-Tn5_Transposon_Disruption

CTAGTACTCCTCGGTATTCGGAGTTTGGTTAGGAGCAGTAAGGCGGTGAGCCCCCATAGCCTATCCAGTGCTCTACCCCCGAGGGTATTCAGTTAACGCT

>B488_11830|1291771..1291672|Liberibacter_crescens_BT-1_EZ-Tn5_Transposon_Disruption

TCGTACTTTGAGCTAAAGCGATAGGAAATTGAATTATTATTATAATAAAAATAATGAATAAGGATAAAAAAGACACTCTGTCCGTTTTCATTTACGATCC

>B488_06660|738842..738743|Liberibacter_crescens_BT-1_EZ-Tn5_Transposon_Disruption

GGCTGGTTCTGCTTCTGGTTTTATGGGAGCTTTACAAATCGCTTCTGCAGCAGTGACAACTGCCTATATTGGTCAGTTTTCTCACCATCAGCCTGGCAGA

>B488_10940|1191364..1191265|Liberibacter_crescens_BT-1_EZ-Tn5_Transposon_Disruption

AAATAGTATATATTTAATTTTTAAGCTAATCTACTCGTTGATAACGATAATTTTATGAGCGTTGATGATATGTCTTCTCTGAATAATACAAATAAAGAGA

>B488_11650|1270239..1270140|Liberibacter_crescens_BT-1_EZ-Tn5_Transposon_Disruption

GCCCTATTGTTGAAAGGAAGCGCTATAATTTTTATTGATGGCCGGTATAATCTTCTCGTGGAACAACAACTTGATACAACATTATTTTCTAAACTGGATC

>B488_05020|545585..545684|Liberibacter_crescens_BT-1_EZ-Tn5_Transposon_Disruption

CATAAATACCTTAAGCGTTGGATTCTCTCTTCAAGTTGTTTACCACGCACCCATTTGTCTGGGTTTTTCTTATAAGAATCCATGATTTCTTTAATATCAA

>Intergenic_Region|329143..329044|Liberibacter_crescens_BT-1_EZ-Tn5_Transposon_Disruption

CTAGTACTCCTCGGTATTCGGAGTTTGGTTAGGAGCAGTAAGGCGGTGAGCCCCCATAGCCTATCCAGTGCTCTACCCCCGAGGGTATTCAGTTAACGCT

>Intergenic_Region|418388..418289|Liberibacter_crescens_BT-1_EZ-Tn5_Transposon_Disruption

GGTTACAGACTTACCCATTATAGATCCTTGCCTATATTATTCAACCTTGAAACATCTGAAGAAGAAGTTGCTATACACAGAAACCCTTATAAACTTATTG

>B488_01000|116614..116713|Liberibacter_crescens_BT-1_EZ-Tn5_Transposon_Disruption

ATATACTTCTAAAGAAGTTACCGCTTATACTATTAACATTTTAAAAGATGATATCTCTTTAGCTGTTGATATACTTGGAGATATGTTAAGTAATTCTTTA

>B488_05670|616816..616717|Liberibacter_crescens_BT-1_EZ-Tn5_Transposon_Disruption

ATCACGACCTTGATCAACATAACTAAGATGCACGGTTTCACCAATTCGAATAACGCCTGAATCTGGCTGCTCATAGCCAGTAATAATCCGAAATAAAGTG

>B488_00200|26689..26788|Liberibacter_crescens_BT-1_EZ-Tn5_Transposon_Disruption

ACATAGAAATGGTTATGGCACATCTTGTTGTACATGATGGGTGTATAGTCGATAATATAAAAAGCGAAACAAACAGACGCAATTTTATTGGTAGAGGTCG

>B488_06590|732233..732332|Liberibacter_crescens_BT-1_EZ-Tn5_Transposon_Disruption

ACGTTGAAGTTGGAATTTTATGGTCCATTCGGAAAAATACATCGGCGTATTGATGCTGTTGAAATTATAAATTATATATCTATGCGTTCTTATGAACAGA

>B488_05530|599547..599646|Liberibacter_crescens_BT-1_EZ-Tn5_Transposon_Disruption

GTATATGTTTATATGTGACATGACAGAAACAATTAATTTTTCATTTCTTGAAACATTCATTTTAAGCTTAATATTGTTCCTTTTCTACATGGGCATAATA

>Intergenic_Region|329143..329044|Liberibacter_crescens_BT-1_EZ-Tn5_Transposon_Disruption

CTAGTACTCCTCGGTATTCGGAGTTTGGTTAGGAGCAGTAAGGCGGTGAGCCCCCATAGCCTATCCAGTGCTCTACCCCCGAGGGTATTCAGTTAACGCT

>Intergenic_Region|329143..329044|Liberibacter_crescens_BT-1_EZ-Tn5_Transposon_Disruption

CTAGTACTCCTCGGTATTCGGAGTTTGGTTAGGAGCAGTAAGGCGGTGAGCCCCCATAGCCTATCCAGTGCTCTACCCCCGAGGGTATTCAGTTAACGCT

>B488_04960|544298..544199|Liberibacter_crescens_BT-1_EZ-Tn5_Transposon_Disruption

GTTATGGGAGCCAGCTTTACTTTACTCAGGTTTGGAGCACCTTTTCTAGGGCATTGATTCAGCGTCCGCCTGTCACTTTTGGAGCACAAATTCATTTGCC

>B488_00200|26689..26788|Liberibacter_crescens_BT-1_EZ-Tn5_Transposon_Disruption

ACATAGAAGTGGTTATGGCACATCTTGTTGTACATGATGGGTGTATAGTCGATAATATAAAAAGCGAAACAAACAGACGCAATTTTATTGGTAGAGGTCG

>Intergenic_Region|329143..329044|Liberibacter_crescens_BT-1_EZ-Tn5_Transposon_Disruption

CTAGTACTCCTCGGTATTCGGAGTTTGGTTAGGAGCAGTAAGGCGGTGAGCCCCCATAGCCTATCCAGTGCTCTACCCCCGAGGGTATTCAGTTAACGCT

>B488_06540|723308..723407|Liberibacter_crescens_BT-1_EZ-Tn5_Transposon_Disruption

ATTCTGTACAGCGCTGAAGAAATAAAAAATCACATTAATTTTGAAAATGTGCTTGCCGTTGTCCCAGGAACAGAAACAGGCGTTTATTGCACTGAAGAGC

>B488_10750|1173712..1173614|Liberibacter_crescens_BT-1_EZ-Tn5_Transposon_Disruption

CTTTACTTAGTTATCATTTATTTAGGAACGTACGAGATAACAAATCTCTATTCTGTAGTAAAGCGTGTGACACAGATTTCAAGCTCTATGGCAGATATGT

>B488_05030|547056..546957|Liberibacter_crescens_BT-1_EZ-Tn5_Transposon_Disruption

AAACCAAGTCAAGAGGCTGTCAATAAAATATTAAACGTCCTTACTCTTATATCCCCAATAAATAGCTAAATCTTCAAGACAGTTCTTAATATTGGATGCA

>B488_05170|558849..558750|Liberibacter_crescens_BT-1_EZ-Tn5_Transposon_Disruption

GCCTTGAATGTGTAAGCAGGTGTGCCCAGAGCCTGCTGAACCTGTGACCCCCGTTGGCCCGCAGAAGGCAACATCGGATTGTTCTGAAGAGCAGACATGA

>Intergenic_Region|329143..329044|Liberibacter_crescens_BT-1_EZ-Tn5_Transposon_Disruption

CTAGTACCCCTCGGTATTCGGAGTTTGGTTAGGAGCAGTAAGGCGGTGAGCCCCCATAGCCTATCCAGTGCTCTACCCCCGAGGGTATTCAGTTAACGCT

>Intergenic_Region|329143..329044|Liberibacter_crescens_BT-1_EZ-Tn5_Transposon_Disruption

CTAGTACTCCTCGGTATTCGGAGTTTGGTTAGGAGCAGTAAGGCGGCGAGCCCCCATAGCCTATCCAGTGCTCTACCCCCGAGGGTATTCAGTTAACGCT

>B488_05420|586312..586213|Liberibacter_crescens_BT-1_EZ-Tn5_Transposon_Disruption

GAGCAATATTGGGCTATAACCTCTGGAGACATTGTCTCCTATACTACACAAATCTAAAGGACGATGATAGAGAACGGTTACCTATCATTCCATCCTGGGA

>B488_12800|1395329..1395231|Liberibacter_crescens_BT-1_EZ-Tn5_Transposon_Disruption

GTTTAACATGCTAGCTTGTCTAAGTGCATCTTTTTTAACTGCTCTTGTTGTGCCGCGGTTTTTATTGAATTACTTAATAAAAAAACGTCAAGCTAAATTT

>Intergenic_Region|329140..329045|Liberibacter_crescens_BT-1_EZ-Tn5_Transposon_Disruption

CTTGTACTCCTTACGTATTCGGAGTTTGGTTAGGAGCAATAAGGCGGTGAGCCCCCTTAGCCTATCCTGTGCTCTACCCCCGAGGGTATTCAGTTAACGC

>Intergenic_Region|459384..459483|Liberibacter_crescens_BT-1_EZ-Tn5_Transposon_Disruption

GATCATGGGTTACTGTCTTCAGAGTGATTCTAGAGAAATAGAGATCTTACAGATGAAAAGCTGAGAATTTTTTTATAAATAACACTCAATTTATAGCTAA

>Intergenic_Region|329143..329044|Liberibacter_crescens_BT-1_EZ-Tn5_Transposon_Disruption

CTAGTACTCCTCGGTATTCGGAGTTTGGTTAGGAGCAGTAAGGCGGTGAGCCCCCATAGCCTATCCAGTGCTCTACCCCCGAGGGTATTCAGTTAACGCT

>B488_12940|1406087..1405988|Liberibacter_crescens_BT-1_EZ-Tn5_Transposon_Disruption

GGCTAATCTTATGTTATCAGCTGACGGTAAAGAGCTTATTTCAGAGAACGCATCCCTTGCATATCCAATTCGCAAAGGTATTCCTATTCTGCTTGCATCA

>B488_11600|1259887..1259986|Liberibacter_crescens_BT-1_EZ-Tn5_Transposon_Disruption

CTCCTGGACCACCCAGGAAACCAGTACCTCCTCGTTGCCGCTCAAGATGCGTCCAACTACGAACAAGACGACCTTTTTGATAATTAAGATGAGCTAATTC
[truncated: 34,785 more chars]
